# Direct *α*‐C(sp^3^)─H Alkylation and Arylation of Ethers Enabled by Carbon Nitride/Nickel Metallaphotoredox Catalysis

**DOI:** 10.1002/cssc.71111

**Published:** 2026-10-23

**Authors:** Miguel M. de Vries Ibáñez, Nathan G. G. Husy, Elisabetta Inico, Kaan Karaca, Milla Vigliengo, Andrea Olivati, Annamaria Petrozza, Carlo Cavallotti, Gianvito Vilé

**Affiliations:** ^1^ Department of Chemistry, Materials and Chemical Engineering “Giulio Natta” Politecnico di Milano Milano Italy; ^2^ Center for Nano Science and Technology Istituto Italiano di Tecnologia Milano Italy

**Keywords:** carbon materials, C(sp^3^)─H functionalization, green chemistry, photocatalysis, sustainable synthesis

## Abstract

The direct functionalization of *α*‐C(sp^3^)─H bonds offers a powerful strategy for converting abundant feedstocks and complex molecules into value‐added products, yet sustainable catalytic platforms that avoid precious‐metal photocatalysts remain underdeveloped. Here, we report a metallaphotoredox system that combines recyclable carbon nitride nanosheets with nickel catalysis for the direct *α*‐C(sp^3^)─H cross‐coupling of ethers, amides, and benzylic substrates under visible light. The method operates at mild temperature in air and enables both *α*‐C(sp^3^)─C(sp^2^) and *α*‐C(sp^3^)─C(sp^3^) bond formation using aryl and alkyl halides, including challenging ether donors such as THF, 2‐MeTHF, 1,4‐dioxane, and DME. Broad functional group tolerance is demonstrated across electronically diverse aryl halides, while late‐stage alkylation of pharmaceutically relevant molecules including loratadine, caffeine, and fenofibrate highlights the utility of the platform for molecular diversification. The carbon nitride photocatalyst is readily recovered and reused over five cycles without loss of activity, and the process is further translated to continuous flow, providing accelerated reaction kinetics and reduced photocatalyst loading. Mechanistic studies combining Stern–Volmer analysis, radical trapping, cyclic voltammetry, NMR spectroscopy, and DFT calculations support a dual catalytic cycle in which photoexcited carbon nitride promotes oxidative generation of *α*‐heteroatom‐stabilized radicals, while nickel mediates radical capture, cross‐coupling, and product‐forming reductive elimination. This work establishes carbon nitride as an earth‐abundant, recyclable alternative to noble‐metal photocatalysts for metallaphotoredox C─H functionalization and expands the scope of heterogeneous photocatalysis to direct *α*‐C(sp^3^)─H cross‐coupling.

## Graphical abstract

A recyclable carbon nitride/nickel metallaphotoredox platform enables direct *α*‐C(sp^3^)─H arylation and alkylation of ethers, amides, and benzylic substrates under visible light. Broad substrate scope, late‐stage functionalization, five‐cycle photocatalyst reuse, and continuous‐flow translation are demonstrated, while mechanistic studies support oxidative radical generation coupled with nickel catalysis, offering an earth‐abundant alternative to precious‐metal photoredox systems.

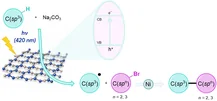

## 1 Introduction

The direct conversion of abundant, unfunctionalized hydrocarbon feedstocks into value‐added products represents a pivotal objective in modern synthetic organic chemistry. In this context, catalytic C─H bond functionalization offers a streamlined pathway to diversify drug‐like scaffolds without the need for preactivation [1]. While substantial progress has been made in the functionalization of C(sp^2^)─H bonds through directed *ortho*‐metalation or oxidative cross‐dehydrogenative couplings [2, 3], the selective activation of *α*‐C(sp^3^)─H bonds characterized by high bond dissociation energies and low polarity remains challenging. Traditional approaches based on deprotonation and nucleophilic substitution often require strongly basic organometallic reagents, such as organolithium or Mg‐based Grignard reagents [4], thereby limiting functional group tolerance. Consequently, the development of sustainable catalytic systems capable of overcoming the kinetic barriers associated with *α*‐C(sp^3^)─H bonds while maintaining high selectivity represents a key goal. This approach is not merely a matter of synthetic convenience but is driven by the growing demand for structurally complex, 3D molecules, which are associated with improved success in drug discovery [5].

In this context, a notable challenge is the functionalization of tetrahydrofuran (THF), a ubiquitous motif in bioactive compounds [6, 7]. THF lacks readily ionizable protons, precluding simple base‐mediated activation. Nevertheless, the *α*‐C(sp^3^)─H bonds of THF are relatively reactive compared with other aliphatic sites due to the adjacent oxygen atom; however, activating these bonds in a controlled, selective manner requires sophisticated catalytic paradigms involving radical or photochemical activation. Among the strategies available, Shirakawa and coworkers developed a notable method for the functionalization of THF using di‐*tert*‐butyl peroxide (DTBP) as a radical precursor (Figure 1a) [8]. In this system, the thermal homolysis of the peroxide occurs at 120 °C, generating *tert*‐butoxy radicals that promote hydrogen atom transfer, leading to a homolytic aromatic substitution. Similarly, Gong and colleagues introduced a nickel‐catalyzed thermal redox protocol (Figure 1a) [9]. This method merges the use of DTBP as an oxidant with zinc as a reductant to facilitate nickel‐mediated carbo‐functionalization of *N*‐ and *O*‐*α*‐C(sp^3^)─H bonds. Nevertheless, the requirement for high temperatures and the reliance on stoichiometric peroxides, which can be hazardous on a large scale, are significant drawbacks.

**FIGURE 1:**
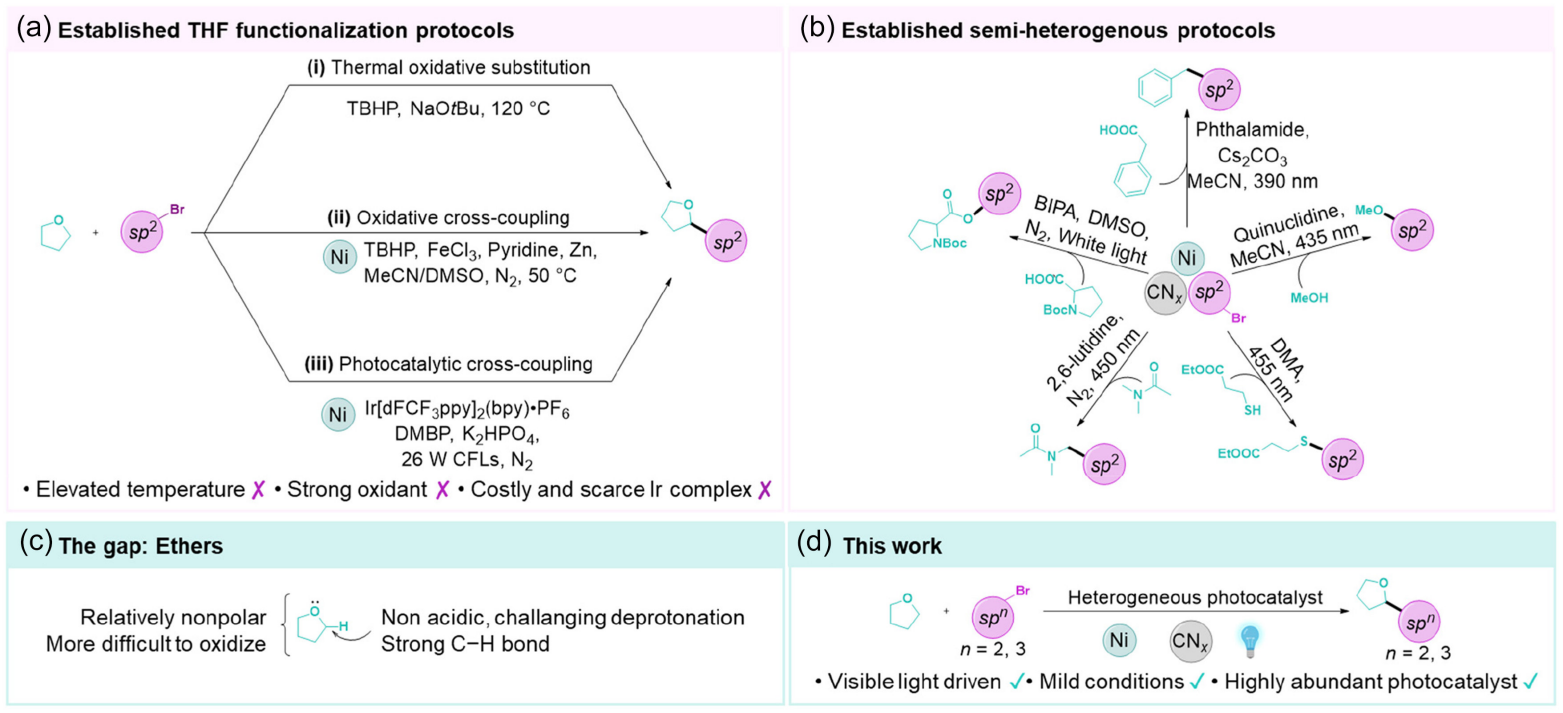
(a) Representative strategies for the direct *α*‐C(sp^3^)─H arylation of ethers, including thermal oxidative protocols, nickel‐catalyzed oxidative cross‐coupling, and homogeneous iridium‐based photoredox catalysis. (b) Representative semi‐heterogeneous carbon nitride/nickel metallaphotoredox systems, highlighting the broad range of activated substrates explored to date and the absence of ether substrates. (c) Key challenges associated with the direct functionalization of ethers arising from their strong, nonacidic *α*‐C(sp^3^)─H bonds and relatively high oxidation potentials. (d) Overview of the present work, which combines recyclable carbon nitride and nickel catalysis to enable visible‐light‐driven *α*‐C(sp^3^)─H alkylation and arylation of ethers under mild reaction conditions.

Alternatively, *α*‐C(sp^3^)─H functionalization can rely on a combination of visible‐light photoredox catalysis and a nickel transition‐metal catalyst. This dual catalysis platform leverages the ability of a photocatalyst to absorb light and generate reactive radicals under mild conditions (Figure 1a) [10, 11, 12]. The Doyle, Molander, and MacMillan groups established that dual iridium‐photoredox/nickel systems could effectively arylate ethers and amides, providing high levels of activity through oxidant‐free, open‐shell pathways that are not accessible through classical thermal activation. However, these approaches rely on noble‐metal catalysts, raising concerns regarding cost, recyclability, and long‐term sustainability at a large scale [13, 14]. To provide a comprehensive overview of these developments, Table S1 presents a comparison of representative methods for the C─H arylation of THF, highlighting key differences in catalytic systems and reaction conditions [8, 9, 10, 12, 15, 16].

A frontier in addressing the limitations of noble metal photocatalysts lies in the use of carbon‐based semiconductors [17], specifically carbon nitride (CN_*x*_). This material offers a unique combination of facile preparation, tunable electronic structures, exceptional chemical and thermal stability, and a heterogeneous nature that enables straightforward recovery and recycling [18, 19, 20, 21, 22]. Recent contributions have expanded the utility of CN_*x*_ from traditional applications in water splitting and CO_2_ reduction to more complex photocatalytic transformations (Figure 1b) [23, 24, 25, 26, 27, 28, 29, 30, 31, 32, 33, 34]. Photoexcited CN_*x*_ can mediate the Giese reaction of 1,3‐dioxolane with a wide array of SOMOphiles [35]. Similarly, CN_*x*_ has also been employed for the synthesis of arylated amides through a mechanism likely involving energy transfer (EnT) from the semiconductor to the nickel center [36]. However, the use of heterogeneous photocatalysts for the activation of unactivated ethers and benzylic *α*‐C(sp^3^)─H sites remains scarce (Figure 1c), [37, 38], particularly for the formation of *α*‐C(sp^3^)─C(sp^3^) bonds. Existing methods are largely limited to more readily activated substrates and predominantly favor *α*‐C(sp^3^)─C(sp^2^) coupling, leaving the direct *α*‐C(sp^3^)─C(sp^3^) cross‐coupling from unactivated C─H donors largely unexplored.

Herein, we report a dual catalytic system combining CN_*x*_ and nickel complexes for the direct *α*‐C(sp^3^)─H functionalization of a wide range of donors, specifically targeting benzylic positions, ethers, and amides in C(sp^3^)─C(sp^2^) and C(sp^3^)─C(sp^3^) cross‐coupling reactions (Figure 1d). The method operates under mild, visible‐light conditions at 40 °C in air, offering significant advantages in catalyst recyclability and operational simplicity. This shift from decarboxylative radical generation in our previous investigations to the in situ C─H activation reported here represents a distinct and significant mechanistic advance. Through a combination of experimental mechanistic studies supported by Stern–Volmer analysis, radical trapping experiments, cyclic voltammetry (CV), and NMR spectroscopy, alongside density functional theory (DFT) calculations, this work uncovers the elementary steps for radical formation and new insights into the dual catalytic cycle, revealing how the heterogeneous photocatalyst and the homogeneous nickel cocatalyst cooperate to drive selective bond formation.

## 2 Materials and Methods

Unless otherwise noted, the reagents and solvents were purchased from commercial suppliers and used without further purification.

### 2.1 Catalyst Synthesis

The catalyst was synthesized by calcination of melamine in an alumina crucible at 550 °C for 3 h using a heating rate of 10 °C min^−1^, yielding the graphitic carbon nitride (gCN_*x*_) intermediate. The resulting material was subsequently ground using a mortar and pestle and subjected to a second calcination step at 550 °C for 3 h, employing a heating rate of 2 °C min^−1^, to afford carbon nitride nanosheets (nCN_*x*_).

### 2.2 Catalyst Characterization

Attenuated total reflectance Fourier‐transform infrared (ATR‐FTIR) spectra were acquired using a Thermo Scientific Nicolet iS20 FTIR Spectrometer equipped with a Smart iTX accessory for ATR measurement and a deuterated triglycine sulfate (DTGS) detector. For each spectrum, 128 scans were recorded with a resolution of 4 cm^−1^ over the 4000–700 cm^−1^ range. Background spectra were also collected under the same conditions before each sample measurement. X‐ray diffraction (XRD) measurements were performed using a Bruker D2 Phaser X‐ray diffractometer equipped with a Cu K*α* radiation source (*λ* = 0.15405 nm). The powder samples were evenly spread on a PMMA sample holder. Diffractograms were collected over the 2*θ* range of 5°–80° with a step size of 0.016° and 0.4 s per step. Nitrogen physisorption measurements were carried out using a 3P Sync 400 instrument at 77 K. Powder samples were degassed at 150 °C under vacuum for 24 h to remove residual moisture and adsorbed species. The specific surface area was determined using the Brunauer–Emmett–Teller (BET) method, applied to the adsorption isotherms in the 0.05–‍0.3 *p*/*p*_0_ range. Pore size distribution was obtained using the Barrett–Joyner‐Halenda (BJH) method applied to the desorption branch of the isotherm. Elemental analysis of the C, H, and N content of the catalysts was determined by performing combustion analysis using a Vario MICRO Elemental Analyzer. Inductively coupled plasma optical emission spectrometry (ICP‐OES) was conducted using a Perkin Elmer Optima 8300 equipped with a photomultiplier tube detector. Scanning electron microscopy (SEM) images were acquired using a Zeiss EVO 50 EP microscope equipped with a Bruker Quantax 200 6/30. Energy‐dispersive X‐ray (EDX) samples were mounted on aluminum stubs with conductive carbon tape and subsequently gold‐coated using an Edwards S150B pure gold sputter coater, producing a layer of approximately 10 nm. Imaging was performed under high vacuum at an accelerating voltage of 20.0 kV with a working distance of 8.5 mm. Transmission electron microscopy (TEM) images were acquired using a Philips CM200 FEG equipped with a field emission gun (FEG). TEM samples were prepared by dropping the catalyst dispersion on a carbon‐coated copper grid and air‐dried at room temperature before imaging. The images were acquired at 200 kV. For Stern–Volmer experiments, the excitation source was an iBeam smart, single‐mode diode laser from Toptica with the emission centered at 375 nm. The laser was then focused on the sample; the emission was collected through a dichroic mirror (430 nm longpass) to filter the laser light. The emission is then focused on the fiber and collected by a Maya 2000pro spectrometer from Ocean Optics. The carbon nitride nCN_*x*_ in a solution with a concentration of 0.25 mg mL^−1^ was continuously stirred, and 10 μL of the 0.05 M stock quencher solution was added at each step.

### 2.3 Catalytic Transformations

Unless otherwise specified, the reactions were performed without inert gas or anhydrous solvent, using a commercial Solstice parallel 12‐Position LED Photoreactor (Uniqsis Ltd.), equipped with a Borealis LED light source (Uniqsis Ltd.) at 420 nm and 120 W. Thin‐layer chromatography (TLC) was performed on precoated silica gel 60 F254 plates (Merck) and visualized using UV light. Organic solutions were concentrated under reduced pressure on a Büchi rotary evaporator using a water bath. The crude products were analyzed via high‐performance liquid chromatography (HPLC). The conversion of the limiting reagent was deduced using an Agilent 1200 instrument, with an ultraviolet–visible detector (G1315D) set at 210 nm, injecting the samples and the standard (5 µL) directly onto a 250 mm × 4.6 mm Hypersil GOLD aQ 5 µm 175 Å column (Thermo Scientific). NMR measurements of the isolated products were carried out using a 400 MHz Bruker AVANCE II with a BBO probe at 298 K. Chemical shifts are given in ppm relative to the residual solvent peak (^1^H‐NMR *δ*: CDCl_3_ = 7.26; ^13^C‐NMR *δ*: CDCl_3_ = 77.16), and all coupling constants (*J*) are given in Hertz (Hz) with their multiplicity: (*s* = singlet, *d* = doublet, *t* = triplet, *q* = quartet, *p* = quintet, *m* = multiplet, *dd* = doublet of doublets, *dt* = doublet of triplets, *br* = broad). Cyclic voltammograms were collected with a BioLogic VSP 300 Potentiostat.

### 2.4 Computational Methods

The system reactivity was investigated using two different levels of DFT calculations to determine energies, structures, and Hessians of the relevant stationary points. nCN_*x*_ species were investigated using the rev‐DSDPBEP86 double‐hybrid functional [39, 40] and the jun‐cc‐pVTZ basis set [41], corrected for dispersion using the D3(BJ) model, which proved to be accurate to determine energies and structures of reacting systems [42]. The free‐energy barriers for H abstraction from the [THF⋯CO_3_]^•^^–^ complex were determined using the rev‐DSDPBEP86 functional and the def2‐TZVPPd basis set [43], as the jun‐cc‐pVTZ is not available for K. Simulations of systems involving the Ni‐based catalyst were performed using the PBE0 hybrid functional [44], corrected for dispersion using the D3(BJ) model (PBE0‐D3), as it was found to give results in good agreement with higher level CCSD(T) calculations [45], and the def2‐TZVP basis set [46]. For consistency, energies and structures of reactions involving both nCN_*x*_ and Ni‐based catalyst reactants were determined at the PBE0‐D3/def2‐TZVP level. A selected number of transition states, all characterized by having a single imaginary frequency, were determined for the main reaction paths. Preliminary searches of minimum energy structures and transition states were performed at the same level of theory using the 6‐311G(d,p) and SDD [47] smaller basis sets for${\text{nCN}}_{x}^{–}$ and Ni‐based systems, respectively. Solvation effects in THF were described using the SMD continuum model [48]. All simulations involving anions were performed by adding Na cations, so that a globally neutral system is simulated that better describes experimental conditions and minimizes shortcomings related to the use of an implicit solvation model. Entropies and Gibbs free energies were determined at 298 K and 1 bar in the rigid rotor harmonic oscillator (RRHO) approximation using structures and frequencies determined from Hessians computed at the chosen DFT level of theory. In the simulation of the (bpy)Ni compounds, *tert*‐butyl groups were replaced with methyl groups to speed up the simulations, with negligible loss of accuracy. All quantum chemical calculations were performed with Gaussian 16, using the protocols implemented in EStokTP (available at https://github.com/EStokTP) [49] to automate the calculations.

## 3 Results and Discussion

### 3.1 Catalyst Synthesis and Characterization

Melamine (4 g) was thermally condensed at 550 °C for 3 h using a heating rate of 10 °C min^−1^ in an alumina crucible, yielding 2.2 g of graphitic carbon nitride (gCN_*x*_). This intermediate was then ground with a pestle and mortar, followed by a second thermal exfoliation step at 550 °C for 3 h with a reduced heating rate of 2 °C min^−1^, affording 1.8 g of carbon nitride nanosheets (nCN_*x*_). The structural properties of the nCN_*x*_ catalysts were investigated using various characterization methods. The crystallographic structure of nCN_*x*_ was analyzed by XRD (Figure S1a). The XRD diffractogram revealed two major diffraction peaks at 2*θ* 12.8° and 27.3°, corresponding to the (100) and (002) planes, respectively. While the (100) reflection is attributed to the distance between adjacent melon units in the crystal structure, the intense (002) reflection corresponds to the spacing between graphitic layers, revealing that a layered structure is retained in nCN_*x*_. The XRD patterns are in good agreement with previously reported results [50, 51], indicating that the desired crystallographic structure was successfully obtained for nCN_*x*_. The chemical structure of the catalyst was further characterized by attenuated total reflectance Fourier transform infrared (ATR‐FTIR) spectroscopy (Figure S1b). The ATR‐FTIR spectrum consists of multiple absorption bands in the 1000–1700 cm^−1^ region, sharp peaks at 804 and 882 cm^−1^, and a broad band at 3163 cm^−1^. The bands in the 1000–1700 cm^−1^ region and the peak at 882 cm^−1^ are characteristic of stretching and bending modes of *N*‐containing heterocyclic compounds, respectively, and the peak at 804 cm^−1^ is attributed to the out‐of‐plane bending mode of triazine or heptazine units. Additionally, the broad band at 3163 cm^−1^ is attributed to N─H stretching vibrations of terminal nitrogen atoms [52, 53]. The CHN elemental analysis results (Table S2) indicate a C/N ratio of 0.60, which aligns with the reported values for nCN_*x*_ materials [54]. Additionally, the presence of a small quantity of hydrogen in the structure is in agreement with the ATR‐FTIR (Figure S1b) findings, suggesting the presence of residual amino functionalities typical of nCN_*x*_ materials. The specific surface area of nCN_*x*_ was determined by N_2_ physisorption (Figure S1c). BET analysis of the isotherms revealed a surface area of 28 m^2^ g^−1^ (Table S2). Finally, TEM images confirmed the irregular, sheet‐like morphology of nCN_*x*_ (Figure S2a,b).

### 3.2 Reaction Optimization and Control Experiments

Once the photocatalyst was synthesized and characterized, the optimization campaign began with identifying the most effective irradiation wavelength (Table S3). Purple light (420 nm) proved more effective than blue light (460 nm), and green light (520 nm) was found inactive. Next, the influence of dilution on the reaction efficiency was examined by varying the reaction concentration from 0.2 to 0.05 M. More dilute conditions favored the formation of **3aa**, likely due to improved light penetration and reduced aggregation or quenching effects (Table S4). These results are consistent with the typical observations in photocatalytic systems. To evaluate the role of THF, the reaction was performed in MeCN using THF as a stoichiometric reagent (1 equiv.). Under these conditions, the desired product was obtained in only a 3% yield. Increasing the amount of THF to 10 equiv., while maintaining all other reaction parameters, improved the yield to 17%. These results are consistent with the dual role of THF as both the hydrogen‐atom donor and the reaction medium, where its use as the solvent ensures a sufficiently high local concentration to compensate for its relatively high oxidation potential and to facilitate efficient C─H activation at the photocatalyst surface. Optimization of the photocatalyst loading identified 12.5 mg mL^−1^ of nCN_*x*_ as the concentration for subsequent studies (Table S5). Increasing the amount of carbon nitride beyond the optimized conditions resulted in a progressive decrease in yield, which is attributed to enhanced light scattering and reduced effective photon penetration in the heterogeneous suspension [55], together with increased nonproductive charge carrier recombination at the catalyst surface [56]. Screening of different nickel sources showed that NiCl_2_·glyme (10 mol%) gave the highest yield, making it the preferred Ni source (Table S6). The ligand was also found to play a crucial role in the reaction outcome. Among those tested, 4,4′‐di‐*tert*‐butyl‐2,2′‐bipyridine (dtbbpy) emerged as the optimal ligand. Its bulky structure enhances the catalyst stability and activity, and it also improves the solubility of the metal complex (Table S7). Base screening (Table 1) revealed only relatively modest differences in reactivity, with mild, nonnucleophilic inorganic bases performing best overall. Among inorganic carbonates, Na_2_CO_3_ proved to be the most effective base (43% yield), finding its optimal loading in 2 equivalents (Table S8), whereas K_2_CO_3_ (29%) and Cs_2_CO_3_ (12%) were less effective. Phosphate bases showed comparable but slightly reduced activity (K_2_HPO_4_, 35%; K_3_PO_4_, 26%). In contrast, strongly basic conditions were detrimental as KOH led to almost complete suppression of reactivity (4%). The reaction also proceeds in the presence of organic amine bases such as quinuclidine and DABCO, albeit with lower efficiency (25% and 22%, respectively). However, given their significantly lower oxidation potentials relative to carbonate‐derived species, these amines may undergo facile single‐electron oxidation by the excited photocatalyst to generate the corresponding amine radical cations [57, 58, 59]. These intermediates are capable of participating in hydrogen atom transfer (HAT), thereby leading to an alternative reaction pathway [57]. Consequently, mechanistic conclusions derived under inorganic base conditions cannot be directly extended to reactions employing quinuclidine or DABCO. Finally, monitoring the reaction over time revealed that it reached completion after 48 h (Figure S5). The reaction time is fully comparable to state‐of‐the‐art homogeneous photoredox systems based on homogeneous photocatalysts, which also require 24–‍72 h to reach full conversion in analogous C─H functionalization reactions [10, 15, 16, 58]. Control experiments confirmed the necessity of each reaction component (Table 1). Under the optimized conditions, the reaction delivered an 84% yield of **3aa** (Table 1, **entry 1**). In contrast, when 2,6‐lutidine was employed to reproduce the conditions reported [36], no formation of **3aa** was observed (Table 1, **entry 11**). Omitting the photocatalyst (nCN_*x*_) or the nickel catalyst resulted in no product formation (Table 1, **entries 12** and **13**), confirming their essential roles. Exclusion of the ligand led to a significant drop in yield to 8% (Table 1, **entry 14**). When the base was omitted, no reaction occurred (Table 1, **entry 15**). Furthermore, conducting the reaction in the dark yielded no product, highlighting the critical role of light activation in the photocatalytic process (Table 1, **entry 16**). Consistent with this proposal, a light on/off experiment revealed that product formation occurred exclusively during the illuminated periods and essentially ceased when the light source was switched off (Figure S6), demonstrating that continuous photoexcitation is required for catalysis. Under the optimized reaction conditions, an apparent quantum yield (AQY) of 0.09% was determined (Equation S1). The measured quantum yield (*Φ* < 1), determined using the incident‐photon method commonly employed for heterogeneous photocatalytic systems [59, 60, 61], is consistent with a nonchain photocatalytic mechanism in which sustained photoexcitation is required rather than a propagating radical chain process [62, 63].

**TABLE 1:** Optimized reaction conditions and control experiments.

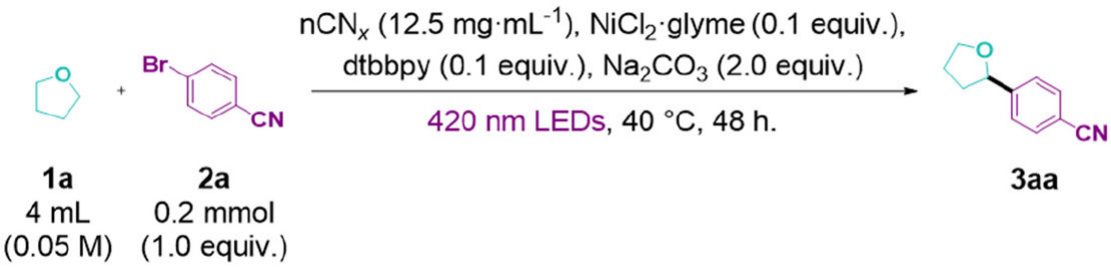		
Entry	Conditions	Yield of **3aa** a
1	As above	84%
2	For 24 h	43%^b^
3	With K_2_CO_3_	29%^b^
4	With Cs_2_CO_3_	12%^b^
5	With K_2_HPO_4_	35%^b^
6	With K_3_PO_4_	26%^b^
7	With NaF	n.d.^b^^,^ c
8	With KOH	4%^b^
9	With quinuclidine	25%^b^
10	With DABCO	22%^b^
11	With 2,6‐lutidine^d^	n.d.^c^^,^ d
12	No nCN_*x*_	n.d.^c^
13	No Ni source	n.d.^c^
14	No ligand	8%
15	No base	n.d.^c^
16	No light	n.d.^c^

a Yields of **3aa** were determined by HPLC using calibration standards.

b The reaction was conducted for 24 h.

c Product was not detected.

d The reaction was carried out using NiBr_2_·glyme (0.05 equiv.), bipyridine (0.05 equiv.), and 2,6‐lutidine (2.0 equiv.) in 1 mL of THF using 460 nm LEDs under N_2_ at 30 °C for 48 h.

The heterogeneous nature of the photocatalyst was further verified by a hot filtration experiment (Figure S7). After 24 h, the reaction afforded **3aa** in a 46% yield, after which the carbon nitride photocatalyst was removed by filtration. Subsequent irradiation of the filtrate under otherwise identical reaction conditions resulted in no further product formation, confirming that the photocatalytic activity originates from the heterogeneous carbon nitride material and excluding the presence of catalytically active species in solution.

To further evaluate the sustainability of the developed methodology, an E‐factor analysis was performed and benchmarked against representative homogeneous thermal [9] and photocatalytic [10] protocols reported for analogous C─H functionalization reactions (Table S9). Despite requiring prolonged irradiation and employing THF as both the reagent and solvent, the present protocol exhibits the most favorable process metrics among the methods evaluated, affording the lowest E‐factor (12.2), a 64% reduction in waste generation relative to the benchmark thermal process, and the highest isolated yield (88%). These findings highlight the sustainability of the methodology, which combines high material efficiency and reduced waste generation with the use of a recyclable heterogeneous carbon nitride photocatalyst, thereby avoiding nonrecoverable homogeneous photocatalysts employed in existing protocols.

### 3.3 Catalytic Experiments and Substrate Tolerance

With the optimized metallaphotoredox conditions established, the generality of the method was explored by evaluating a range of C─H donors and aryl halides (Figure 2). The model reaction with the corresponding THF **1a** and aryl bromide **2a** afforded product **3aa** in a 74% isolated yield. Additionally, the reaction was successfully scaled up by a factor of 12, from 0.2 to 2.4 mmol of **2a**, using a single round‐bottom flask illuminated by four Kessil lamps (427 nm). This yielded product **3aa** in a 71% isolated yield, demonstrating the feasibility of a 12‐fold scale‐up with comparable yields under our conditions. Beyond batch processing, the system was also adapted for continuous‐flow synthesis to see the practical translation of the heterogeneous photocatalytic platform to continuous operation (Figure S8). An FEP tube (80 cm in length, 2 mm inner diameter) was packed with a composite mixture of nCN_*x*_ and glass beads and connected to a peristaltic pump. The incorporation of glass beads as a copacking agent within the flow reactor bed promoted efficient dispersion and separation of catalyst particles, thereby minimizing photon absorption competition under visible‐light irradiation. Quinuclidine was used as a base in order to prevent clogging within the flow reactor; therefore, the mechanistic pathway operating under flow conditions cannot be assumed to be identical to that established for the Na_2_CO_3_‐based batch reaction. This setup led to a significant enhancement, up to 7.5‐fold compared to batch conditions, and enabled a three‐fold scale‐up of the reaction. Additionally, a lower loading of nCN_*x*_ was required (8 vs. 12.5 mg mL^−1^ in batch), and the system demonstrated excellent stability without detectable photocatalyst loss, paving the way for straightforward recyclability of the system. Next, using 2‐methyltetrahydrofuran (2‐MeTHF) as the C─H donor led to similar yields of both the anti‐Markovnikov (**3ba**, 35%) and Markovnikov (**3ba’**, 38%) products. This outcome reflects the balance between the enhanced stability of the tertiary carbon radical and its increased steric hindrance. Interestingly, the *trans* configuration of **3ba** was favored, likely due to steric interactions between the methyl group and the Ni complex during the radical‐trapping step. Acyclic ether diethyl ether was also arylated, giving **3ca** in a 31% yield. The lower reactivity relative to its cyclic analogs is attributed to greater steric flexibility and hindrance in the linear ether. Benzylic C─H donors such as toluene and mesitylene underwent efficient arylation, affording **3da** (69%) and **3ea** (75%), respectively. The slightly higher yield observed with mesitylene likely results from its lower oxidation potential and increased availability to form benzylic radicals. Next, amides proved to be excellent C─H donors. Arylation of *N*,*N*‐dimethylacetamide (DMA) and *N*,*N*‐dimethylformamide (DMF) furnished **3fa** and **3ga** in high yields (96% and 79%). Notably, *N*‐methyl pyrrolidone (NMP) underwent complete conversion, with a preference for the Markovnikov product (**3ha**, 66%) over the anti‐Markovnikov isomer (**3ha’**, 31%). This high reactivity is attributed to the low oxidation potentials and high polarity of these amides, which enhance both radical generation and overall solubility in the reaction medium. Finally, the more challenging arylation of 1,4‐dioxane yielded **3ia** in 24%, likely due to its high oxidation potential. In contrast, its linear analog 1,2‐dimethoxyethane (DME) was arylated more efficiently, delivering **3ja** in 63% yield.

**FIGURE 2:**
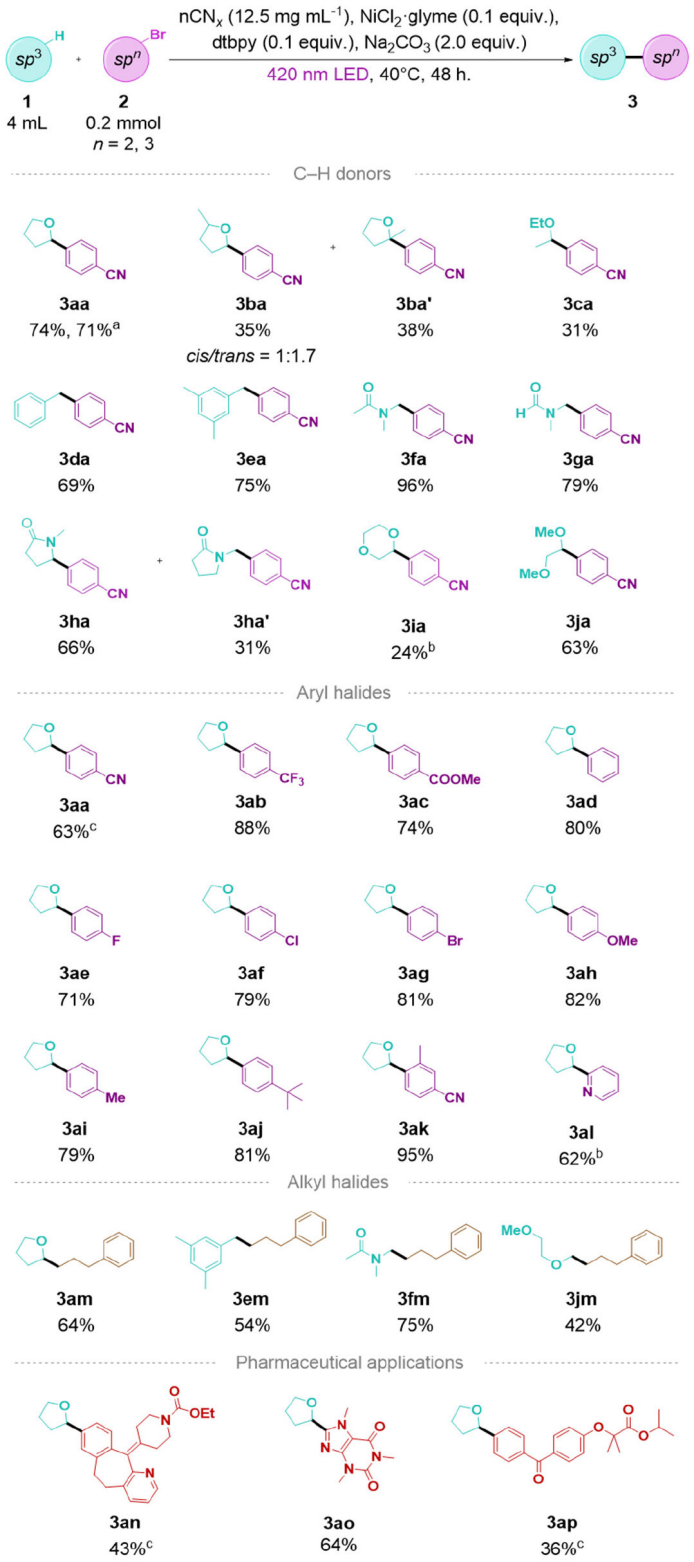
Substrate scope of the C(sp^3^)─H cross‐coupling reaction. Characterization data for all isolated products are provided in the Supporting Information. (a) Reaction performed on a 2.4 mmol scale. (b) Yield determined by ^1^H‐NMR spectroscopy. (c) Reaction performed using the corresponding aryl chloride.

Next, a variety of aryl halides were evaluated under the optimized conditions. The analogous aryl chloride was also competent, delivering **3aa** in a 63% yield. Electron‐deficient bromoarenes underwent efficient alkylation, affording **3ab** and **3ac** in 88% and 74% yields, respectively. Reactions with electron‐neutral bromoarenes, including unsubstituted bromobenzene and para‐substituted halogenated derivatives, proceeded similarly well, yielding **3ad**, **3ae**, **3af**, and **3ag** in 80%, 71%, 79%, and 81% yields, respectively. Encouragingly, electron‐rich bromoarenes also performed effectively, delivering **3ai** and **3aj** in 79% and 81% yields, respectively, indicating that electronic variations have minimal impact on reaction efficiency. This suggests that neither oxidative addition nor reductive elimination is the rate‐limiting step in the catalytic cycle. Further supporting this conclusion, introducing steric hindrance via an *ortho*‐methylated bromoarene had no detrimental effect on the outcome, with **3ak** obtained in an excellent 95% yield.

Notably, the optimized conditions were successfully extended to a C(sp^3^)─C(sp^3^) cross‐coupling, affording compound **3am** in a 64% yield. This result represents the first reported example of C─H donor alkylation in a cross‐coupling reaction using a heterogeneous photocatalyst. Additional C─H donors were successfully alkylated, delivering compounds **3em**, **3fm**, and **3jm** in 54%, 75%, and 42% yield, respectively. Notably, while the arylation of DME preferentially occurred at the internal methylene position to give **3ja**, the alkylation was selectively directed to the terminal position. To further demonstrate the versatility of the methodology, several pharmaceutical compounds, including loratadine, caffeine, and fenofibrate, were successfully alkylated, affording **3an**, **3ao**, and **3ap** in 43%, 64%, and 36% yield, respectively. The relatively lower yields observed for the functionalization of loratadine and fenofibrate can be attributed to the more challenging nature of chloroarene activation. These results highlight the potential of the nCN_*x*_ photocatalyst for late‐stage functionalization of bioactive molecules, providing a powerful tool for the rapid diversification of complex scaffolds without the need for de novo synthesis. This is particularly advantageous in medicinal chemistry, where the ability to modify C─H bonds in intermediates or drug candidates using the protocol developed herein can accelerate structure–activity relationship studies, reduce synthetic steps, and improve the overall efficiency during lead optimization. Beyond reactivity considerations, replacing iridium photocatalysts with carbon nitride also offers significant sustainability implications, considering that iridium is one of the scarcest elements in the Earth’s crust and is classified as a critical raw material, whereas carbon nitride is composed exclusively of earth‐abundant elements [64]. At typical catalyst loadings, Ir‐based photocatalysts contribute several euros per mmol of product, while the heterogeneous carbon nitride photocatalyst contributes a negligible cost [29]. Furthermore, the recoverability and reuse of carbon nitride substantially reduce catalyst waste compared to homogeneous iridium systems, which are generally employed in a single‐use fashion. Taken together, this approach reduces the precious metal intensity by orders of magnitude while maintaining the synthetic utility of metallaphotoredox C─H functionalization.

### 3.4 Recyclability and Post‐Catalysis Characterization

The nCN_*x*_ photocatalyst maintained its catalytic performance over five consecutive recycling experiments, demonstrating excellent operational stability and robustness. Specifically, the arylated product **3aa** was consistently obtained in high HPLC yields, ranging from 75% to 94% across these cycles (Figure 3a), indicating no detectable catalyst deactivation. Following each reaction, the nCN_*x*_ photocatalyst was quantitatively recovered by centrifugation, thoroughly washed with ethyl acetate and water, and dried overnight at 65 °C. Post‐catalysis characterization was carried out to evaluate the stability and robustness of nCN_*x*_ after the five catalytic cycles by comparing the structural features of the fresh and recycled nCN_*x*_. The XRD diffractograms (Figure 3b) and ATR‐FTIR spectra (Figure 3c) reveal that the crystallographic and chemical structure of the catalyst was preserved after catalysis, as both analyses showed matching features for the fresh and recycled nCN_*x*_. Elemental analysis indicated only minor changes in the bulk composition of the recycled material (Table S10), likely attributed to residual carbonaceous species that were weakly adsorbed during the reaction. Furthermore, ICP‐OES analysis (Table S10) detected only a small amount of physisorbed Ni species from the reaction medium onto nCN_*x*_, consistent with previous reports [27, 36]. This partial deposition had no measurable impact on catalytic performance as identical activity was maintained throughout the recycling study (Figure 3a), indicating no catalyst deactivation. In addition, the deposited nickel species exhibited no detectable metallaphotoredox activity as no product was formed if homogeneous Ni was not added. Importantly, these species could be readily removed by a simple post‐reaction acid wash (Figures S10–S12). The facile removal of nickel under these mild conditions suggests that the deposited species are only weakly adsorbed on the carbon nitride surface rather than being strongly coordinated or incorporated into the catalyst framework.

**FIGURE 3:**
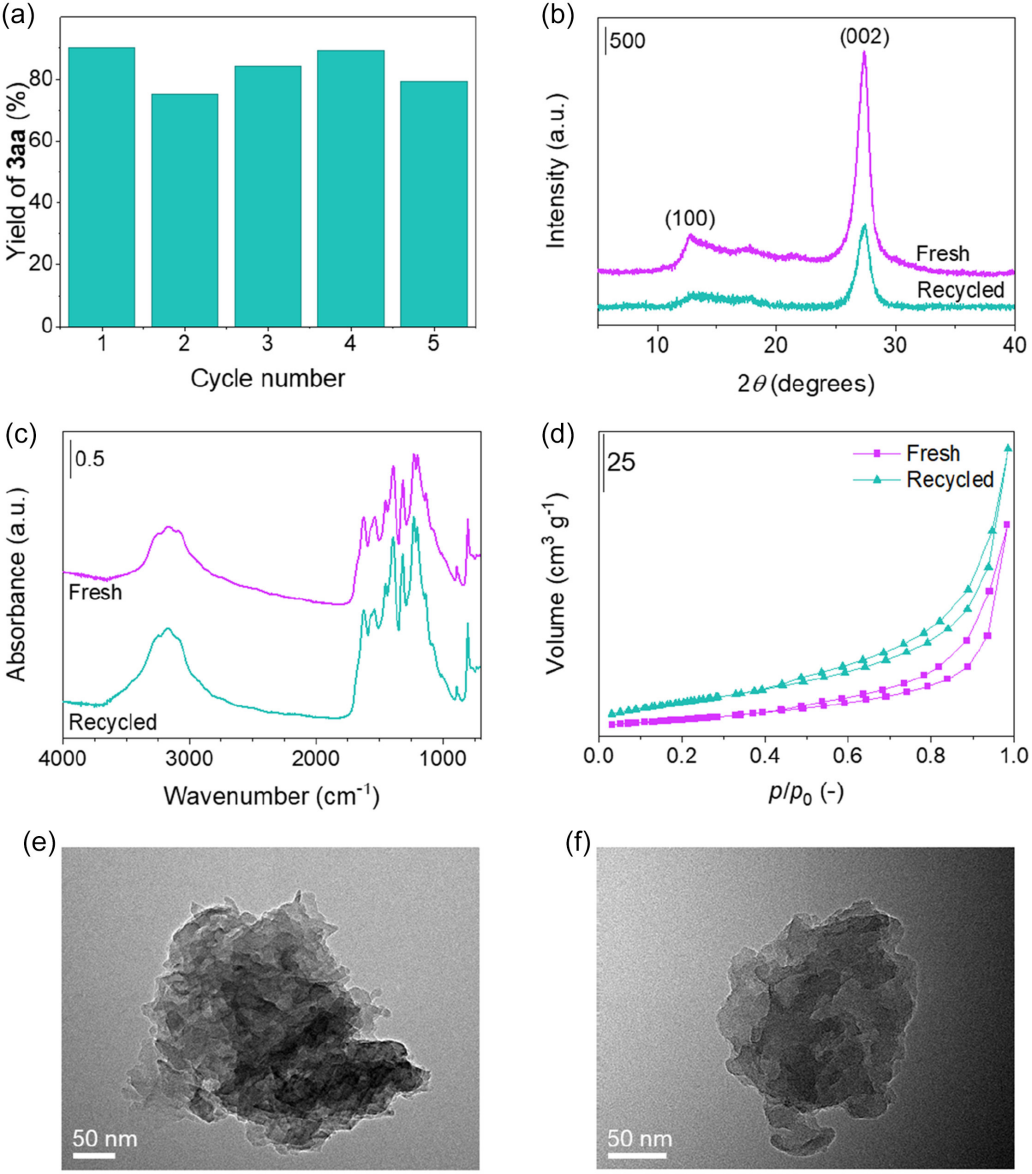
(a) Recyclability of nCN_*x*_ over five consecutive catalytic cycles. Product yields were determined by HPLC using a calibration curve of **3aa**. Structural characterization of fresh and recycled nCN_*x*_ after five catalytic cycles by (b) XRD, (c) ATR‐FTIR, and (d) N_2_ physisorption isotherm comparison, and TEM images of (e) fresh and (f) recycled nCN_*x*_.

N_2_ physisorption isotherms (Figure 3d) revealed an increase in the specific surface area of nCN_*x*_ from 28 to 58 m^2^ g^−1^ in the recycled nCN_*x*_, consistent with partial exfoliation and separation of the nanosheet layers during the reaction. In addition, the average pore diameter decreased from 21.06 to 13.98 nm. TEM images (Figure 3e,f) of the fresh catalyst show a more pronounced layered structure, whereas the recycled sample exhibits a less layered morphology, in agreement with exfoliation. SEM analysis (Figures S2, S9, and S11) shows no major structural variation as the carbon nitride framework remains structurally intact after reaction and washing, as confirmed by ATR‐FTIR and XRD. To gain further insight into whether the increase in surface area affects the photophysical properties of nCN_*x*_, UV‐Vis spectra of the fresh and recycled catalysts were acquired (Figure S13a,b). To compare the absorbance capacity of both materials, the area under the absorption curve between 300 and 800 nm was calculated, obtaining values of 21 AU nm for the fresh catalyst and 45 AU nm for the recycled catalyst. These values were subsequently normalized to the corresponding BET surface areas, resulting in 0.75 AU nm g m^−2^ for the fresh catalyst and 0.77 AU nm g m^−2^ for the recycled catalyst. Additionally, the TOF values obtained for the fresh and recycled catalysts were also normalized by their respective BET‐normalized absorbance values, leading to 9.2 and 8 × 10^−3^ h m^2^ AU^−1^ nm^−1^ g^−1^ for the fresh and recycled catalyst (Figure S13c). These estimated values indicate that, despite the increase in the overall surface area, the light absorption capacity per unit of the surface area remains essentially unchanged. Overall, the combined characterization results indicate that repeated catalysis induces only surface and morphological changes without altering the chemical structure or compromising the photocatalytic activity of nCN_*x*_.

### 3.5 Mechanistic Investigations

With its broad substrate scope and robust catalytic performance established, detailed mechanistic studies were undertaken using a combination of experimental and computational approaches. The reaction mechanism, named M1, proposed to interpret the present experiments and the corresponding free‐energy surface diagrams is sketched in Figure 4a–c. To maintain computational feasibility, the nCN_*x*_ photocatalyst was modeled using the 2,5,8‐triamino‐heptazine monomer, which represents the fundamental structural and electronic building block of graphitic carbon nitride. Although this finite‐molecular model does not explicitly account for long‐range electronic and structural effects associated with the extended polymeric nanosheet, it provides a reliable approximation for evaluating relative reaction energetics and identifying plausible mechanistic pathways. Previous computational studies have extensively employed triazine‐ and heptazine‐based molecular models to investigate the electronic structure, charge distribution, adsorption behavior, and catalytic activity of carbon nitride materials as these models preserve the local coordination environment and frontier electronic characteristics governing photocatalytic processes. Moreover, finite molecular models typically contain only a few tens of atoms compared with several hundred atoms required for periodic carbon nitride supercells. Consequently, such calculations are generally one to two orders of magnitude less computationally expensive while retaining the essential chemical features required for mechanistic investigations. The mechanism consists of two interconnected catalytic cycles, one where nCN_*x*_ is photoexcited and initiates the photocatalytic cycle through the formation of a reactive radical and the other where the Ni and ligand complex ((bpy)Ni) catalyzes the cross‐coupling transformation. The elementary steps are

$${\mathrm{nCN}}_{x\;}{\text{→}}^{\text{3}}\;{\mathrm{nCN}}_{x}\;\left(R1\right)$$

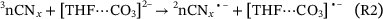

$${\left[\mathrm{THF}\cdots {\mathrm{CO}}_{3}\right]}^{•–}\to {{\mathrm{HCO}}_{3}}^{–}+{\mathrm{THF}}^{•}\;\left(R3\right)$$

$$(\mathrm{bpy}){\mathrm{Ni}}^{0}\text{ + }\mathrm{Br}\text{‐}\mathrm{PhCN}\;\to \;\left(\mathrm{bpy}\right){\mathrm{Ni}}^{\mathrm{II}}\left(\mathrm{PhCN}\right)\mathrm{Br}\;\left(R4\right)$$

$$\begin{array}{c} \begin{array}{c} \left(\mathrm{bpy}\right){\mathrm{Ni}}^{\mathrm{II}}\left(\mathrm{PhCN}\right)\mathrm{Br}+{\mathrm{THF}}^{•}\;\to \left(\mathrm{bpy}\right){\mathrm{Ni}}^{\mathrm{III}}\mathrm{THF}\left(\mathrm{PhCN}\right){\mathrm{Br}}^{•}\;\left(R5\right) \end{array} \end{array}$$

$$\begin{array}{c} \left(\mathrm{bpy}\right){\mathrm{Ni}}^{\mathrm{III}}\mathrm{THF}\left(\mathrm{PhCN}\right){\mathrm{Br}}^{•}\to \left(\mathrm{bpy}\right){\mathrm{Ni}}^{I}{\mathrm{Br}}^{•}+\mathrm{THF}\text{‐}\mathrm{PhCN}\;\left(R6\right) \end{array}$$

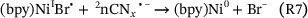




**FIGURE 4:**
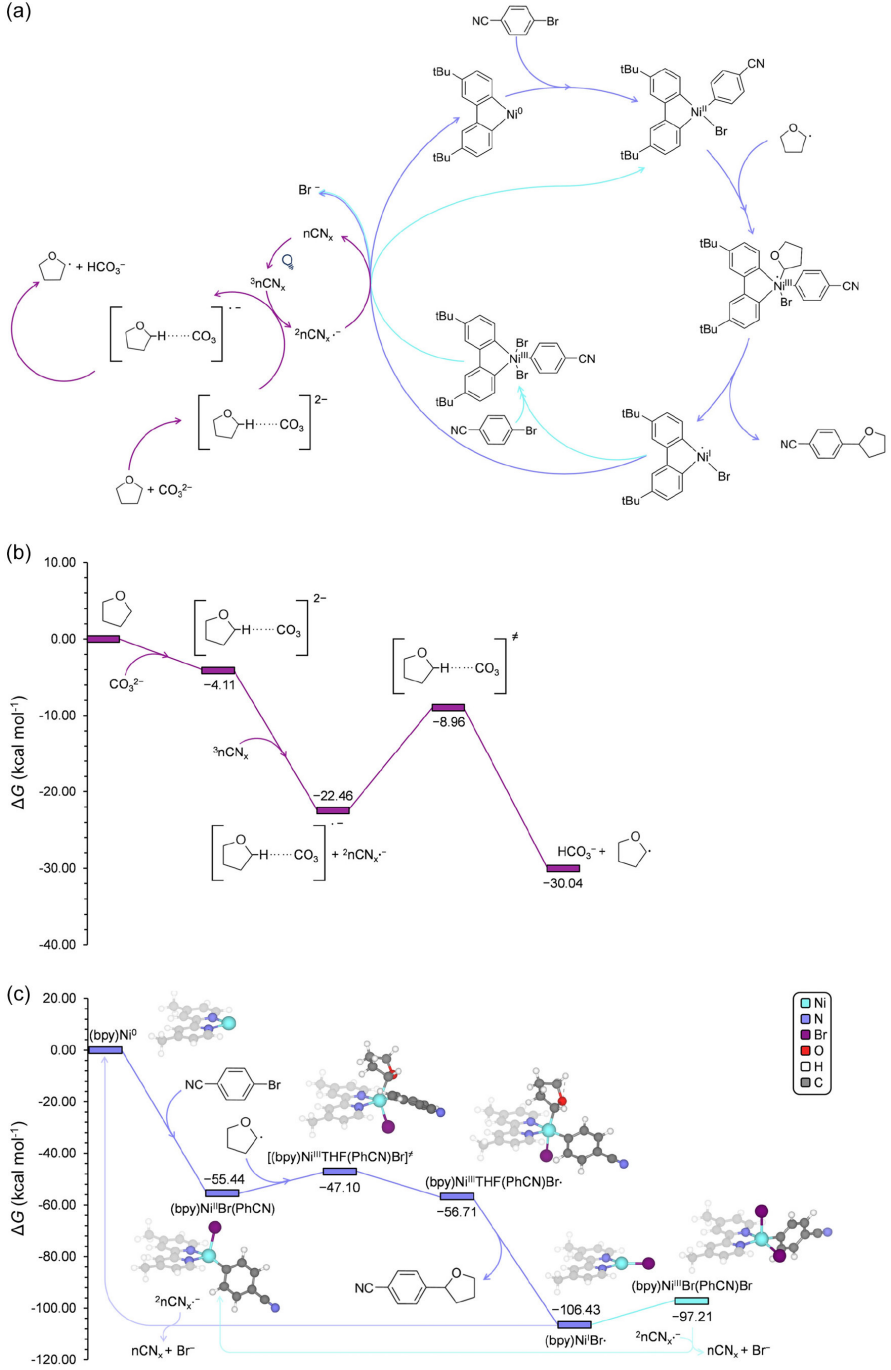
(a) Proposed dual carbon nitride/nickel metallaphotoredox mechanism for *α*‐C(sp^3^)─H arylation of THF. (b) Computed Gibbs free‐energy profile for the carbon nitride‐mediated radical‐generation pathway, including THF/carbonate association, photoinduced electron transfer, and formation of the *α*‐oxy THF radical. (c) Computed Gibbs free‐energy profile for the nickel catalytic cycle, including oxidative addition of the aryl bromide, radical capture, product‐forming reductive elimination, and catalyst regeneration. Energies are reported in kcal mol^–1^.

The activation of the metal‐free carbon nitride support (nCN_*x*_) from the ground singlet state to the triplet excited state (^3^nCN_*x*_) takes place through reaction R1 and requires 92.1 kcal mol^–1^. This step is expected to take place through the photoexcitation of carbon nitride, which provides the energy necessary to sustain the system reactivity, and its relevance as an initiation process is consistent with the experimental evidence that without photoexcitation the reaction does not proceed (Table 1, **entry 16**). The formation of a triplet excited state as the initiation step of the reactive process is consistent with the experimental evidence of the role of triplet states in the photoexcitation of carbon nitride motifs. Moreover, triplet states are expected to contribute more to the system reactivity than excited singlet states because of the longer lifetime. Following R1, the successive R2 step is the single electron transfer (SET) between ^3^nCN_*x*_ and THF in a basic environment, leading to the formation of the reactive radical intermediate, [THF⋯CO_3_]^•^^–^ and the successive H‐abstraction from THF by the carbonate in R3 to form THF^•^. The theoretical analysis shows that these reactive pathways are both thermodynamically favored, with mildly negative free‐energy changes. While R2 is expected to be a barrierless process, R3 has a considerable free‐energy barrier, mostly determined by the interaction between the base and THF. It was hypothesized that the presence of Na_2_CO_3_ modifies the local acid–base and hydrogen‐bonding environment in solution, thereby influencing the electronic environment of THF and facilitating subsequent H‐abstraction events. In fact, as shown by in situ ^1^H‐NMR spectroscopy (Figure 5a), upon contacting THF in D_2_O with increasing equivalents (from 0 to 2.0) of Na_2_CO_3_, both the coupling constants and the chemical shifts of the THF protons are affected, and, as the concentration of the base increases, the signals move upfield. These observations are attributed to changes in the bulk basic medium and dynamic intermolecular interactions, which increase the electron density around the THF protons.

**FIGURE 5:**
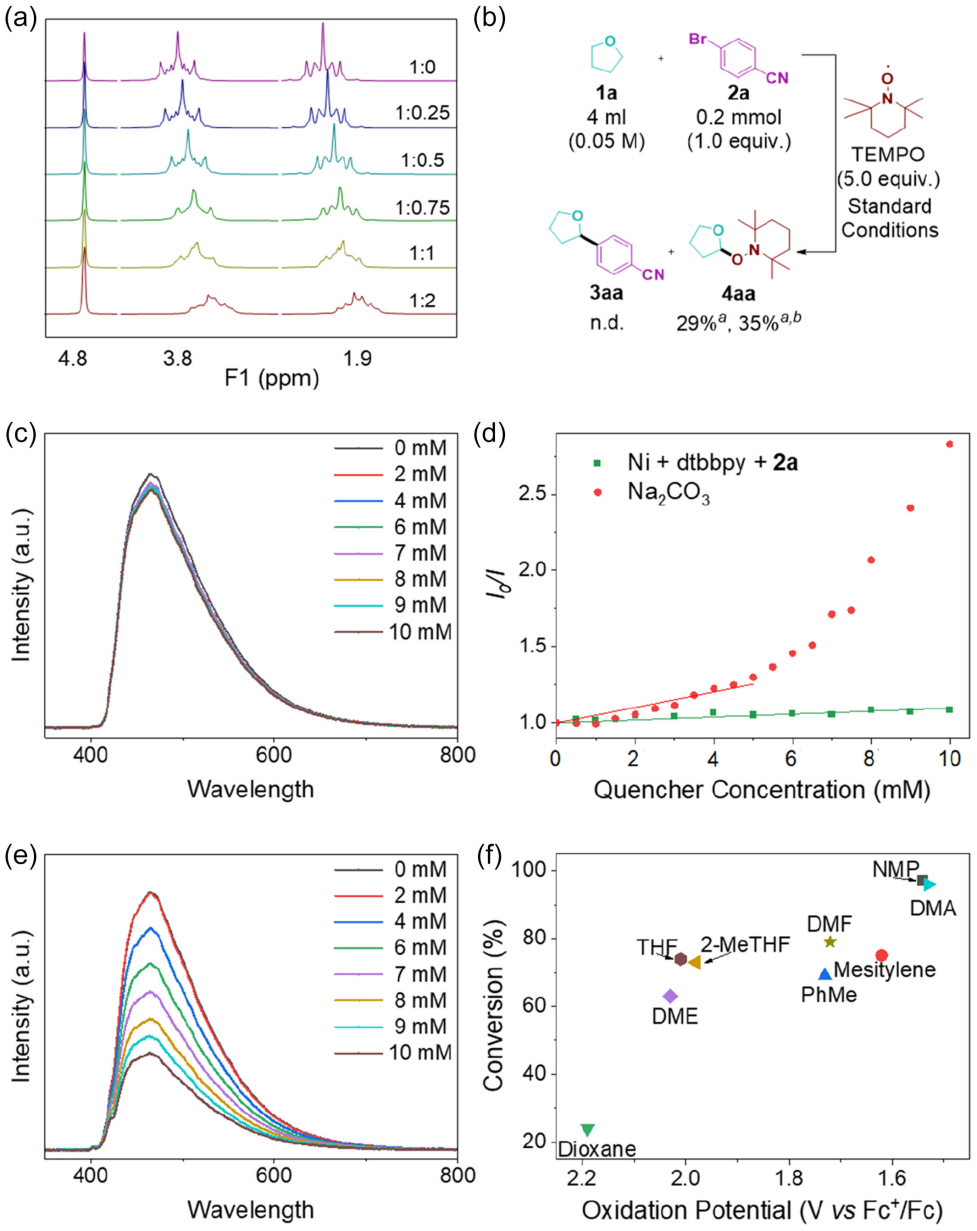
(a) In situ ^1^H‐NMR spectra of THF in D_2_O recorded with increasing Na_2_CO_3_ loading. (b) Radical‐trapping experiment with TEMPO under the standard reaction conditions. (c) Photoluminescence spectra of nCN_*x*_ upon addition of a mixture of NiCl_2_·glyme, dtbbpy, and **2a** in THF. (d) Stern–Volmer analysis comparing quenching by the Ni/dtbbpy/**2a** mixture and Na_2_CO_3_. (e) Photoluminescence spectra of nCN_*x*_ upon addition of Na_2_CO_3_ in THF. (f) Correlation between the oxidation potentials of the C─H donors and reaction conversion. The yields were determined by ^1^H‐NMR using TMB as an internal standard. The experiment in (b) was performed with THF (4 mL), nCN_*x*_ (50 mg), and Na_2_CO_3_ (0.4 mmol) under 420 nm irradiation.

Once formed, THF^•^ adds in R5 to the (bpy)Ni^II^(PhCN)Br complex, formed through the oxidative addition of Br‐PhCN in R4 to the (bpy)Ni^0^ complex, to form (bpy)Ni^III^THF(PhCN)Br^•^. Although reaction R5 is exothermic by about 15 kcal mol^–1^, the free‐energy change is only mildly negative, mainly because of the entropy loss caused by the addition reaction. The generation of the reaction product takes place through R6, passing through a transition state having a trigonal bipyramidal‐like shape and a free‐energy barrier of about 9 kcal mol^–1^. Catalyst regeneration occurs in R7 through electron transfer from ^2^nCN_*x*_^•^^–^ (byproduct in R2), releasing bromide and forming nCN_*x*_ and the (bpy)Ni^0^ complex, thus closing the reactive cycle [65]. All reaction steps are characterized by negative free‐energy changes, with the exception of reaction R7, which leads to the generation of the highly reactive (bpy)Ni^0^ complex with a positive free‐energy change of about 35 kcal mol^–1^, thus suggesting that this reaction step should take place simultaneously with reaction R4, which is thermodynamically favored. However, once the (bpy)Ni^I^Br^•^ intermediate is formed, the regeneration of the (bpy)Ni^0^ complex can be avoided if the (bpy)Ni^I^Br^•^ intermediate reacts with Br‐PhCN to form the (bpy)Ni^III^Br(PhCN)Br complex, which can then react with ^2^nCN_*x*_^•^^–^ to regenerate nCN_*x*_ and form (bpy)Ni^II^(PhCN)Br through reactions R8 and R9

$$\begin{array}{c} \left(\mathrm{bpy}\right){\mathrm{Ni}}^{I}{\mathrm{Br}}^{•}\text{+}\mathrm{Br}‐\mathrm{PhCN}\text{ → }\left(\mathrm{bpy}\right){\mathrm{Ni}}^{\mathrm{III}}\mathrm{Br}\left(\mathrm{PhCN}\right)\mathrm{Br}\;\left(R8\right) \end{array}$$

$$\begin{array}{c} \left(\mathrm{bpy}\right){\mathrm{Ni}}^{\mathrm{III}}\mathrm{Br}\left(\mathrm{PhCN}\right)\mathrm{Br}{\text{+}}^{\text{2}}{{\mathrm{nCN}}_{x}}^{\text{•–}}\to \left(\mathrm{bpy}\right){\mathrm{Ni}}^{\mathrm{II}}\left(\mathrm{PhCN}\right)\mathrm{Br}\text{+}{\mathrm{Br}}^{–}\;\left(R9\right) \end{array}$$

Reactions R8 and R9 represent a thermodynamically favorable alternative to reactions R4–R6, with free‐energy changes of +9.2 and −58.0 kcal mol^–1^, respectively. It can then be reasonably argued that the reaction mechanism is started by a reaction cycle given by R1–R7 (Figure 4a,c) and can continue once the (bpy)Ni^I^Br^•^ intermediate is formed with reaction R8 and R9 replacing R4 and R7.

While the mechanism proposed herein provides a consistent rationalization of the experimental insights, it is instructive to place the observed reactivity within the broader context of established literature precedents. In particular, two EnT mechanisms [66], specifically M2 (Figure S24), proposed by Molander et al. [10], and M3 (Figure S25), proposed by Doyle et al. [11], were also considered. Both mechanisms are described and discussed in detail in the Supporting Information. Among the two pathways, mechanism M3 appears most consistent with the photocatalytic scenario considered in this work as it can be sustained by the excitation energy of nCN_*x*_ from the ground singlet state to the triplet state. However, M3 presents several intrinsic limitations that are expected to slow down the overall reactivity. First, the formation of the Br^•^ radical from the (bpy)Ni^II^Br_2_ requires the formation of an excited triplet or singlet state to proceed as the triplet and singlet states are stable with respect to Br^•^ loss. However, it is likely that the main fate of the excited complex would be internal conversion to the ground state, which would rapidly dissipate the internal energy through thermal relaxation. Second, the (bpy)Ni^II^Br_2_ complex is thermodynamically stable relative to other (bpy)Ni^I^Br^•^ complexes, which is the reason why its formation through the decomposition of (bpy)Ni^II^Br_2_ and Br^•^ is favored. This, however, also means that its successive reactivity is hindered by its thermodynamic stability. Notably, the formation of the (bpy)Ni^III^Br(PhCN)Br complex is energetically unfavored. This is likely to have a dramatic impact on the system reactivity since, once formed, (bpy)Ni^III^Br(PhCN)Br must react with (bpy)Ni^I^Br^•^ to further progress the reactive cycle. Since this reaction step is likely to be slow, it follows that (bpy)Ni^I^Br^•^ will accumulate in the system, thus favoring the recombination reaction with Br^•^. Instead, the mechanism proposed herein involves the reactivity only of ground states and the free‐energy changes of all reaction steps are negative, which means that the forward reactions are thermodynamically favored.

Several experimental measurements support the mechanism proposed. Evidence for the involvement of radical intermediates was obtained via a radical‐trapping experiment using TEMPO (Figure 5b). Under optimized reaction conditions, the addition of TEMPO completely suppressed the formation of product **3aa**, leading to the corresponding TEMPO adduct **4aa** in 29% yield by ^1^H‐NMR [67, 68], suggesting that TEMPO acts predominantly as an electronic quencher of the oxidized intermediate. A control experiment performed in the presence of only THF, nCN_*x*_, Na_2_CO_3_, and TEMPO afforded the **4aa** adduct in a 35% ^1^H‐NMR yield, providing direct evidence for the generation of an *α*‐oxy radical intermediate and further supporting the proposed reaction mechanism. Next, the interaction between different substrates and nCN_*x*_ was investigated using photoluminescence (PL) quenching and Stern–Volmer analysis. Stern–Volmer analysis is used to evaluate the efficiency of charge transfer from nCN_*x*_ to a quencher, where quenching of the excited state leads to a decrease in emission intensity. For the analysis, we integrated the PL emission and calculated the ratio *I*_0_ */I*, where *I*_0_ is the emission intensity in the absence of the quencher and *I* is the intensity upon addition of different substrates. The addition of a mixture of NiCl_2_·glyme (0.1 equiv.), dtbbpy (0.1 equiv.) and **2a** (1.0 equiv.) in THF does not significantly quench the emission (Figure 5c), yielding an essentially flat Stern–Volmer plot (Figure 5d), indicating weak or negligible interaction with nCN_*x*_. In contrast, addition of Na_2_CO_3_ in THF with nCN_*x*_ leads to a strong decrease in emission intensity, indicating efficient interaction with the carbon nitride and effective quenching (Figure 5e). At low quencher concentrations, a linear Stern–Volmer regime is observed. However, at higher concentrations, an upward deviation (increase in *I*_0_ */I*) is observed (Figure 5d), suggesting a combination of dynamic and static quenching. This behavior indicates that part of the quencher may already be associated with the nCN_*x*_ surface prior to excitation, allowing quenching to occur by preventing the photoinduced encounter.

Finally, if the reaction indeed proceeds via the M1 pathway, then the oxidation potential of the C─H donor should be a key determinant of reactivity, in addition to factors such as solvent polarity and substrate solubility. To test this hypothesis, cyclic voltammetry (CV) measurements were performed (Table S11 and Figures S14–S23), and the observed oxidation potentials were correlated with reaction efficiency (Figure 5f). A clear trend emerged: substrates with higher oxidation potentials, such as 1,4‐dioxane (2.19 V vs. Fc^+^/Fc), afforded low conversion (24%), indicating poor reactivity. In contrast, substrates with moderately lower oxidation potentials such as DME (2.03 V vs. Fc^+^/‍Fc), THF (2.01 V vs. Fc^+^/Fc), and 2‐MeTHF (1.98 V vs. Fc^+^/Fc) showed significantly improved conversions of 63%, 74%, and 73%, respectively. Notably, substrates with substantially lower oxidation potentials, such as DMA (1.53 V vs. Fc^+^/Fc) and NMP (1.54 V vs. Fc^+^/Fc), achieved nearly quantitative conversion (96% and 97%, respectively). These observations are fully consistent with mechanism M1, in which the ease of substrate oxidation directly impacts the reaction outcome. Finally, the M1 mechanism allows us to explain the experimental evidence that the system reactivity decreases significantly with a change of the counterion (Table 1) going from Na_2_CO_3_ (43% conversion) to K_2_CO_3_ (29% conversion) and Cs_2_CO_3_ (12% conversion). Indeed, the theoretical simulations showed that the counterion plays an important role in modulating the hydrogen‐bonding/proton‐transfer environment at the interface, which is reflected in the observed changes in the in situ NMR spectra since it interacts both with the oxygen atoms of THF and those of CO_3_^2–^. This interaction, necessary for the subsequent deprotonation, is partially lost at the transition state, whose lowest‐energy conformer (Figure S26) has a seven‐membered ring structure. Simulations performed with the Na^+^ and K^+^ counterions reveal that the reaction free‐energy barrier increases from 13.5 to 14.6 kcal mol^–1^ going from Na^+^ to K^+^, respectively, thus providing a theoretical explanation for the observed decrease in conversion.

## 4 Conclusion

This study establishes a semi‐heterogeneous metallaphotoredox platform for visible‐light‐driven *α*‐C(sp^3^)─H functionalization using a dual nickel/carbon nitride catalytic system. The protocol demonstrates broad utility, enabling efficient C(sp^3^)─C(sp^2^) and C(sp^3^)─C(sp^3^) cross‐coupling reactions with excellent functional group compatibility, wide substrate applicability, and strong performance in modifying pharmaceutically relevant compounds such as loratadine, caffeine, and fenofibrate. Additionally, recyclability tests confirmed the robustness of nCN_*x*_ over five consecutive catalytic cycles while retaining its structural integrity. Mechanistic studies supported by radical trapping experiments, cyclic voltammetry, NMR, and DFT analysis revealed a dual catalytic cycle involving oxidative single‐electron transfer initiated by carbon nitride and nickel–catalyzed bond formation. The work highlights the promise of earth‐abundant materials as practical alternatives to precious‐metal systems, offering a straightforward and robust platform for the synthesis of complex molecules.

## Author Contributions

**Miguel M. de Vries Ibáñez** prepared and characterized the catalyst, optimized the reaction conditions, conducted recyclability studies, performed the mechanistic investigations, evaluated the substrate scope, and carried out the flow catalytic reactions. **Nathan G. G. Husy** contributed to catalyst preparation and reaction optimization. **Elisabetta Inico** and **Carlo Cavallotti** performed DFT calculations. **Kaan Karaca** carried out the catalyst characterization. **Milla Vigliengo** performed cyclic voltammetry experiments, while **Andrea Olivati** and **Annamaria Petrozza** conducted the Stern–Volmer analysis. **Gianvito Vilé** conceived and supervised the project. All authors contributed to writing the manuscript and approved the final version.

## Funding

This study was supported by HORIZON EUROPE Marie Sklodowska‐Curie Actions (101073089), HORIZON EUROPE European Research Council (101075832), and HORIZON EUROPE Global Challenges and European Industrial Competitiveness (101057430).

## Conflicts of Interest

The authors declare no conflicts of interest.

## Supporting information

Additional characterization and catalytic results of the materials, additional DFT calculations, continuous flow procedure, and NMR characterizations of the reaction products.

## Data Availability

The data that support the findings of this study are available from the corresponding author upon reasonable request.
