# Supplementary material for: Direct α‐C(sp3)─H Alkylation and Arylation of Ethers Enabled by Carbon Nitride/Nickel Metallaphotoredox Catalysis
Source: ChemSusChem. 2026 Oct 23;19(20):e71111. doi: 10.1002/cssc.71111 (PMC13693888; doi:10.1002/cssc.71111)
Supplement: Supplementary file 1 [file CSSC-19-e71111-s001.pdf]

Supporting Information

Direct α-C(sp^3)–H Alkylation and Arylation of Ethers Enabled by Carbon Nitride/Nickel Metallaphotoredox Catalysis

Miguel M. de Vries Ibáñez,[a] Nathan G. G. Husy,[a] Elisabetta Inico,[a] Kaan Karaca,[a] Milla Vigliengo,[a] Andrea Olivati,[b] Annamaria Petrozza,[b] Carlo Cavallotti,[a] and Gianvito Vilé,[a]*

[a] *Department of Chemistry, Materials, and Chemical Engineering "Giulio Natta", Politecnico di Milano, Piazza Leonardo da Vinci 32, 20133 Milano, Italy.*

[b] *Center for Nano Science and Technology, Istituto Italiano di Tecnologia, via Rubattino 81, 20133 Milano, Italy.*

* *Corresponding author. E-mail: gianvito.vile@polimi.it (GV)*

Literature analysis

Table S1. *Protocols reported in the literature for the α-arylation of THF.*

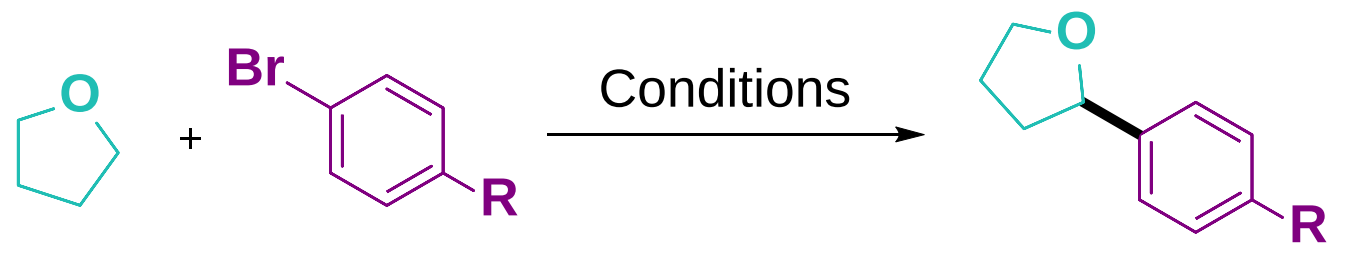

Entry	Reaction class	Conditions	–R	t (h)	T (°C)	Product yield	Reference
1	Thermal oxidative	THF (80 equiv.), *t*BuOOtBu (1.0 equiv.), N_2	–CN	24	120	26%	[1]
2	Thermal oxidative homogeneous catalysis	$NiBr_2$ (0.06 equiv.), dtbbpy (0.06 equiv.), $FeCl_3$ (0.05 equiv.), pyridine (1.5 equiv.), CH_3CN/DMSO 1:1 (0.75 M), Zn (2.0 equiv.), DTBP (4.0 equiv.), N_2	$–CF_3$	12	50	37%	[2]
3	Homogeneous Ir based photocatalysis	3-acetoxyquinuclidine (1.1 equiv.), Ir photocatalyst (0.01 equiv.), $NiBr_2$ (0.01 equiv.), 4,7-dOMe-phen (0.01 equiv.), H_2O (40 equiv.), DMSO (0.25 M), blue light, N_2	–COOMe	24	Not specified	76%	[3]
4	Homogeneous Ir based photocatalysis	THF (0.05 M), Ir photocatalyst (0.02 equiv.), $Ni(NO_3)_2{\cdot}6H_2O$ (0.05 equiv.), dtbbpy (0.05 equiv.), K_2HPO_4 (2.0 equiv.), blue light, N_2	$–CF_3$	72	25	63%	[4]
5	Homogeneous ketone based photocatalysis	$Ni(acac)_2$ (0.1 equiv.), dmbpy (0.01 equiv.) Na_2CO_3 (1.0 equiv.), THF (0.075 M), FMBP (0.1 equiv.), blue light, N_2	$–CF_3$	60	Not specified	95%	[5]
6	Homogeneous ketone based photocatalysis	THF (0.2 M), PhCHO (0.5 equiv.), $NiBr_2{\cdot}$glyme (0.1 equiv.) dtbbpy (0.1 equiv.), K_2HPO_4 (2.0 equiv.), UV light, N_2	$–CF_3$	72	Not specified	98%	[6]
7	Semi-heterogeneous carbon nitride based photocatalysis	THF (0.05 M), $NiCl_2$ glyme (0.1 equiv.), dtbbpy (0.1 equiv.), Na_2CO_3 (2.0 equiv.), nCN_x (12.5 mg mL^{-1}), purple light	$–CF_3$	48	40	88%	This work

Carbon nitride nanosheets characterization

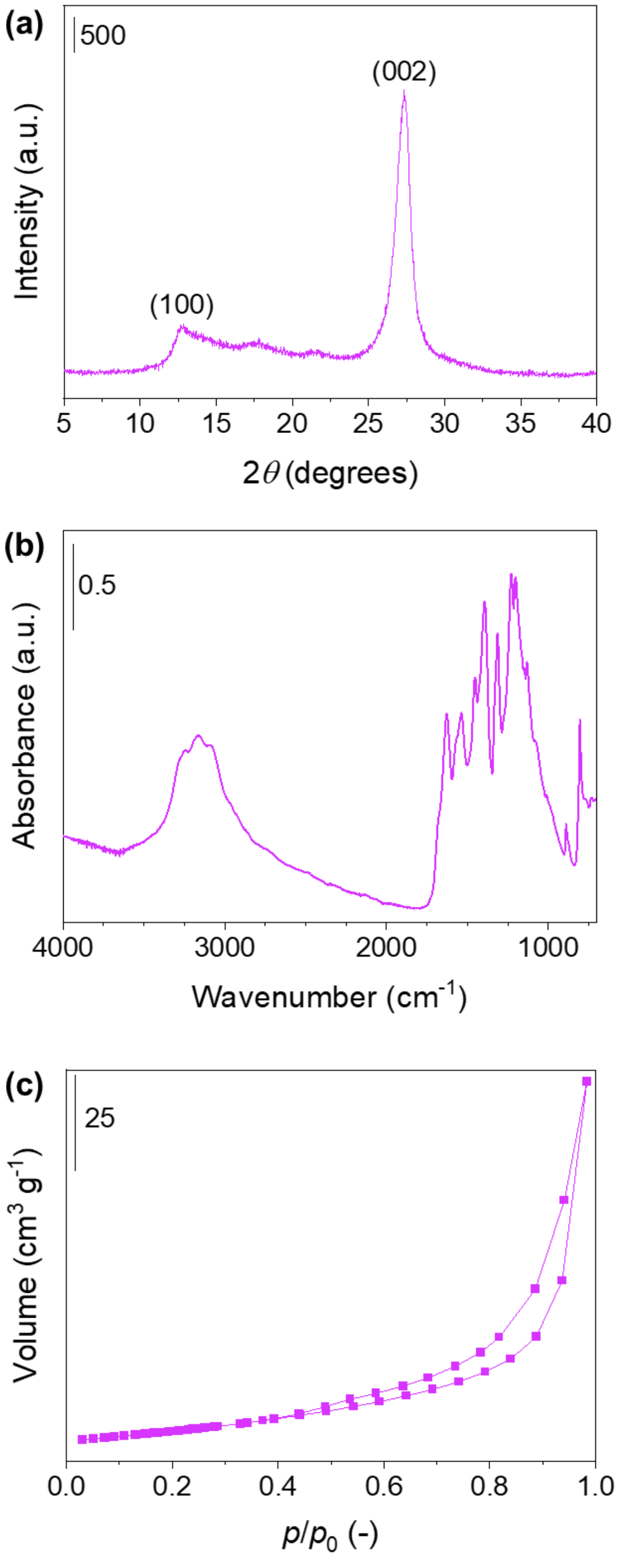

Figure S1. *Pristine* nCN_x *characterization including* ***(a)*** *XRD,* ***(b)*** *ATR-FTIR, and* ***(c)*** N_2 *physisorption isotherm of* nCN_x.

Table S2. *Elemental composition and textural properties of nCN$_x$.*

Catalyst	C[a] (wt.%)	N[a] (wt.%)	H[a] (wt.%)	C/N	Ni[b] (wt.%)	S_{BET}[c] (m^2 g^{-1})	d_{pore}[d] (nm)
Pristine nCN$_x$	35.48	58.69	2.34	0.60	-	28	21.06

[a]*CHN combustion analysis.* [b]*ICP-OES.* [c]*BET specific surface area from* N_2 *isotherms.* [d]*BJH pore diameters.*

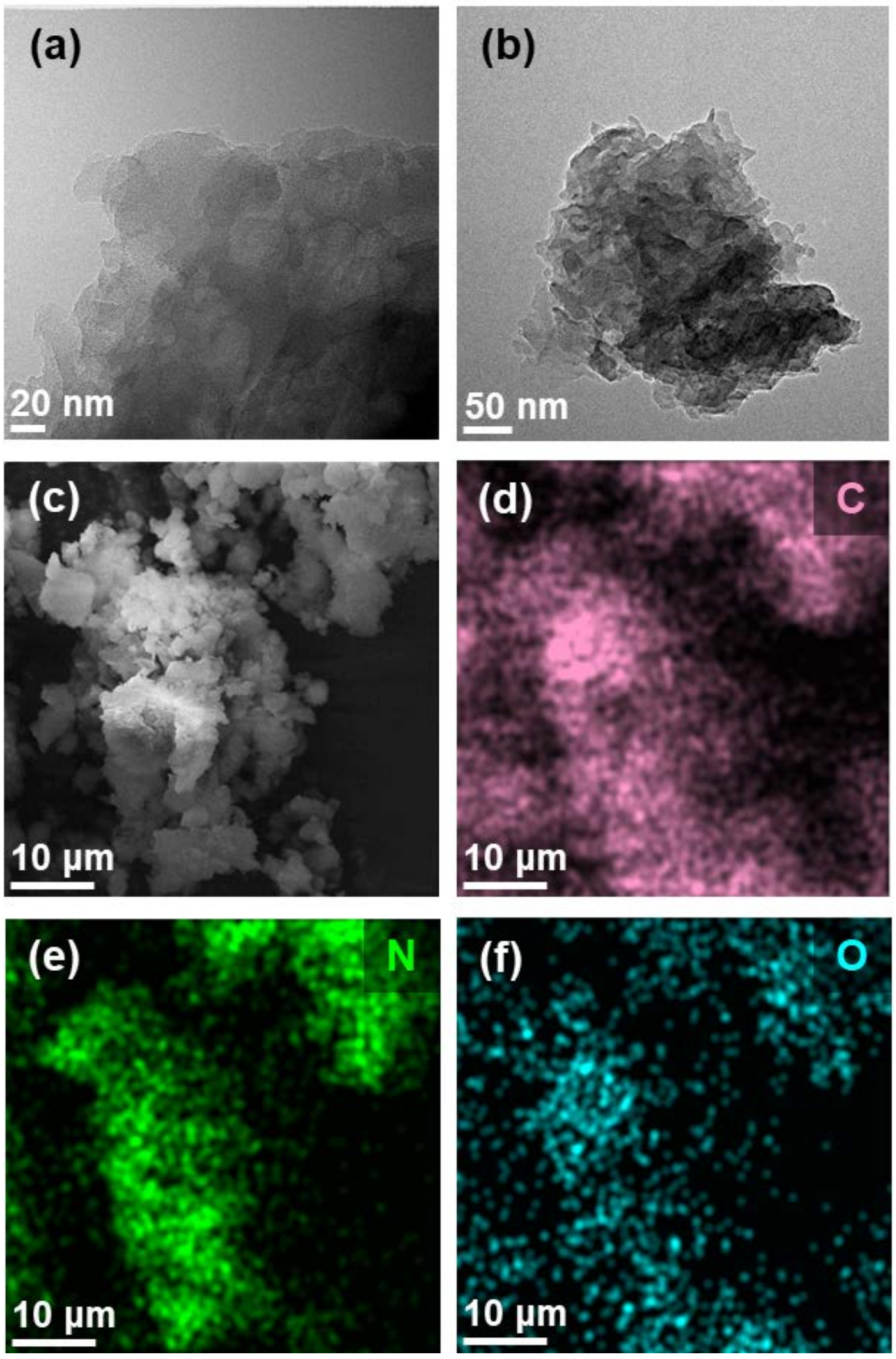

Figure S2*. Microscopic analysis of the pristine nCN$_x$. (**a**, **b**) TEM images of nCN$_x$, (**c**) SEM image, and EDX maps of elemental (**d**) C, (**e**) N and (**f**) O.*

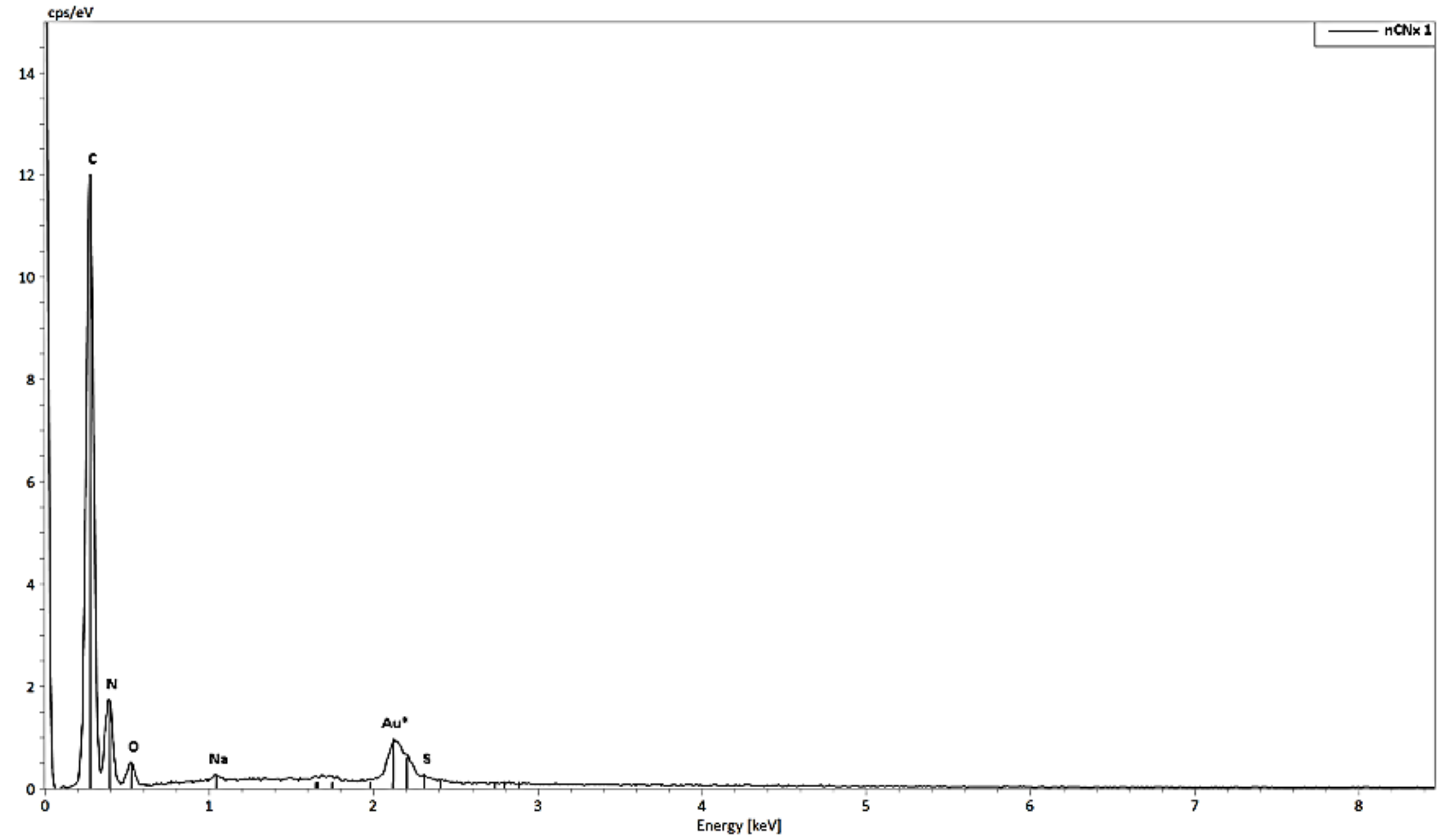

Figure S3. *EDX spectrum of the fresh nCN$_x$.*

Reaction optimization

The crude reaction products were analyzed using high-performance liquid chromatography (HPLC). Upon completion of the reaction, a 100 µL aliquot was taken and diluted with acetonitrile in a 2 mL HPLC vial. The conversion of product **3aa** was determined using an Agilent 1200 instrument equipped with a UV-Vis detector (G1315D) set to 210 nm. Samples and standards (5 µL each) were injected directly onto a 250 mm × 4.6 mm Hypersil GOLD^{TM} aQ column (Thermo Scientific) with 5 µm particle size and 175 Å pore size. A calibration curve was constructed (**Figure S4b**) by measuring the area under the curve (AUC) for different **3aa** solutions corresponding to yields of 100% (0.1 M), 75% (0.075 M), 50% (0.05 M), 25% (0.025 M), and 12.5% (0.0125 M).

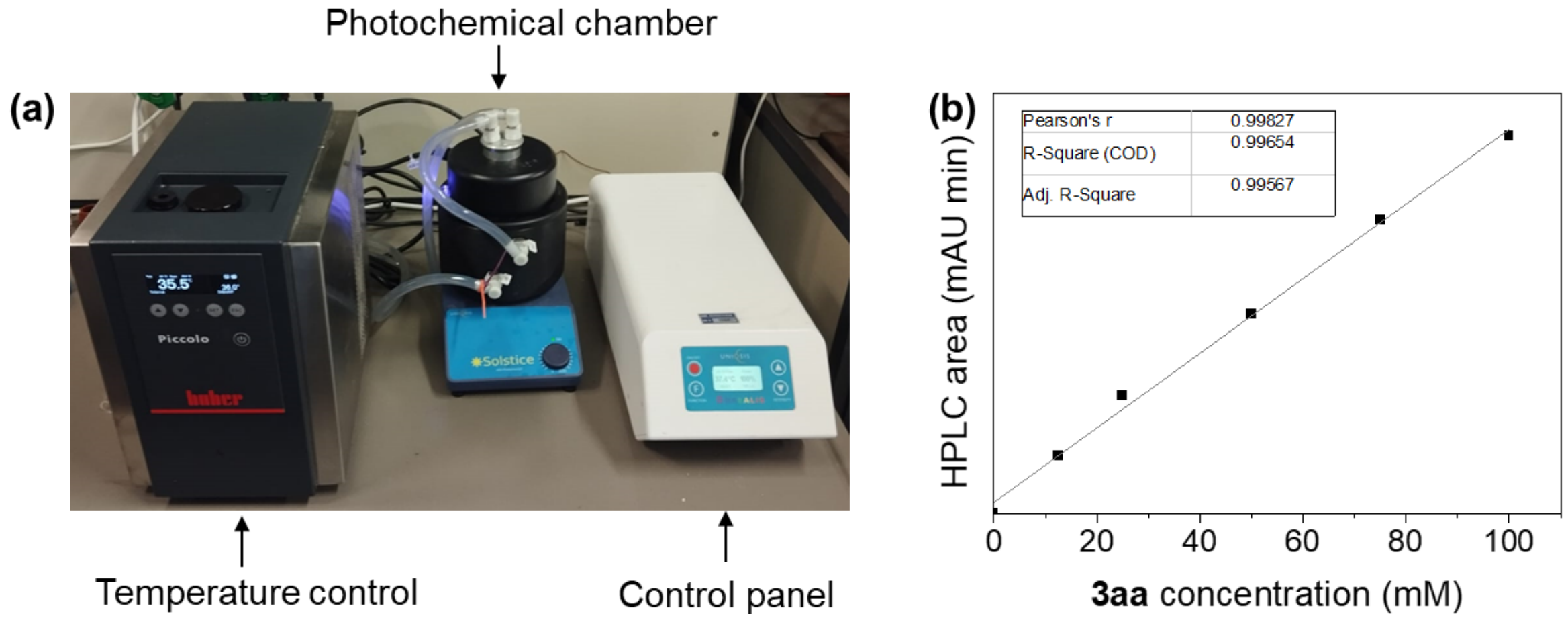

Figure S4. *(**a**) Uniqsis photoreactor used for photocatalytic experiments. (**b**) High-performance liquid chromatography calibration curve for the model product **3aa**.*

Table S3. *Optimization of the wavelength.*

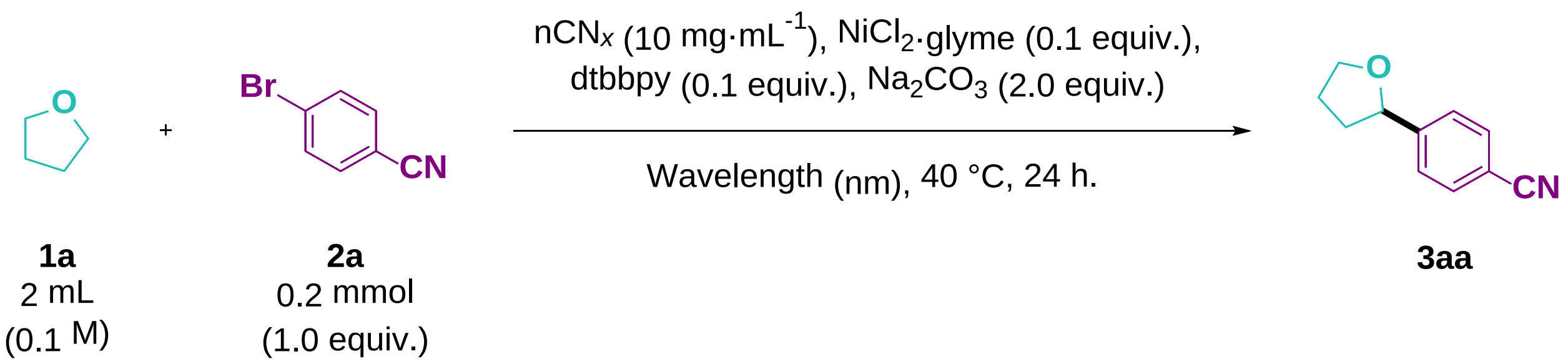

Entry	Wavelength (nm)	Yield of **3aa**[a]
1	Purple (420 nm)	23%
2	Blue (460 nm)	3%
3	Green (520 nm)	n.d.[b]

[a]Yields were calculated by HPLC using a calibration curve. [b]Not detected.

Table S4. *Optimization of reaction concentration.*

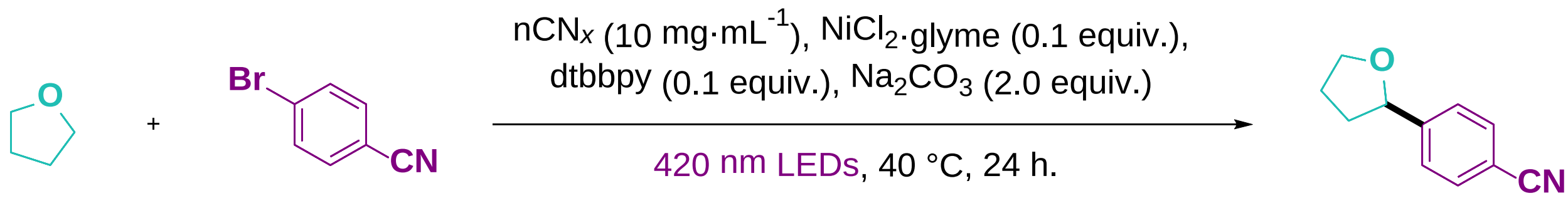

1a mL (M) | **2a** 0.2 mmol (1.0 equiv.) | **3aa**

Entry	**1a** mL (M)	Yield of **3aa**[a]
1	1 mL (0.2 M)	11%
2	2 mL (0.1 M)	23%
3	3 mL (0.067 M)	31%
4	4 mL (0.05 M)	35%
5	1.0 equiv.	3%[b]
6	10.0 equiv.	17%[b]

[a]*Yields were calculated by HPLC using a calibration curve.* [b]*The reaction was carried out using MeCN (2 mL) as solvent.*

Table S5. *Optimization of nCN$_x$ concentration.*

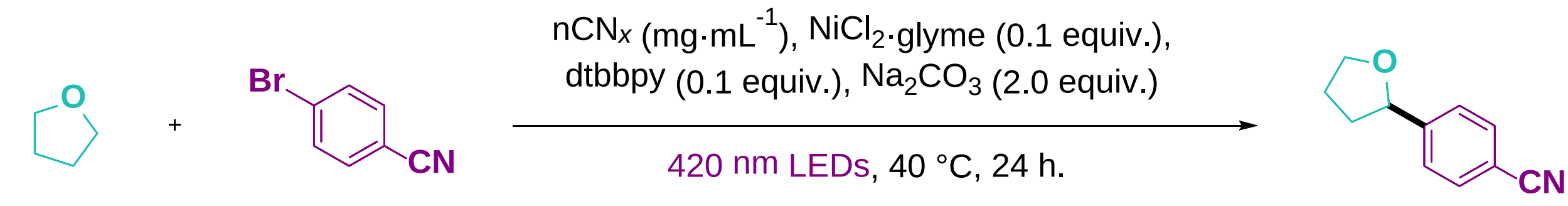

1a 4 mL (0.05 M) + **2a** 0.2 mmol (1.0 equiv.) → **3aa**

Entry	nCN$_x$ (mg·mL^{-1})	Yield of **3aa**[a]
1	7.5 mg·mL^{-1}	27%
2	10 mg·mL^{-1}	35%
3	12.5 mg·mL^{-1}	43%
4	15 mg·mL^{-1}	40%
5	17.5 mg·mL^{-1}	22%

[a]*Yields were calculated by HPLC using a calibration curve.*

Table S6. *Optimization of Nickel catalyst source.*

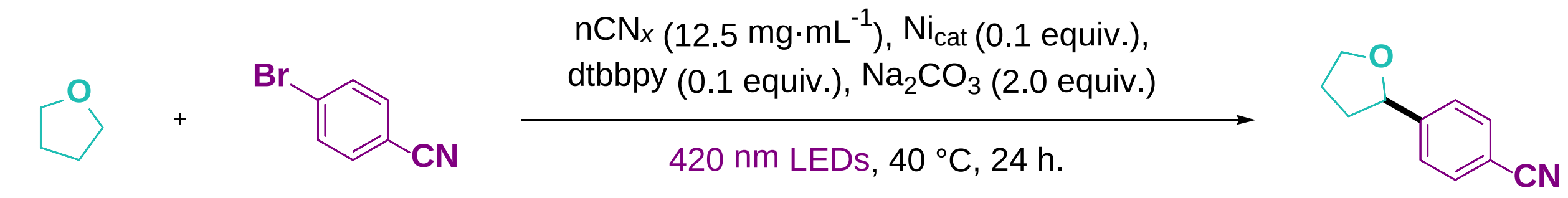

1a 4 mL (0.05 M) + **2a** 0.2 mmol (1.0 equiv.) → **3aa**

Entry	Ni_{cat} source	Yield of **3aa**[a]
1	$NiCl_2$·glyme	43%
2	$NiCl_2$	36%
3	$NiBr_2$·glyme	35%
4	$NiBr_2$	35%
5	$Ni(OCOCH_3)_2·4H_2O$	29%
6	$Ni(NO_3)_2·6H_2O$	32%
7[b]	$NiCl_2$·glyme	32%
8[c]	$NiCl_2$·glyme	24%

[a]*Yields were calculated by HPLC using a calibration curve.* [b]*Using 0.2 equiv. of* $NiCl_2$*·glyme.* [c]*Using 0.05 equiv. of* $NiCl_2$*·glyme.*

Table S7. Optimization of ligand screening.

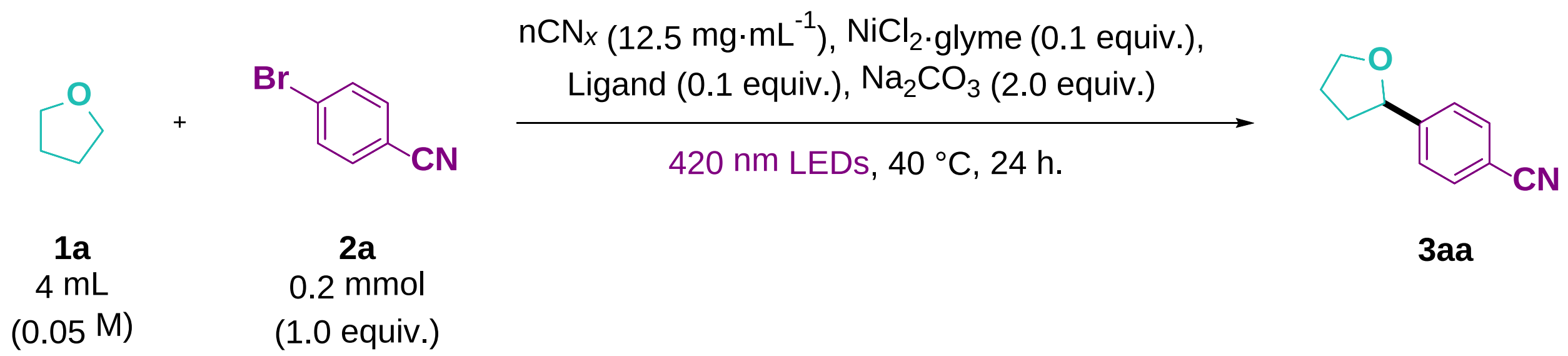

Entry	Ligand	Yield of **3aa**[a]
1	4,4'-dimethoxybipyridine	29%
2	2,2'-bipyridine	24%
3	4,4'-di-*tert*-butyl-2,2'-bipyridine	43%
4	4,4'-dimethylbiphenyl	16%
5[b]	4,4'-di-*tert*-butyl-2,2'-bipyridine	37%

[a]Yields were calculated by HPLC using a calibration curve. [b]Using 0.15 equiv. of 4,4'-di-tert-butyl-2,2'-bipyridine (in 0.05 equiv. excess).

Table S8. *Optimization of base equivalents.*

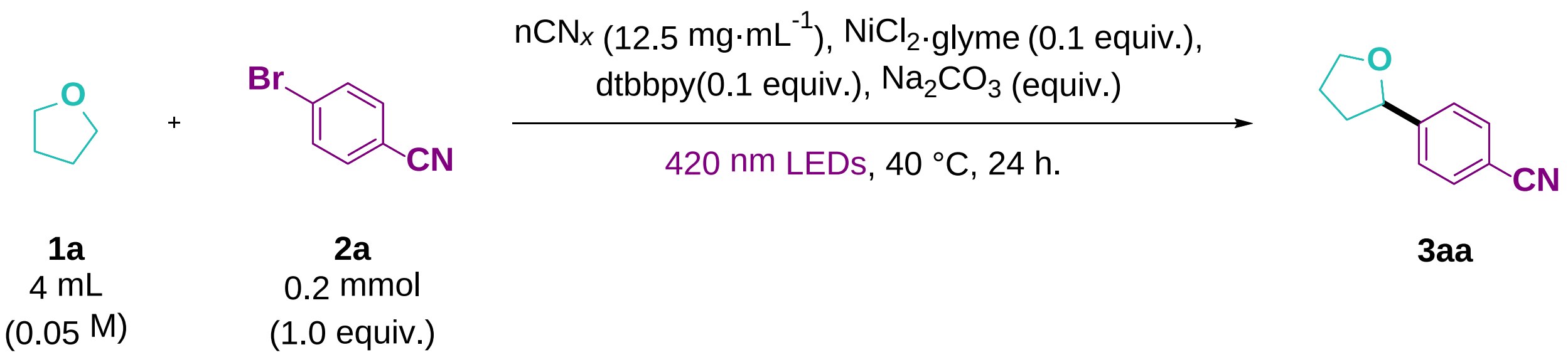

Entry	Na_2CO_3 equiv.	Yield of **3aa**[a]
1	1	30%
2	1.5	34%
3	2	43%
4	3	33%

[a]*Yields were calculated by HPLC using a calibration curve.*

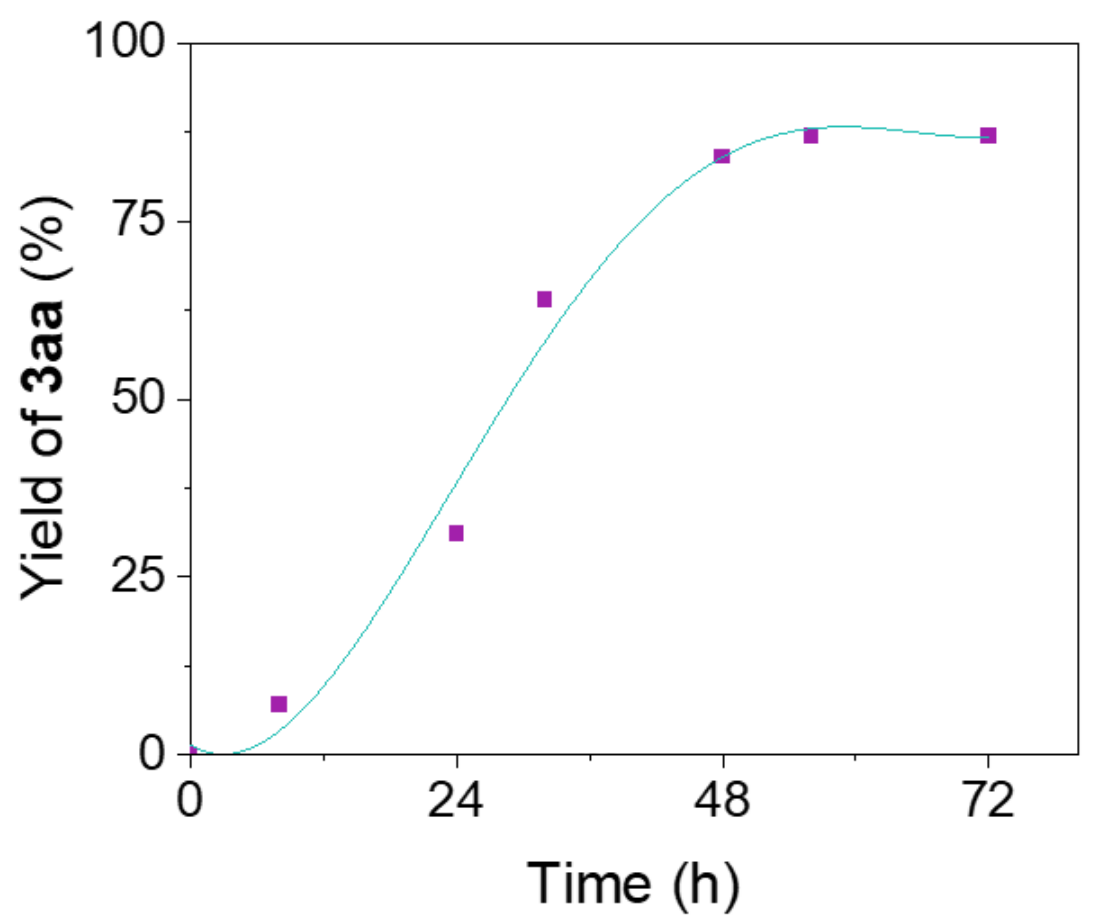

Figure S5. *Reaction kinetic profile. Yields were calculated by HPLC using a calibration curve.*

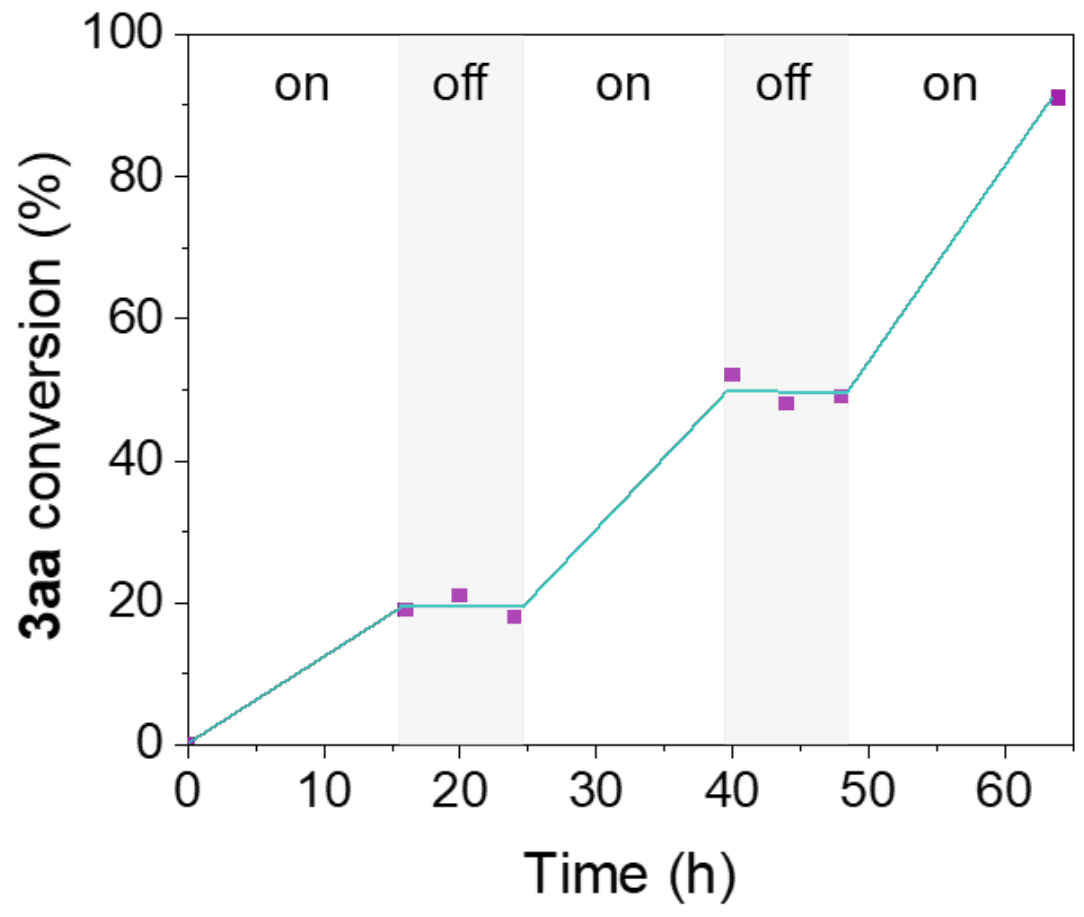

Figure S6. *Light on/off experiment. Product formation occurring only when the reaction mixture was exposed to light. During the dark intervals, the reaction essentially ceased. Yields were calculated by HPLC using a calibration curve.*

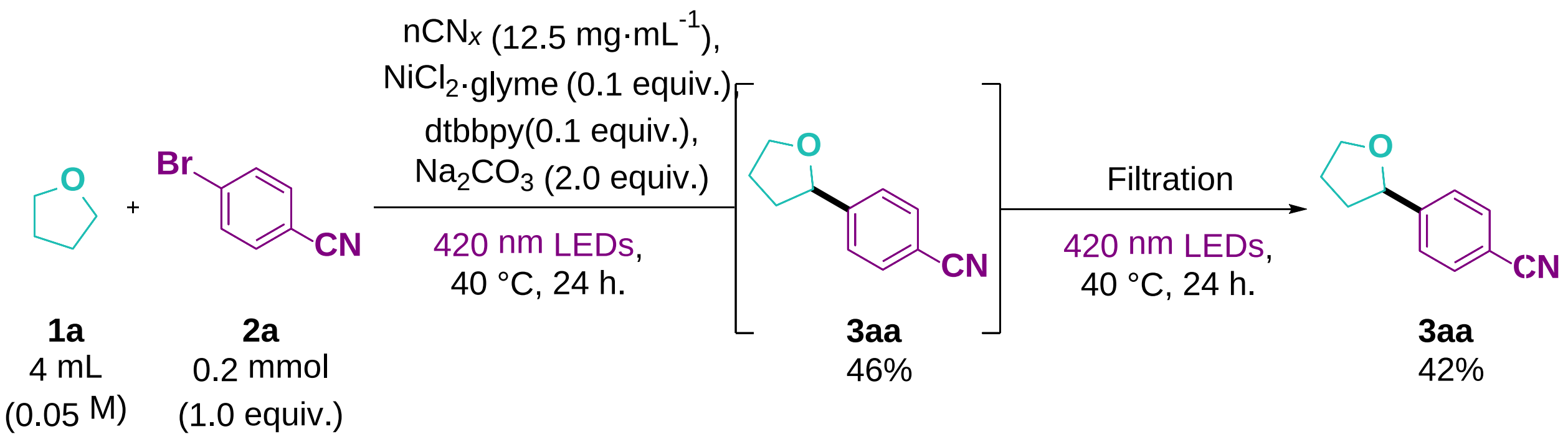

Figure S7. *Hot filtration experiment. The reaction was conducted under the standard conditions for 24 h, affording **3aa** in 46% yield. The carbon nitride photocatalyst was then removed by hot filtration, and the resulting filtrate was irradiated for an additional 24 h under otherwise identical conditions. No further product formation was observed, and **3aa** was obtained in 42% yield after the second irradiation period. Yields were calculated by HPLC using a calibration curve.*

Apparent quantum yield calculation

The quantum yield (QY) of a photocatalytic process describes the efficiency with which light drives a chemical reaction. It is defined as the ratio between the number of electrons involved in the reaction and the total number of photons absorbed by the system under irradiation at a specific wavelength. In heterogeneous photocatalytic systems, however, accurately determining the number of photons actually absorbed by the catalyst is challenging. Phenomena such as light scattering, reflection, and other optical losses prevent a precise measurement of the absorbed photon flux, making a direct calculation of the true quantum yield impractical, besides the photogenerated charge carriers in semiconductor photocatalysts such as carbon nitride are susceptible to bulk and surface recombination, which competes with productive charge transfer to the substrate. To address this limitation, the number of absorbed photons is replaced with the number of incident photons reaching the system. The efficiency calculated using this approximation is known as the apparent quantum yield (AQY). The equation used to determine the apparent quantum yield is given below.

$$AQY = \frac{Ne}{Np} \times 100\% = \frac{1.2 \times 10^{8}(\nu \times K)}{(I \times A \times \lambda)} \times 100\% \qquad \text{(Eq. S1)}$$

where:

Ne: Total number of electrons transferred

Np: Number of incident photons

ν: Reaction rate ($mol \cdot s^{-1}$) = 1.94×10^{-9}

K: Number of transferred electrons = 1

I: Light power density ($W \cdot m^{-2}$) = 1750

A: Incident light area (m^2) = 0.00035

λ: Incident light wavelength (nm) = 420

Sustainability analysis

Table S9. *Assessment of the sustainability of the synthetic methodology.*

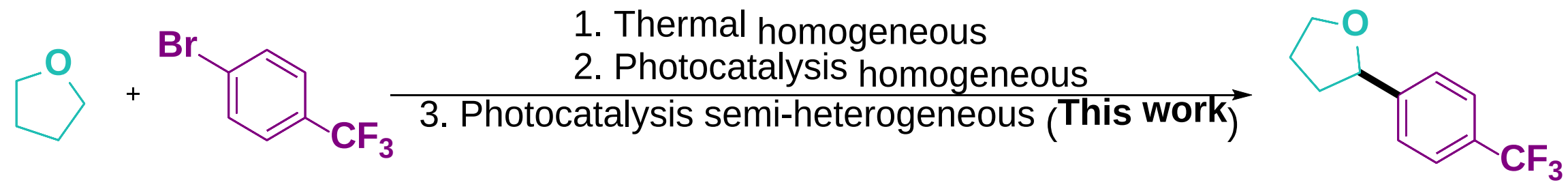

Entry	Conditions	Reference	Product yield	Reaction time (h)	E-Factor (-)	Process improvement (%)
1	Thermal homogeneous	[2]	37%	12	34.0	-
2	Photocatalysis homogeneous	[4]	63%	72	16.9	50
3	Semi-heterogeneous photocatalysis	This work	88%	48	12.2	64

Continuous-flow experiment

A reaction mixture containing substrate **2a** (0.6 mmol, 1.0 equiv), $NiCl_2$·glyme (0.1 equiv.), dtbbpy (0.1 equiv.), and quinuclidine (2.0 equiv.) dissolved in 12 mL of THF was continuously pumped through a packed-bed reactor loaded with nCN$_x$ (100 mg) and glass beads (2.5 wt.%) at a flow rate of 10 μL min^{-1}, providing a residence time of 3 h. Quinuclidine was employed in place of Na_2CO_3 to improve the solubility of the base under these reaction conditions, thereby preventing potential clogging within the flow system.

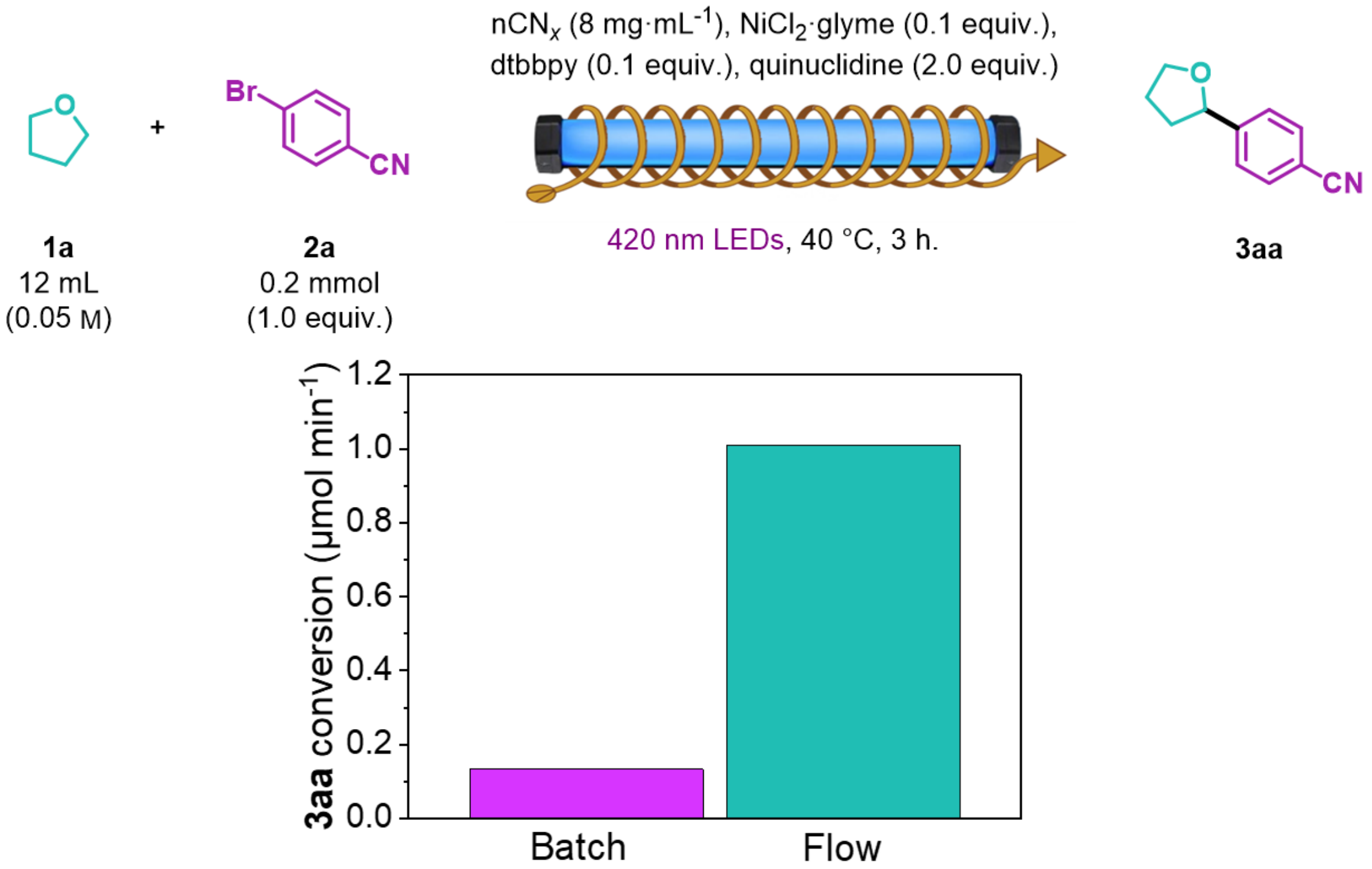

Figure S8. *Reaction profile comparison of batch and continuous-flow photocatalytic C–H arylation of THF with 4-bromobenzonitrile under visible-light irradiation.*

Post-catalysis characterization

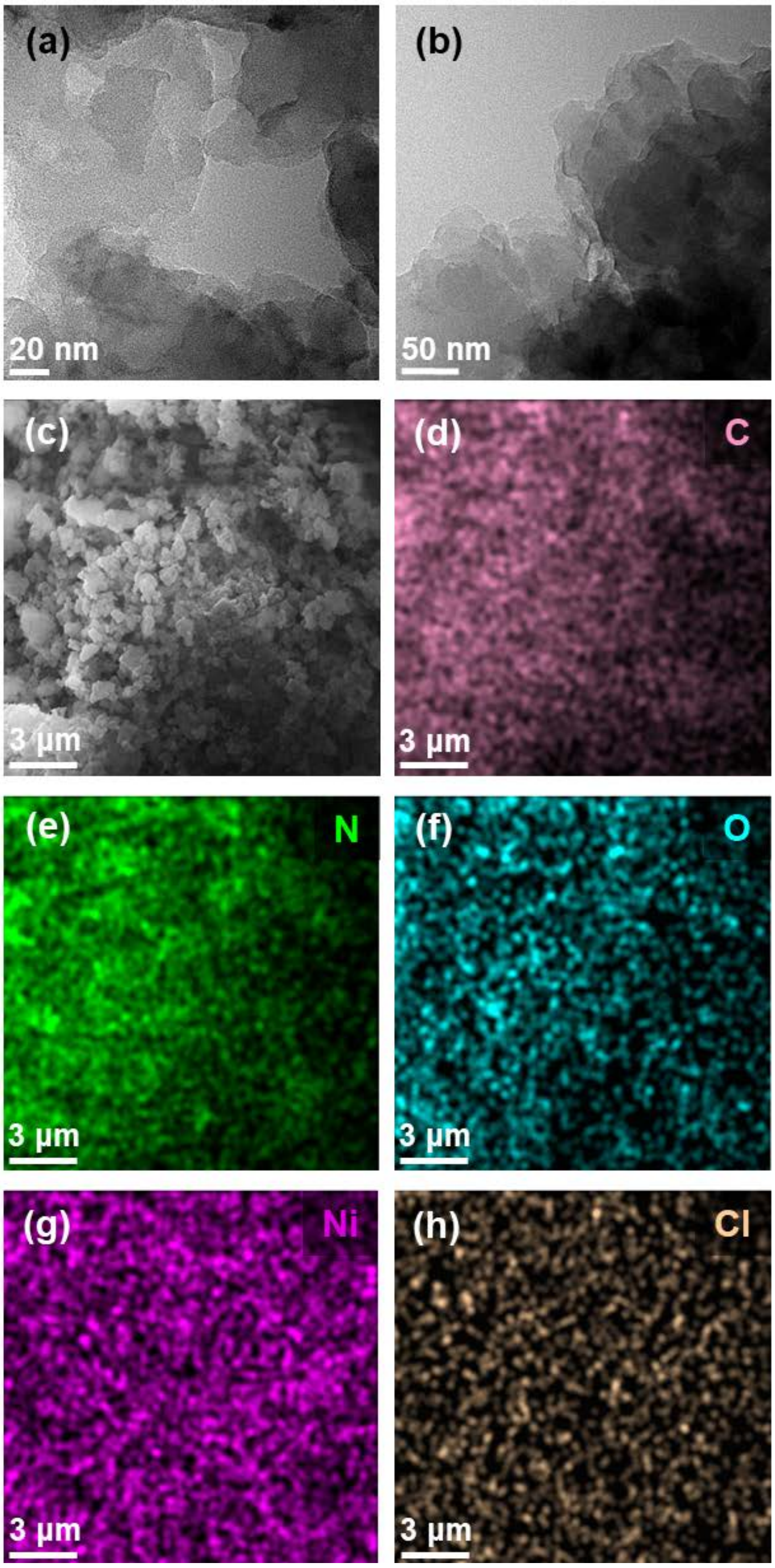

Figure S9. *Microscopic analysis of the recycled nCN$_x$. (**a**, **b**) TEM images of nCN$_x$, (**c**) SEM image, and EDX elemental maps of (**d**) C, (**e**) N, (**f**) O, (**g**) Ni and (**h**) Cl.*

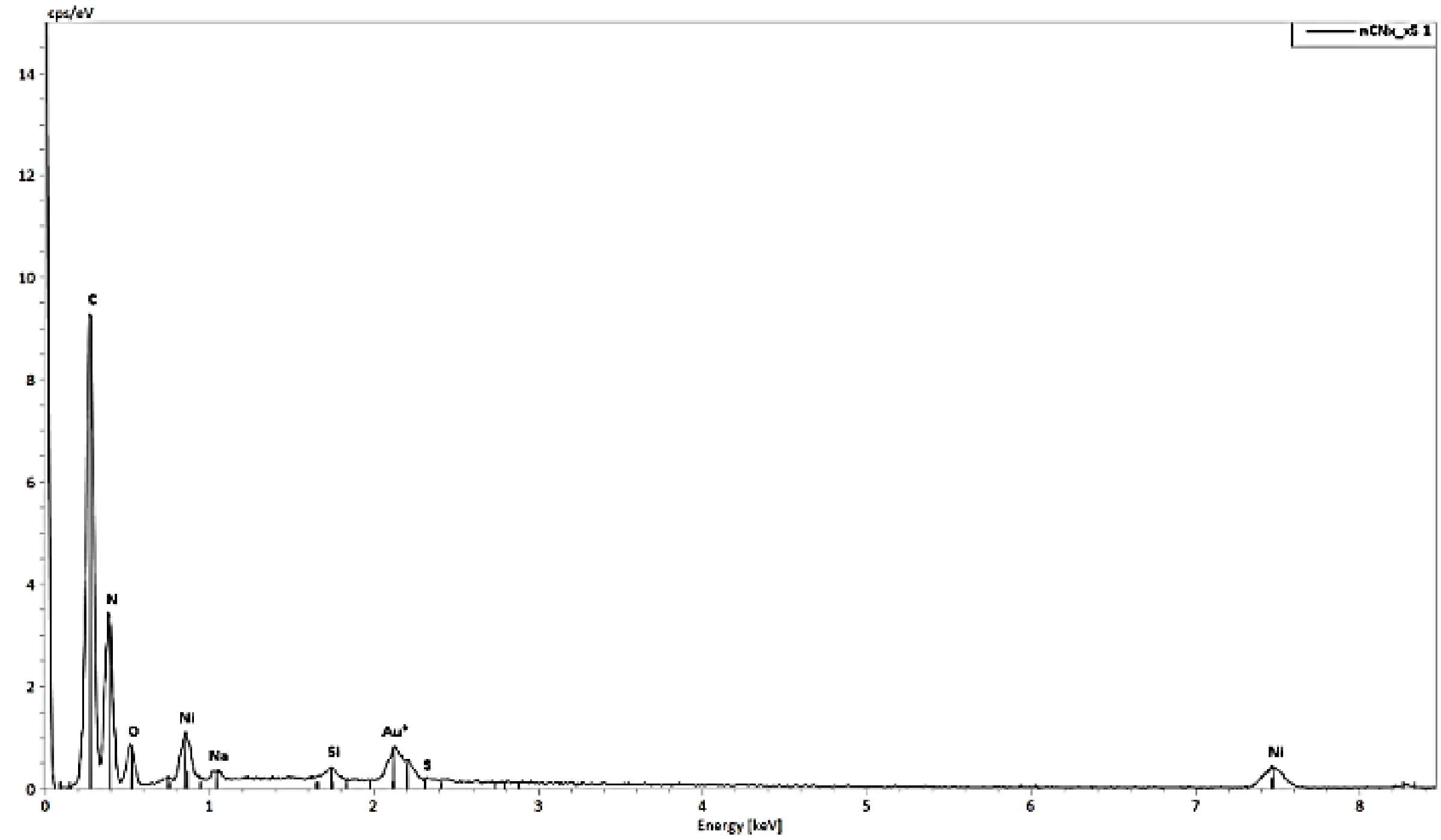

Figure S10. *EDX spectrum of the recycled nCN$_x$.*

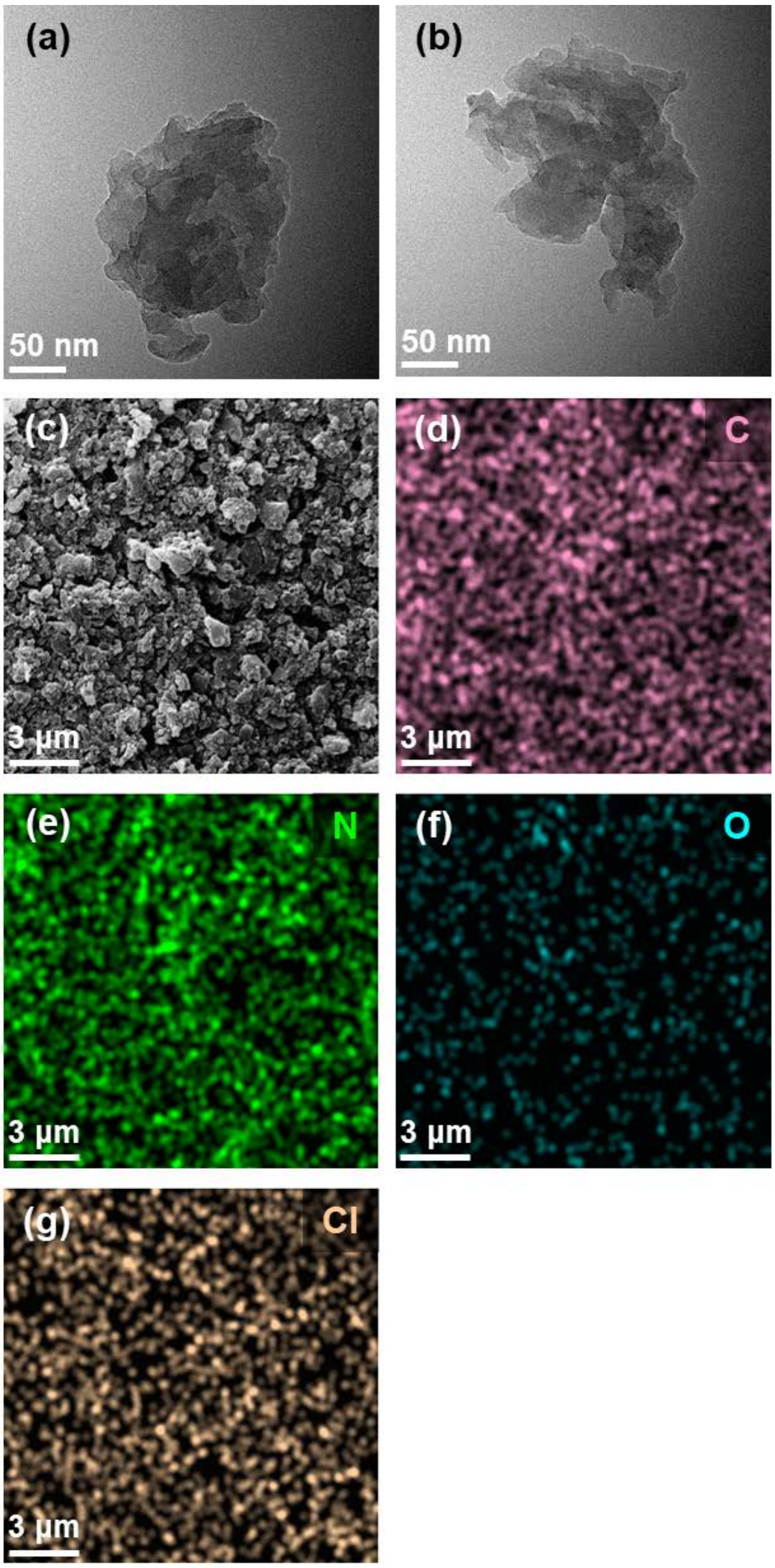

Figure S11. *Microscopic analysis of the washed recycled nCN$_x$ including (**a**, **b**) TEM images of nCN$_x$, (**c**) SEM image, and EDX elemental maps of (**d**) C, (**e**) N, (**f**) O and (**g**) Cl.*

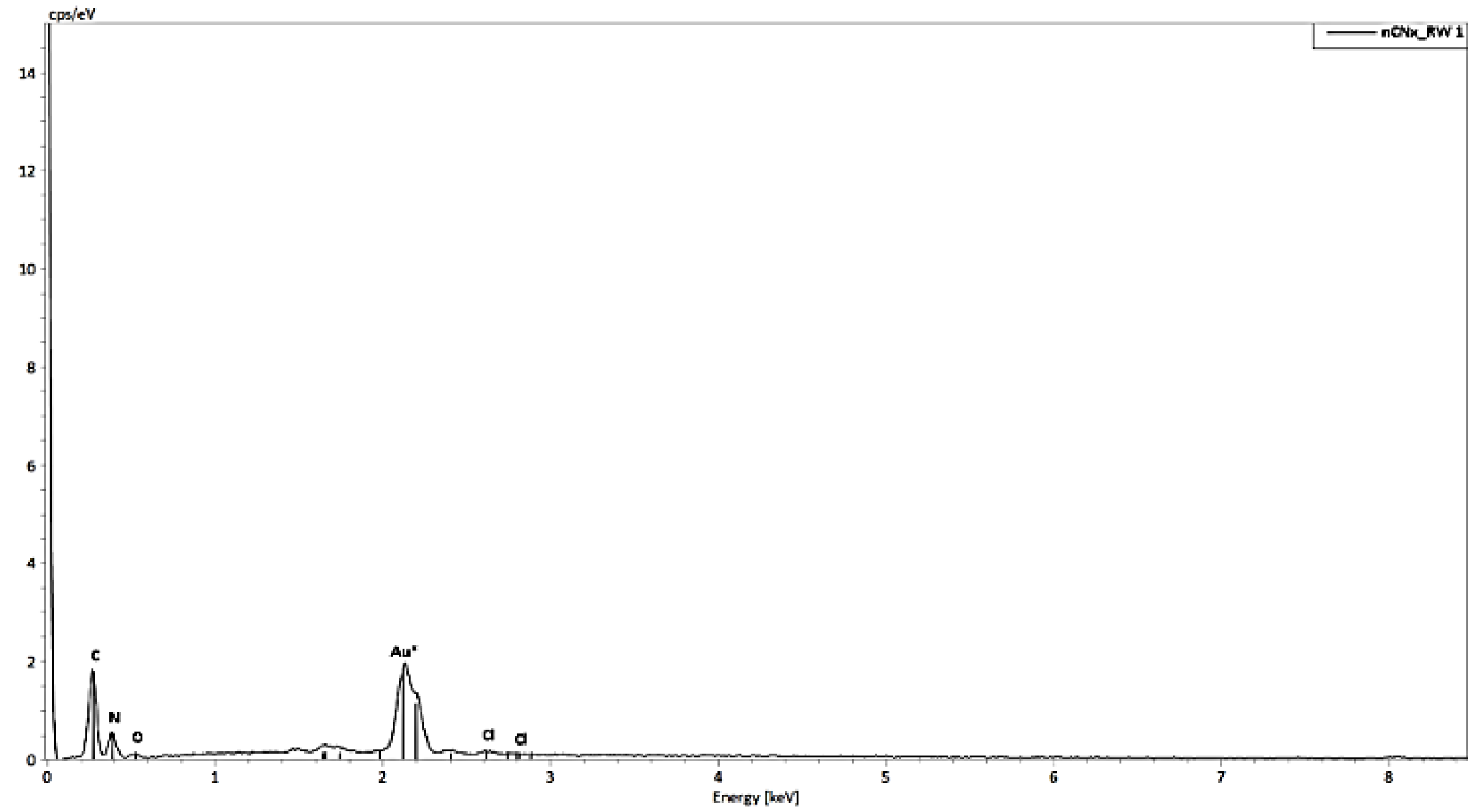

Figure S12. *EDX spectrum of the washed recycled nCN_x.*

Table S10. *Elemental composition and textural properties of the fresh and recycled nCN_x.*

Catalyst	C^a (wt.%)	N^a (wt.%)	H^a (wt.%)	C/N	Ni^b (wt.%)	S_{BET}^c (m^2 g^{-1})	d_{pore}^d (nm)
nCN_x-fresh	35.48	58.69	2.34	0.60	-	28	21.06
nCN_x-recycled	31.39	47.07	1.98	0.67	5.23	58	13.98

[a]CHN combustion analysis. [b]ICP-OES. [c]BET specific surface area from N_2 isotherms. [d]BJH pore diameters.

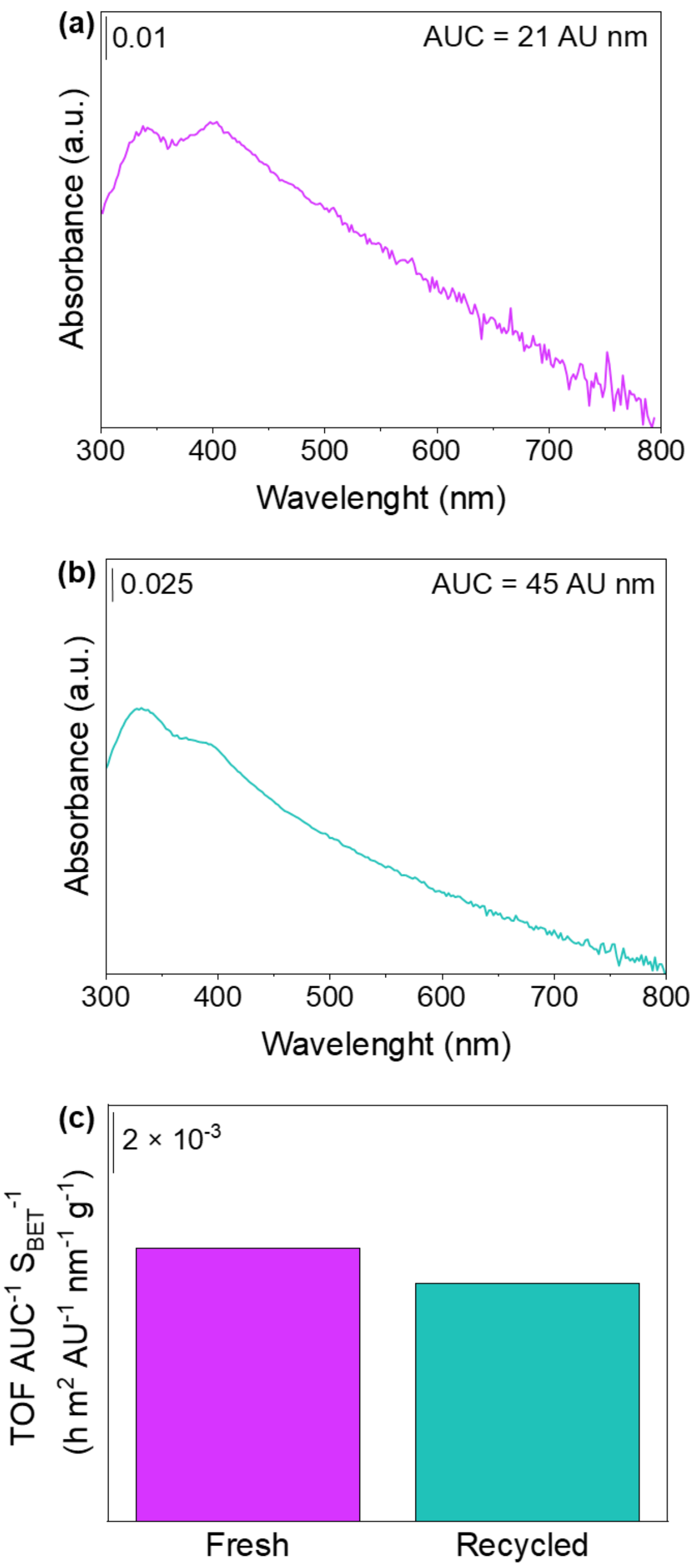

Figure S13. *Light absorption investigations. (**a**) UV-Vis of the fresh nCN$_x$. (**b**) UV-Vis of the 5-time recycled nCN$_x$. The area under the adsorption curve (AUC) was calculated from 300 to 800 nm. (**c**) TOF of the fresh and recycled nCN$_x$ was normalized according to their AUC and S$_{BET}$.*

Mechanistic studies

Cyclic voltammetry experiments. Electrochemical potentials were obtained with a standard set of conditions to maintain consistency. The samples were prepared with 0.06 mmol of substrate in 6 mL of 0.1M tetra-*n*-butylammonium tetrafluoroborate (TBATFB) in degassed acetonitrile. The measurements were performed in a three-electrode system, with a glassy carbon working electrode, a platinum wire counter electrode, a 0.1 M TBATFB Ag/$AgNO_3$ reference electrode, under inert atmosphere and at a scan rate of 100 mV s^{-1}. Oxidation potentials were measured by scanning in the positive direction and have been referenced against internal standard ferrocene.

Table S11. *Experimentally obtained oxidation peak potential and half-potential of organic solvents.*

Entry	Compound	$E_{p/2}$ (V vs. Ag/$AgNO_3$)	$E_{p/2}$ (V vs. Fc^+/Fc)
1	Fc^+/Fc	0.085	0
2	THF[a]	2.09	2.01
3	2-MeTHF	2.07	1.98
4	Et_2O[a]	N.D.	N.D.
5	Toluene	1.82	1.73
6	Mesitylene	1.70	1.62
7	DMF	1.81	1.72
8	DMA	1.62	1.53
9	NMP	1.62	1.54
10	Dioxane	2.28	2.19
11	DME	2.12	2.03

[a]*Distilled compound to remove BHT stabilizers.*

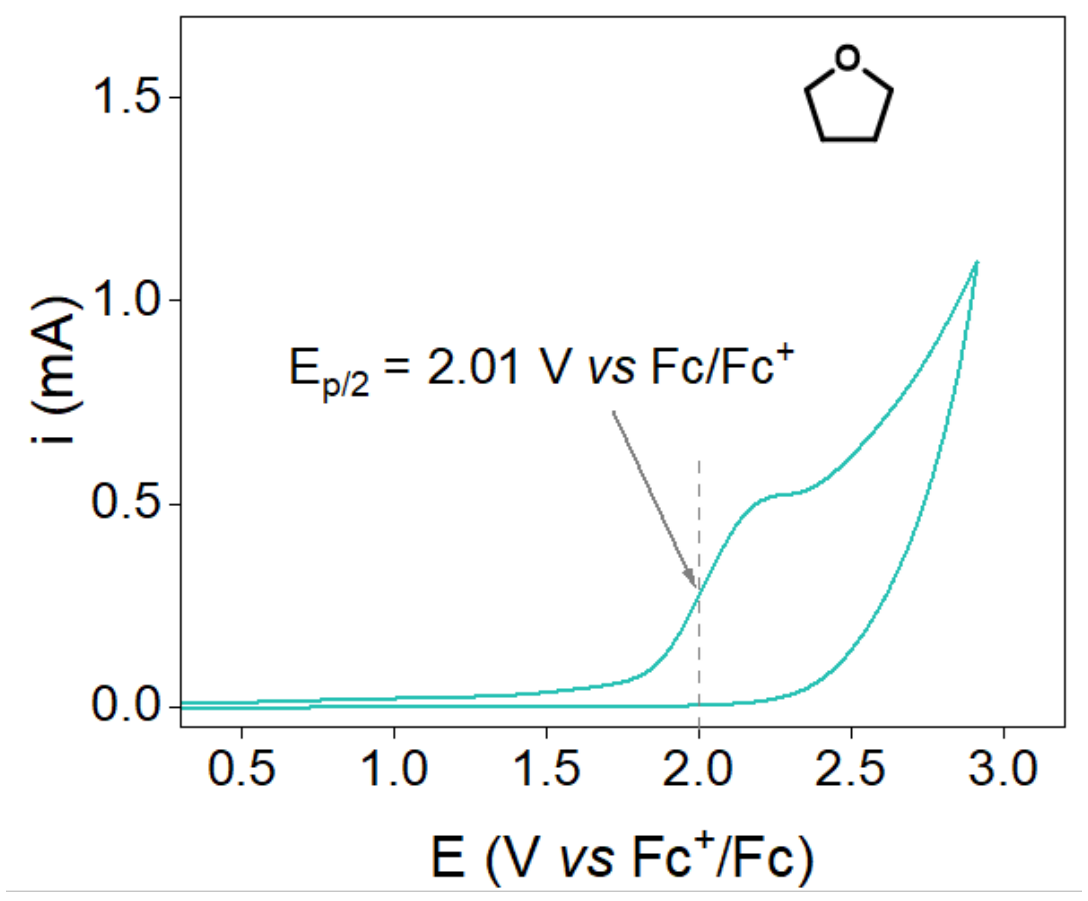

Figure S14. *Cyclic voltammogram of THF.*

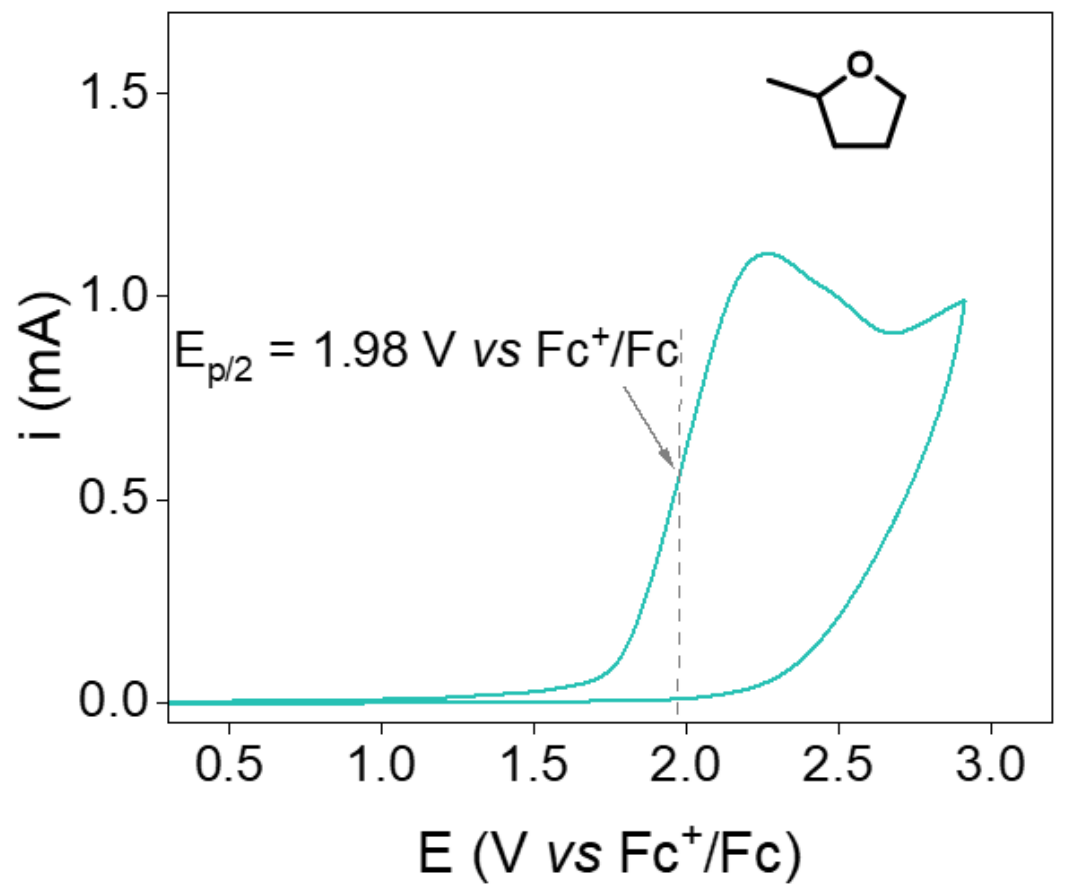

Figure S15. *Cyclic voltammogram of 2-Methyl-THF.*

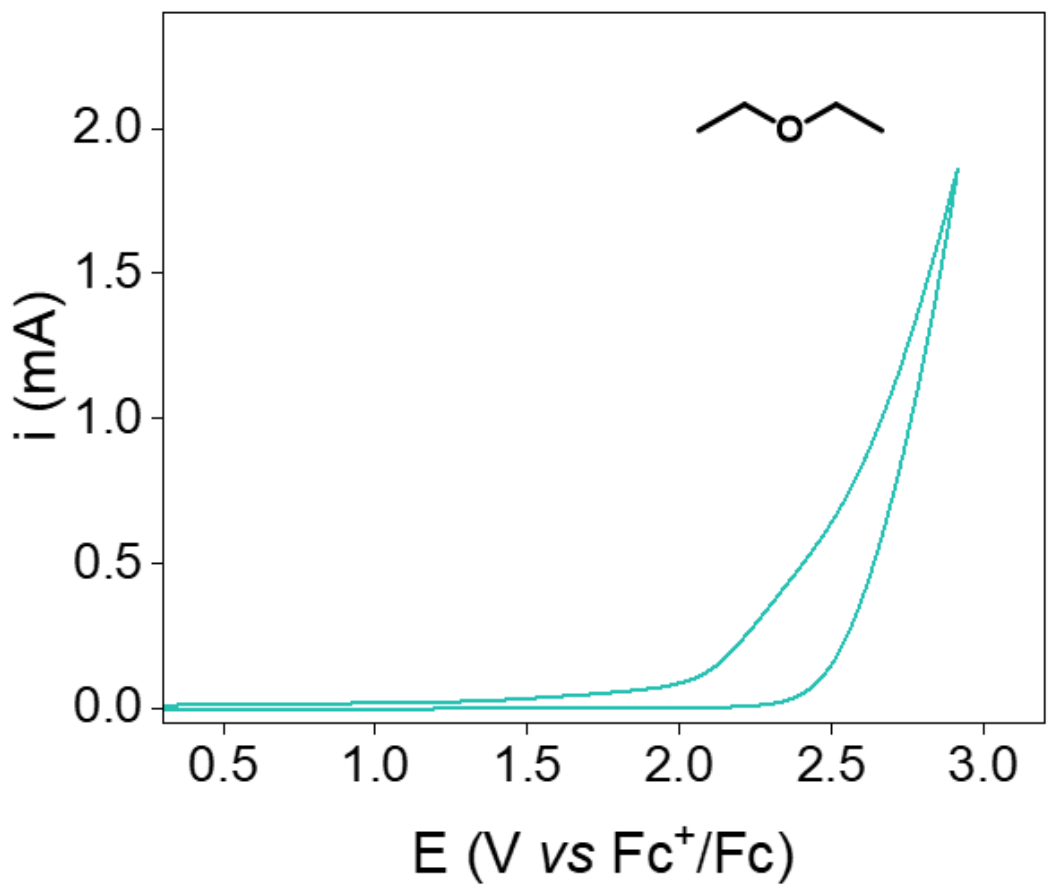

Figure S16. *Cyclic voltammogram of diethyl ether.*

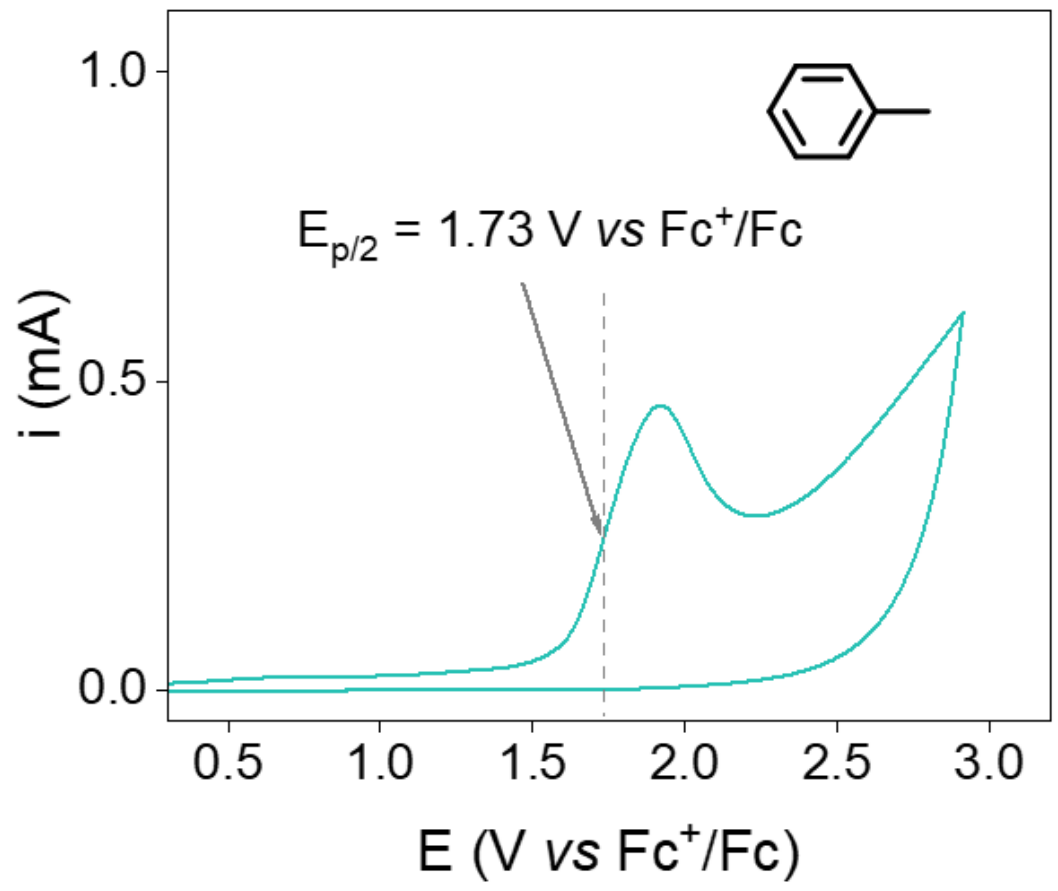

Figure S17. *Cyclic voltammogram of toluene.*

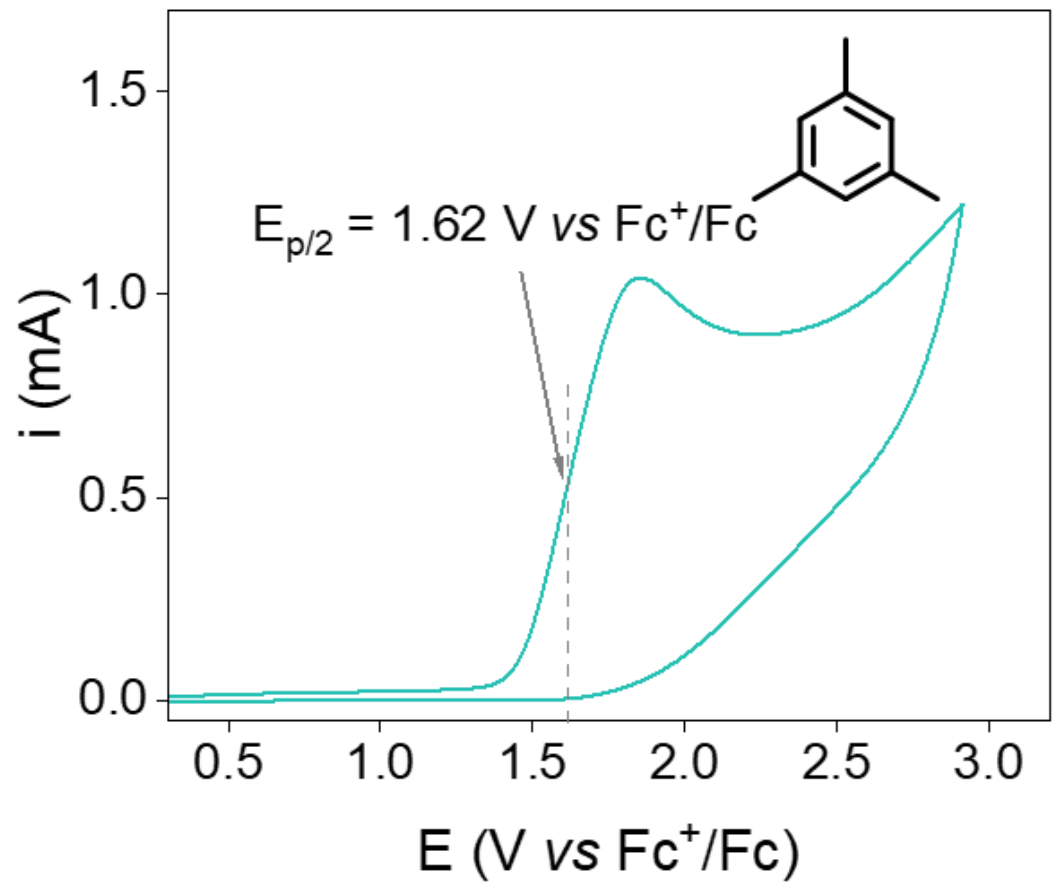

Figure S18. *Cyclic voltammogram of mesitylene.*

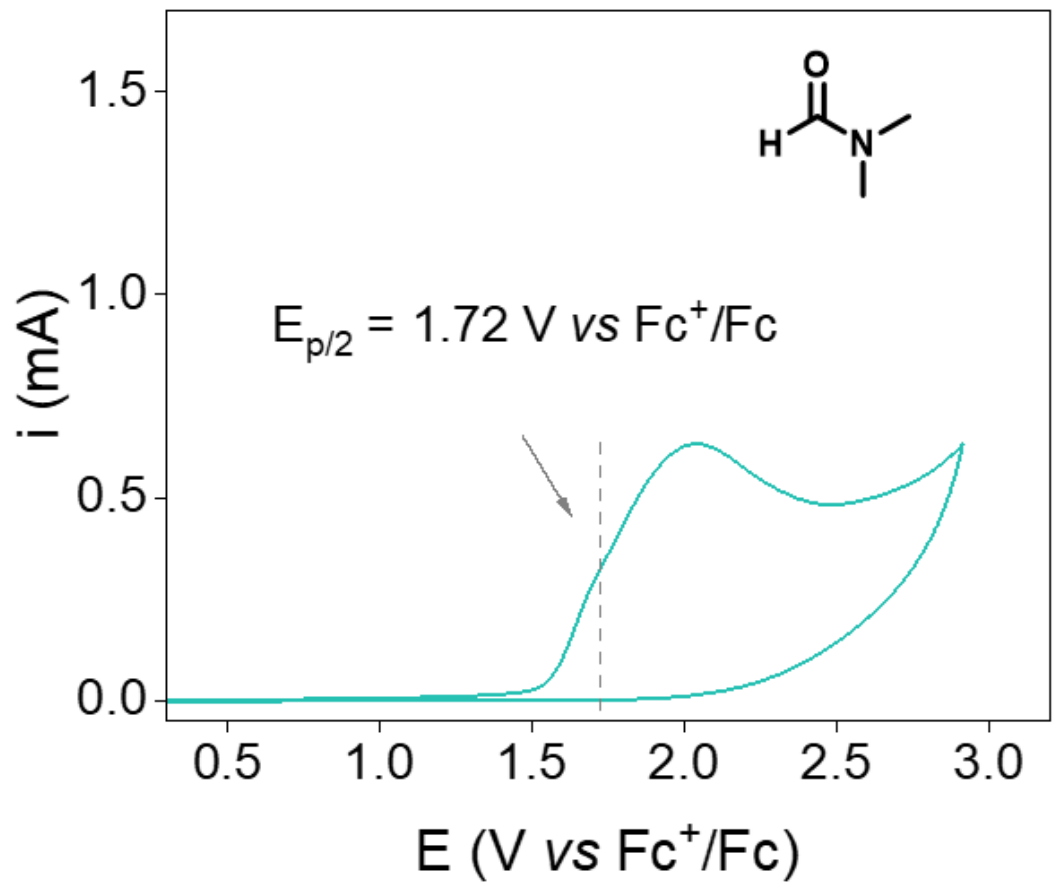

Figure S19. *Cyclic voltammogram of DMF.*

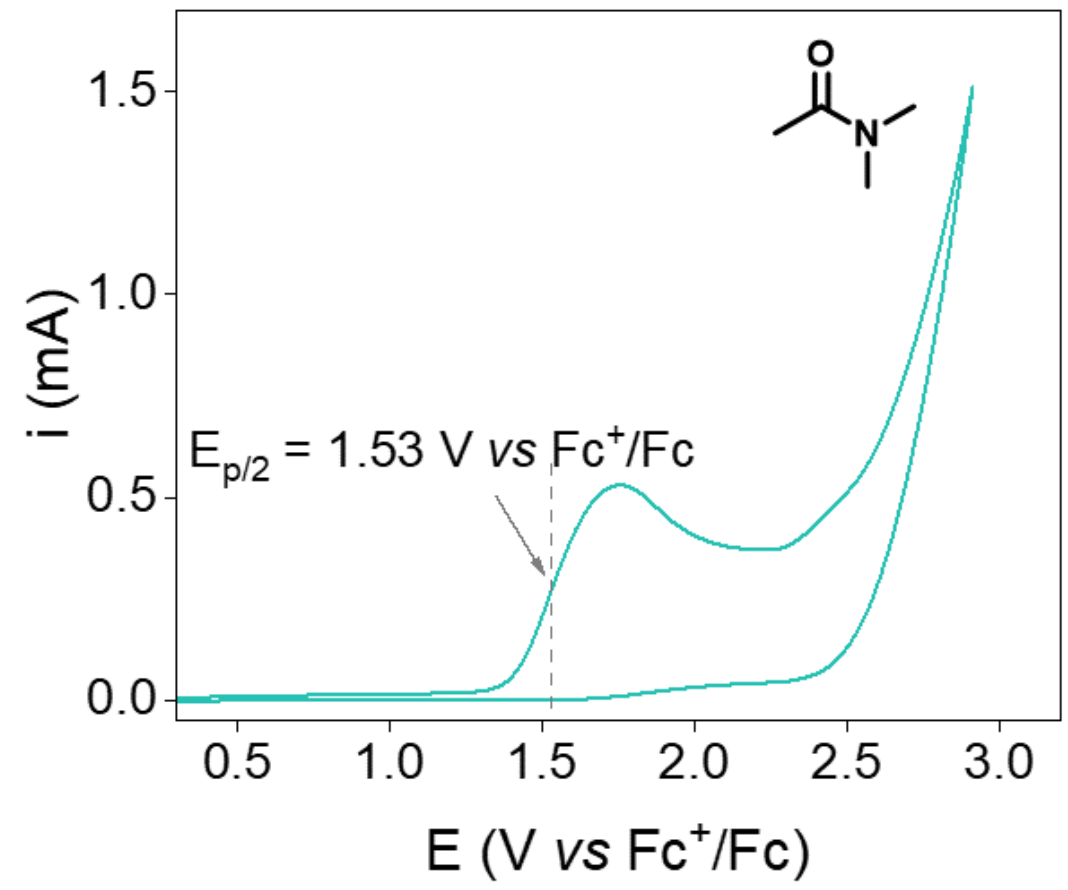

Figure S20. *Cyclic voltammogram of DMA.*

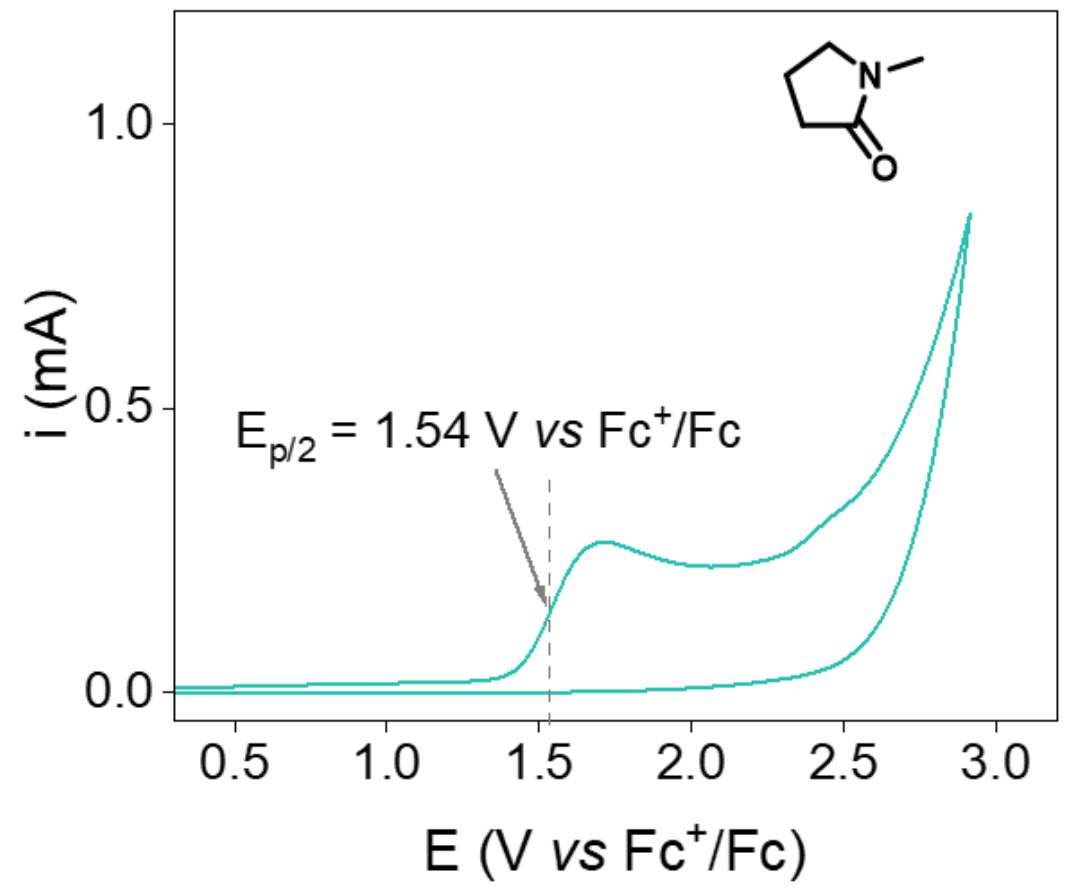

Figure S21. *Cyclic voltammogram of NMP.*

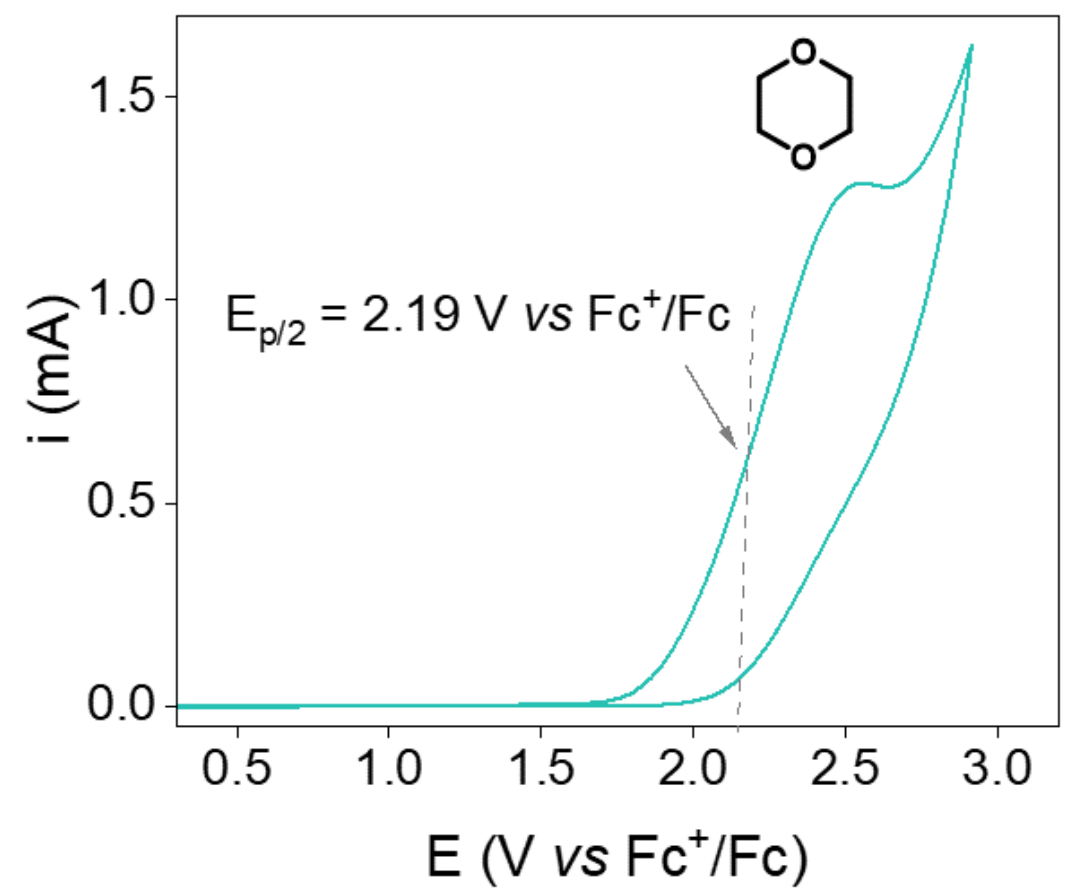

Figure S22. *Cyclic voltammogram of dioxane.*

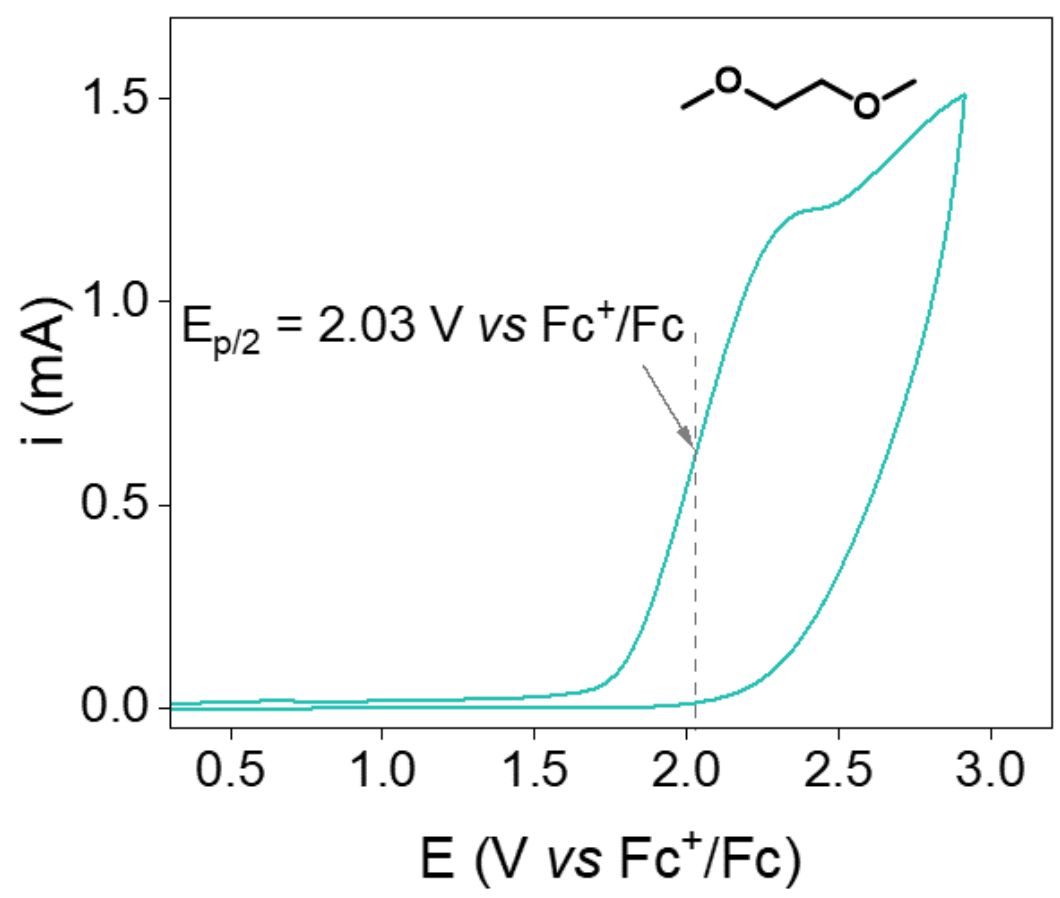

Figure S23. *Cyclic voltammogram of DME.*

DFT Calculations.

Energy transfer mechanism. In this section we report and briefly discuss two mechanisms based on an energy transfer process to activate the reactivity that can be used to describe the present experimental evidence. They are indicated in the following as M2 and M3.

Mechanism M2. The mechanism was formulated based on a literature reaction mechanism proposed by Molander *et al*.[1] to describe the reactivity for a similar system, in which the photoexcitation is, however, performed using an Ir complex. It is composed of the following reaction steps:

$nCN_x \rightarrow {}^3nCN_x$ [R10]

${}^3nCN_x$ + (bpy)Ni^IBr• → nCN_x + (bpy)Ni^0 + Br• [R11]

THF + Br• → THF• + HBr [R12]

(bpy)Ni^0 + Br-PhCN → (bpy)Ni^{II}Br(PhCN) [R13]

(bpy)Ni^{II}Br(PhCN) + THF• → (bpy)Ni^{III}THF(PhCN)Br• [R14]

(bpy)Ni^{III}THF(PhCN)Br• → (bpy)Ni^IBr• + THF-PhCN [R15]

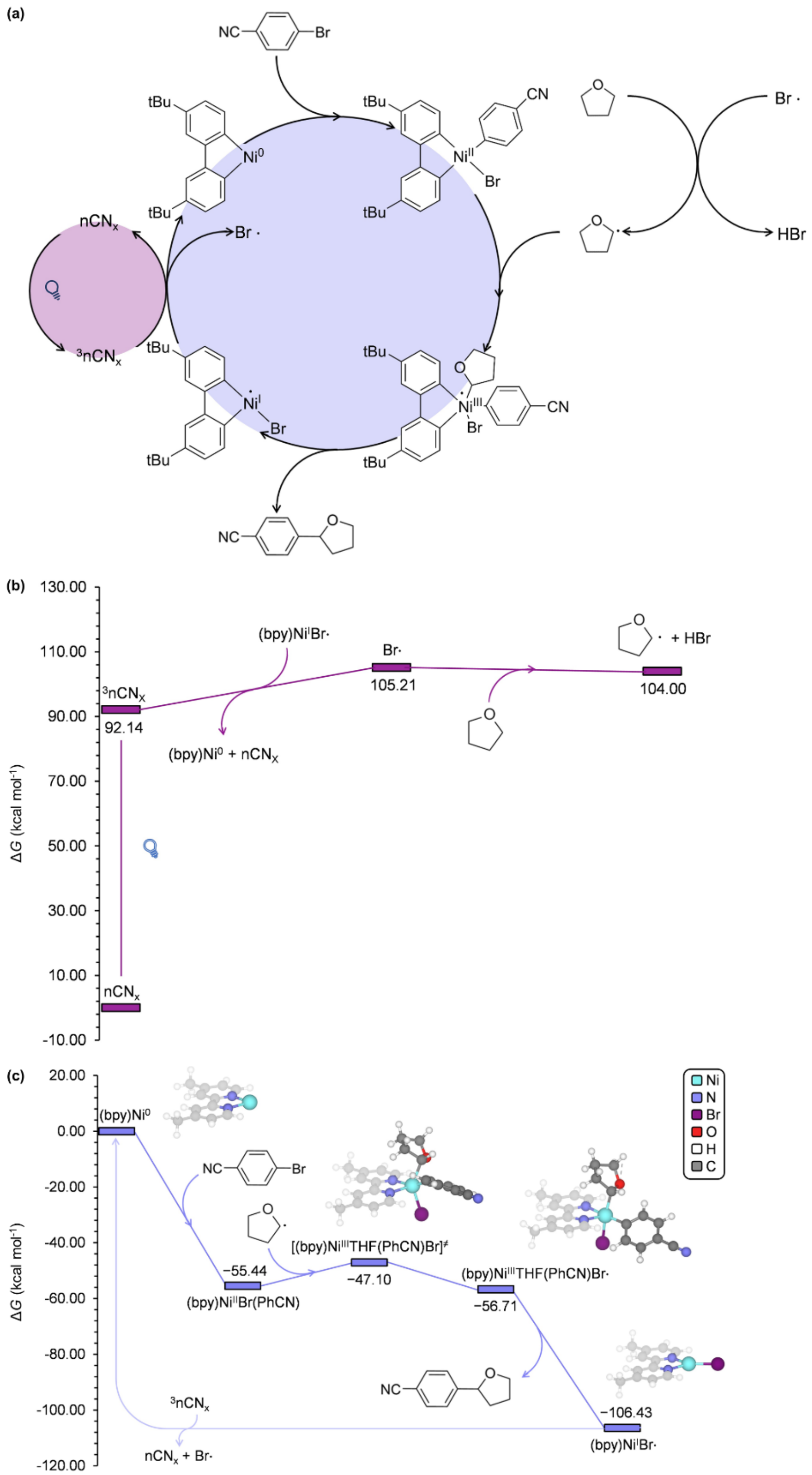

Figure S24. *(**a**) Energy transfer mechanism M2 for the C(sp^3)–H functionalization. The colour coding of the dual cycle refers to the corresponding energy diagrams in panels b and c. (**b**) Corresponding free energy diagrams for nCN$_x$ photoexcitation and (**c**) C(sp^3)–H functionalization.*

The reaction follows a synergic dual catalytic cycle, where the metal free carbon nitride, after light irradiation, promotes energy transfer to the (bpy)Ni^{I}Br• species, which upon excitation decomposes to form the Br• radical. The Br• radical successively performs a H-abstraction reaction from THF to form the THF•. As shown in the Gibbs free energy diagram in panel a, the excitation energy of nCN_x to the ground triplet electronic state, which is endothermic by about 92 kcal mol^{-1}, does not provide energy sufficient to release Br from the (bpy)Ni^{I}Br• complex, which is endothermic by 13.1 kcal mol^{-1}, thus indicating that nCN_x should be photoexcited to an energy state that is higher than the ground triplet state for this mechanism to be effective, as also suggested in the literature for a similar system.[2] Once formed THF• reacts with the Ni catalyst following a reaction mechanism that is similar to mechanism M1 proposed in the main text.

Though the reaction mechanism does not evidence any explicit role for the base, it has been proposed[1] that the HBr acid formed as a result of the progress of the overall reaction accumulates in solution until it reaches a concentration sufficient to react with the THF radical, forming back THF and thus inhibiting the further progress of the reaction. The contribution of the base, Na_2CO_3 for the present system, to the reaction mechanism would then be that of reacting with HBr to form NaBr and $NaHCO_3$, which are the same products of the M1 mechanisms. Though quite reasonable, this mechanism is not consistent with the experimental evidence that the reaction does not proceed in the absence of the base.

Mechanism M3. The mechanism was formulated based on a literature reaction mechanism recently proposed by Doyle *et al*.[3] to describe the reactivity of the same system studied by Molander *et al.*,[1] in which the photoexcitation is performed using an Ir complex. It is composed of the following reaction steps:

$nCN_x \rightarrow {}^{3}nCN_x$ [R16]

${}^{3}nCN_x$ + (bpy)$Ni^{II}Br_2$ → (bpy)Ni^{I}Br• + Br• + nCN_x [R17]

THF + Br• → THF• + HBr [R18]

(bpy)Ni^{I}Br• + Br-PhCN → (bpy)Ni^{III}Br(PhCN)Br [R19]

(bpy)Ni^{III}Br(PhCN)Br + (bpy)Ni^{I}Br• → (bpy)$Ni^{II}Br_2$ + (bpy)Ni^{II}Br(PhCN) [R20]

(bpy)Ni^{II}Br(PhCN) + THF• → (bpy)Ni^{III}Br(PhCN)THF• [R21]

(bpy)Ni^{III}THF(PhCN)Br• → (bpy)Ni^{I}Br• + THF-PhCN [R22]

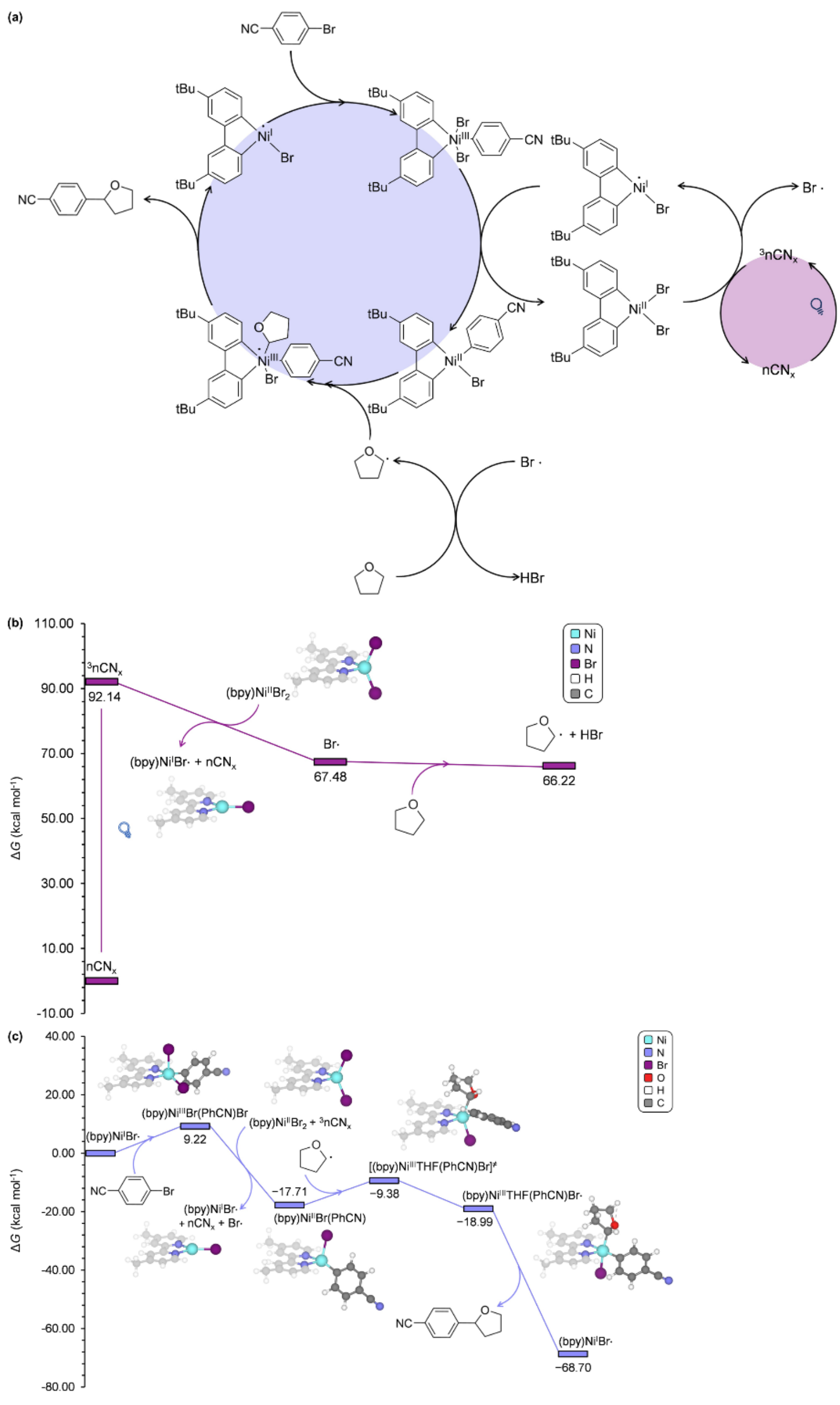

Figure S25. *(**a**) Energy transfer mechanism M3 for the C(sp^3)–H functionalization. The colour coding of the dual cycle is referring to the corresponding energy diagrams in panels b and c. (**b**) Corresponding free energy diagrams for nCN$_x$ photoexcitation and (**c**) C(sp^3)–H functionalization.*

Also, in this case the reaction follows a synergic dual catalytic cycle, where the metal free carbon nitride, after light irradiation, can activate the THF reagent with the contribution of a radical bromine. There is however a considerable difference with respect to M2, as in this mechanism the energy transfer takes place between the excited nCN_x and the $(bpy)Ni^{II}Br_2$ dibrominated chemical species, rather than the less reactive $(bpy)Ni^{I}Br•$ species. As a consequence, the energy transferred from $^3nCN_x$ to $(bpy)Ni^{II}Br_2$ is sufficient to support the release of the Br•, making this process significantly exothermic. Following the formation of Br•, the reaction mechanism proceeds similarly to mechanism M1 proposed in the main text, with the main difference of reaction R20, where $(bpy)Ni^{III}Br(PhCN)Br$ is converted to $(bpy)Ni^{II}Br(PhCN)$ while contextually $(bpy)Ni^{I}Br•$ is converted to $(bpy)Ni^{II}Br_2$. Also, in this case the contribution of the base can be interpreted as in M2 mechanism and the same considerations apply.

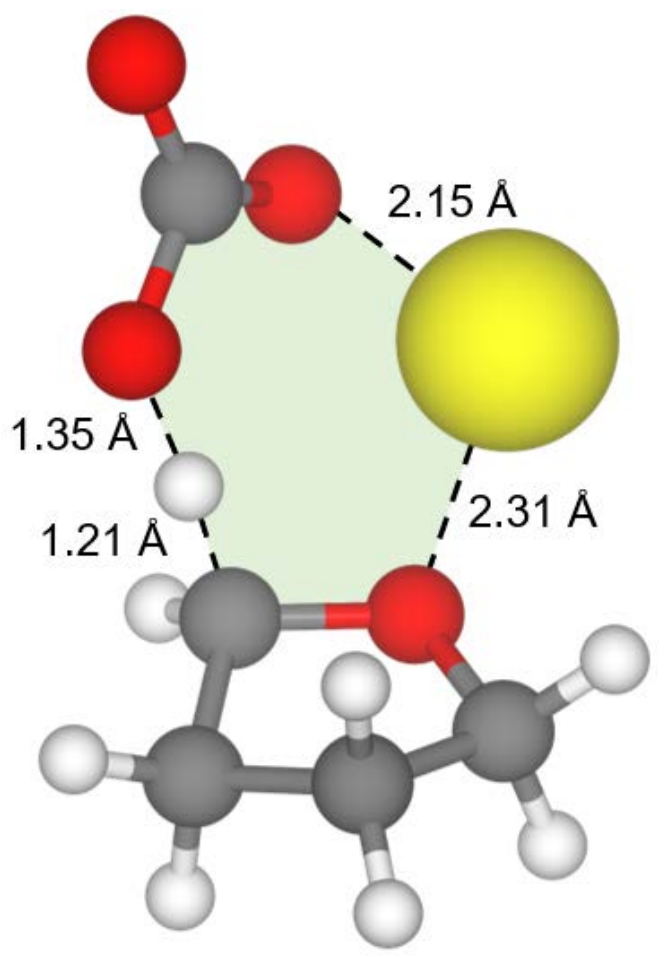

Figure S26. *Proposed interaction model between CO_3^{2-}, Na^+ and THF, highlighting the coordination of Na^+ cation between the carbonate oxygen atoms and the THF oxygen atom. White, grey, red and yellow balls correspond to hydrogen, carbon, oxygen and sodium atoms.*

Structures and vibrational frequencies

4-bromobenzonitrile (Br-PhCN)

- *XYZ Coordinates of Optimized Structures*

H 0.00000 0.00000 0.00000

C 0.00000 0.00000 1.08243

C 1.19240 0.00000 1.79083

Br 2.82455 0.00000 0.84191

C 1.21959 -0.00000 3.17731

H 2.16043 -0.00000 3.71227

C 0.02426 -0.00000 3.87181

H 0.02519 -0.00000 4.95482

C -1.18340 -0.00000 3.17384

C -2.41433 -0.00000 3.89108

C -1.19419 -0.00000 1.77893

H -2.13557 -0.00000 1.24362

N -3.40961 -0.00000 4.47112

- *Frequencies (cm^{-1})*

77.276	138.28	226.64	266.98	279.29	409.50	450.54	555.35	574.11	577.00
653.34	737.27	796.21	849.80	857.04	990.83	993.07	1032.5	1101.5	1133.4
1196.6	1236.2	1324.4	1347.3	1436.7	1523.5	1624.5	1652.4	2358.5	3219.1
3220.2	3232.2	3233.2							

CO_3^{2-}

- *XYZ Coordinates of Optimized Structures*

C 0.14535 -0.26310 0.17428

O -0.09199 0.16437 1.41089

O 1.26189 0.09611 -0.44994

O -0.73379 -1.04965 -0.43790

HCO_3^-

- *XYZ Coordinates of Optimized Structures*

C 0.27924 -0.16443 0.31265

O -0.28056 0.36540 1.31519

O 1.31182 0.17435 -0.36284

O -0.36605 -1.37916 -0.15989

H 0.15708 -1.69266 -0.92600

nCN_x

- *XYZ Coordinates of Optimized Structures*

N 0.00000 -0.00002 0.01342

C -0.00002 -0.00073 1.34341

H 0.87234 0.00043 -0.48723

H -0.87232 -0.00051 -0.48726

N -1.19682 -0.00175 1.94558

N 1.19676 -0.00048 1.94562

C -1.20863 -0.00260 3.26324

C 1.20852 -0.00133 3.26329

N -2.34404 -0.00358 3.93217

N -0.00007 -0.00238 3.96116

N 2.34390 -0.00108 3.93227

C -2.26715 -0.00442 5.26971

C -0.00010 -0.00335 5.35659

C 2.26696 -0.00193 5.26980

N -1.14724 -0.00447 6.00512

N -3.41877 -0.00513 5.93510

N 1.14702 -0.00307 6.00516

N 3.41856 -0.00174 5.93523

H -4.28933 -0.00661 5.43138

H -3.41580 -0.00731 6.94069

H 4.28913 -0.00119 5.43155

H 3.41556 -0.00272 6.94082

- *Frequencies (cm^{-1})*

95.328	97.505	112.63	227.65	248.58	251.82	264.94	290.80	291.05	298.62
301.72	312.95	393.57	454.44	455.18	543.62	586.62	587.24	587.50	587.61
593.54	657.82	743.38	744.03	760.80	766.39	792.93	793.80	800.88	801.28
855.47	1016.8	1017.6	1065.6	1076.9	1078.6	1195.4	1216.4	1216.9	1331.1
1353.2	1353.8	1438.4	1504.1	1504.2	1541.2	1578.2	1578.2	1581.5	1616.3
1617.6	1672.7	1700.6	1700.6	3608.2	3608.5	3610.3	3739.3	3739.4	3740.0

^{2} nCN$_x$•$^-$

- *XYZ Coordinates of Optimized Structures*

N -0.00195 0.15574 0.00184

C -0.00068 0.05658 1.34926

H 0.86531 0.02180 -0.48827

H -0.86606 0.00263 -0.48821

N -1.19929 0.01127 1.93706

N 1.19860 0.03774 1.93669

C -1.21571 -0.06869 3.26541

C 1.21745 -0.03978 3.26517

N -2.34547 -0.11956 3.92247

N 0.00116 -0.09937 3.94791

N 2.34785 -0.06195 3.92214

C	-2.26711	-0.19202	5.28706
C	0.00226	-0.16412	5.38903
C	2.27156	-0.13060	5.28709
N	-1.16987	-0.20520	6.01289
N	-3.48493	-0.31323	5.92996
N	1.17468	-0.16848	6.01288
N	3.49211	-0.21995	5.93057
H	-4.25656	0.09102	5.42035
H	-3.46433	-0.05120	6.90401
H	4.25344	0.20373	5.42122
H	3.46422	0.04259	6.90430

- *Frequencies (cm^{-1})*

89.121	103.27	109.71	149.14	229.76	249.45	254.52	263.96	269.32	272.36
343.58	365.11	372.20	407.90	411.76	448.95	460.24	528.68	528.91	584.21
585.82	607.78	614.34	652.79	678.57	732.47	735.39	749.59	750.72	783.06
801.00	971.61	977.28	1049.4	1074.1	1093.0	1111.6	1155.3	1172.1	1233.9
1237.5	1343.1	1348.8	1448.6	1457.7	1476.4	1502.3	1533.6	1565.8	1587.4
1602.5	1610.8	1615.6	1665.1	3567.0	3567.5	3618.4	3678.7	3678.8	3746.8

^{3} nCN_x

- *XYZ Coordinates of Optimized Structures*

N	-0.00020	-0.00405	0.01446
C	-0.00004	-0.00147	1.34449
H	0.87188	-0.00077	-0.48675
H	-0.87189	-0.03293	-0.48658
N	-1.19400	-0.03572	1.95695
N	1.19459	0.00758	1.95649

C -1.18839 0.04048 3.27544

C 1.18671 0.08347 3.27503

N -2.33314 -0.02981 3.94988

N -0.00286 0.18055 3.94677

N 2.33354 0.05420 3.94894

C -2.25016 -0.09270 5.29467

C -0.00823 0.47261 5.34795

C 2.25361 -0.00855 5.29394

N -1.15641 0.12025 6.02714

N -3.38210 -0.36538 5.95277

N 1.15346 0.16735 6.02656

N 3.39483 -0.23895 5.95207

H -4.23801 -0.50173 5.44308

H -3.38988 -0.35715 6.95806

H 4.25399 -0.35297 5.44237

H 3.40136 -0.23556 6.95739

- *Frequencies (cm^{-1})*

78.160	79.783	97.434	213.84	217.68	224.54	236.18	252.79	256.07	275.52
285.48	299.06	383.69	437.72	440.91	494.88	544.53	545.99	566.15	577.56
581.25	621.99	638.16	679.95	702.84	730.23	743.82	758.69	779.29	797.31
813.32	949.66	990.25	1009.8	1037.1	1068.9	1159.5	1194.4	1201.6	1249.5
1264.5	1326.1	1354.6	1407.3	1434.6	1506.8	1507.1	1518.8	1530.4	1555.5
1599.1	1623.5	1644.0	1655.3	3608.5	3611.6	3612.8	3739.1	3744.8	3744.9

Na(nCN$_x$)

- *XYZ Coordinates of Optimized Structures*

N	-0.00339	0.03522	0.00770
C	-0.00157	0.00707	1.34779
H	0.87048	0.03893	-0.48851
H	-0.86983	0.05574	-0.49940
N	-1.20741	0.00881	1.94642
N	1.18425	-0.01924	1.94817
C	-1.20705	-0.02261	3.28099
C	1.20371	-0.04343	3.28139
N	-2.36225	-0.02426	3.91156
N	-0.02022	-0.04727	3.96272
N	2.32094	-0.06460	3.94744
C	-2.30836	-0.06290	5.28900
C	-0.03413	-0.07293	5.40660
C	2.23594	-0.09491	5.31744
N	-1.21734	-0.09235	6.01322
N	-3.53403	0.00251	5.91399
N	1.13448	-0.10734	6.03478
N	3.44792	-0.05155	5.96527
H	-4.29447	-0.41429	5.39743
H	-3.51455	-0.26850	6.88586
H	4.22695	-0.40019	5.42856
H	3.43080	-0.35601	6.92617
Na	-3.55004	0.12889	1.93520

- *Frequencies (cm^{-1})*

-52.015	47.525	100.45	105.84	113.73	133.43	183.89	225.74	238.30	253.20	260.06
273.66	276.12	329.06	384.14	401.17	407.22	422.97	452.18	476.95	529.89	545.59
586.99	591.86	594.76	616.35	656.36	670.57	735.80	741.67	751.89	759.77	796.06

805.10 979.92 986.25 1050.0 1085.9 1106.0 1120.5 1175.4 1198.5 1234.7 1239.3 1353.2 1380.5 1450.9 1464.0 1498.8 1505.7 1539.3 1561.0 1587.9 1605.9 1618.3 1621.0 1672.1 3567.0 3581.7 3621.1 3677.0 3695.8 3752.4

(bpy)Ni^0

- *XYZ Coordinates of Optimized Structures*

N	-0.01851	0.05256	-0.00862
C	-0.03139	0.06868	1.32763
H	0.94236	0.09549	1.80792
C	-1.17706	0.05370	2.08401
H	-1.11597	0.06812	3.16546
C	-2.42984	0.01968	1.42002
C	-2.42867	0.00226	0.05018
H	-3.37502	-0.02331	-0.47698
C	-1.22039	0.01808	-0.68747
H	1.25899	0.03196	-4.37253
C	0.24131	0.01234	-3.99359
N	0.11826	0.02488	-2.66277
C	-1.14696	0.00192	-2.10972
C	-2.27335	-0.03548	-2.96623
H	-3.26807	-0.05516	-2.53727
C	-2.13519	-0.04810	-4.32909
C	-0.82125	-0.02277	-4.86234
H	-0.65004	-0.03093	-5.93197
Ni	1.44047	0.07162	-1.26480
C	-3.69704	0.00363	2.20934
H	-3.73522	-0.86879	2.86995
H	-4.57335	-0.02032	1.55940

H -3.76910 0.88809 2.85083

C -3.31585 -0.08738 -5.24215

H -4.25317 -0.10199 -4.68342

H -3.28485 -0.97386 -5.88413

H -3.32563 0.78287 -5.90662

- *Frequencies* (cm^{-1})

61.522	74.335	87.236	123.51	132.78	137.21	198.30	232.70	234.51	245.58
259.08	289.22	351.74	381.46	417.23	433.45	477.32	488.16	559.05	566.03
599.21	672.59	706.00	747.16	750.89	765.27	770.24	844.04	857.50	871.63
924.60	963.69	964.71	997.28	1007.0	1019.1	1043.8	1054.0	1055.2	1078.1
1134.4	1146.4	1216.7	1239.6	1248.0	1310.2	1341.7	1344.3	1363.4	1397.4
1399.4	1424.1	1451.6	1456.9	1457.5	1466.7	1482.5	1517.0	1532.8	1539.5
1566.7	1636.1	1647.1	3036.8	3037.6	3093.2	3093.3	3134.8	3135.1	3174.1
3174.9	3201.8	3212.0	3212.4	3213.6					

(bpy)Ni^{I}Br•

- *XYZ Coordinates of Optimized Structures*

C 0.01185 -0.00049 -0.00785

C -0.00577 -0.00307 1.37172

N 1.10570 -0.00324 2.10731

C 2.29262 -0.00083 1.47979

C 2.38469 0.00182 0.09611

C 1.23228 0.00130 -0.67921

Ni 1.21633 -0.00153 4.07869

Br -0.19606 -0.00068 5.90643

C 3.45899 0.00110 2.38098

N 3.15179 0.00125 3.68770

C	4.14460	0.00309	4.57723
C	5.47475	0.00477	4.21150
C	5.81602	0.00444	2.86085
C	4.77461	0.00252	1.94218
C	7.24195	0.00618	2.42373
C	1.29614	-0.00896	-2.16922
H	3.84999	0.00325	5.62051
H	6.24109	0.00627	4.97787
H	4.99784	0.00208	0.88314
H	-0.94064	-0.00343	1.92059
H	3.35260	0.00622	-0.38862
H	-0.92307	0.00189	-0.55580
H	7.76097	0.88785	2.80973
H	7.32625	0.00303	1.33671
H	7.76493	-0.87065	2.81528
H	2.31236	0.16040	-2.52626
H	0.64223	0.75744	-2.59204
H	0.95386	-0.97322	-2.55707

- *Frequencies (cm^{-1})*

19.804	35.286	38.908	49.798	58.806	87.121	121.24	127.58	176.16	198.52
226.61	247.94	254.90	286.93	306.09	351.45	384.89	437.22	459.30	485.35
532.03	556.94	565.84	631.38	683.88	761.66	763.12	794.73	851.99	860.28
864.50	918.66	927.03	951.74	1004.8	1008.7	1009.6	1021.5	1041.5	1049.7
1060.3	1060.5	1104.3	1141.2	1156.5	1223.3	1255.7	1307.8	1322.2	1325.3
1342.3	1348.5	1398.6	1400.3	1442.2	1443.5	1458.1	1461.0	1473.7	1486.9
1530.2	1532.8	1615.0	1625.3	1673.1	1681.3	3054.2	3055.3	3118.8	3119.0
3149.9	3150.4	3203.1	3203.4	3217.9	3218.3	3222.5	3232.3		

(bpy)$Ni^{II}Br_2$

- *XYZ Coordinates of Optimized Structures*

C 0.03051 0.03859 0.01198

C 0.03012 0.03310 1.39081

N 1.15498 0.01710 2.09880

C 2.33662 0.00700 1.46698

C 2.40810 0.01026 0.08309

C 1.24288 0.02507 -0.67349

Ni 1.29545 -0.00801 4.08977

N 3.24184 -0.01186 3.66220

C 4.23686 -0.02157 4.54390

C 5.55995 -0.02617 4.15714

C 5.87799 -0.02051 2.80088

C 4.82520 -0.00968 1.89467

C 3.51655 -0.00558 2.35122

C 7.29646 -0.02601 2.34367

C 1.28616 0.01384 -2.16322

H 3.95262 -0.02567 5.58972

H 6.33823 -0.03411 4.91075

H 5.03457 -0.00454 0.83334

H -0.89423 0.04240 1.95653

H 3.36778 0.00248 -0.41672

H -0.91077 0.05394 -0.52391

H 7.82707 0.84600 2.73537

H 7.36599 -0.01676 1.25596

H 7.81473 -0.91241 2.71940

H 2.29778 0.17776 -2.53471

H 0.62802 0.78169 -2.57616

H 0.93364 -0.95022 -2.54215

Br 0.76161 2.17945 4.85971

Br 0.75019 -2.22653 4.76166

Frequencies (cm^{-1})

25.157	33.791	49.124	49.970	57.567	63.276	77.775	84.184	113.69	131.11
171.70	227.29	228.54	250.37	265.99	274.10	281.74	289.85	350.33	384.62
434.96	463.07	486.04	534.92	571.62	574.64	628.76	683.91	762.36	762.88
792.85	853.91	862.33	865.03	920.13	927.25	954.75	1008.8	1010.7	1012.9
1022.7	1055.1	1060.6	1061.0	1063.8	1109.5	1146.8	1161.1	1221.0	1256.2
1310.6	1326.3	1330.3	1346.5	1357.4	1398.0	1400.1	1441.6	1444.9	1457.7
1461.2	1475.7	1488.7	1540.2	1543.7	1623.4	1632.4	1675.3	1688.2	3056.6
3057.6	3121.9	3122.0	3154.5	3154.9	3210.3	3210.7	3224.3	3224.5	3227.3
3238.1									

(bpy)Ni^{II}Br(PhCN)

- *XYZ Coordinates of Optimized Structures*

N 0.00400 -0.04415 -0.00838

C -0.00496 -0.06379 1.32707

H 0.95948 -0.08096 1.81420

C -1.16594 -0.06065 2.07029

H -1.09987 -0.07472 3.15158

C -2.39931 -0.03774 1.42868

C -2.38389 -0.01600 0.04065

H -3.32000 0.00413 -0.50169

C -1.18287 -0.01958 -0.64910

H 1.37538 -0.01063 -4.23938

C 0.34384 0.00352 -3.90864

N	0.16289	-0.01177	-2.58952
C	-1.09212	0.00321	-2.11386
C	-2.19220	0.03722	-2.95604
H	-3.19436	0.05128	-2.54873
C	-2.01430	0.05515	-4.33314
C	-0.70498	0.03573	-4.80412
H	-0.49659	0.04630	-5.86746
Ni	1.57174	-0.04559	-1.15663
C	-3.67654	-0.05147	2.19550
H	-3.84043	-1.04082	2.63372
H	-4.52867	0.18346	1.55733
H	-3.64238	0.66376	3.02029
C	-3.17379	0.09772	-5.26864
H	-4.12248	0.06422	-4.73255
H	-3.13569	-0.74350	-5.96570
H	-3.14594	1.01193	-5.86811
Br	3.40959	-0.04546	-2.58115
C	2.78307	-0.07450	0.27782
C	3.21712	-1.28242	0.83604
C	4.05258	-1.31492	1.93820
C	4.48326	-0.11704	2.51387
C	4.07245	1.10211	1.96910
C	3.23658	1.11149	0.86683
H	2.89803	-2.22530	0.40314
H	4.37782	-2.25987	2.35879
C	5.33734	-0.13855	3.64975
H	4.41319	2.03045	2.41371
H	2.93285	2.07027	0.45845

N 6.02807 -0.15582 4.57380

- *Frequencies (cm^{-1})*

10.308	29.325	29.438	42.685	50.046	62.369	69.284	83.557	106.88	108.39
115.53	131.40	160.14	164.15	182.87	205.69	226.27	241.17	277.94	291.25
304.17	305.33	327.53	361.19	392.87	393.37	438.30	460.64	479.68	503.89
534.68	535.00	557.30	564.44	580.88	582.62	630.42	652.91	687.49	752.47
762.60	764.69	789.93	790.94	841.86	852.40	855.00	865.07	865.30	909.29
918.50	956.97	986.27	988.45	1014.2	1014.6	1024.4	1025.1	1034.8	1042.5
1049.0	1057.8	1059.8	1087.5	1110.5	1124.9	1147.9	1167.1	1192.2	1230.9
1240.3	1259.4	1311.0	1312.2	1322.2	1330.7	1333.5	1348.3	1353.2	1397.3
1401.8	1426.1	1442.8	1446.4	1457.7	1461.0	1476.8	1491.0	1514.5	1537.7
1544.6	1590.9	1618.6	1627.6	1634.5	1678.5	1688.1	2340.2	3056.3	3057.1
3121.6	3122.0	3152.3	3154.9	3178.3	3178.6	3205.5	3206.5	3207.1	3215.7
3218.8	3230.9	3237.4	3245.8						

(bpy)Ni^{III}Br(PhCN)Br

- *XYZ Coordinates of Optimized Structures*

C -0.01005 -0.03083 0.00663

C 0.00051 0.00138 1.39638

C 1.19531 0.03161 2.09567

C 2.39319 0.06869 1.39979

C 2.38861 0.06276 0.00468

C 1.18164 0.00856 -0.69480

Ni -1.73707 0.14071 2.18568

Br -1.63568 -2.20232 2.15997

C 3.61833 0.10831 -0.71068

N 4.61310 0.14855 -1.29173

N	-2.01298	2.00313	1.56208
C	-1.07322	2.84066	1.12652
C	-1.34505	4.14810	0.78872
C	-2.64434	4.63425	0.90038
C	-3.61096	3.75488	1.36914
C	-3.27250	2.45223	1.69801
C	-2.97503	6.04174	0.54225
C	-4.22615	1.48648	2.25903
N	-3.69432	0.30376	2.59680
C	-4.46738	-0.61872	3.16123
C	-5.80755	-0.40759	3.40646
C	-6.39051	0.80578	3.05124
C	-5.56856	1.76058	2.46651
C	-7.83576	1.06892	3.30106
H	-0.06873	2.44736	1.05365
H	-0.53895	4.78444	0.44405
H	-4.63035	4.09626	1.49133
H	-3.98313	-1.55328	3.41643
H	-5.98181	2.71850	2.17970
H	-6.39339	-1.19046	3.87298
H	-2.35433	6.73630	1.11430
H	-4.02353	6.26912	0.73435
H	-2.76682	6.22309	-0.51592
H	-8.13829	2.04361	2.91816
H	-8.04610	1.03685	4.37381
H	-8.45290	0.29928	2.83034
H	1.19765	0.03815	3.17746
H	3.33392	0.10117	1.93679

H 1.18161 -0.00721 -1.77828

H -0.94375 -0.07973 -0.54361

Br -1.10698 0.86206 4.46725

- *Frequencies (cm^{-1})*

24.974	28.567	30.862	49.695	60.793	64.415	67.134	74.400	85.091	94.872
104.72	110.31	117.97	135.73	139.51	160.70	178.88	192.34	225.34	232.58
244.35	281.39	293.02	299.12	313.77	325.70	359.01	398.68	401.04	436.92
460.28	472.09	498.41	520.29	536.11	569.10	570.67	577.35	578.20	629.75
646.55	685.45	737.37	759.51	765.27	777.62	803.64	839.36	851.51	853.30
863.89	866.44	916.39	936.00	962.58	986.54	996.62	1010.5	1016.0	1017.6
1018.9	1027.8	1051.2	1058.6	1059.4	1060.8	1069.6	1113.1	1128.7	1150.7
1169.0	1191.9	1234.8	1243.4	1258.8	1317.5	1318.3	1327.3	1331.3	1342.7
1353.9	1358.1	1398.5	1401.5	1418.3	1444.9	1447.0	1458.4	1458.8	1479.3
1492.4	1511.2	1540.1	1549.7	1607.6	1622.7	1629.3	1630.7	1677.9	1687.8
2350.5	3057.3	3057.4	3121.9	3122.0	3153.6	3154.7	3191.7	3210.0	3212.6
3216.2	3216.3	3224.4	3225.4	3226.6	3235.9	3242.0			

(bpy)Ni^{III}Br(PhCN)THF•

- *XYZ Coordinates of Optimized Structures*

N -0.27528 0.18915 0.13975

C -0.46513 0.21747 1.47168

H 0.42166 0.35896 2.07726

C -1.73070 0.07519 2.04365

H -1.83667 0.09962 3.12309

C -2.85415 -0.10279 1.21530

C -2.64163 -0.10016 -0.17244

H -3.48613 -0.21323 -0.84314

C	-1.34768	0.05096	-0.68407
H	1.76806	0.57509	-3.80660
C	0.70856	0.39613	-3.66936
N	0.28379	0.33382	-2.39338
C	-1.02428	0.10386	-2.12263
C	-1.95266	-0.06842	-3.15554
H	-2.99541	-0.26182	-2.92972
C	-1.53303	-0.01201	-4.49372
C	-0.16868	0.23373	-4.74019
H	0.21002	0.28419	-5.75550
Ni	1.51380	0.28655	-0.85154
C	-4.22684	-0.30315	1.79569
H	-4.36875	-1.35276	2.08349
H	-5.01098	-0.04823	1.07646
H	-4.37311	0.30452	2.69442
C	-2.49773	-0.23998	-5.62460
H	-3.53232	-0.06226	-5.31609
H	-2.42977	-1.27668	-5.97845
H	-2.27573	0.41077	-6.47660
Br	1.71162	-2.11360	-1.31301
C	2.91522	0.20239	0.46618
C	4.13439	0.89854	0.31476
C	5.17589	0.75479	1.23703
C	5.01510	-0.08123	2.35929
C	3.81024	-0.79508	2.51929
C	2.78834	-0.66220	1.57504
H	4.28028	1.55105	-0.54234
H	6.11185	1.28794	1.09589

C 6.06734 -0.21298 3.32245

H 3.69289 -1.46630 3.36538

H 1.90729 -1.28957 1.67195

N 6.93199 -0.31740 4.11996

C 1.73554 2.24000 -0.86469

C 0.48029 3.11320 -1.06067

C 0.57071 3.63733 -2.51140

C 2.06943 3.55317 -2.81974

O 2.54792 2.38858 -2.07449

H -0.45116 2.56861 -0.88806

H 0.50008 3.94744 -0.34735

H 0.18701 4.65740 -2.61148

H 0.00511 2.99310 -3.19133

H 2.60139 4.44803 -2.46616

H 2.30346 3.38775 -3.87459

H 2.32112 2.55293 0.00280

[(bpy)Ni^{III}Br(PhCN)THF]$^{\neq}$

- *XYZ Coordinates of Optimized Structures*

N -0.00061 -0.06442 0.02221

C -0.02467 -0.04116 1.35252

H 0.93771 -0.06237 1.84840

C -1.19368 0.00171 2.08280

H -1.14822 0.01772 3.16537

C -2.41619 0.02136 1.41743

C -2.38263 -0.04233 0.03154

H -3.31268 -0.06522 -0.52103

C -1.16769 -0.09896 -0.63821

H	1.38010	-0.85503	-4.17124
C	0.35511	-0.70685	-3.85305
N	0.17708	-0.44353	-2.56231
C	-1.06573	-0.27652	-2.09972
C	-2.16664	-0.32290	-2.94544
H	-3.16571	-0.17113	-2.55800
C	-1.99268	-0.57417	-4.29970
C	-0.69084	-0.78238	-4.74931
H	-0.49124	-0.99801	-5.79243
Ni	1.67617	-0.21785	-1.16440
C	-3.70337	0.08730	2.16696
H	-3.78516	-0.75215	2.86256
H	-4.56049	0.06622	1.49365
H	-3.75173	1.00300	2.76278
C	-3.15366	-0.64669	-5.23319
H	-4.06464	-0.26775	-4.76883
H	-3.33101	-1.68386	-5.53371
H	-2.95781	-0.07688	-6.14433
Br	2.17642	-2.63895	-1.00665
C	3.09314	0.58962	-0.15277
C	4.37284	0.01492	-0.27098
C	5.30096	0.13196	0.73729
C	4.99600	0.86134	1.89547
C	3.74874	1.48182	2.00738
C	2.82454	1.35944	0.99100
H	4.62664	-0.55435	-1.15698
H	6.26987	-0.34509	0.64440
C	5.94891	0.98308	2.93812

H 3.51482 2.06308 2.89211

H 1.87909 1.88177 1.07993

N 6.72394 1.08505 3.78804

C 2.52018 1.60911 -1.73449

C 1.31221 2.55112 -1.87858

C 1.10326 2.61736 -3.39825

C 2.37879 2.01254 -3.96396

O 2.81364 1.05360 -3.00297

H 0.41690 2.20655 -1.36378

H 1.56897 3.52829 -1.46553

H 0.95304 3.63823 -3.75099

H 0.23679 2.02650 -3.69526

H 3.15708 2.77815 -4.08243

H 2.24444 1.49771 -4.91624

H 3.37703 2.20990 -1.43046

- *Frequencies (cm^{-1})*

19.253	24.013	27.116	39.911	46.164	51.307	60.775	73.655	77.043	86.476
104.95	109.88	113.88	125.43	134.97	143.49	144.69	160.89	173.64	189.33
214.79	218.33	242.88	248.56	268.01	276.91	279.89	287.39	290.44	346.58
386.58	391.22	419.52	444.09	467.87	489.71	508.74	525.58	535.74	556.72
559.23	576.36	581.22	620.35	631.01	651.11	686.18	713.53	729.76	760.33
768.33	780.26	795.74	824.50	836.87	848.89	855.70	861.23	867.41	897.30
917.37	919.06	922.30	941.74	954.34	983.23	986.13	989.69	1010.9	1013.9
1021.2	1025.1	1026.0	1039.1	1043.6	1048.8	1055.7	1059.6	1060.8	1077.8
1080.8	1111.7	1134.0	1143.3	1160.6	1187.7	1193.2	1213.9	1234.1	1247.0
1257.1	1259.5	1291.7	1304.4	1312.5	1313.4	1325.6	1327.2	1328.8	1332.9
1347.5	1351.6	1357.7	1384.0	1399.4	1401.2	1442.1	1443.0	1447.6	1459.9

1460.9	1474.9	1477.8	1486.4	1494.7	1504.7	1507.8	1538.4	1539.3	1578.8
1620.3	1631.8	1632.5	1674.1	1684.3	2329.3	3020.4	3055.1	3056.5	3079.2
3090.9	3096.7	3120.6	3120.9	3123.9	3134.9	3149.9	3150.3	3152.5	3193.9
3200.5	3206.9	3208.6	3211.2	3215.2	3221.0	3222.8	3226.3	3233.9	

THF

- *XYZ Coordinates of Optimized Structures*

O	0.02953	-0.00251	0.02106
C	0.01974	-0.00095	1.44614
C	1.37508	0.00098	-0.44863
C	1.43775	0.32004	1.88164
H	-0.71449	0.73185	1.79306
H	-0.28747	-0.99124	1.80867
C	2.24746	-0.30729	0.75452
H	1.47080	-0.73636	-1.25090
H	1.61271	0.99012	-0.86278
H	1.67491	-0.08247	2.86759
H	1.59439	1.40251	1.90369
H	2.32692	-1.38843	0.90137
H	3.25423	0.10200	0.65827

- *Frequencies (cm^{-1})*

73.508	260.61	577.29	676.35	852.40	887.99	920.04	930.82	950.45	973.62
1045.2	1113.6	1166.8	1188.6	1196.4	1261.8	1262.7	1314.9	1338.3	1369.3
1403.4	1466.9	1477.9	1510.0	1520.8	3007.7	3014.3	3060.3	3064.3	3082.2
3084.6	3125.6	3132.4							

THF•

- *XYZ Coordinates of Optimized Structures*

O	0.01949	-0.04124	0.02080
C	0.01727	-0.00889	1.45272
C	1.31803	0.00639	-0.39205
C	1.31509	-0.68173	1.85724
H	-0.01448	1.03578	1.78543
H	-0.88293	-0.51915	1.79634
C	2.26425	-0.24648	0.73387
H	1.46864	-0.26230	-1.43035
H	1.19028	-1.76732	1.84848
H	1.64883	-0.37890	2.85014
H	3.01352	-1.00628	0.49421
H	2.81143	0.66185	1.01854

- *Frequencies (cm^{-1})*

169.55	249.76	499.86	603.36	735.21	863.77	883.73	945.67	955.93	977.25
1037.3	1076.3	1184.9	1203.0	1218.0	1252.8	1297.4	1307.3	1354.6	1409.7
1458.6	1475.3	1503.6	3010.2	3034.0	3070.6	3082.9	3130.5	3139.6	3214.8

THF-PhCN

- *XYZ Coordinates of Optimized Structures*

H	0.00000	0.00000	0.00000
C	0.00000	0.00000	1.08283
C	1.21406	0.00000	1.76283
C	2.51863	-0.07590	1.02494
C	1.21442	0.02587	3.15546
H	2.15751	0.04186	3.69155
C	0.02864	0.03627	3.86344

H	0.03174	0.05609	4.94651
C	-1.18382	0.03050	3.17151
C	-2.41149	0.04720	3.89234
C	-1.19557	0.01564	1.77606
H	-2.14029	0.01869	1.24587
N	-3.40541	0.05898	4.47595
O	2.40038	0.44758	-0.28982
H	3.26185	0.51684	1.57577
C	3.03565	-1.50628	0.85827
H	2.19416	-2.18708	0.69989
C	3.86956	-1.38444	-0.40657
H	3.59397	-1.84443	1.73151
C	3.02662	-0.42755	-1.22689
H	4.84306	-0.94013	-0.18162
H	4.03198	-2.33787	-0.91110
H	2.25520	-0.96602	-1.79215
H	3.60930	0.17549	-1.92752

- *Frequencies (cm^{-1})*

34.159	49.372	67.237	135.02	162.71	218.31	246.99	291.39	341.91	410.25
497.69	524.53	566.35	583.36	587.12	647.46	671.37	746.05	769.52	837.64
860.12	868.05	890.17	904.67	947.45	960.61	965.94	992.06	998.50	1039.7
1047.4	1108.9	1128.6	1141.2	1188.6	1194.2	1203.4	1236.6	1249.2	1263.2
1298.6	1322.4	1334.7	1338.0	1353.8	1385.9	1398.3	1450.8	1465.3	1477.4
1516.0	1547.4	1627.6	1673.7	2351.4	3010.9	3021.9	3066.0	3070.6	3097.3
3132.2	3140.6	3194.7	3212.3	3220.3	3226.5				

[THF⋯CO_3Na_2]

- *XYZ Coordinates of Optimized Structures*

O -0.02943200 -0.08121700 0.02979400

C 0.00208200 -0.05656900 1.46660300

C 1.31122200 -0.00751100 -0.47948500

C 1.46840400 -0.08056500 1.86280700

H -0.48617400 0.86281000 1.80913500

H -0.56026400 -0.91411900 1.84448700

C 2.13842400 0.55782500 0.65303500

H 1.64486100 -1.01495900 -0.75594900

H 1.30642400 0.62541900 -1.36928000

H 1.81459800 -1.11023500 1.98657400

H 1.64965900 0.45653500 2.79458800

H 3.19627000 0.30849300 0.56144800

H 2.03673800 1.64632300 0.68125700

O 0.78913500 4.88057700 -0.39823200

C 0.06977000 3.84680400 -0.61597300

O 0.17678800 3.13898500 -1.66998100

O -0.82233400 3.49827500 0.29511100

Na -1.24644400 1.65705800 -0.85890300

Na -0.22366900 5.21764100 1.50644700

- *Frequencies* (cm^{-1})

18.48 25.27 37.15 43.24 77.28 92.37 111.40 137.99 175.03 220.45 266.65 304.64 316.80 339.79 576.86 678.90 706.61 737.12 851.35 878.84 885.72 916.90 925.83 950.92 977.63 1051.32 1055.33 1088.02 1166.71 1191.44 1206.10 1267.21 1275.24 1318.70 1343.14 1373.52 1379.27 1408.73 1466.69 1478.60 1504.56 1515.72 1542.85 3030.46 3039.22 3067.94 3073.11 3096.79 3113.39 3131.33 3139.44

[THF⋯CO3Na]•

- *XYZ Coordinates of Optimized Structures*

O 0.37206 0.72357 -0.06870

C -0.14706 -0.14280 0.95885

C 1.80684 0.67795 -0.04923

C 1.05761 -0.79828 1.61344

H -0.70748 0.46869 1.67375

H -0.82991 -0.86135 0.49943

C 2.16642 0.21047 1.34215

H 2.15470 -0.02935 -0.81143

H 2.18604 1.67269 -0.29221

H 1.28687 -1.74977 1.12666

H 0.89265 -0.98619 2.67466

H 3.16629 -0.22205 1.39176

H 2.11120 1.04607 2.04581

O 1.24725 4.40640 2.94539

C 0.60269 3.99594 1.91268

O 1.13879 4.15951 0.78933

O -0.51410 3.45536 2.10455

Na -0.51164 2.80173 -0.13331

- *Frequencies (cm^{-1})*

29.242 35.337 42.287 52.376 79.595 93.217 131.83 160.87 225.07 251.45 272.01 278.42 578.49 589.74 682.46 837.45 849.26 887.03 915.31 927.83 951.22 975.96 1052.6 1071.2 1084.1 1165.4 1190.7 1208.7 1265.5 1274.6 1297.3 1318.9 1342.8 1376.9 1406.8 1467.5 1479.0 1505.2 1514.6 1517.9 3035.9 3049.4 3070.0 3074.5 3102.9 3113.1 3134.9 3142.3

[THF⋯CO3Na] ≠

- *XYZ Coordinates of Optimized Structures*

O	0.07811	0.21296	0.04432
C	0.01156	0.04931	1.41698
C	1.44521	0.17824	-0.38378
C	1.31639	-0.56867	1.86202
H	-0.09439	1.09280	1.93643
H	-0.90576	-0.48420	1.68445
C	2.28873	0.07013	0.87597
H	1.56354	-0.69566	-1.03476
H	1.65051	1.07873	-0.96583
H	1.27810	-1.65578	1.73164
H	1.54808	-0.35351	2.90546
H	3.19304	-0.51834	0.71720
H	2.56520	1.06741	1.22169
O	-0.22553	2.31878	2.81980
C	0.42874	3.33871	2.30714
O	0.35477	4.39512	2.99510
O	1.05747	3.22825	1.22553
Na	1.60244	5.39493	1.40170

- *Frequencies (cm^{-1})*

29.686 41.983 47.330 89.103 105.09 136.18 187.51 219.77 286.20 326.43 524.58 594.52 672.63 707.62 843.16 845.57 872.33 918.84 946.63 955.68 967.00 1004.8 1049.0 1129.8 1152.6 1189.2 1207.5 1239.5 1252.9 1309.4 1329.4 1351.4 1373.0 1384.7 1402.2 1461.2 1472.8 1500.4 1580.2 1722.5 3039.7 3049.9 3083.1 3087.2 3109.7 3132.4 3145.7

Experimental data

General procedure (GP) for C–H functionalization. In an 8 mL vial were added the corresponding aryl or alkyl halide (0.2 mmol, 1.0 equiv.), $NiCl_2$·glyme (0.02 mmol, 0.1 equiv.), dtbbpy (0.02 mmol, 0.1 equiv.), nCN_x (50 mg) and Na_2CO_3 (0.4 mmol, 2.0 equiv.). The mixture was dissolved in the corresponding C–H donor (4 mL). The vial was then closed with a screw cap rubber septum and inserted in the Uniqsis photoreactor. The suspension was vigorously stirred under purple light (120 W, 420 nm) and connected to a chiller to keep the reaction temperature at 40 °C for 48 h. After this time, the crude was filtered off through a PTFE syringe filter (0.45 µm) and purified *via* silica gel column chromatography to afford product **3.**

Note: solvents containing a stabilizer were previously distilled.

4-(tetrahydrofuran-2-yl)benzonitrile (3aa)

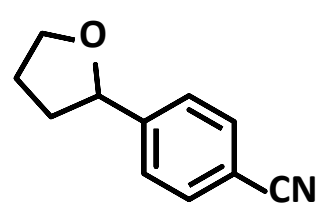

The reaction was performed according to the GP, using 4-bromobenzonitrile (0.2 mmol, 1.0 equiv.), $NiCl_2$·glyme (0.02 mmol, 0.1 equiv.), dtbbpy (0.02 mmol, 0.1 equiv.), nCN_x (50 mg), Na_2CO_3 (0.4 mmol, 2.0 equiv.) in 4 mL of THF. The mixture was purified via silica gel column chromatography (0 to 10% EtOAc/Hexane) to give product **3aa** as a colorless oil (36 mg, 74% yield). Large scale reaction was carried out in a 50 mL round bottom flask, where 4-bromobenzonitrile (2.4 mmol, 1.0 equiv.), $NiCl_2$·glyme (0.24 mmol, 0.1 equiv.), dtbbpy (0.24 mmol, 0.2 equiv.), nCN_x (500 mg), Na_2CO_3 (4.8 mmol, 2.0 equiv.), were added in 48 mL of THF. Four kessil lamps (427 nm) at 6 cm distance, and with a fan placed above for cooling reasons, irradiated the suspension, which was vigorously stirred for 48 h, obtaining **3aa** (295 mg, 71% yield).

R_f (10% EtOAc/Hexane): 0.3.

^{1}H NMR (400 MHz, $CDCl_3$) δ 7.62 (2H, d, J = 8.3 Hz), 7.43 (2H, d, J = 8.1 Hz), 4.93 (1H, t, J = 7.2 Hz), 4.10 (1H, dt, J = 8.3, 6.8 Hz), 3.96 (1H, dt, J = 8.3, 6.9 Hz), 2.38 (1H, dq, J = 13.2, 6.8 Hz), 2.05 – 1.98 (2H, m).

^{13}C NMR (101 MHz, $CDCl_3$) δ 149.4, 132.3, 126.3, 119.1, 111.0, 80.0, 69.1, 34.8, 26.1.

This data is consistent with literature precedent.[1]

4-(5-methyltetrahydrofuran-2-yl)benzonitrile (3ba)

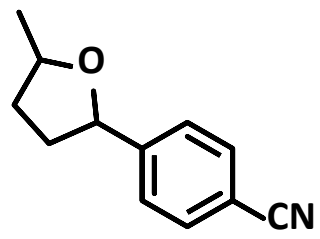

The reaction was performed according to the GP, using 4-bromobenzonitrile (0.2 mmol, 1.0 equiv.), $NiCl_2$·glyme (0.02 mmol, 0.1 equiv.), dtbbpy (0.02 mmol, 0.1 equiv.), nCN_x (50 mg), Na_2CO_3 (0.4 mmol, 2.0 equiv.) in 4 mL of 2-MeTHF. The mixture was purified via silica gel column chromatography (0 to 10% EtOAc/Hexane) to give product **3ba** as a colorless oil (13.1 mg, 35% yield).

R_f (10% EtOAc/Hexane): 0.3.

^{1}H NMR (400 MHz, $CDCl_3$) δ 7.61 (2H, dd, J = 8.4, 1.9 Hz), 7.44 (2H, dd, J = 9.9, 8.1 Hz), 5.07 (t, J = 7.4 Hz) & 4.90 (1H combined, t , J = 7.3 Hz), 4.39 – 4.31 (m) & 4.24 – 4.16 (1H combined, m), 2.48 – 2.40 (m) & 2.38 – 3.32 (1H combined, m), 2.18 – 2.06 (1H, m), 1.84 – 1.75 (1H, m), 1.69 – 1.55 (1H, m), 1.37 (d, J = 6.1 Hz) & 1.33 (3H combined, d, J = 6.1 Hz).

^{13}C NMR (101 MHz, $CDCl_3$) cis/trans mixture, minor isomer signals are enclosed in parenthesis: δ 150.0, (149.5), 132.4, (132.3), 126.5, (126.2), 127.7, (119.1), 111.0, (110.9), 80.2, (79.6), 76.6, (76.6), 35.7, (34.8), 34.2, (33.0), 21.5, (21.3).

4-(2-methyltetrahydrofuran-2-yl)benzonitrile (3ba')

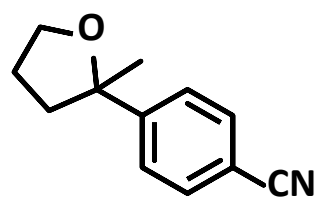

The reaction was performed according to the GP, using 4-bromobenzonitrile (0.2 mmol, 1.0 equiv.), $NiCl_2$·glyme (0.02 mmol, 0.1 equiv.), dtbbpy (0.02 mmol, 0.1 equiv.), nCN_x (50 mg), Na_2CO_3 (0.4 mmol, 2.0 equiv.) in 4 mL of 2-MeTHF. The mixture was purified via silica gel column chromatography (0 to 10% EtOAc/Hexane) to give product **3ba'** as a colorless oil mixed with **3ba** (22.5 mg, 64% pure, 38% yield).

R_f (10% EtOAc/Hexane): 0.3.

^{1}H NMR (400 MHz, $CDCl_3$) δ 7.61 (2H, d, J = 8.3 Hz), 7.50 (2H, d, J = 8.5 Hz), 4.06 – 4.01 (1H, m), 3.93 – 3.86 (1H, m), 2.08 – 1.96 (2H, m), 1.85 – 1.74 (1H, m), 1.69 – 1.60 (1H, m), 1.51 (3H, s).

^{13}C NMR (101 MHz, $CDCl_3$) δ 154.0, 132.2, 125.7, 119.2, 110.5, 84.2, 68.0, 39.7, 29.5, 25.9.

4-(1-ethoxyethyl)benzonitrile (3ca)

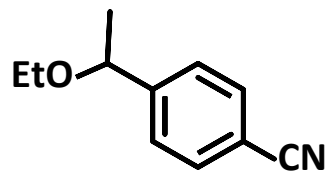

The reaction was performed according to the GP, using 4-bromobenzonitrile (0.2 mmol, 1.0 equiv.), $NiCl_2$·glyme (0.02 mmol, 0.1 equiv.), dtbbpy (0.02 mmol, 0.1 equiv.), nCN_x (50 mg), Na_2CO_3 (0.4 mmol, 2.0 equiv.) in 4 mL of diethyl ether. The mixture was purified via silica gel column chromatography (0 to 10% EtOAc/Hexane) to give product **3ca** as a colorless oil (11 mg, 31% yield).

R_f (5% EtOAc/Hexane): 0.2.

^{1}H NMR (400 MHz, $CDCl_3$) δ 7.64 (2H, d, *J* = 8.2 Hz), 7.43 (2H, d, *J* = 8.1 Hz), 4.45 (1H, q, *J* = 6.6 Hz), 3.44 – 3.30 (2H, m), 1.42 (3H, d, *J* = 6.5 Hz), 1.20 (3H, t, *J* = 7.1 Hz).

^{13}C NMR (101 MHz, $CDCl_3$) δ 150.1, 132.5, 126.9, 119.0, 111.3, 77.3, 64.6, 24.1, 15.5.

This data is consistent with literature precedent.[1]

4-benzylbenzonitrile (3da)

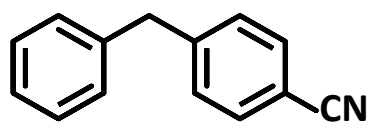

The reaction was performed according to the GP, using 4-bromobenzonitrile (0.2 mmol, 1.0 equiv.), $NiCl_2$·glyme (0.02 mmol, 0.1 equiv.), dtbbpy (0.02 mmol, 0.1 equiv.), nCN_x (50 mg), Na_2CO_3 (0.4 mmol, 2.0 equiv.) in 4 mL of toluene. The mixture was purified via silica gel column chromatography (0 to 5% EtOAc/Hexane) to give product **3da** as a colorless oil (27 mg, 69% yield).

R_f (2% EtOAc/Hexane): 0.3.

^{1}H NMR (400 MHz, $CDCl_3$) δ 7.57 (2H, d, *J* = 8.3 Hz), 7.33 – 7.22 (5H, m), 7.16 (2H, d, *J* = 6.9 Hz), 4.03 (2H, s).

^{13}C NMR (101 MHz, $CDCl_3$) δ 146.9, 139.5, 132.5, 129.8, 129.1, 128.9, 126.8, 119.1, 110.3, 42.1.

This data is consistent with literature precedent.[1]

4-(3,5-dimethylbenzyl)benzonitrile (3ea)

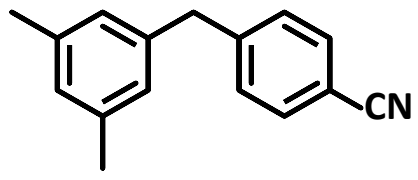

The reaction was performed according to the GP, using 4-bromobenzonitrile (0.2 mmol, 1.0 equiv.), $NiCl_2$·glyme (0.02 mmol, 0.1 equiv.), dtbbpy (0.02 mmol, 0.1 equiv.), nCN_x (50 mg), Na_2CO_3 (0.4 mmol, 2.0 equiv.) in 4 mL of mesitylene. The mixture was purified via silica gel column chromatography (0 to 5% EtOAc/Hexane) to give product **3ea** as a colorless oil (33.2 mg, 75% yield).

R_f (2% EtOAc/Hexane): 0.3.

^{1}H NMR (400 MHz, $CDCl_3$) δ 7.57 (2H, d, J = 8.2 Hz), 7.28 (2H, d, J = 8.0 Hz), 6.88 (1H, s), 6.77 (2H, s), 3.95 (2H, s), 2.28 (6H, s).

^{13}C NMR (101 MHz, $CDCl_3$) δ 147.2, 139.4, 138.5, 132.4, 129.8, 128.4, 126.9, 119.2, 110.1, 42.0, 21.4.

This data is consistent with literature precedent.[4]

***N*-(4-cyanobenzyl)-*N*-methylacetamide (3fa)**

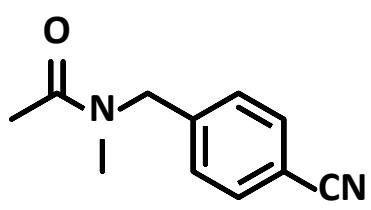

The reaction was performed according to the GP, using 4-bromobenzonitrile (0.2 mmol, 1.0 equiv.), $NiCl_2$·glyme (0.02 mmol, 0.1 equiv.), dtbbpy (0.02 mmol, 0.1 equiv.), nCN_x (50 mg), Na_2CO_3 (0.4 mmol, 2.0 equiv.) in 4 mL of DMA. The mixture was purified via silica gel column chromatography (20 to 80% EtOAc/Hexane) to give product **3fa** as a colorless solid (36.1 mg, 96% yield).

R_f (60% EtOAc/Hexane): 0.2.

^{1}H NMR (400 MHz, $CDCl_3$) δ 7.66 (d, J = 8.2 Hz) & 7.59 (2H combined, d, J = 8.2 Hz), 7.33 (d, J = 8.1 Hz) & 7.28 (2H combined, d, J = 8.1 Hz), 4.61 (s) & 4.57 (2H combined, s), 2.95 (s) & 2.93 (3H combined, s), 2.16 (s) & 2.10 (3H combined, s).

^{13}C NMR (101 MHz, $CDCl_3$) rotameric mixture, resonances for minor rotamer are enclosed in parenthesis: δ 171.0, (171.0), 143.1, (142.2), (132.9), 132.5, 128.6, (127.0), 118.8, (118.5), (111.9), 111.4, (54.1), 50.7, 36.0, (34.0), 21.8, (21.4).

This data is consistent with literature precedent.[5]

N-(4-cyanobenzyl)-N-methylformamide (3ga)

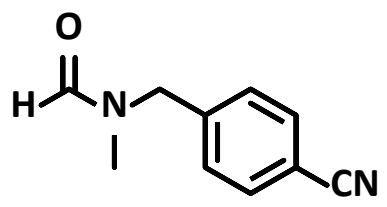

The reaction was performed according to the GP, using 4-bromobenzonitrile (0.2 mmol, 1.0 equiv.), $NiCl_2$·glyme (0.02 mmol, 0.1 equiv.), dtbbpy (0.02 mmol, 0.1 equiv.), nCN_x (50 mg), Na_2CO_3 (0.4 mmol, 2.0 equiv.) in 4 mL of DMF. The mixture was purified via silica gel column chromatography (20 to 80% EtOAc/Hexane) to give product **3ga** as a colorless solid (27.5 mg, 79% yield).

R_f (60% EtOAc/Hexane): 0.2.

^{1}H NMR (400 MHz, $CDCl_3$) δ 8.28 (s) & 8.19 (1H combined, s), 7.68 (d, *J* = 8.3 Hz) & 7.63 (2H combined, d, *J* = 8.2 Hz), 7.36 (d, *J* = 8.1 Hz) & 7.33 (2H combined, d, *J* = 8.3 Hz), 4.57 (s) & 4.46 (2H combined, s), 2.89 (s) & 2.80 (3H combined, s).

^{13}C NMR (101 MHz, $CDCl_3$) rotameric mixture, resonances for minor rotamer are enclosed in parenthesis: δ 162.9, 141.7, (141.5), (132.9), 132.7, 128.9, (128.1), 118.7, (118.4), (112.4), 111.9, (53.2), 47.8, 34.4, (29.9).

This data is consistent with literature precedent.[5]

4-(1-methyl-5-oxopyrrolidin-2-yl)benzonitrile (3ha)

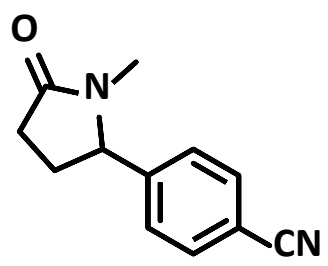

The reaction was performed according to the GP, using 4-bromobenzonitrile (0.2 mmol, 1.0 equiv.), $NiCl_2$·glyme (0.02 mmol, 0.1 equiv.), dtbbpy (0.02 mmol, 0.1 equiv.), nCN_x (50 mg), Na_2CO_3 (0.4 mmol, 2.0 equiv.) in 4 mL of NMP. The mixture was purified via silica gel column chromatography (20 to 100% EtOAc/Pentane) to give product **3ha** as a colorless solid (24.5 mg, 66% yield).

R_f (60% EtOAc/Pentane): 0.2.

^{1}H NMR (400 MHz, $CDCl_3$) δ 7.68 (2H, d, *J* = 8.4 Hz), 7.31 (2H, d, *J* = 8.2 Hz), 4.59 – 4.55 (1H, m), 2.68 (3H, s), 2.60 – 2.41 (3H, m), 1.87 – 1.79 (1H, m).

^{13}C NMR (101 MHz, $CDCl_3$) δ 175.6, 146.8, 133.1, 127.2, 118.5, 112.3, 64.3, 29.9, 28.5, 28.3.

This data is consistent with literature precedent.[1]

4-((2-oxopyrrolidin-1-yl)methyl)benzonitrile (3ha')

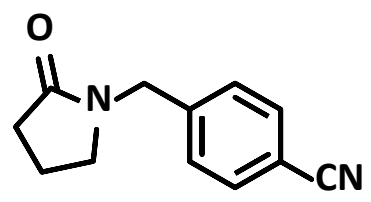

The reaction was performed according to the GP, using 4-bromobenzonitrile (0.2 mmol, 1.0 equiv.), $NiCl_2$·glyme (0.02 mmol, 0.1 equiv.), dtbbpy (0.02 mmol, 0.1 equiv.), nCN_x (50 mg), Na_2CO_3 (0.4 mmol, 2.0 equiv.) in 4 mL of NMP. The mixture was purified via silica gel column chromatography (20 to 100% EtOAc/Pentane) to give product **3ha'** as a colorless solid (12.5 mg, 31% yield).

R_f (60% EtOAc/Pentane): 0.2.

^{1}H NMR (400 MHz, $CDCl_3$) δ 7.62 (2H, d, J = 8.2 Hz), 7.34 (2H, d, J = 8.2 Hz), 4.49 (2H, s), 3.28 (2H, t, J = 7.2 Hz), 2.45 (2H, t, J = 7.9 Hz), 2.07 – 2.00 (2H, m).

^{13}C NMR (101 MHz, $CDCl_3$) δ 175.4, 142.3, 132.7, 128.7, 118.7, 111.7, 46.9, 46.5, 30.7, 17.9.

This data is consistent with literature precedent.[5]

4-(1,2-dimethoxyethyl)benzonitrile (3ja)

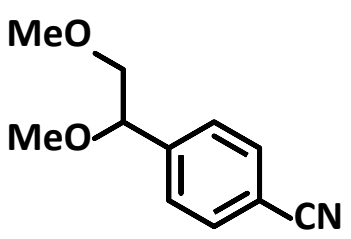

The reaction was performed according to the GP, using 4-bromobenzonitrile (0.2 mmol, 1.0 equiv.), $NiCl_2$·glyme (0.02 mmol, 0.1 equiv.), dtbbpy (0.02 mmol, 0.1 equiv.), nCN_x (50 mg), Na_2CO_3 (0.4 mmol, 2.0 equiv.) in 4 mL of DME. The mixture was purified via silica gel column chromatography (5 to 20% EtOAc/Hexane) to give product **3ja** as a colorless solid (27.5 mg, 63% yield).

R_f (10% EtOAc/Hexane): 0.2.

^{1}H NMR (400 MHz, $CDCl_3$) δ 7.66 (2H, d, J = 8.4 Hz), 7.55 (2H, d, J = 8.1 Hz), 4.41 (1H, dd, J = 7.2, 4.0 Hz), 3.57 (1H, dd, J = 10.3, 7.2 Hz), 3.44 (1H, dd, J = 10.3, 4.1 Hz), 3.37 (3H, s), 3.31 (3H, s).

^{13}C NMR (101 MHz, $CDCl_3$) δ 144.9, 132.4, 127.8, 118.8, 112.0, 82.5, 76.6, 59.5, 57.6.

This data is consistent with literature precedent.[1]

2-(4-(trifluoromethyl)phenyl)tetrahydrofuran (3ab)

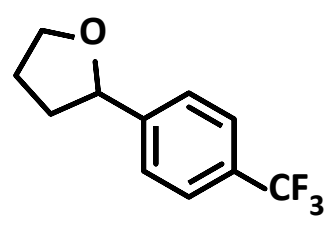

The reaction was performed according to the GP, using 4-bromobenzotrifluoride (0.2 mmol, 1.0 equiv.), $NiCl_2$·glyme (0.02 mmol, 0.1 equiv.), dtbbpy (0.02 mmol, 0.1 equiv.), nCN_x (50 mg), Na_2CO_3 (0.4 mmol, 2.0 equiv.) in 4 mL of THF. The mixture was purified via silica gel column chromatography (0 to 10% EtOAc/Hexane) to give product **3ab** as a colorless oil (38 mg, 88% yield).

R_f (5% EtOAc/Hexane): 0.2.

^{1}H NMR (400 MHz, $CDCl_3$) δ 7.58 (2H, d, *J* = 8.2 Hz), 7.44 (2H, d, *J* = 8.0 Hz), 4.99 (1H, t, *J* = 7.2 Hz), 4.11 (1H, dt, *J* = 8.2, 6.8 Hz), 3.96 (1H, dt, *J* = 8.3, 6.9 Hz), 2.37 (1H, dq, *J* = 12.3, 6.7 Hz), 2.01 (2H, q, *J* = 6.9 Hz), 1.77 (1H, dq, *J* = 12.2, 7.2 Hz).

^{13}C NMR (101 MHz, $CDCl_3$) δ 147.9, 129.5 (d, *J* = 32 Hz), 125.9, 125.4 (q, *J* = 3.8 Hz), 80.1, 69.0, 34.9, 26.1.

^{19}F NMR (376 MHz, $CDCl_3$): δ -62.4

This data is consistent with literature precedent.[1]

methyl 4-(tetrahydrofuran-2-yl)benzoate (3ac)

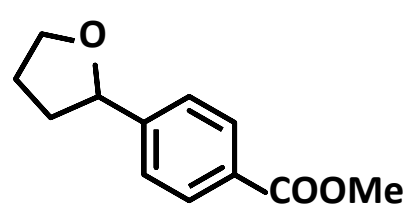

The reaction was performed according to the GP, using methyl 4-bromobenzoate (0.2 mmol, 1.0 equiv.), $NiCl_2$·glyme (0.02 mmol, 0.1 equiv.), dtbbpy (0.02 mmol, 0.1 equiv.), nCN_x (50 mg), Na_2CO_3 (0.4 mmol, 2.0 equiv.) in 4 mL of THF. The mixture was purified via silica gel column chromatography (0 to 20% EtOAc/Hexane) to give product **3ac** as a colorless oil (30.5 mg, 74% yield).

R_f (10% EtOAc/Hexane): 0.2.

^{1}H NMR (400 MHz, $CDCl_3$) δ 8.00 (2H, d, *J* = 8.3 Hz), 7.39 (2H, d, *J* = 8.1 Hz), 4.94 (1H, t, *J* = 7.1 Hz), 4.11 (1H, dt, *J* = 8.2, 6.8 Hz), 3.96 (1H, dt, *J* = 8.3, 6.9 Hz), 3.90 (3H, s), 2.37 (1H, dq, *J* = 12.3, 6.7 Hz), 2.04 – 1.97 (2H, m), 1.78 (1H, dq, *J* = 12.2, 7.8 Hz).

^{13}C NMR (101 MHz, $CDCl_3$) δ 167.2, 149.1, 129.8, 129.1, 125.6, 80.3, 69.0, 52.1, 34.8, 26.1.

This data is consistent with literature precedent.[1]

2-phenyltetrahydrofuran (3ad)

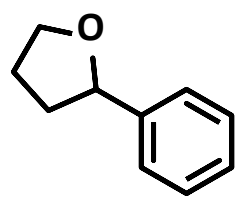

The reaction was performed according to the GP, using bromobenzene (0.2 mmol, 1.0 equiv.), $NiCl_2$·glyme (0.02 mmol, 0.1 equiv.), dtbbpy (0.02 mmol, 0.1 equiv.), nCN_x (50 mg), Na_2CO_3 (0.4 mmol, 2.0 equiv.) in 4 mL of THF. The mixture was purified via silica gel column chromatography (0 to 10% EtOAc/Hexane) to give product **3ad** as a colorless oil (24 mg, 80% yield).

R_f (5% EtOAc/Hexane): 0.3.

^{1}H NMR (400 MHz, $CDCl_3$) δ 7.37 – 7.32 (4H, m), 7.29 – 7.24 (1H, m), 4.91 (1H, t, *J* = 7.0 Hz), 4.11 (1H, dt, *J* = 8.2, 6.8 Hz), 3.95 (1H, dt, *J* = 7.9, 6.4 Hz), 2.34 (1H, dtd, *J* = 12.4, 7.1, 5.5, 6.7 Hz), 2.06 – 1.98 (2H, m), 1.78 (1H, ddt, *J* = 12.1, 8.5, 7.8 Hz).

^{13}C NMR (101 MHz, $CDCl_3$) δ 143.6, 128.4, 127.2, 125.7, 80.8, 68.8, 34.7, 26.1.

This data is consistent with literature precedent.[6]

2-(4-fluorophenyl)tetrahydrofuran (3ae)

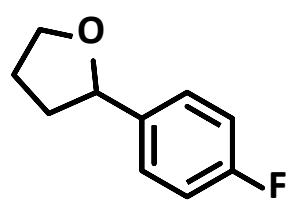

The reaction was performed according to the GP, using 1-bromo-4-fluorobenzene (0.2 mmol, 1.0 equiv.), $NiCl_2$·glyme (0.02 mmol, 0.1 equiv.), dtbbpy (0.02 mmol, 0.1 equiv.), nCN_x (50 mg), Na_2CO_3 (0.4 mmol, 2.0 equiv.) in 4 mL of THF. The mixture was purified via silica gel column chromatography (0 to 10% EtOAc/Hexane) to give product **3ae** as a colorless oil (23.6 mg, 71% yield).

R_f (5% EtOAc/Hexane): 0.2.

^{1}H NMR (400 MHz, $CDCl_3$) δ 7.31 – 7.28 (2H, m), 7.04 – 6.99 (2H, m), 4.85 (1H, t, *J* = 7.1 Hz), 4.08 (1H, dt, *J* = 8.3, 6.8 Hz), 3.92 (1H, dt, *J* = 8.3, 7.0 Hz), 2.31 (1H, dtd, *J* = 12.4, 7.1, 5.6 Hz), 2.04 – 1.97 (2H, m), 1.81 – 1.72 (1H, m).

^{13}C NMR (101 MHz, $CDCl_3$) δ 162.0 (*J* = 240 Hz), 139.1 (d, *J* = 3.1 Hz), 127.3 (d, *J* = 7.8 Hz), 115.1 (d, *J* = 22.6 Hz), 80.1, 68.6, 34.7, 26.0.

^{19}F NMR (376 MHz, $CDCl_3$): δ -116.0.

This data is consistent with literature precedent.[7]

2-(4-chlorophenyl)tetrahydrofuran (3af)

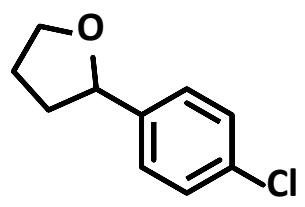

The reaction was performed according to the GP, using 1-bromo-4-chlorobenzene (0.2 mmol, 1.0 equiv.), $NiCl_2$·glyme (0.02 mmol, 0.1 equiv.), dtbbpy (0.02 mmol, 0.1 equiv.), nCN_x (50 mg), Na_2CO_3 (0.4 mmol, 2.0 equiv.) in 4 mL of THF. The mixture was purified via silica gel column chromatography (0 to 10% EtOAc/Hexane) to give product **3af** as a colorless oil (29 mg, 79% yield).

R_f (5% EtOAc/Hexane): 0.2.

^{1}H NMR (400 MHz, $CDCl_3$) δ 7.31 – 7.25 (4H, m), 4.86 (1H, t, *J* = 7.3 Hz), 4.08 (1H, dt, *J* = 8.3, 6.8 Hz), 3.93 (1H, dt, *J* = 8.3, 7.0 Hz), 2.35 – 2.27 (1H, m), 2.04 – 1.96 (2H, m), 1.79 – 1.70 (1H, m).

^{13}C NMR (101 MHz, $CDCl_3$) δ 142.2, 132.9, 128.5, 127.1, 80.1, 68.8, 34.8, 26.1.

This data is consistent with literature precedent.[1]

2-(4-bromophenyl)tetrahydrofuran (3ag)

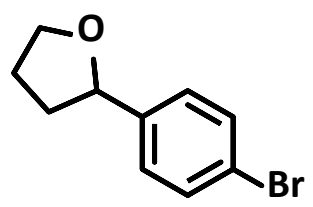

The reaction was performed according to the GP, using 1,4-dibromobenzene (0.2 mmol, 1.0 equiv.), $NiCl_2$·glyme (0.02 mmol, 0.1 equiv.), dtbbpy (0.02 mmol, 0.1 equiv.), nCN_x (50 mg), Na_2CO_3 (0.4 mmol, 2.0 equiv.) in 4 mL of THF. The mixture was purified via silica gel column chromatography (0 to 10% EtOAc/Hexane) to give product **3ag** as a colorless oil (36.8 mg, 81% yield).

R_f (5% EtOAc/Hexane): 0.3.

^{1}H NMR (400 MHz, $CDCl_3$) δ 7.45 (2H, d, *J* = 8.4 Hz), 7.21 (2H, d, *J* = 8.4 Hz), 4.84 (1H, t, *J* = 7.1 Hz), 4.08 (1H, dt, *J* = 8.3, 6.8 Hz), 3.93 (1H, dt, *J* = 8.3, 7.0 Hz), 2.31 (1H, dq, *J* = 13.0, 6.7 Hz), 2.00 (2H, p, *J* = 6.7 Hz), 1.79 – 1.70 (1H, m).

^{13}C NMR (101 MHz, $CDCl_3$) δ 142.7, 131.5, 127.5, 120.9, 80.2, 68.9, 34.8, 26.1.

This data is consistent with literature precedent.[8]

2-(4-methoxyphenyl)tetrahydrofuran (3ah)

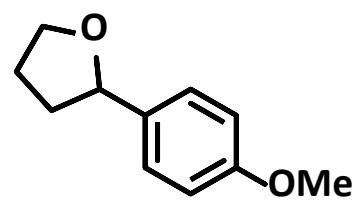

The reaction was performed according to the GP, using 4-bromoanisole (0.2 mmol, 1.0 equiv.), $NiCl_2$·glyme (0.02 mmol, 0.1 equiv.), dtbbpy (0.02 mmol, 0.1 equiv.), nCN_x (50 mg), Na_2CO_3 (0.4 mmol, 2.0 equiv.) in 4 mL of THF. The mixture was purified via silica gel column chromatography (0 to 10% EtOAc/Hexane) to give product **3ah** as a colorless oil (29.2 mg, 82% yield).

R_f (5% EtOAc/Hexane): 0.2.

^{1}H NMR (400 MHz, $CDCl_3$) δ 7.26 (2H, d, *J* = 8.5 Hz), 6.87 (2H, d, *J* = 8.6 Hz), 4.83 (1H, t, *J* = 7.3 Hz), 4.08 (1H, dt, *J* = 8.0, 6.6 Hz), 3.91 (1H, dt, *J* = 7.9, 6.3 Hz), 3.80 (3H, s), 2.31 – 2.24 (1H, m), 2.05 – 1.95 (2H, m), 1.84 – 1.75 (1H, m).

^{13}C NMR (101 MHz, $CDCl_3$) δ 159.0, 135.5, 127.1, 113.9, 80.6, 68.6, 55.4, 34.6, 26.2.

This data is consistent with literature precedent.[1]

2-(4-tolyl)tetrahydrofuran (3ai)

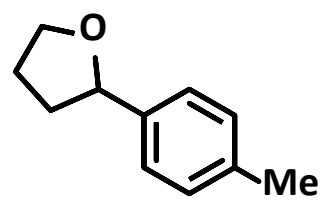

The reaction was performed according to the GP, using 4-bromotoluene (0.2 mmol, 1.0 equiv.), $NiCl_2$·glyme (0.02 mmol, 0.1 equiv.), dtbbpy (0.02 mmol, 0.1 equiv.), nCN_x (50 mg), Na_2CO_3 (0.4 mmol, 2.0 equiv.) in 4 mL of THF. The mixture was purified via silica gel column chromatography (0 to 10% EtOAc/Hexane) to give product **3ai** as a colorless oil (25.6 mg, 79% yield).

R_f (5% EtOAc/Hexane): 0.2.

^{1}H NMR (400 MHz, $CDCl_3$) δ 7.24 (2H, d, *J* = 8.0 Hz), 7.16 (2H, d, *J* = 7.9 Hz), 4.87 (1H, t, *J* = 7.2 Hz), 4.10 (1H, dt, *J* = 8.3, 6.8 Hz), 3.94 (1H, dt, *J* = 8.3, 7.0 Hz), 2.35 (3H, s), 2.34 – 2.27 (1H, m), 2.05 – 1.97 (2H, m), 1.85 – 1.77 (1H, m).

^{13}C NMR (101 MHz, $CDCl_3$) δ 140.5, 136.8, 129.1, 125.7, 80.7, 68.7, 34.7, 26.2, 21.2.

This data is consistent with literature precedent.[9]

2-(4-(*tert*-butyl)phenyl)tetrahydrofuran (3aj)

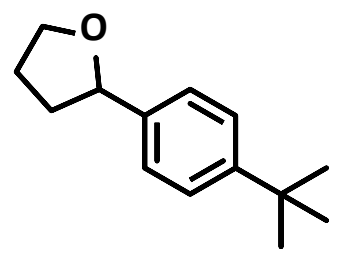

The reaction was performed according to the GP, using 1-bromo-4-*tert*-butylbenzene (0.2 mmol, 1.0 equiv.), $NiCl_2$·glyme (0.02 mmol, 0.1 equiv.), dtbbpy (0.02 mmol, 0.1 equiv.), nCN$_x$ (50 mg), Na_2CO_3 (0.4 mmol, 2.0 equiv.) in 4 mL of THF. The mixture was purified via silica gel column chromatography (0 to 10% EtOAc/Hexane) to give product **3aj** as a colorless oil (33 mg, 81% yield).

R$_f$ (5% EtOAc/Hexane): 0.2.

^{1}H NMR (400 MHz, CDCl$_3$) δ 7.36 (2H, d, *J* = 8.3 Hz), 7.27 (2H, d, *J* = 8.4 Hz), 4.87 (1H, t, *J* = 7.1 Hz), 4.09 (1H, dt, *J* = 8.3, 6.8 Hz), 3.93 (1H, dt, *J* = 8.3, 7.0 Hz), 2.34 – 2.26 (1H, m), 2.06 – 1.95 (2H, m), 1.88 – 1.79 (1H, m), 1.32 (9H, s).

^{13}C NMR (101 MHz, CDCl$_3$) δ 150.2, 140.5, 125.6, 125.3, 80.7, 68.7, 34.6, 34.4, 31.5, 26.2.

This data is consistent with literature precedent.[8]

3-methyl-4-(tetrahydrofuran-2-yl)benzonitrile (3ak)

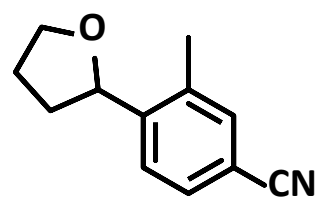

The reaction was performed according to the GP, using 4-bromo-3-methylbenzonitrile (0.2 mmol, 1.0 equiv.), $NiCl_2$·glyme (0.02 mmol, 0.1 equiv.), dtbbpy (0.02 mmol, 0.1 equiv.), nCN$_x$ (50 mg), Na_2CO_3 (0.4 mmol, 2.0 equiv.) in 4 mL of THF. The mixture was purified via silica gel column chromatography (0 to 5% EtOAc/Hexane) to give product **3ak** as a colorless oil (35.6 mg, 95% yield).

R$_f$ (5% EtOAc/Hexane): 0.3.

^{1}H NMR (400 MHz, CDCl$_3$) δ 7.55 (1H, d, *J* = 8.0 Hz), 7.46 (1H, d, *J* = 8.0 Hz), 7.39 (1H, s) 5.04 (1H, t, *J* = 7.2 Hz), 4.16 – 4.11 (1H, m), 3.94 (1H, dt, *J* = 8.3, 6.9 Hz), 2.44 – 2.36 (1H, m), 2.31 (3H, s), 2.07 – 1.93 (2H, m), 1.59 (1H, dq, *J* = 12.3, 7.6 Hz).

^{13}C NMR (101 MHz, CDCl$_3$) δ 147.8, 135.4, 133.5, 130.0, 125.4, 119.2, 110.5, 77.5, 69.0, 33.2, 26.1, 19.1.

2-(3-phenylpropyl)tetrahydrofuran (3am)

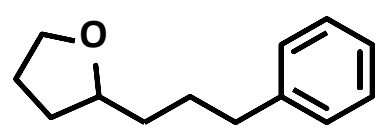

The reaction was performed according to the GP, using 1-bromo-3-phenylpropane (0.2 mmol, 1.0 equiv.), $NiCl_2$·glyme (0.02 mmol, 0.1 equiv.), dtbbpy (0.02 mmol, 0.1 equiv.), nCN_x (50 mg), Na_2CO_3 (0.4 mmol, 2.0 equiv.) in 4 mL of THF. The mixture was purified via silica gel column chromatography (0 to 10% EtOAc/Hexane) to give product **3am** as a colorless oil (20.6 mg, 64% yield).

R_f (2.5% EtOAc/Hexane): 0.2.

^{1}H NMR (400 MHz, $CDCl_3$) δ 7.29 – 7.26 (2H, m), 7.20 – 7.15 (3H, m), 3.88 – 3.78 (2H, m), 3.71 (1H, dt, *J* = 8.3, 7.0 Hz), 2.65 (2H, t, *J* = 6.6 Hz), 2.00 – 1.72 (4H, m), 1.70 – 1.58 (2H, m), 1.55 – 1.39 (2H, m).

^{13}C NMR (101 MHz, $CDCl_3$) δ 142.6, 128.6, 128.4, 125.8, 79.4, 67.8, 36.1, 35.4, 31.5, 28.3, 25.9.

This data is consistent with literature precedent.[6]

***N*-methyl-*N*-(4-phenylbutyl)acetamide (3fm)**

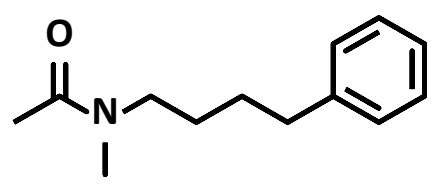

The reaction was performed according to the GP, using 1-bromo-3-phenylpropane (0.2 mmol, 1.0 equiv.), $NiCl_2$·glyme (0.02 mmol, 0.1 equiv.), dtbbpy (0.02 mmol, 0.1 equiv.), nCN_x (50 mg), Na_2CO_3 (0.4 mmol, 2.0 equiv.) in 4 mL of DMA. The mixture was purified via silica gel column chromatography (30 to 80% EtOAc/Hexane) to give product **3fm** as a colorless oil (30.8 mg, 75% yield).

R_f (60% EtOAc/Hexane): 0.2.

^{1}H NMR (400 MHz, $CDCl_3$) δ 7.30 – 7.25 (2H, m), 7.21 – 7.15 (3H, m), 3.88 – 3.78 (2H, m), 3.37 (1H, t, *J* = 6.7 Hz), 3.25 (1H, t, *J* = 7.0 Hz), 2.93 & 2.88 (3H, s), 2.64 (2H, q, *J* = 6.7 Hz), 2.05 (3H, s), 1.67 – 1.51 (4H, m).

^{13}C NMR (101 MHz, $CDCl_3$), rotameric mixture, resonances for minor rotamer are enclosed in parenthesis: δ 170.5, (170.4), 142.4, (141.8), 128.5, (128.5), 128.4, (128.4), 126.1, (125.8), 50.8, (47.3), 36.1, (35.7), 35.7, (33.3), 28.7, (28.5), 28.0, (26.9), 22.0, (21.3).

(4-(2-methoxyethoxy)butyl)benzene (3jm)

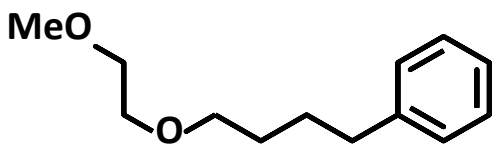

The reaction was performed according to the GP, using 1-bromo-3-phenylpropane (0.2 mmol, 1.0 equiv.), $NiCl_2$·glyme (0.02 mmol, 0.1 equiv.), dtbbpy (0.02 mmol, 0.1 equiv.), nCN_x (50 mg), Na_2CO_3 (0.4 mmol, 2.0 equiv.) in 4 mL of DME. The mixture was purified via silica gel column chromatography (5 to 20% EtOAc/Hexane) to give product **3jm** as a colorless oil (17.5 mg, 42% yield).

R_f (10% EtOAc/Hexane): 0.2.

^{1}H NMR (400 MHz, $CDCl_3$) δ 7.29 – 7.25 (2H, m), 7.18 – 7.15 (3H, m), 3.58 – 3.55 (2H, m), 3.54 – 3.52 (2H, m), 3.48 (2H, t, *J* = 6.4 Hz), 3.38 (3H, s), 2.63 (2H, t, *J* = 7.3 Hz), 1.71 – 1.60 (4H, m).

^{13}C NMR (101 MHz, $CDCl_3$), δ 142.6, 128.6, 128.4, 125.8, 72.2, 71.5, 70.2, 59.2, 35.9, 29.4, 28.1.

This data is consistent with literature precedent.[6]

ethyl 4-(9-(tetrahydrofuran-2-yl)-5,6-dihydro-11H-benzo[5,6]cyclohepta[1,2-b]pyridin-11-ylidene) piperidine-1-carboxylate (3an)

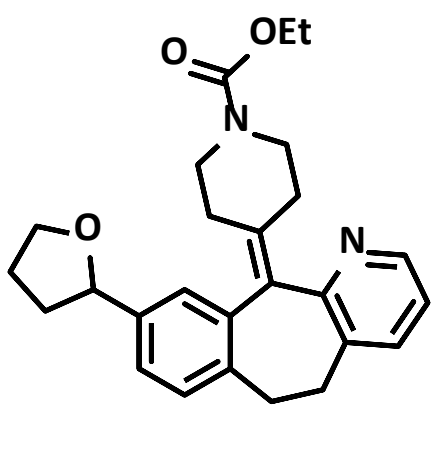

The reaction was performed according to the GP, using loratadine (0.2 mmol, 1.0 equiv.), $NiCl_2$·glyme (0.02 mmol, 0.1 equiv.), dtbbpy (0.02 mmol, 0.1 equiv.), nCN_x (50 mg), Na_2CO_3 (0.4 mmol, 2.0 equiv.) in 4 mL of THF. The mixture was purified via silica gel column chromatography (0 to 50% EtOAc/Hexane) to give product **3an** colorless solid (36 mg, 43% yield).

R_f (20% EtOAc/Hexane): 0.2.

^{1}H NMR (400 MHz, $CDCl_3$) δ 8.35 (1H, dd, *J* = 4.8, 1.8 Hz), 7.40 (1H, d, *J* = 7.7 Hz), 7.15 – 7.02 (4H, m), 4.79 (1H, td, *J* = 7.3, 2.7 Hz), 4.13 – 4.01 (3H, m), 3.90 – 3.74 (3H, m), 3.43 – 3.28 (2H, m), 3.15 – 3.07 (2H, m), 2.87 – 2.78 (2H, m), 2.49 – 2.42 (1H, m), 2.35 (2H, t, *J* = 5.6 Hz), 2.29 – 2.21 (2H, m), 1.99 – 1.91 (2H, m), 1.81 – 1.72 (1H, m), 1.22 (3H, t, *J* = 7.1 Hz).

^{13}C NMR (101 MHz, $CDCl_3$) rotameric mixture, resonances for minor rotamer are enclosed in parenthesis: δ 157.7, (157.7), 155.6, 146.5, 142.5, (142.5), (138.1), 138.1, (137.7), 137.5, 137.4, (137.4), 136.7, 135.2, 133.8, 129.4, (129.3), 126.6, (126.3), 123.7, (123.4), 122.1, 80.5, (80.5), 68.6, 61.3, 45.0, (44.9), 34.3, (34.3), 32.0, 31.8, (31.8), 30.8, (30.6), 26.1, 14.7.

This data is consistent with literature precedent.[9]

1,3,7-trimethyl-8-(tetrahydrofuran-2-yl)-3,7-dihydro-1H-purine-2,6-dione (3ao)

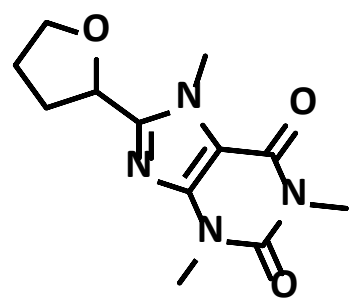

The reaction was performed according to the GP, using 8-bromocaffeine (0.2 mmol, 1.0 equiv.), $NiCl_2$·glyme (0.02 mmol, 0.1 equiv.), dtbbpy (0.02 mmol, 0.1 equiv.), nCN_x (50 mg), Na_2CO_3 (0.4 mmol, 2.0 equiv.) in 4 mL of THF. The mixture was purified via silica gel column chromatography (5 to 30% EtOAc/Hexane) to give product **3ao** as a colorless solid (34 mg, 64% yield).

R_f (20% EtOAc/Hexane): 0.3.

^{1}H NMR (400 MHz, $CDCl_3$) δ 4.97 (1H, dd, J = 7.4, 6.4 Hz), 3.97 (3H, s), 3.93 – 3.83 (2H, m), 3.49 (3H, s), 3.32 (3H, s), 2.56 – 2.47 (1H, m), 2.27 – 2.19 (1H, m), 2.15 – 2.06 (1H, m), 2.04 – 1.93 (1H, m)

^{13}C NMR (101 MHz, $CDCl_3$), δ 177.9, 155.5, 152.4, 151.7, 147.3, 108.3, 72.6, 68.9, 32.2, 29.6, 27.9, 25.9.

This data is consistent with literature precedent.[10]

isopropyl 2-methyl-2-(4-(4-(tetrahydrofuran-2-yl)benzoyl)phenoxy)propanoate (3ap)

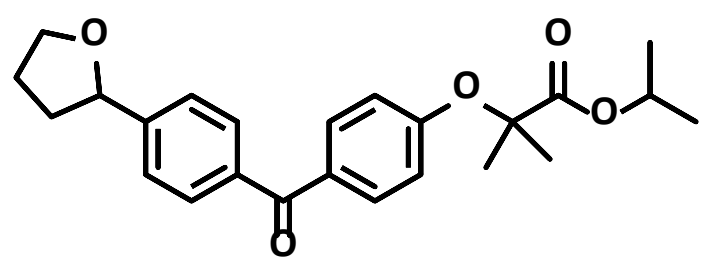

The reaction was performed according to the GP, using fenofibrate (0.2 mmol, 1.0 equiv.), $NiCl_2$·glyme (0.02 mmol, 0.1 equiv.), dtbbpy (0.02 mmol, 0.1 equiv.), nCN_x (50 mg), Na_2CO_3 (0.4 mmol, 2.0 equiv.) in 4 mL of THF. The mixture was purified via silica gel column chromatography (5 to 30% EtOAc/Hexane) to give product **3ap** as a colorless solid (28.5 mg, 36% yield).

R_f (10% EtOAc/Hexane): 0.2.

^{1}H NMR (400 MHz, $CDCl_3$) δ 7.76 – 7.71 (4H, m), 7.42 (2H, d, *J* = 8.0 Hz), 6.87 (2H, d, *J* = 8.9 Hz), 5.08 (1H, hept, *J* = 6.3 Hz), 4.96 (1H, t, *J* = 7.2 Hz), 4.12 (1H, dt, *J* = 8.3, 6.8 Hz), 3.97 (1H, dt, *J* = 8.3, 6.9 Hz), 2.42 – 1.34 (1H, m), 2.03 (2H, p, *J* = 7.2 Hz), 1.86 – 1.77 (1H, m), 1.65 (6H, s), 1.20 (6H, d, *J* = 6.3 Hz)

^{13}C NMR (101 MHz, $CDCl_3$), δ 195.4, 173.3, 159.6, 148.1, 137.1, 132.1, 131.0, 130.1, 125.5, 117.3, 80.4, 79.5, 69.4, 69.0, 34.8, 26.1, 25.5, 21.7.

NMR spectra

4-(tetrahydrofuran-2-yl)benzonitrile (3aa)

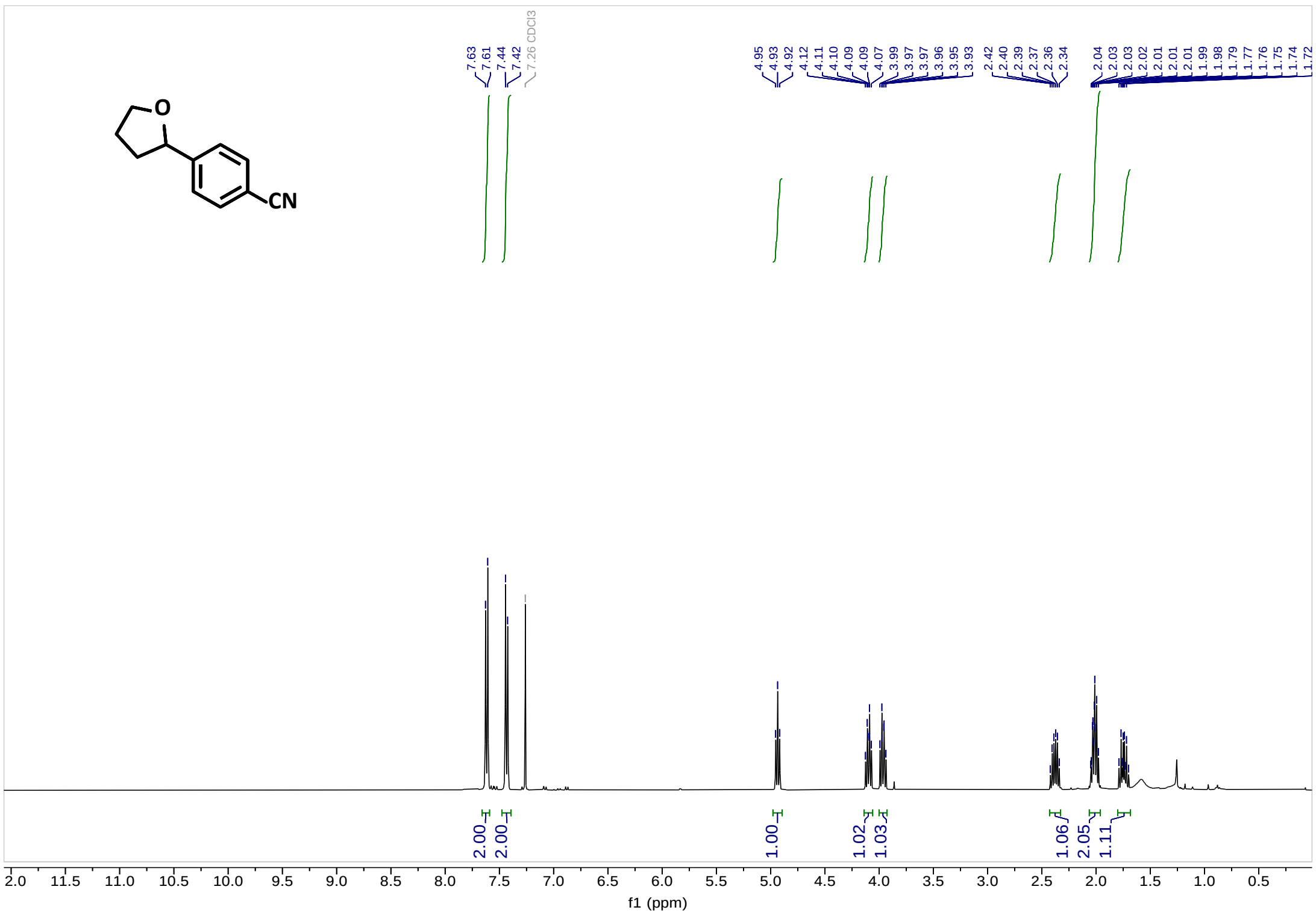

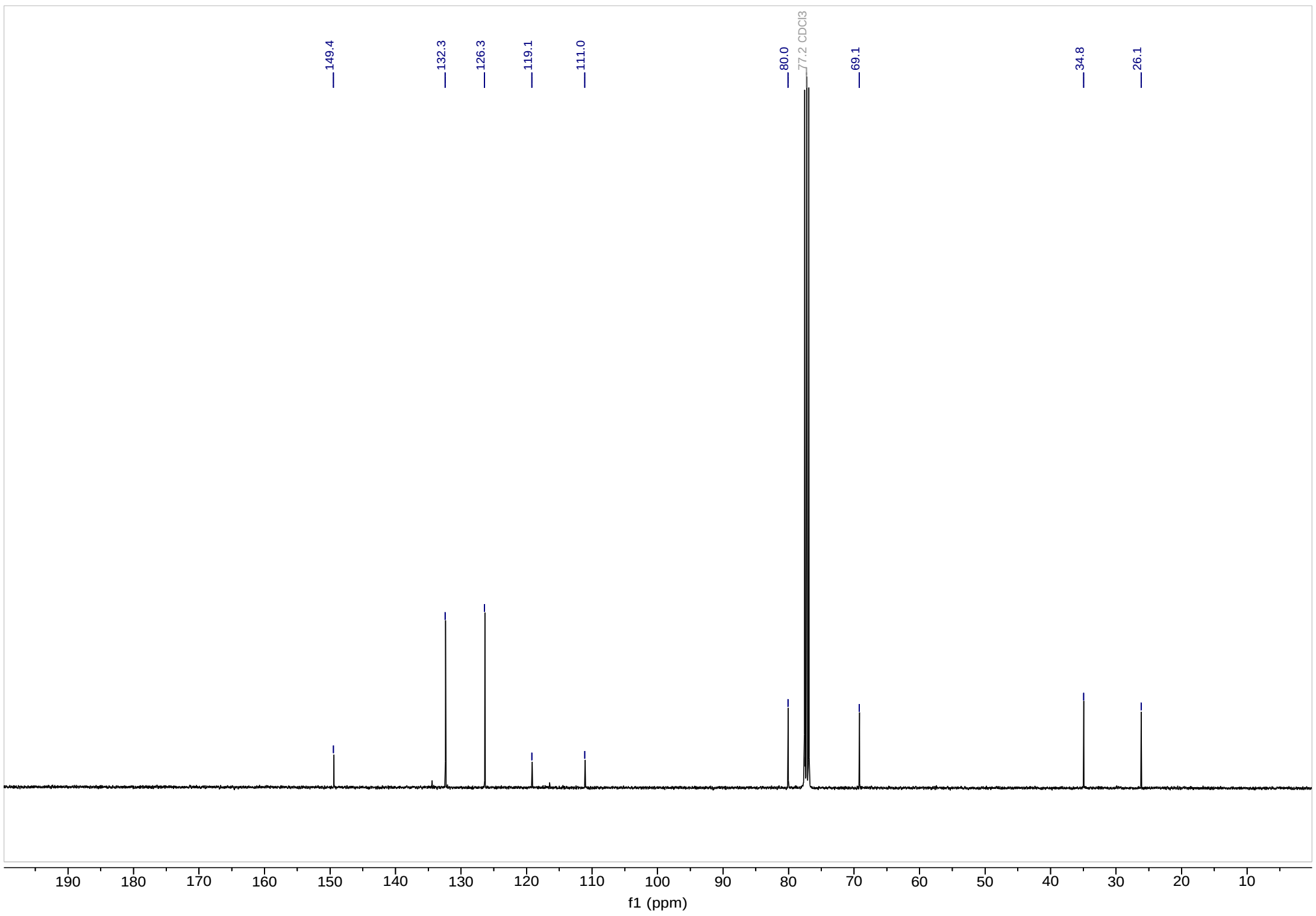

4-(5-methyltetrahydrofuran-2-yl)benzonitrile (3ba)

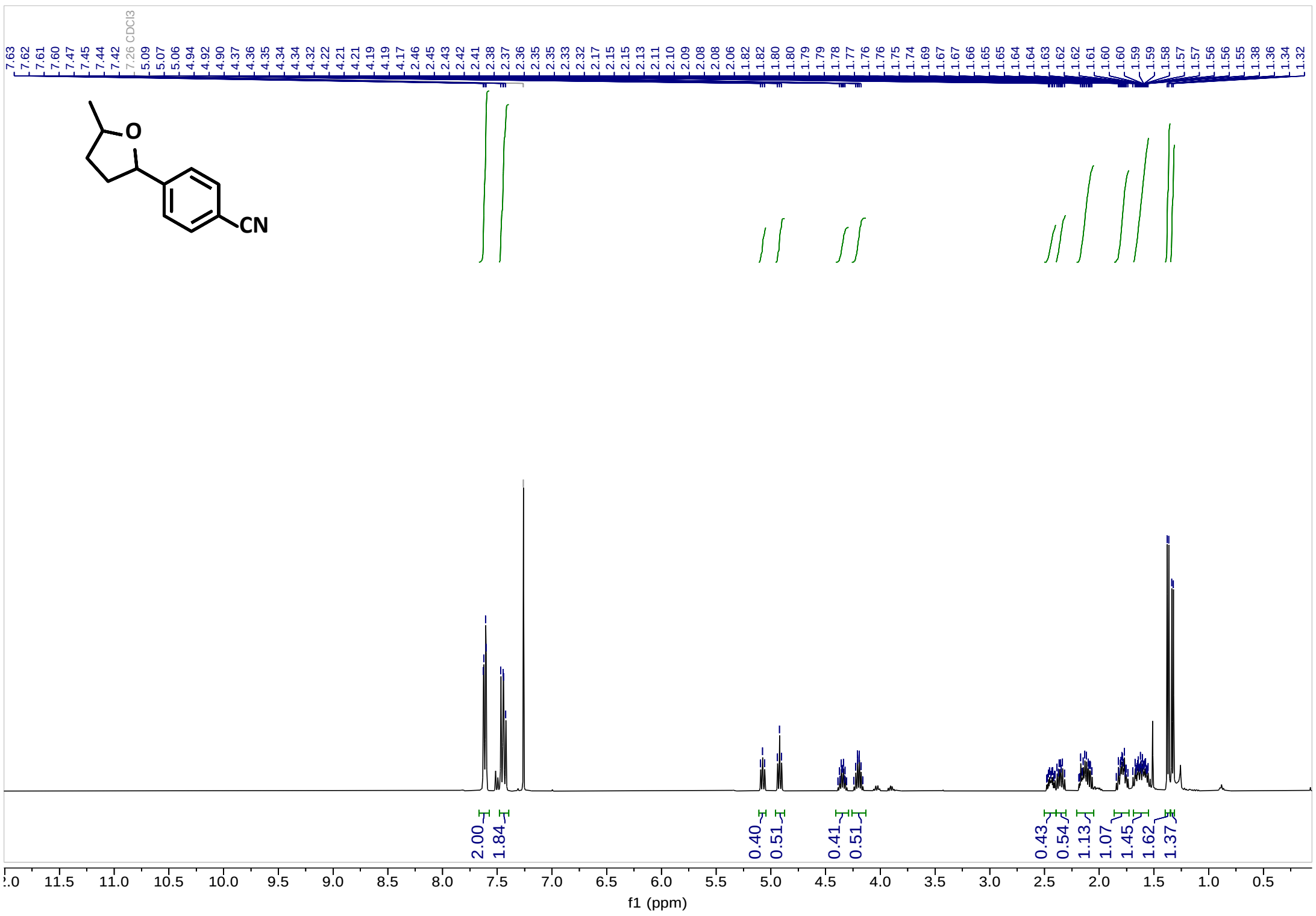

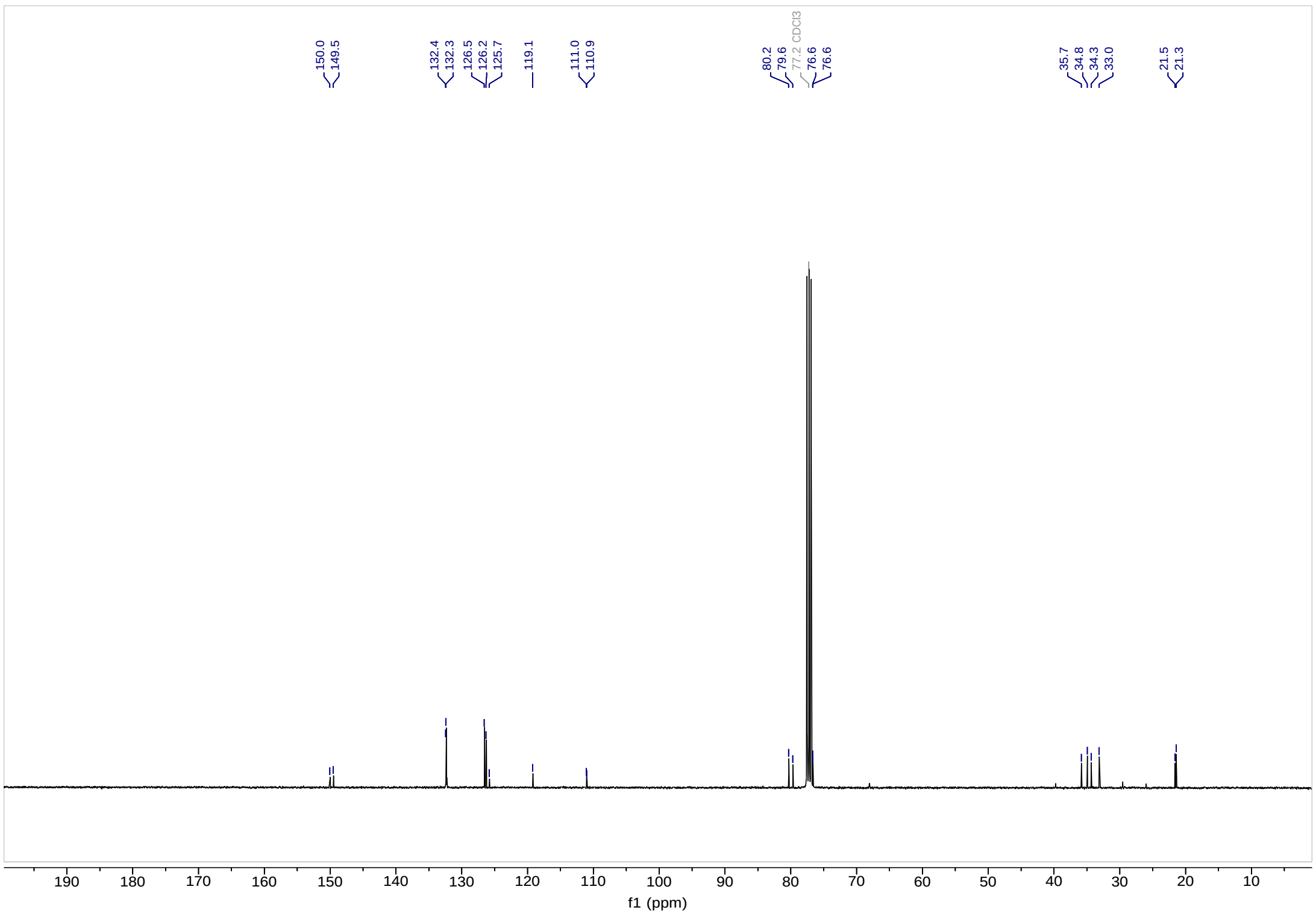

4-(2-methyltetrahydrofuran-2-yl)benzonitrile (3ba')

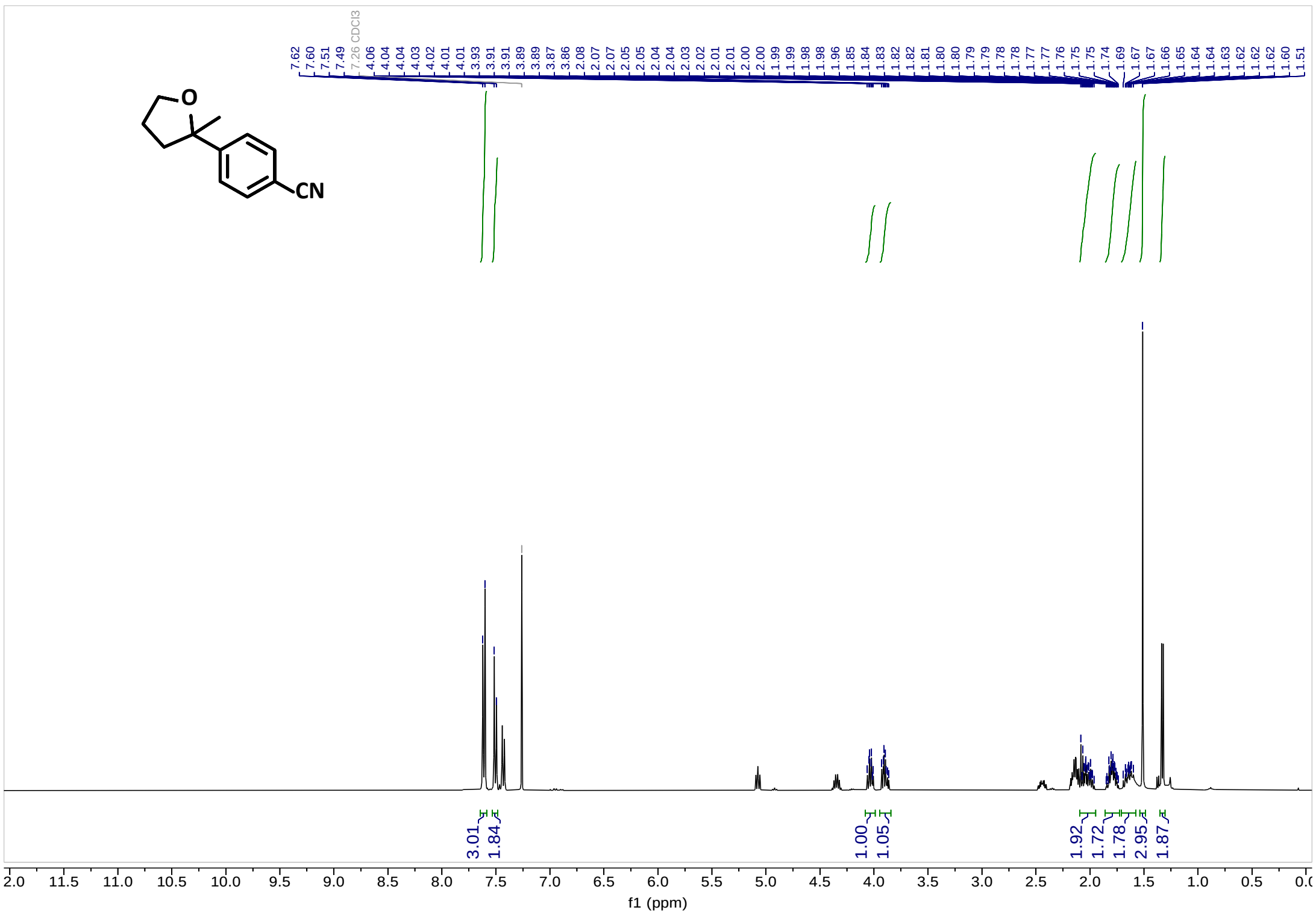

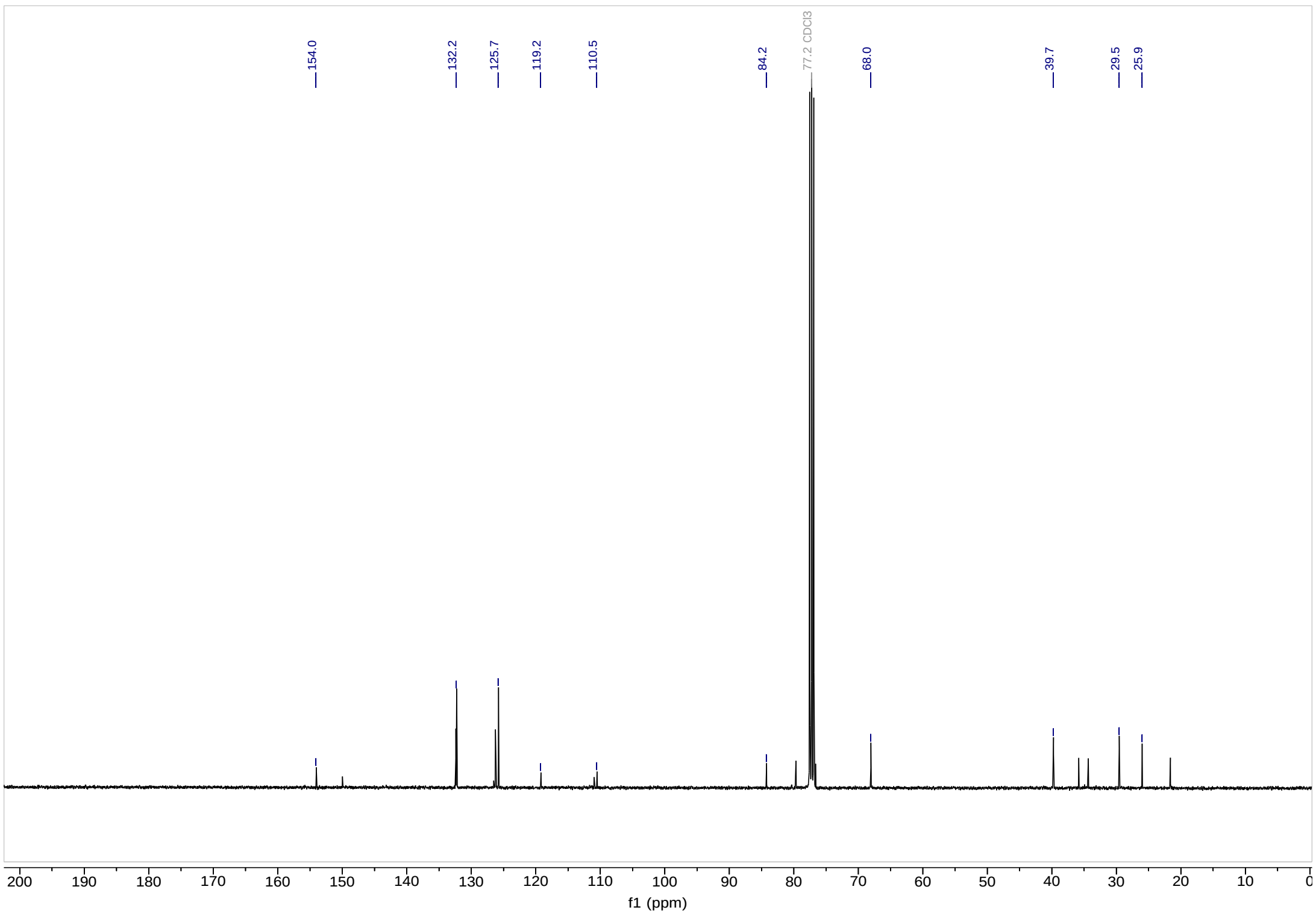

4-(1-ethoxyethyl)benzonitrile (3ca)

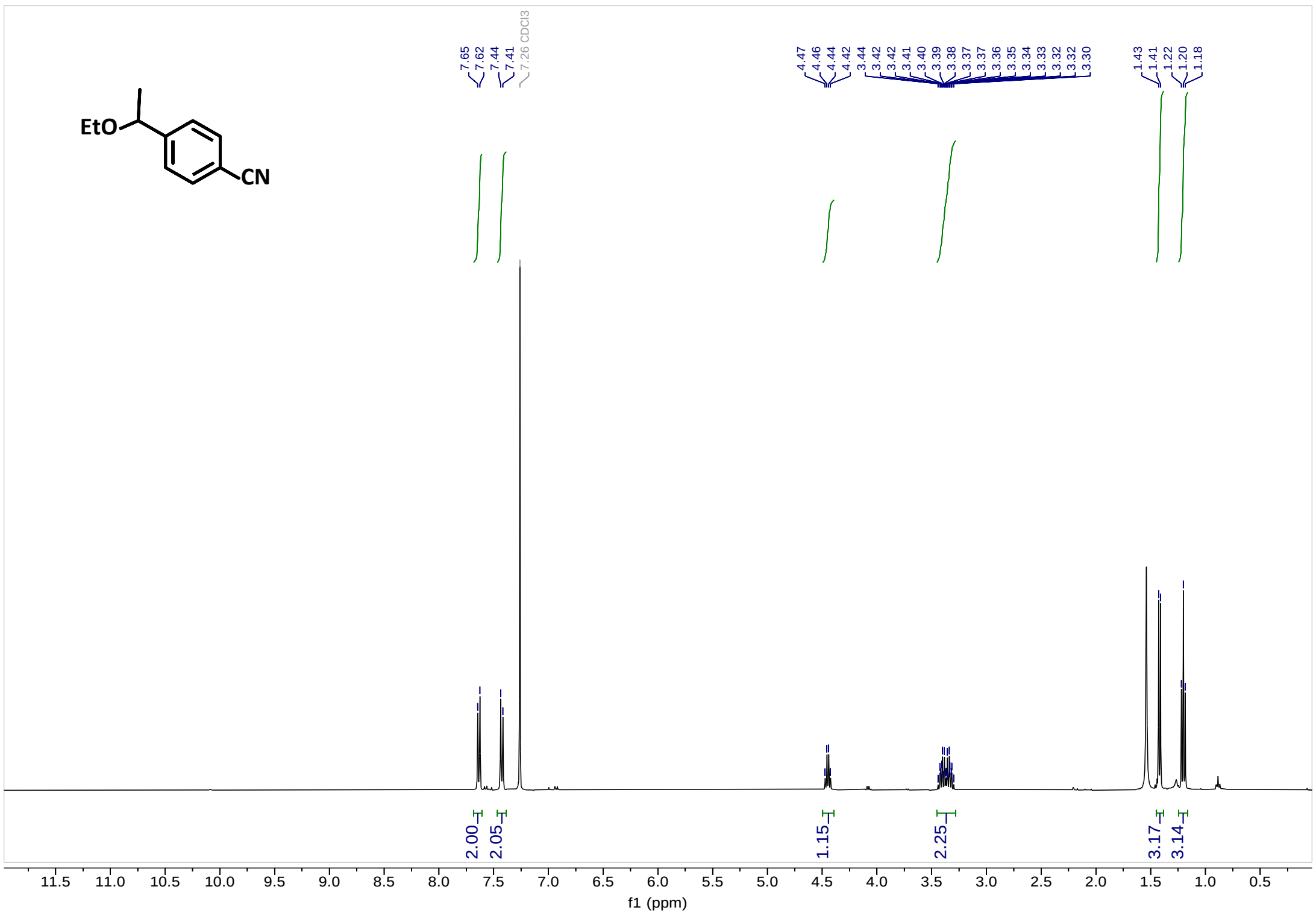

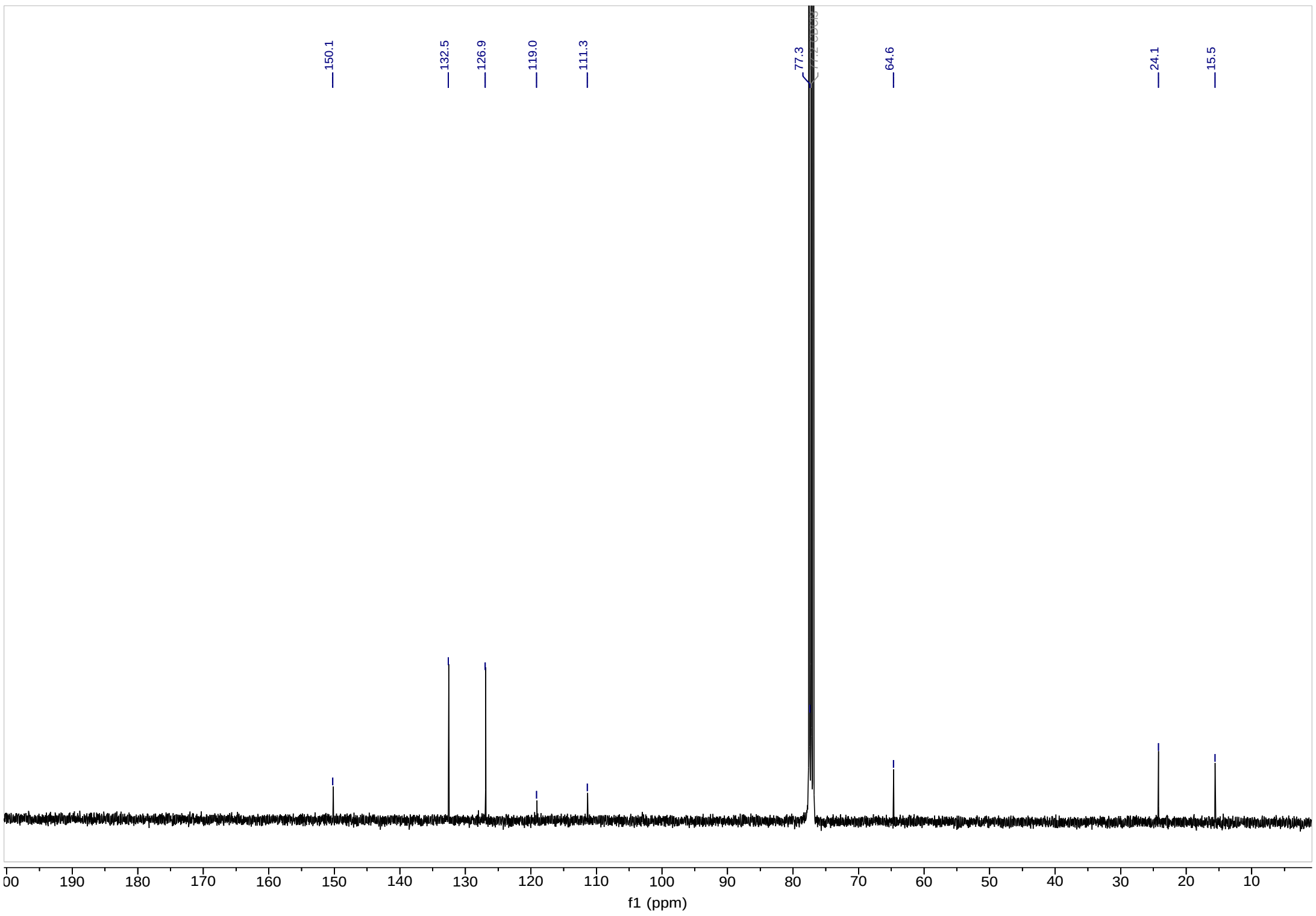

4-benzylbenzonitrile (3da)

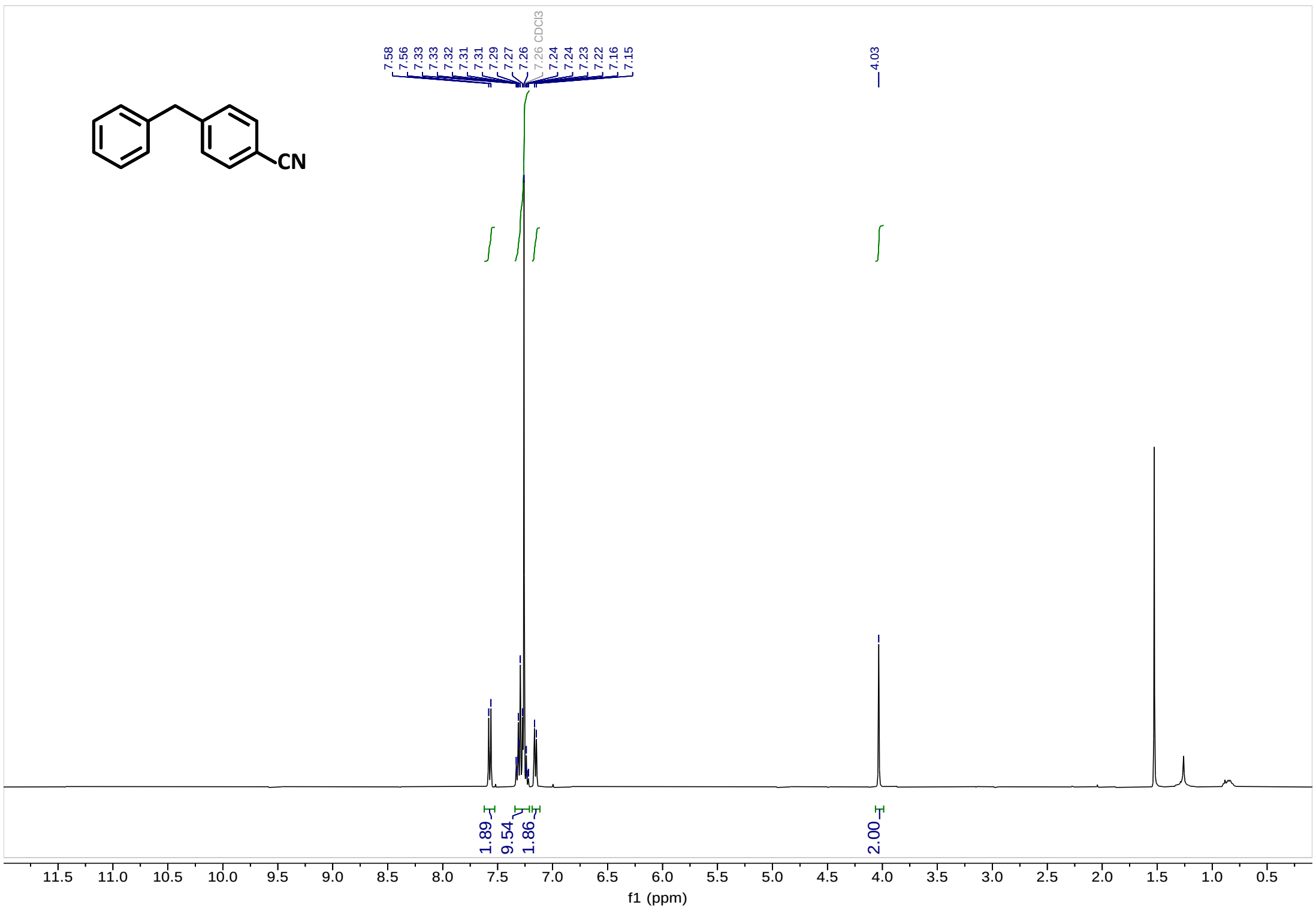

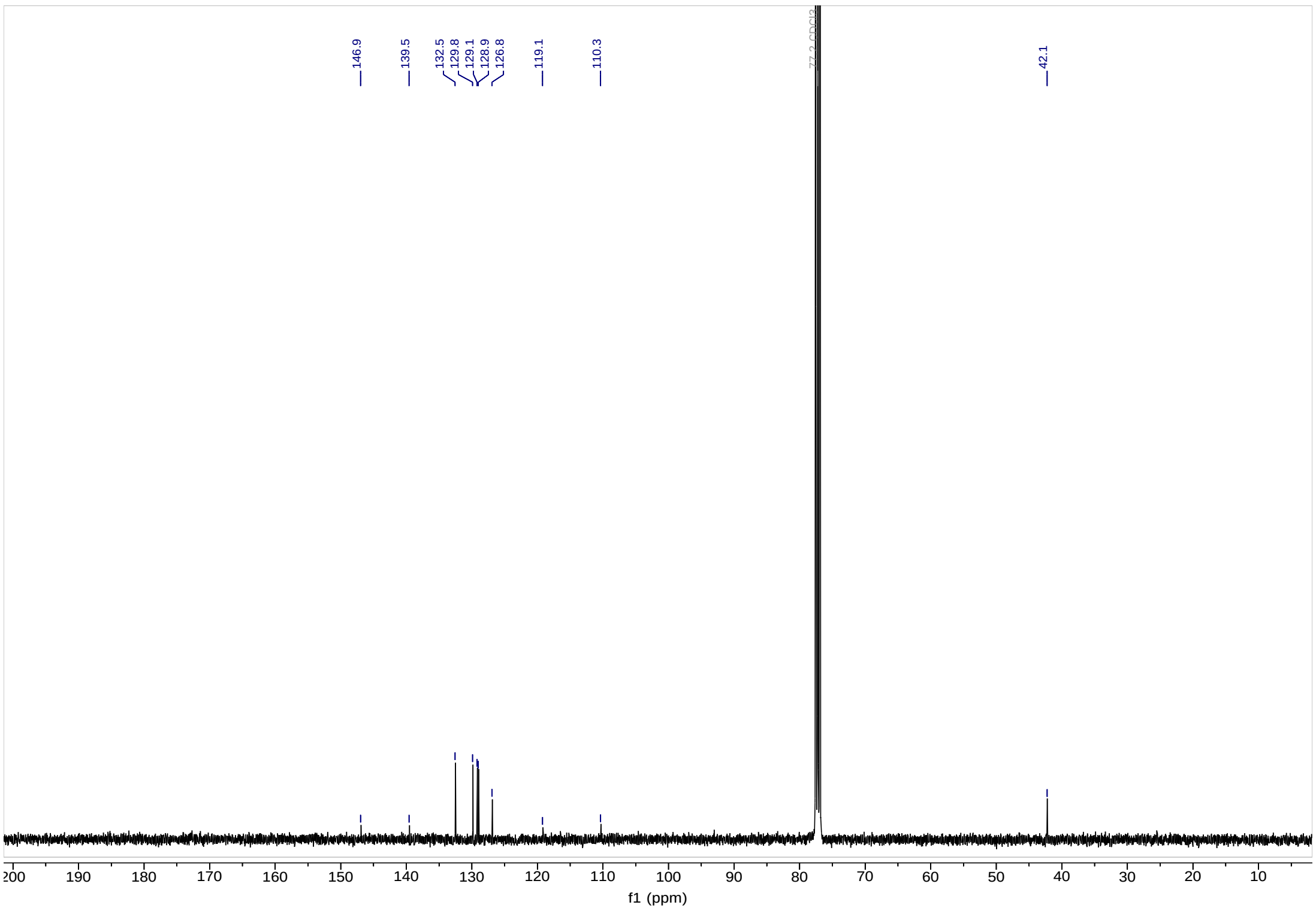

4-(3,5-dimethylbenzyl)benzonitrile (3ea)

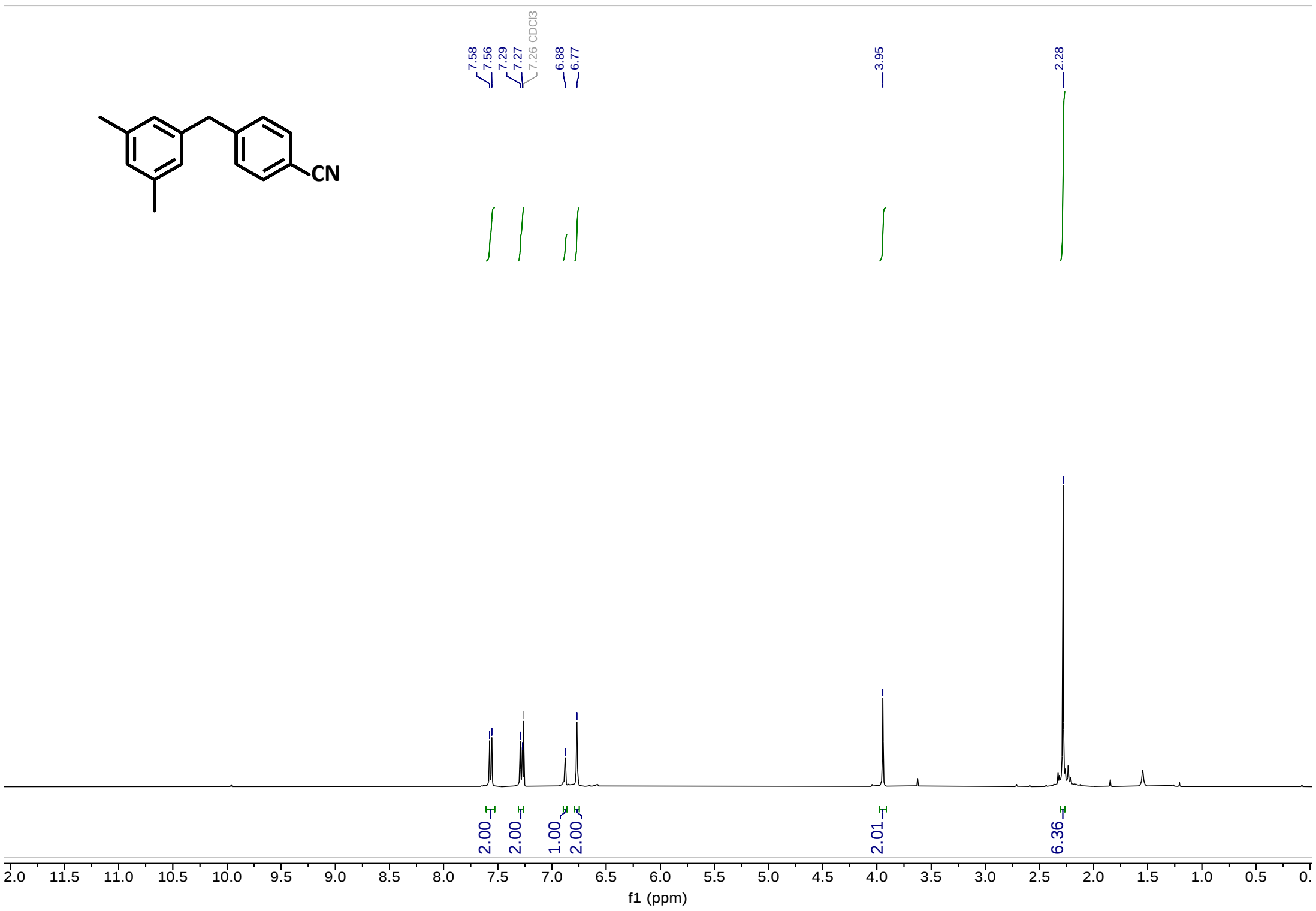

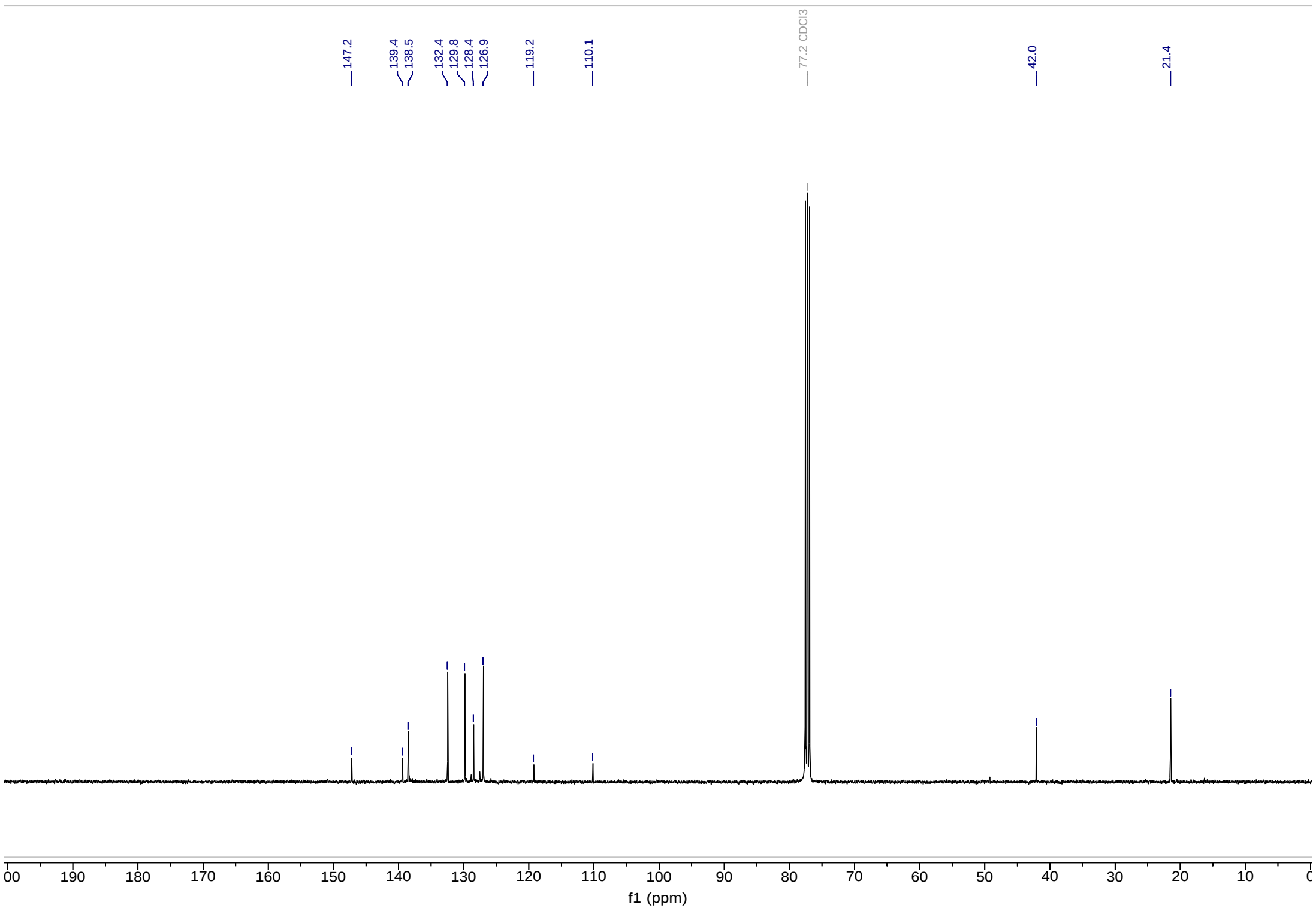

N-(4-cyanobenzyl)-*N*-methylacetamide (3fa)

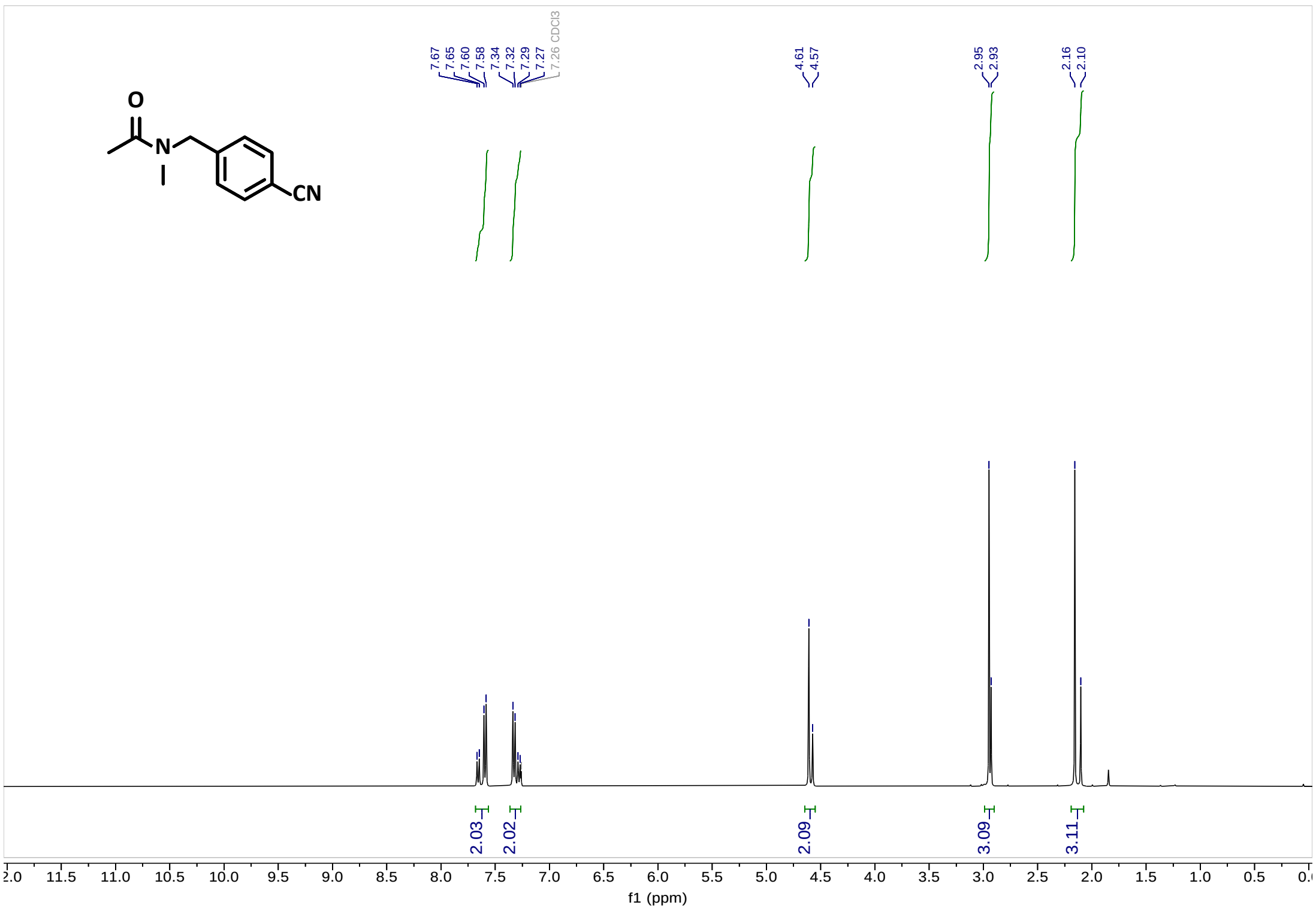

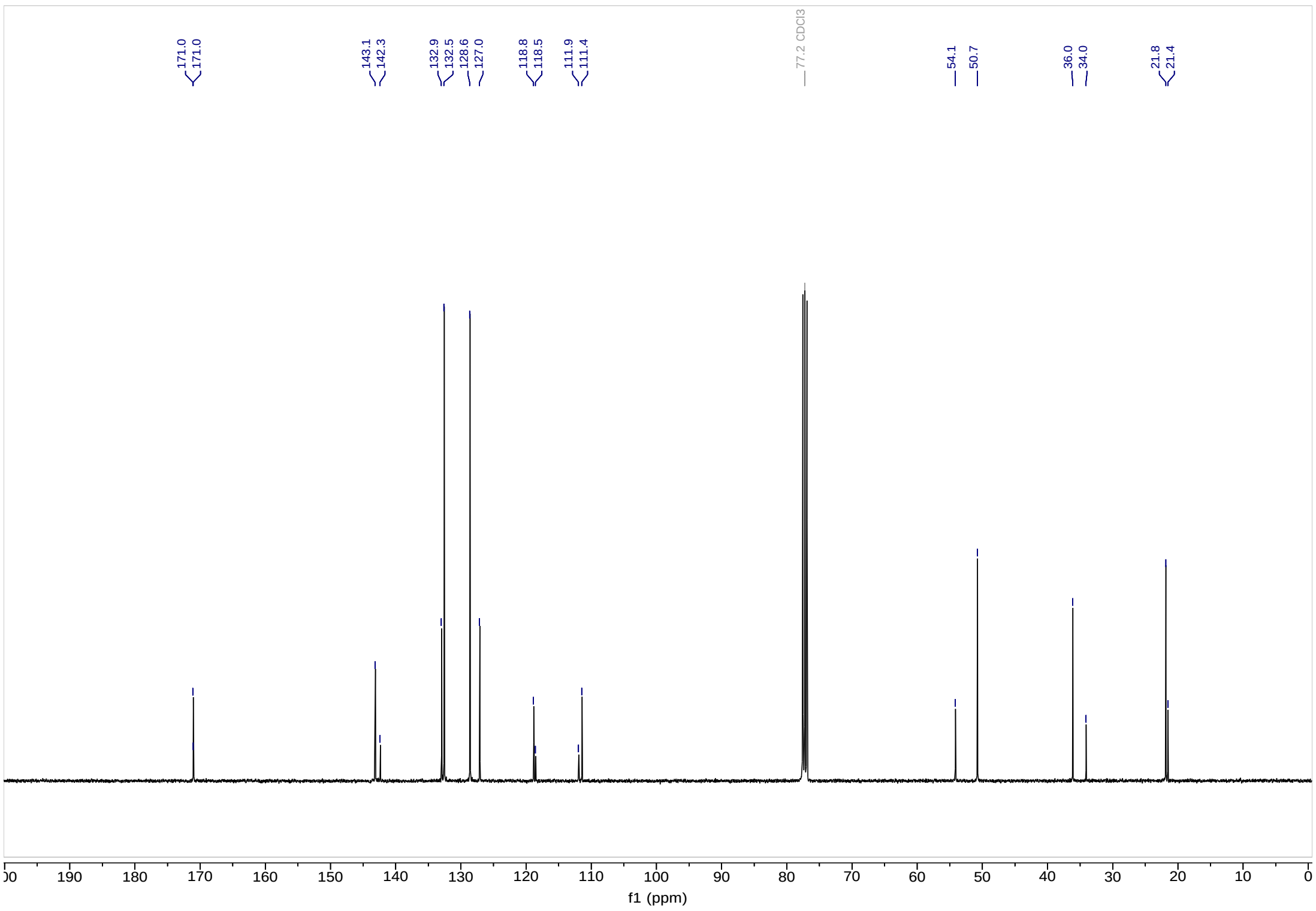

N-(4-cyanobenzyl)-*N*-methylformamide (3ga)

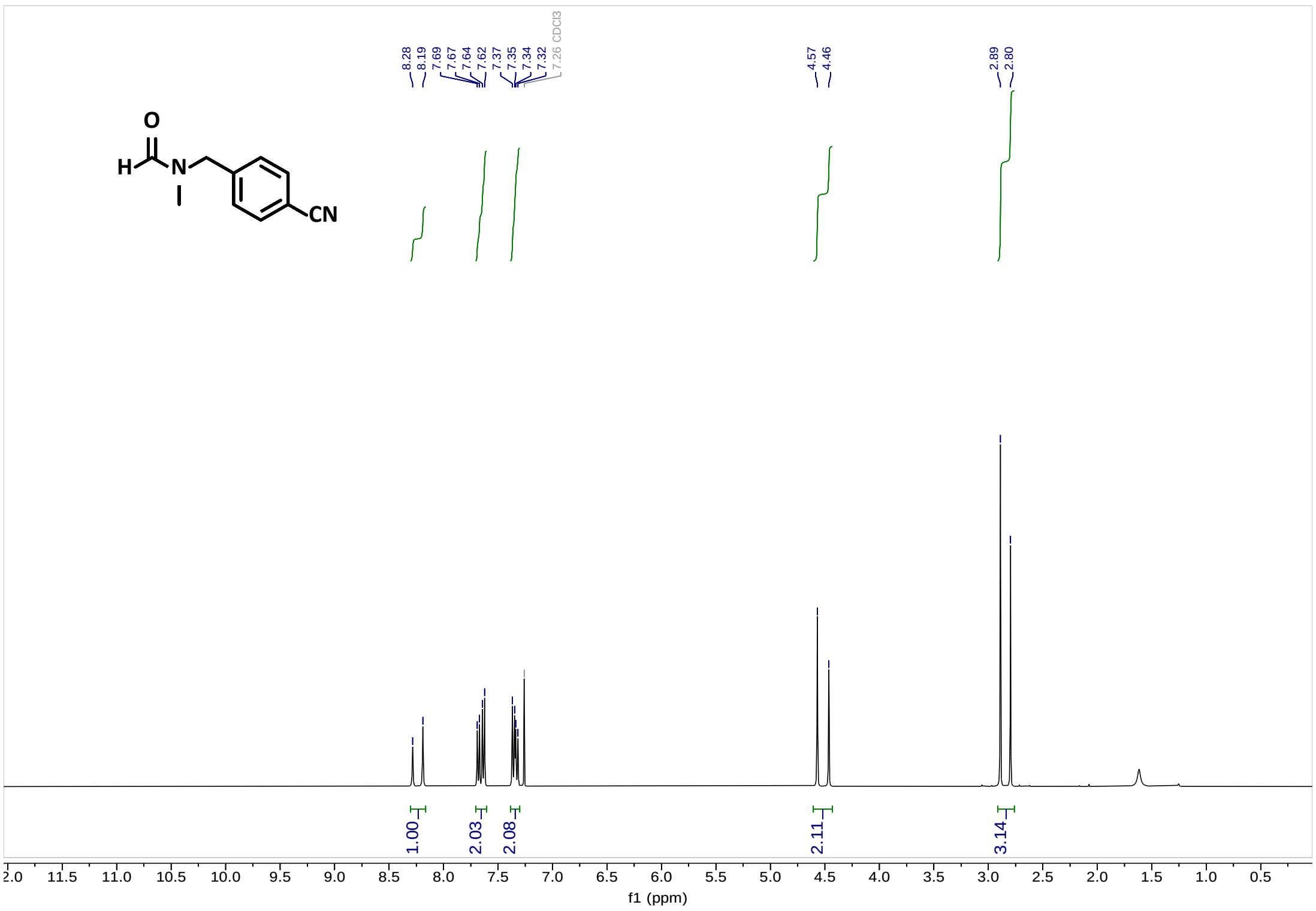

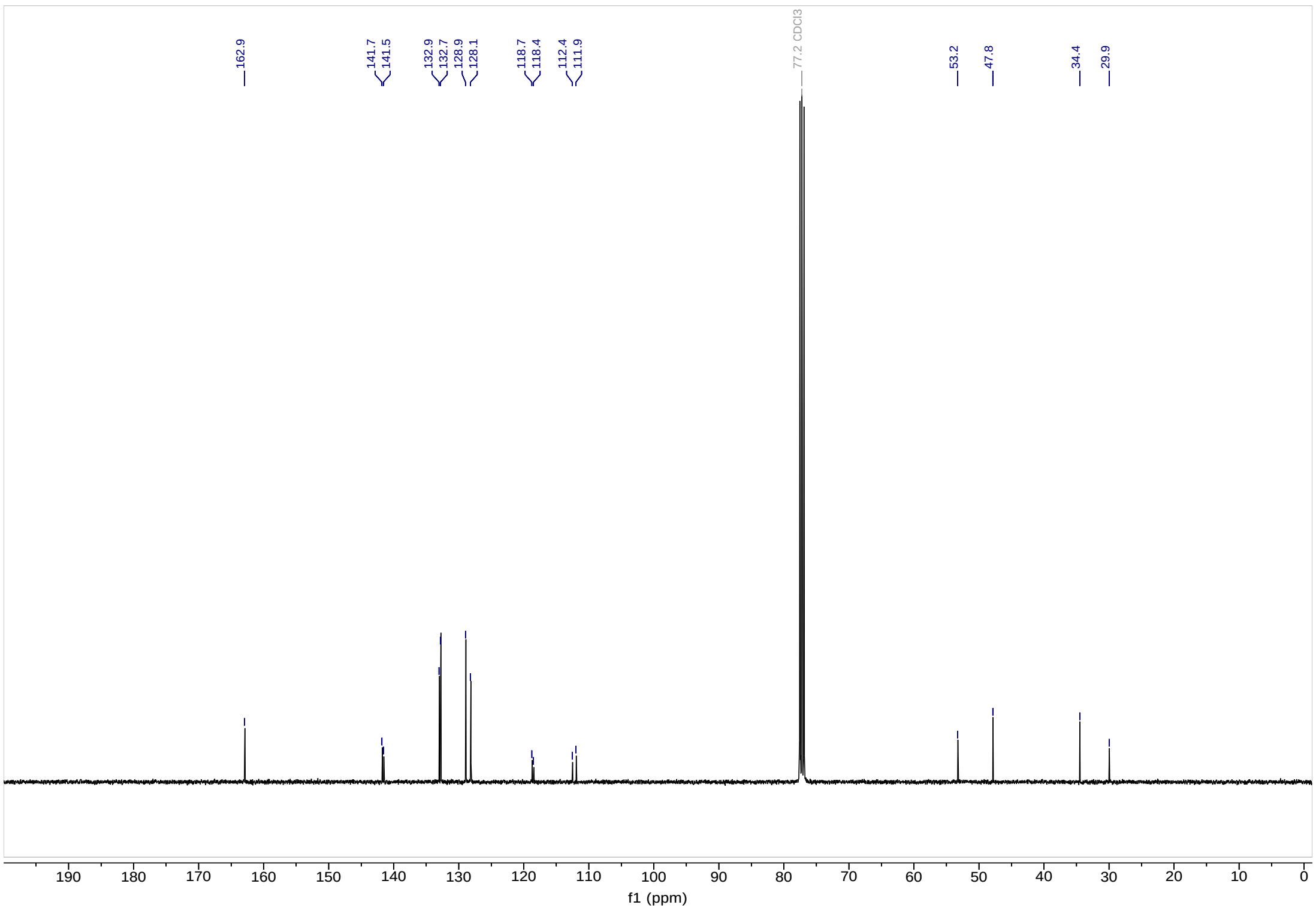

4-(1-methyl-5-oxopyrrolidin-2-yl)benzonitrile (3ha)

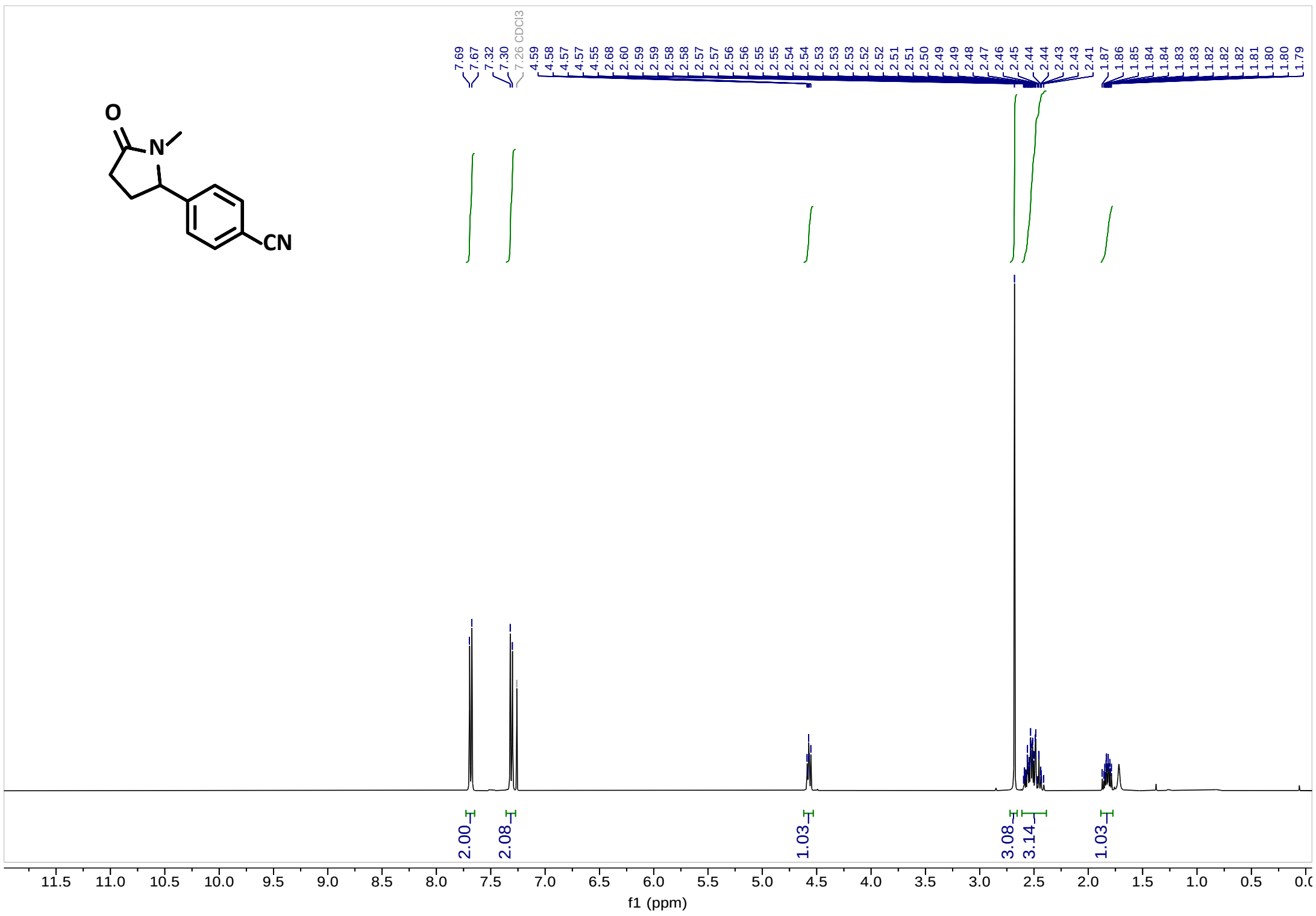

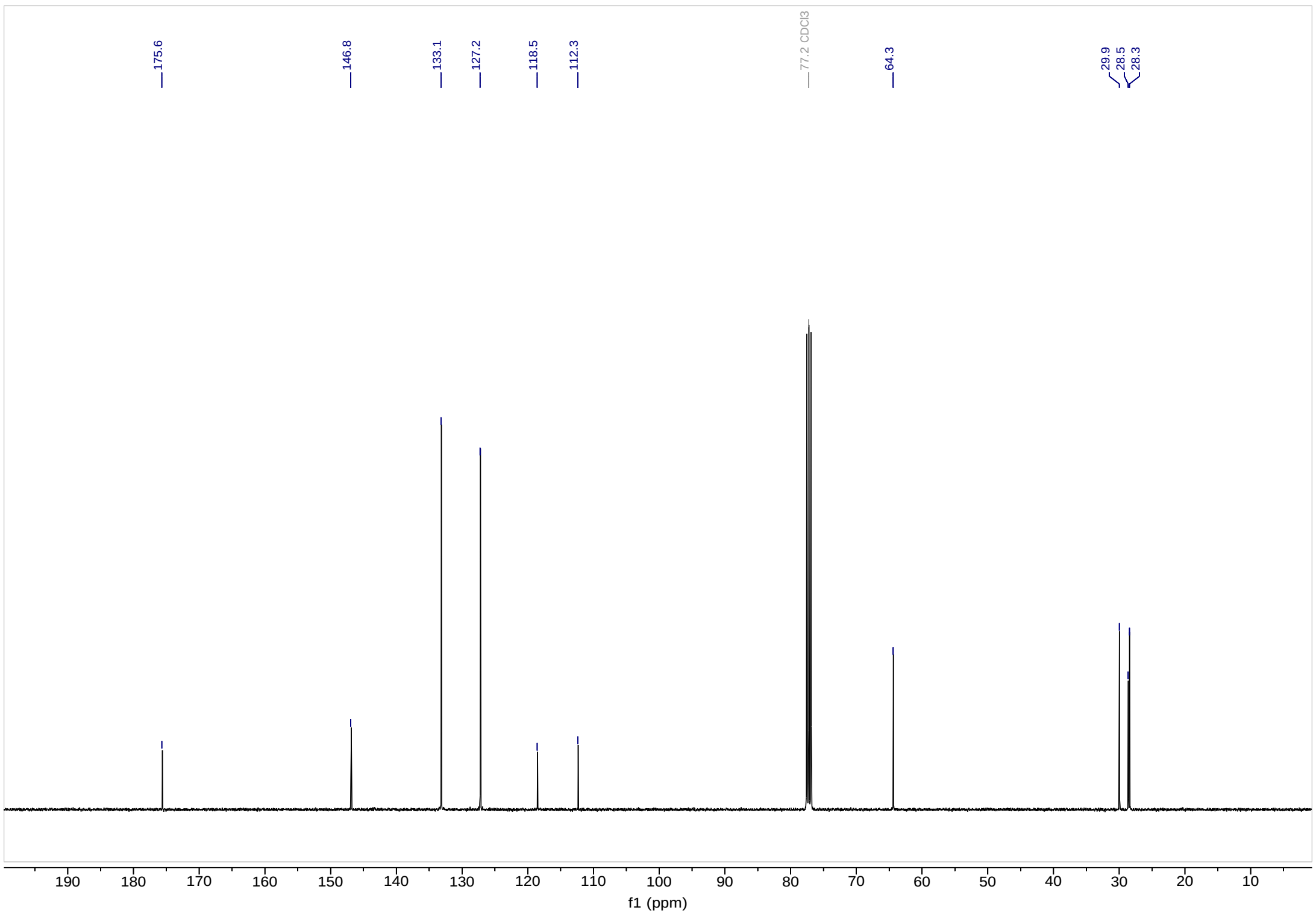

4-((2-oxopyrrolidin-1-yl)methyl)benzonitrile (3ha')

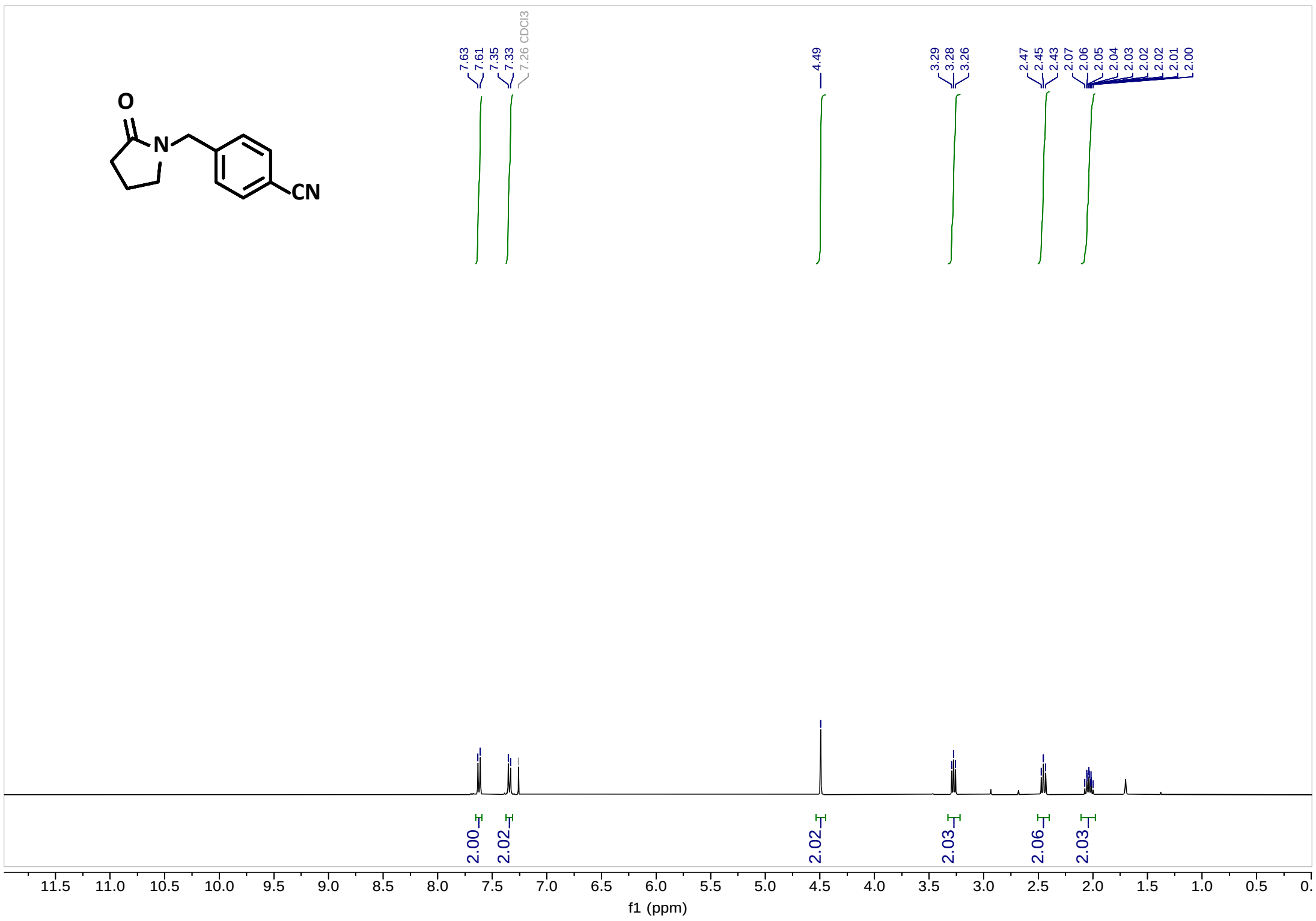

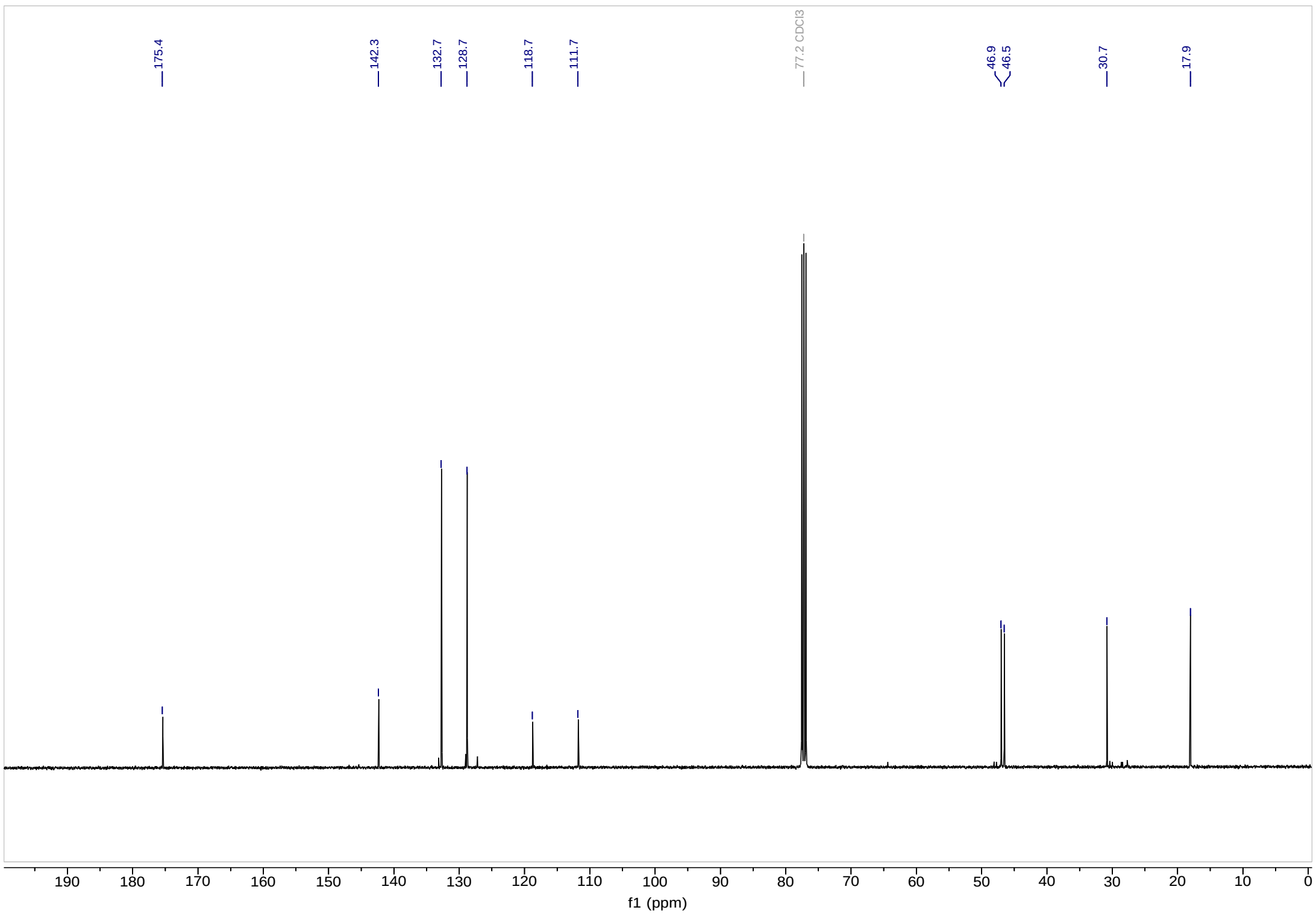

4-(1,2-dimethoxyethyl)benzonitrile (3ja)

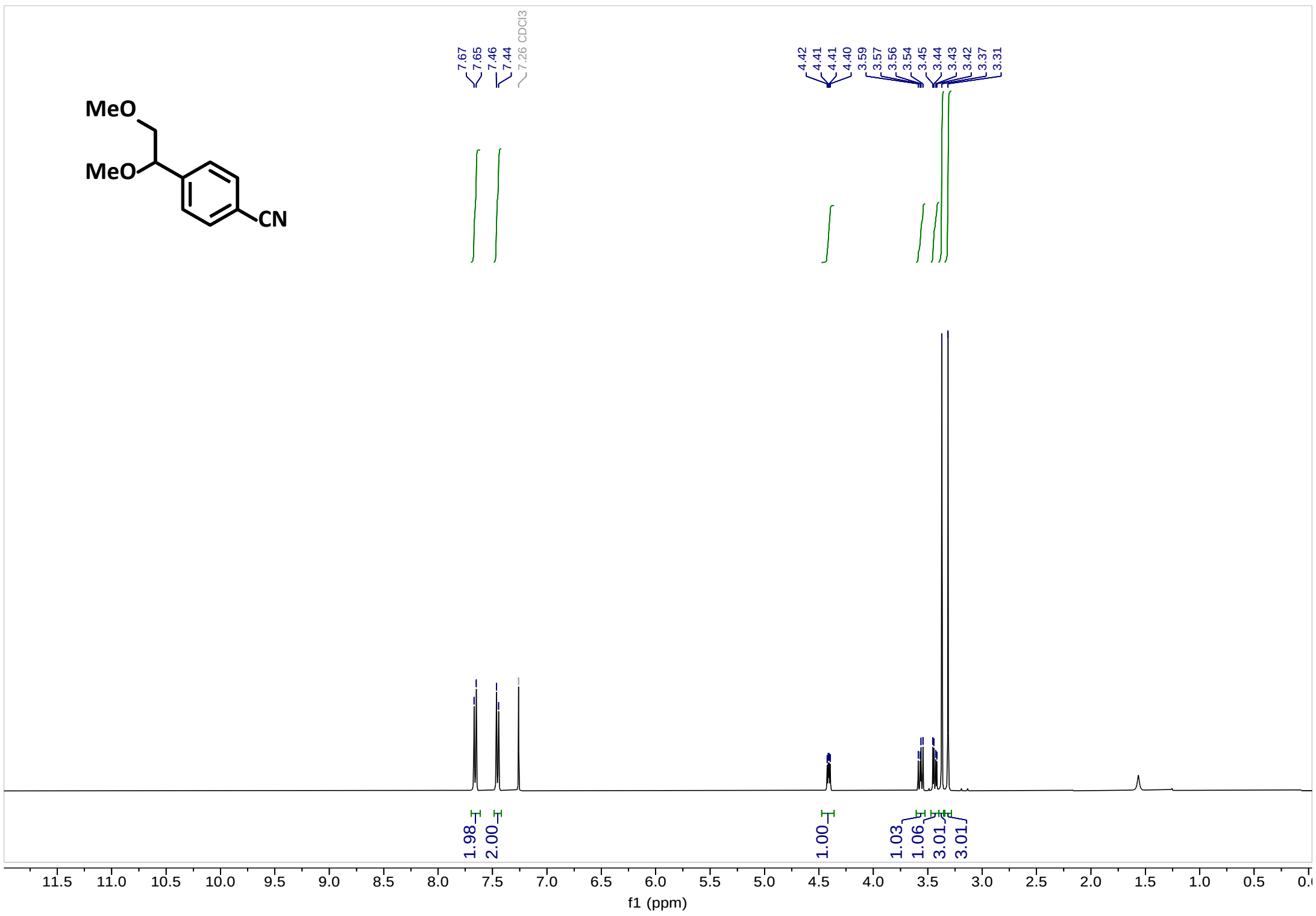

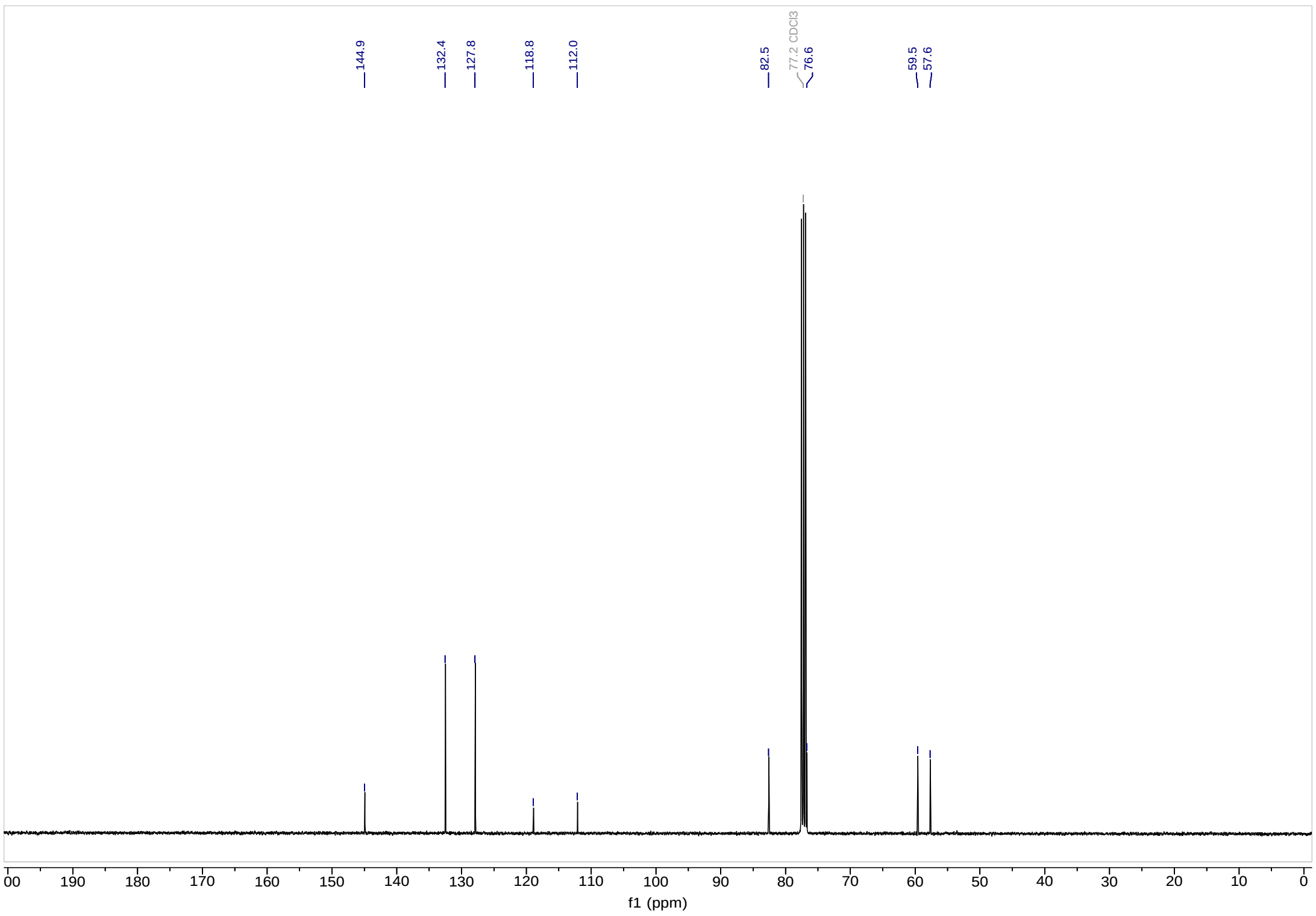

2-(4-(trifluoromethyl)phenyl)tetrahydrofuran (3ab)

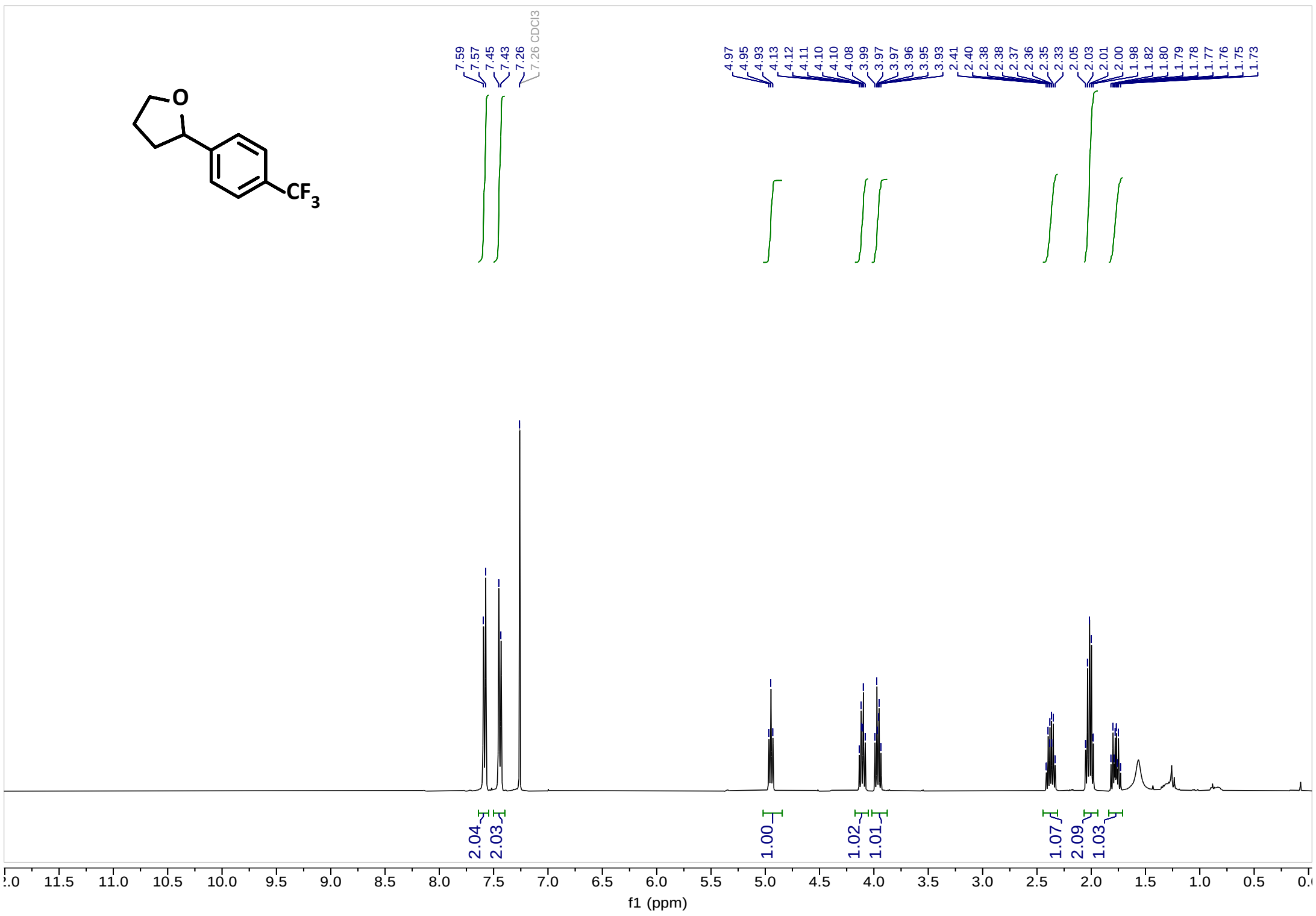

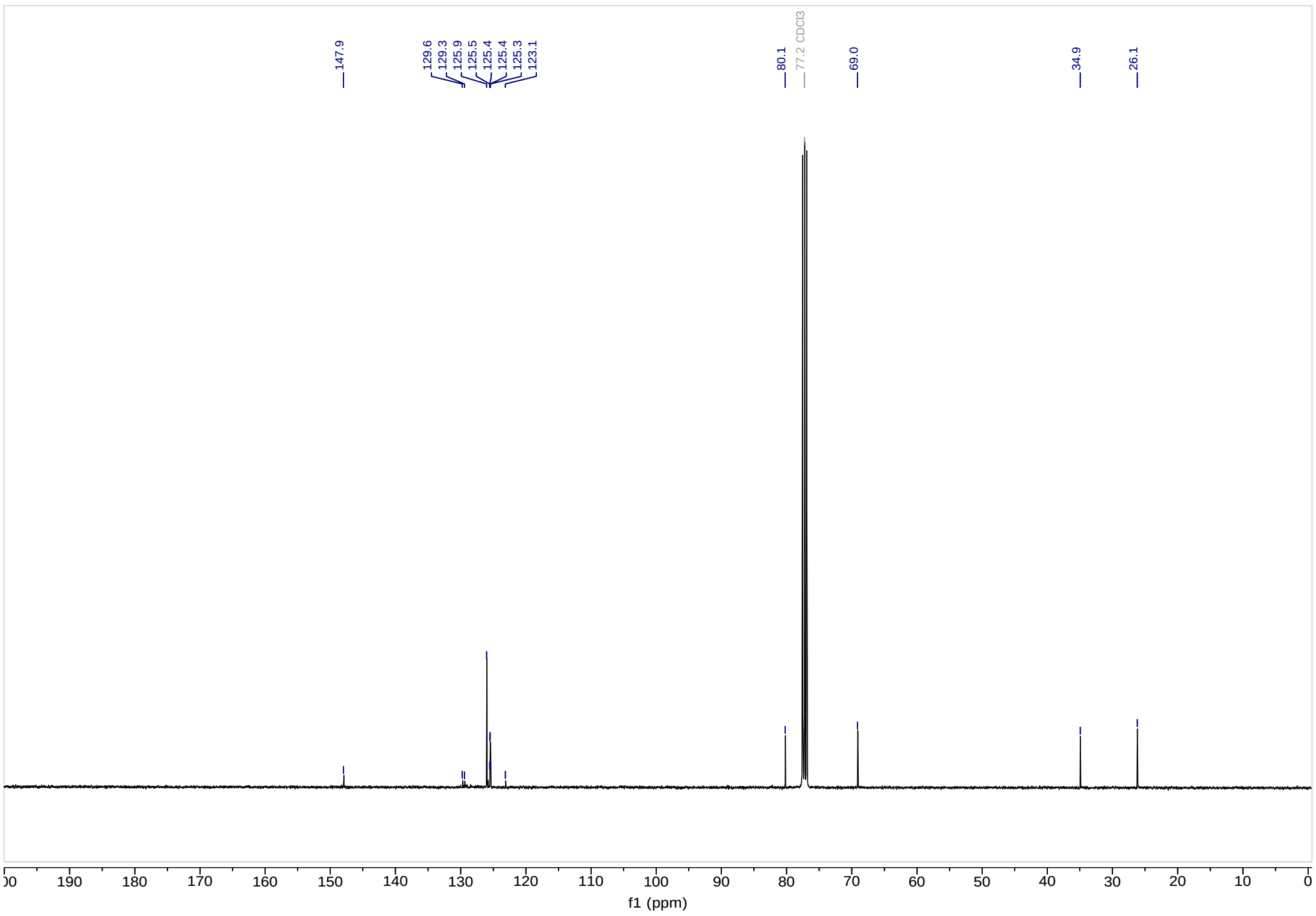

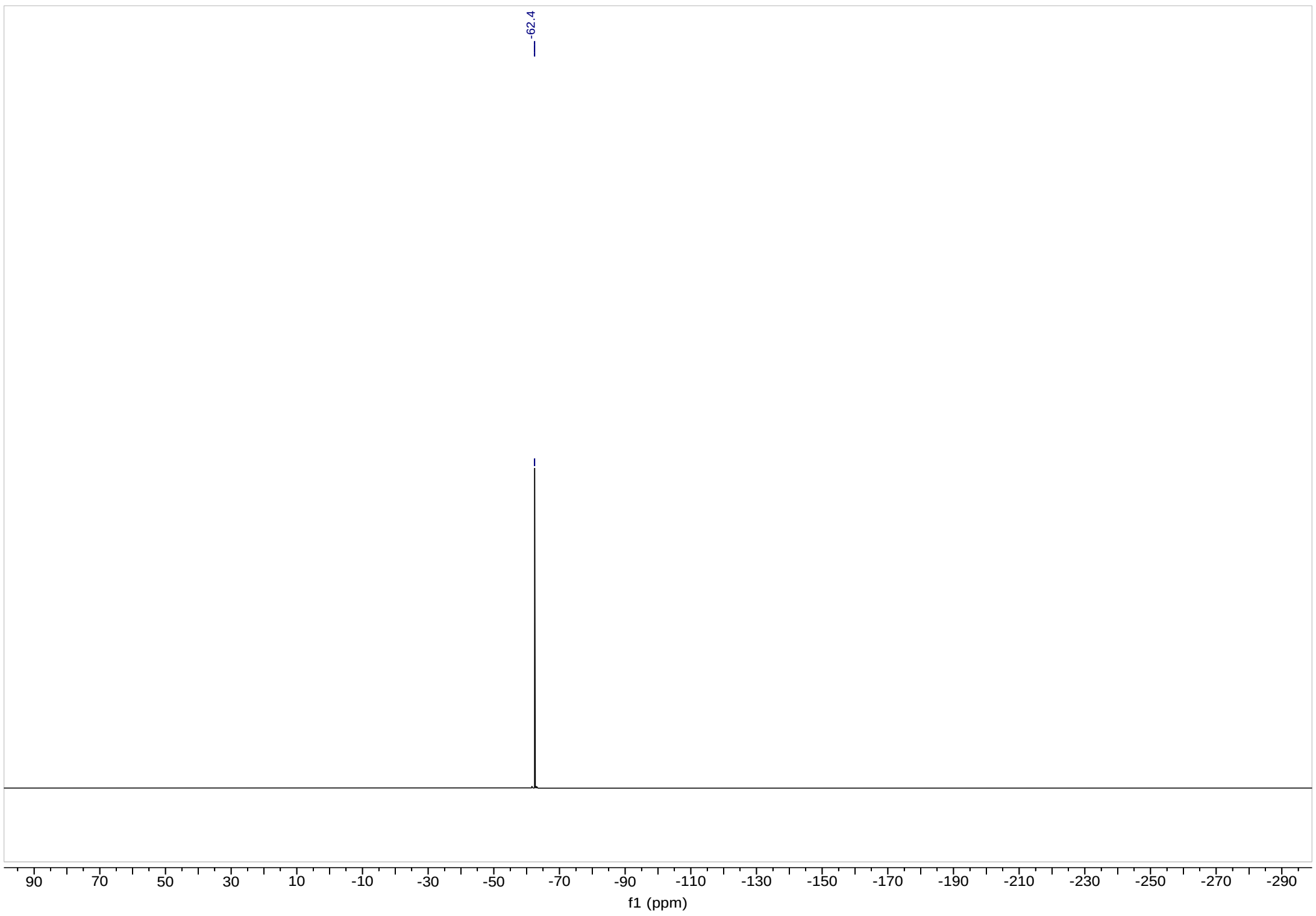
-62.4
90
70
50
30
10
-10
-30
-50
-70
-90
-110
-130
-150
-170
-190
-210
-230
-250
-270
-290
f1 (ppm)

methyl 4-(tetrahydrofuran-2-yl)benzoate (3ac)

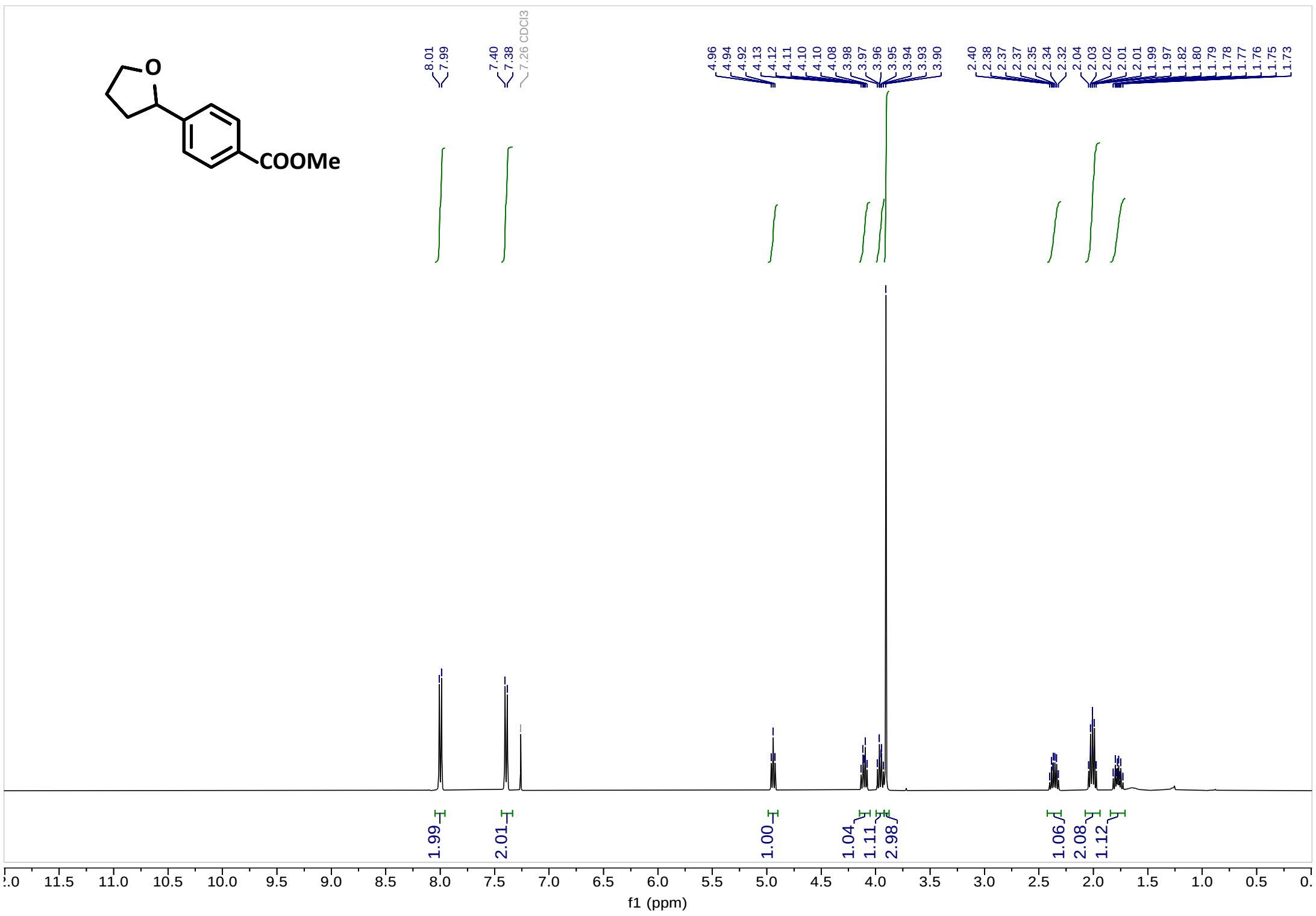

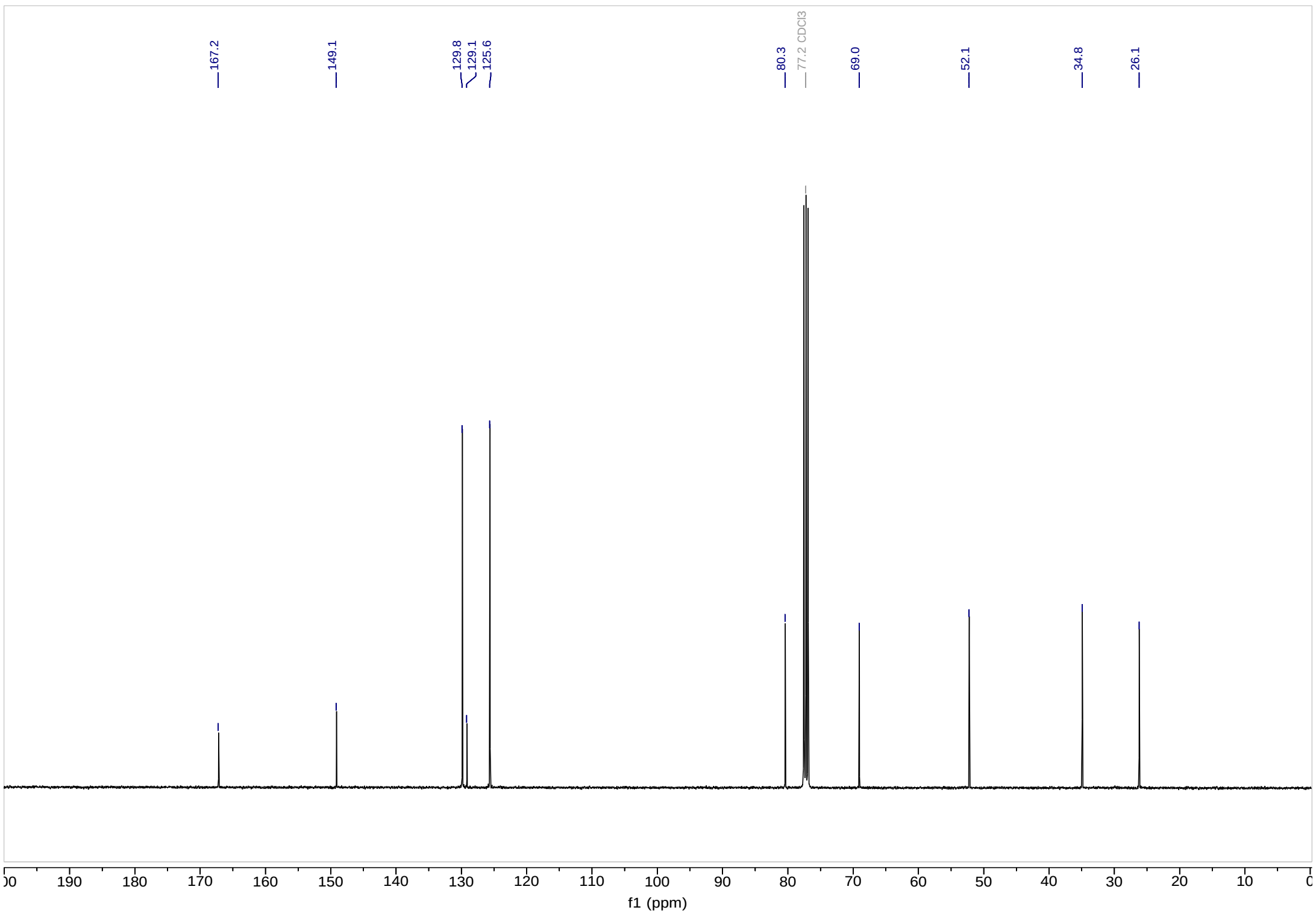

2-phenyltetrahydrofuran (3ad)

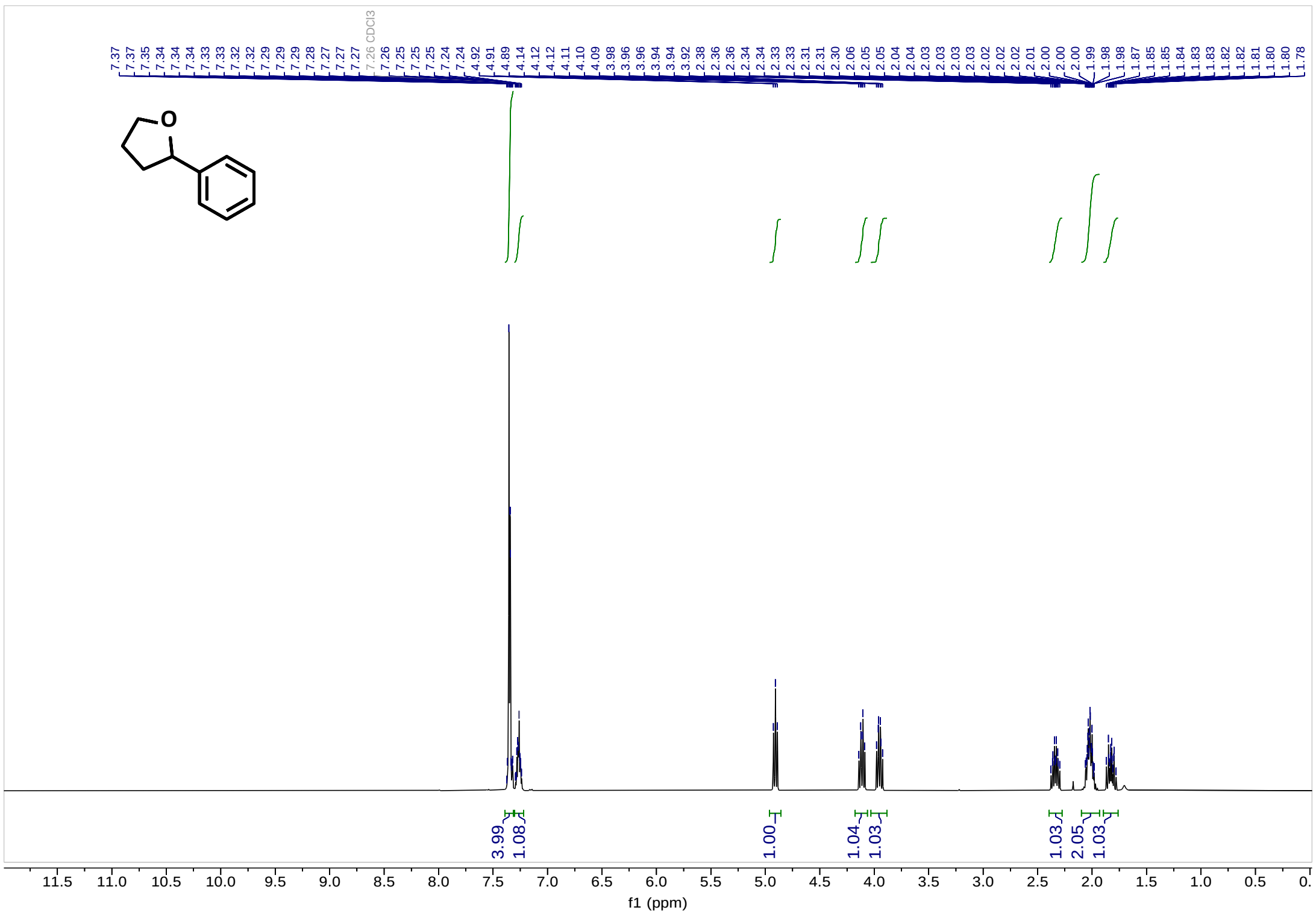

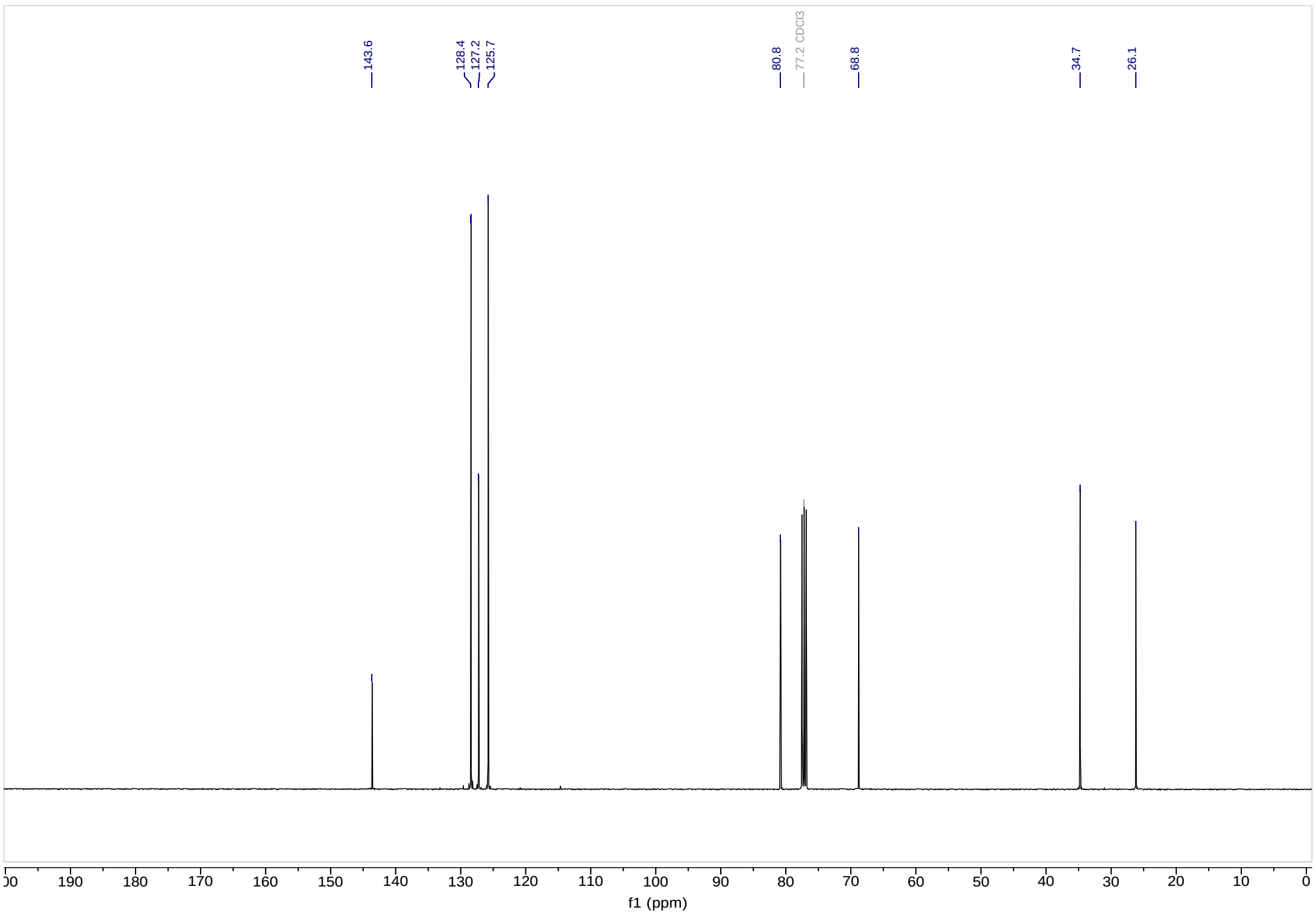

2-(4-fluorophenyl)tetrahydrofuran (3ae)

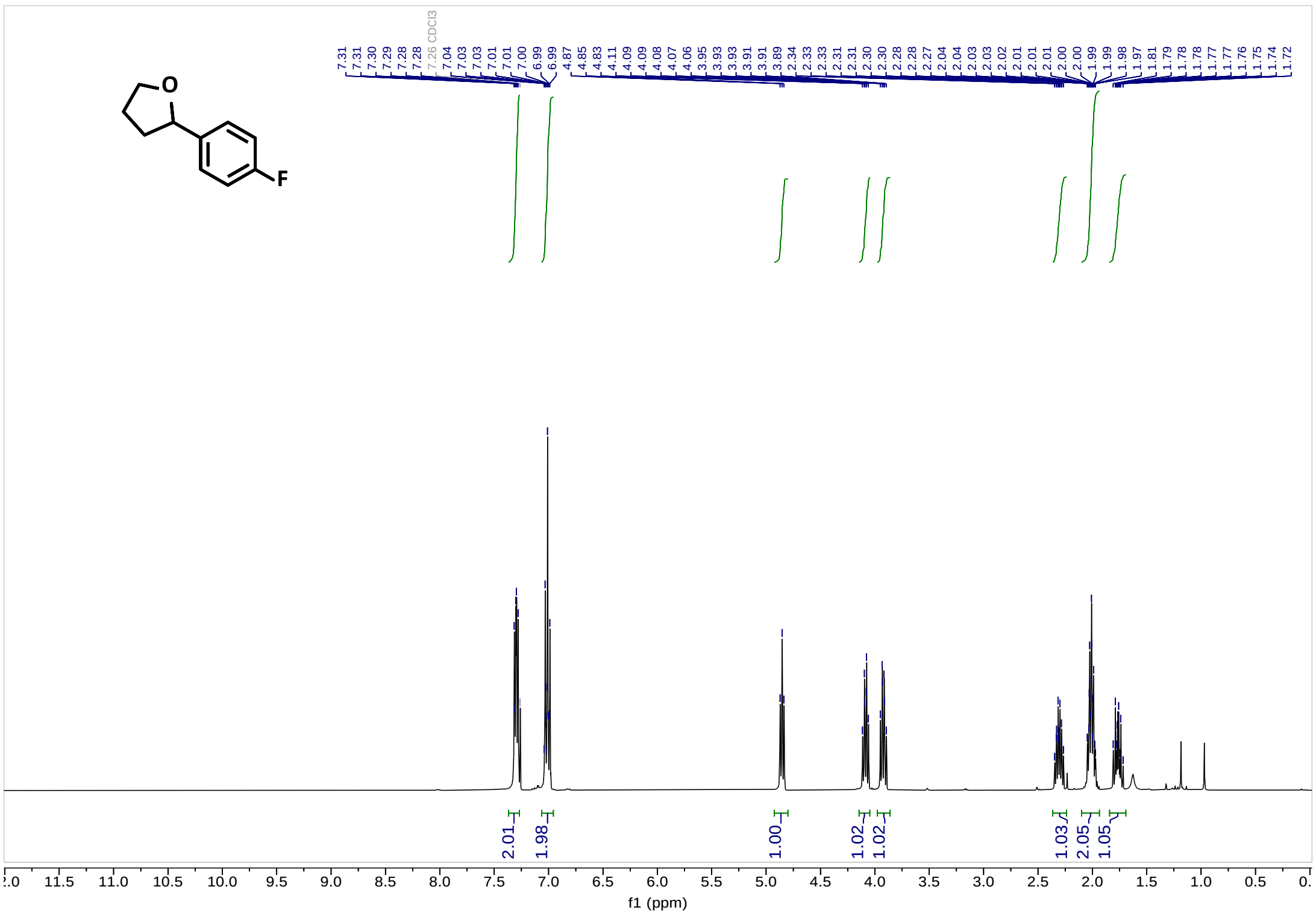

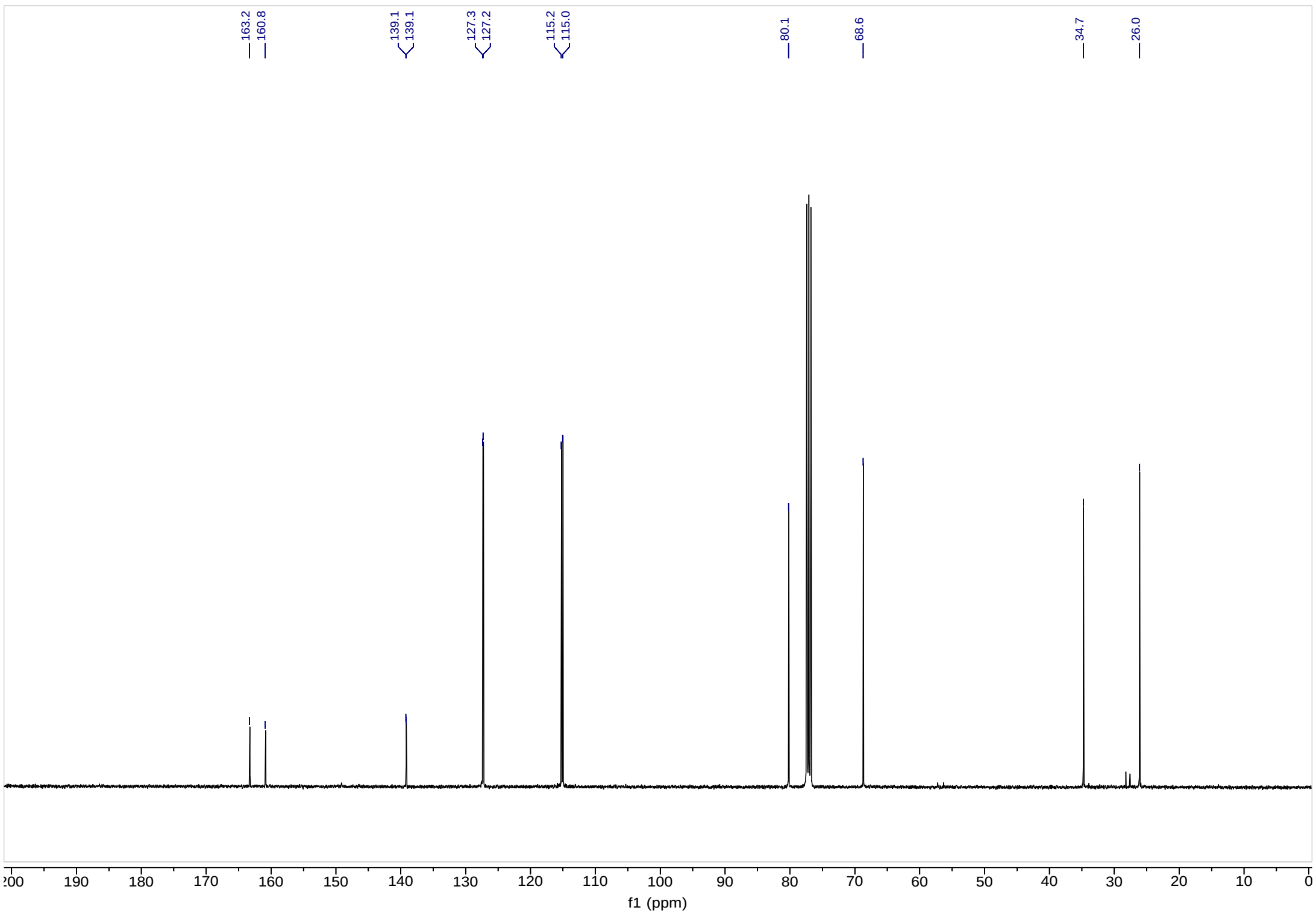

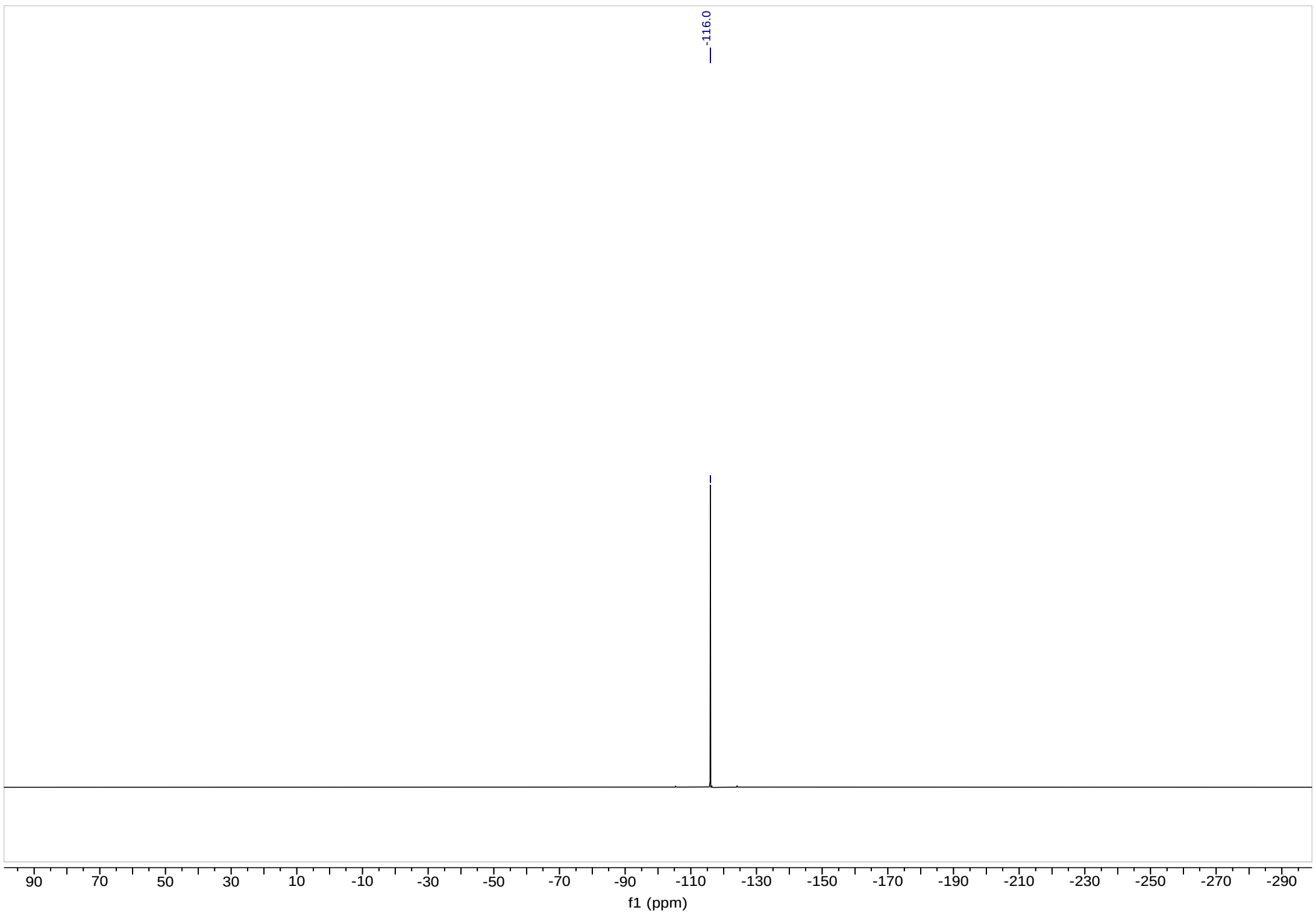
-116.0
90
70
50
30
10
-10
-30
-50
-70
-90
-110
-130
-150
-170
-190
-210
-230
-250
-270
-290
f1 (ppm)

2-(4-chlorophenyl)tetrahydrofuran (3af)

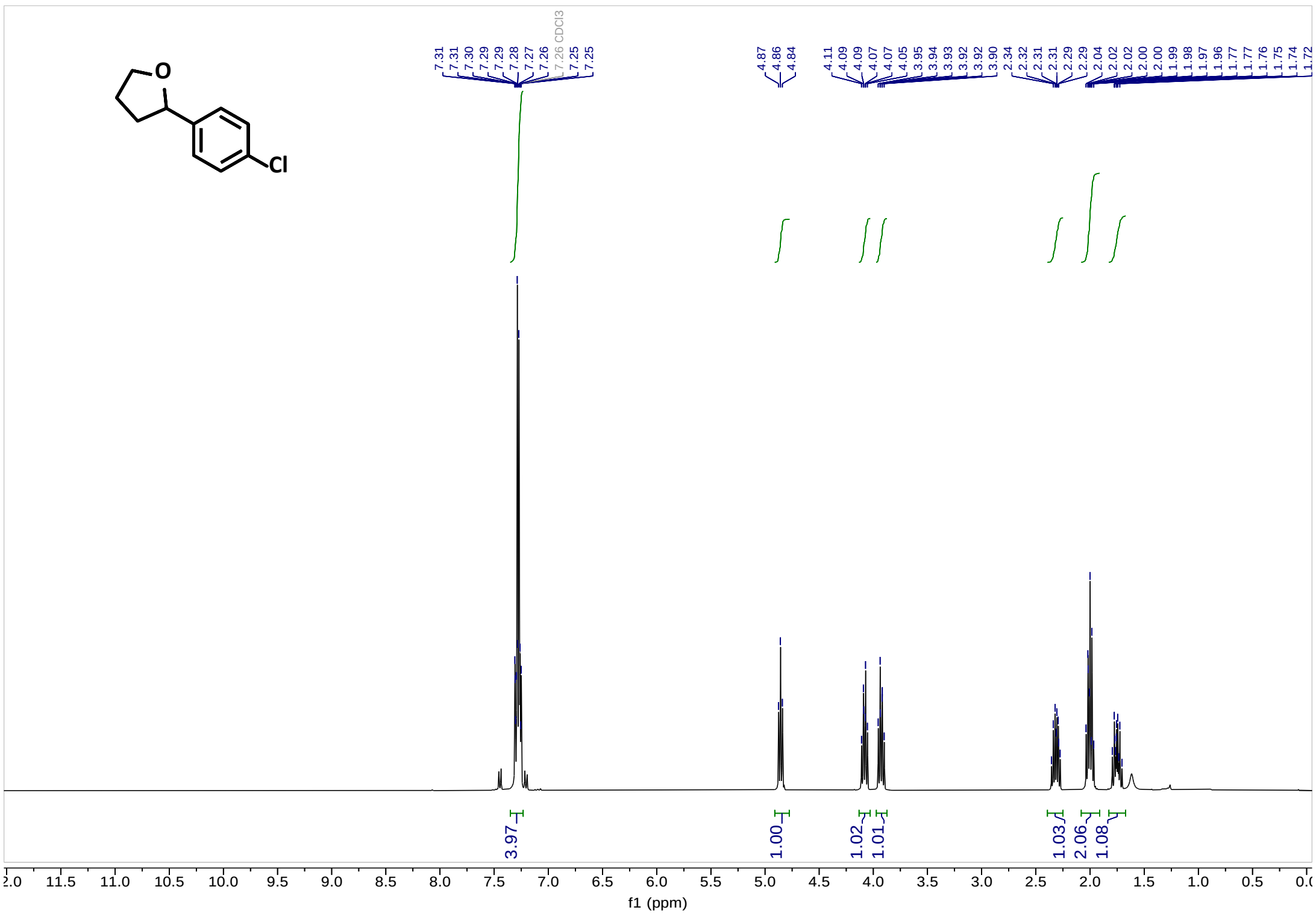

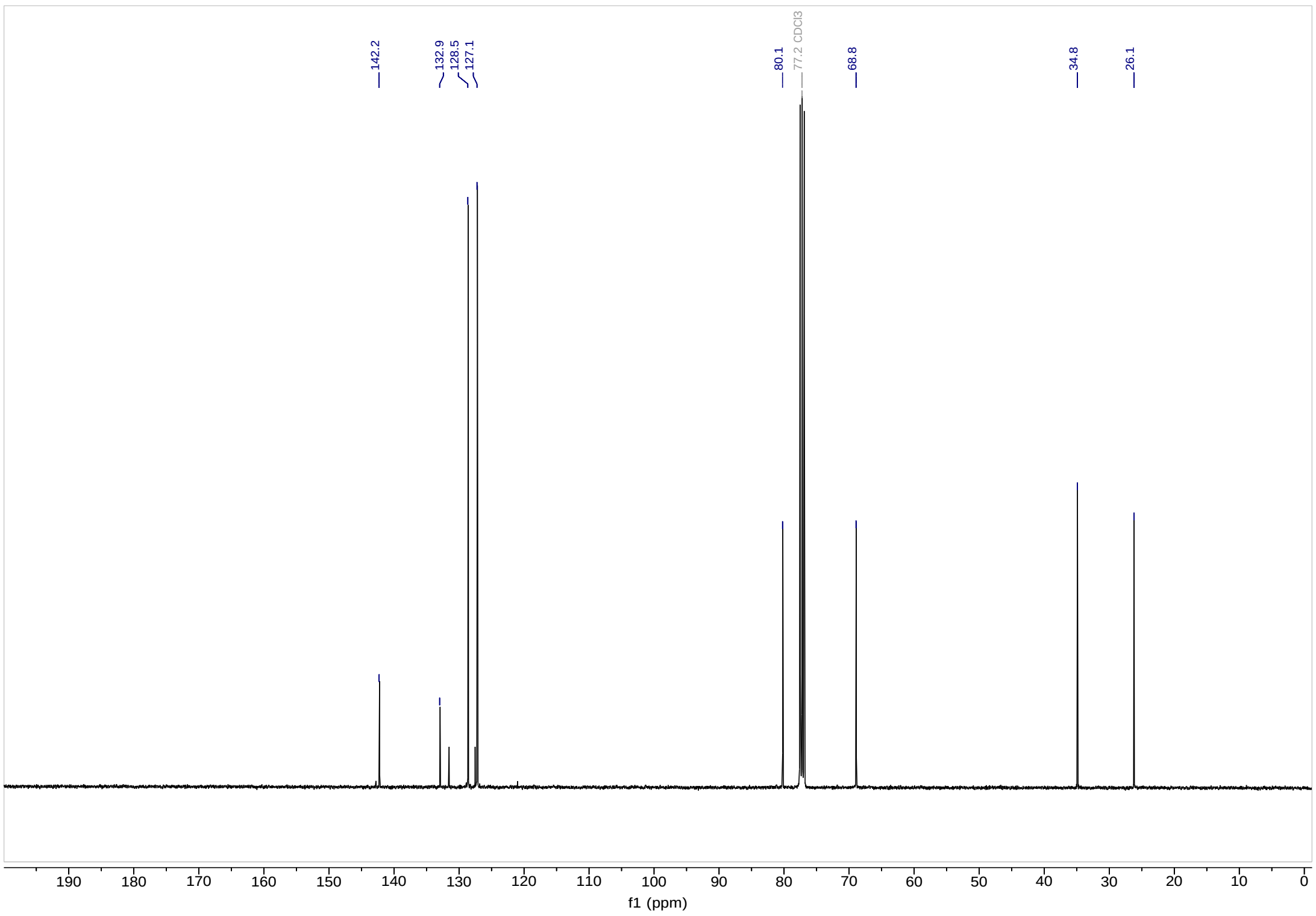

2-(4-bromophenyl)tetrahydrofuran (3ag)

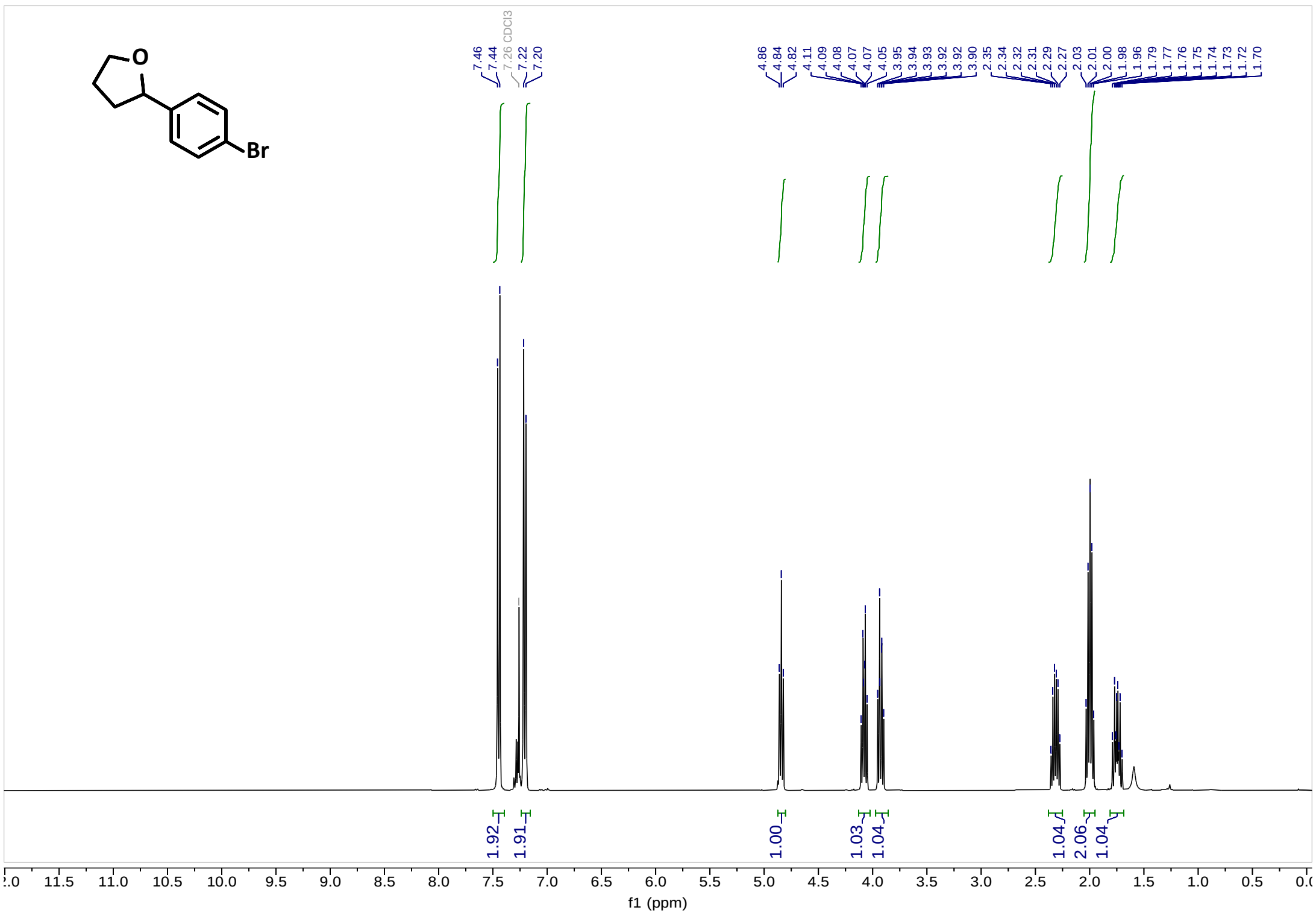

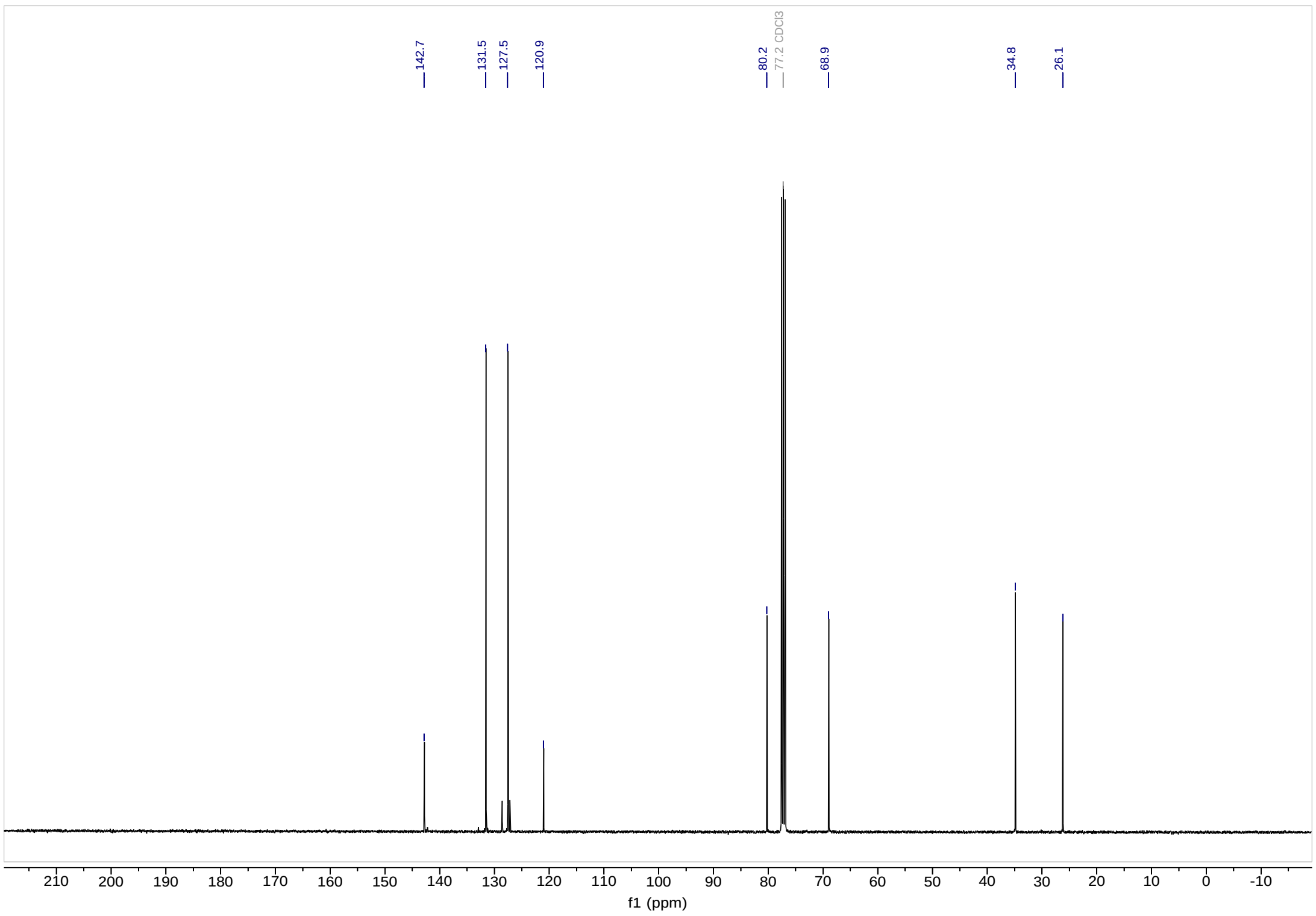

2-(4-methoxyphenyl)tetrahydrofuran (3ah)

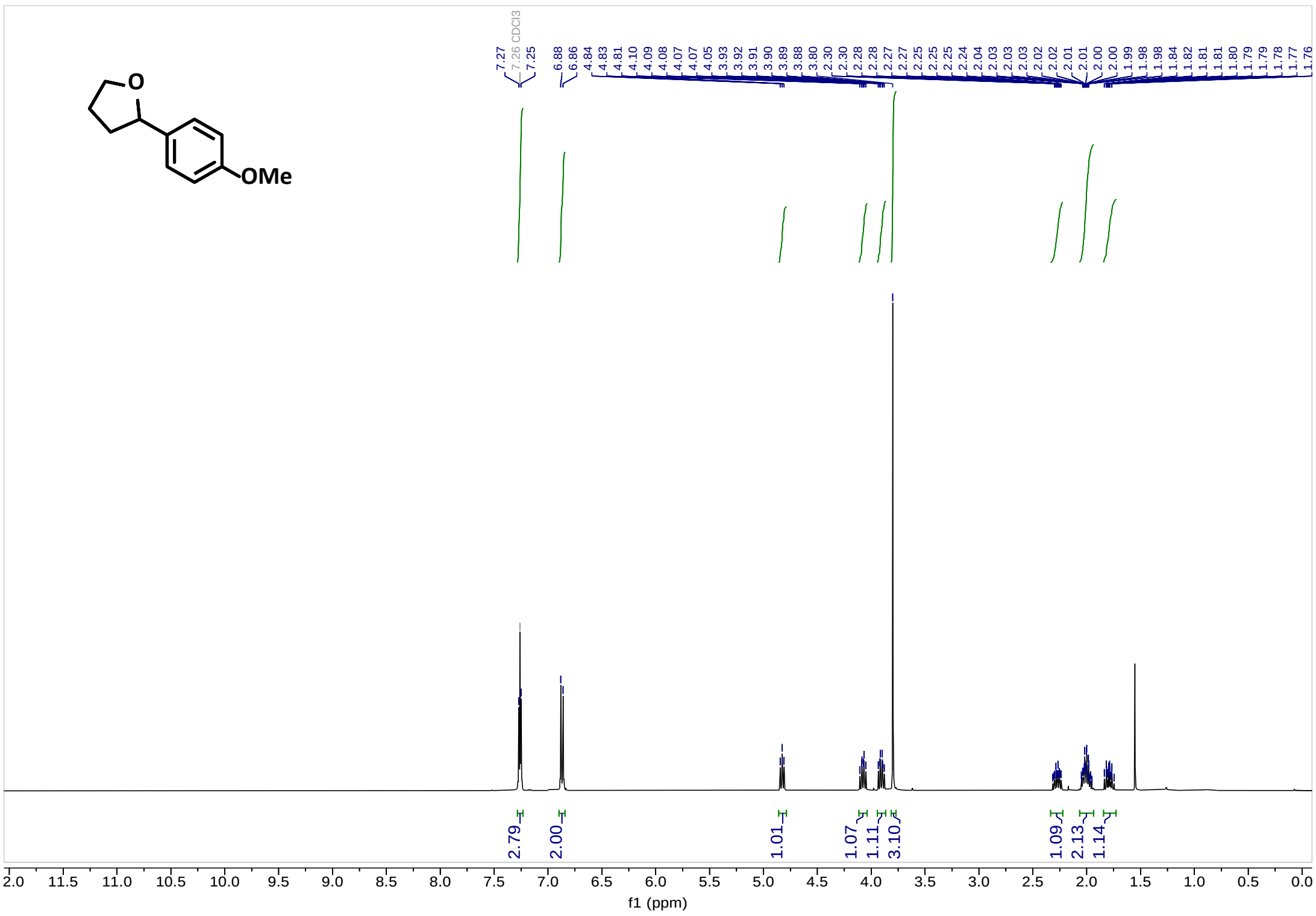

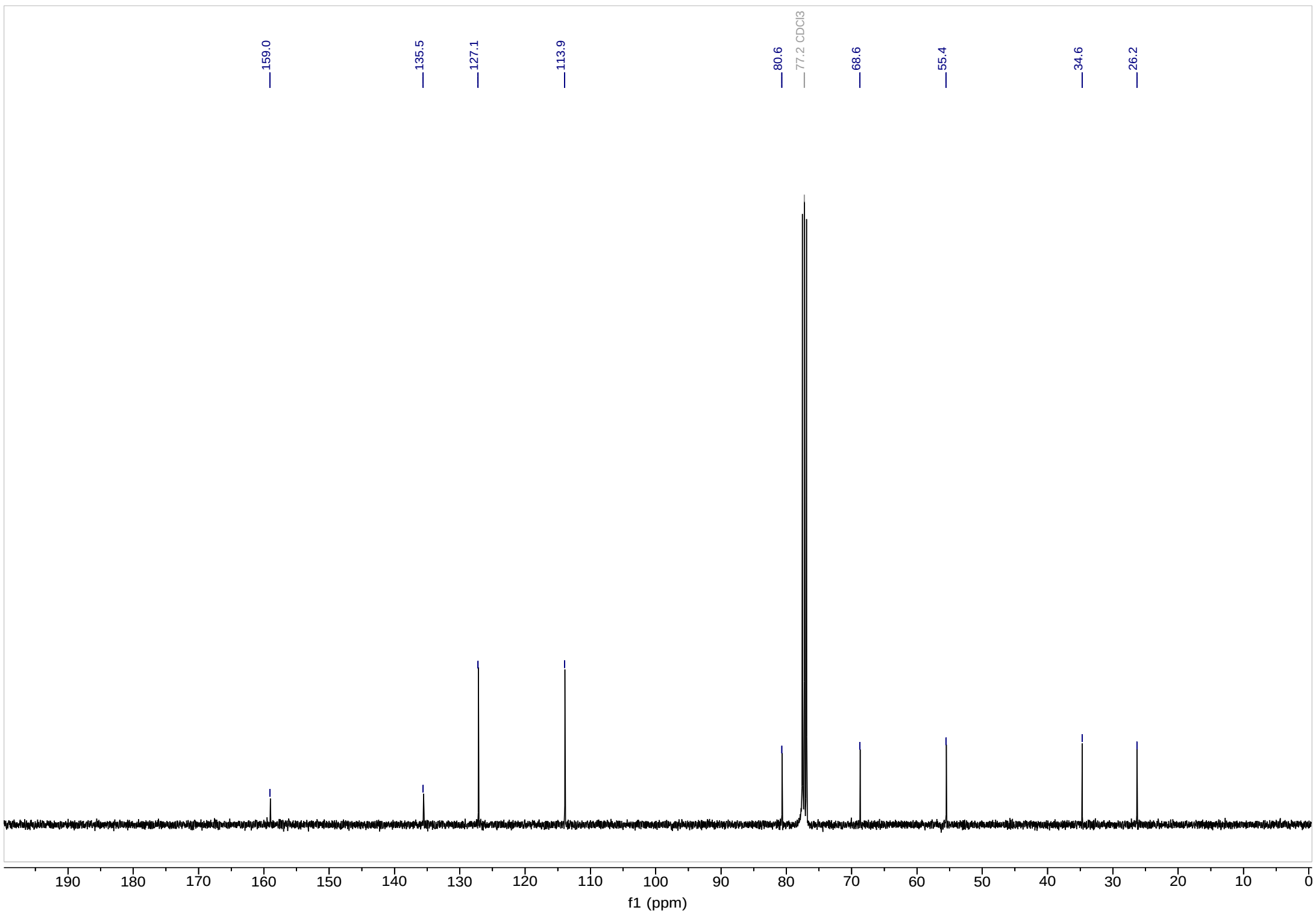

2-(4-tolyl)tetrahydrofuran (ai)

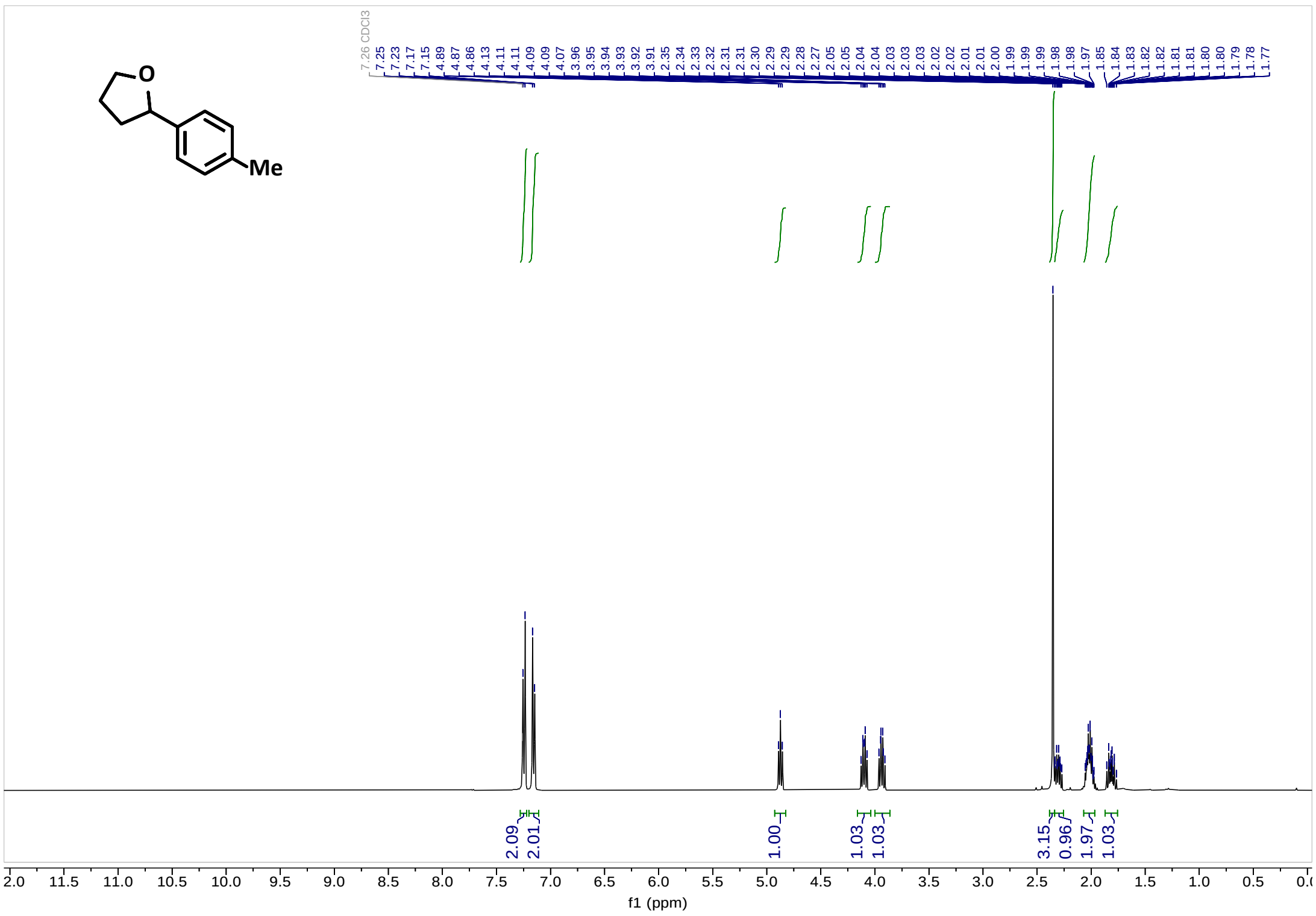

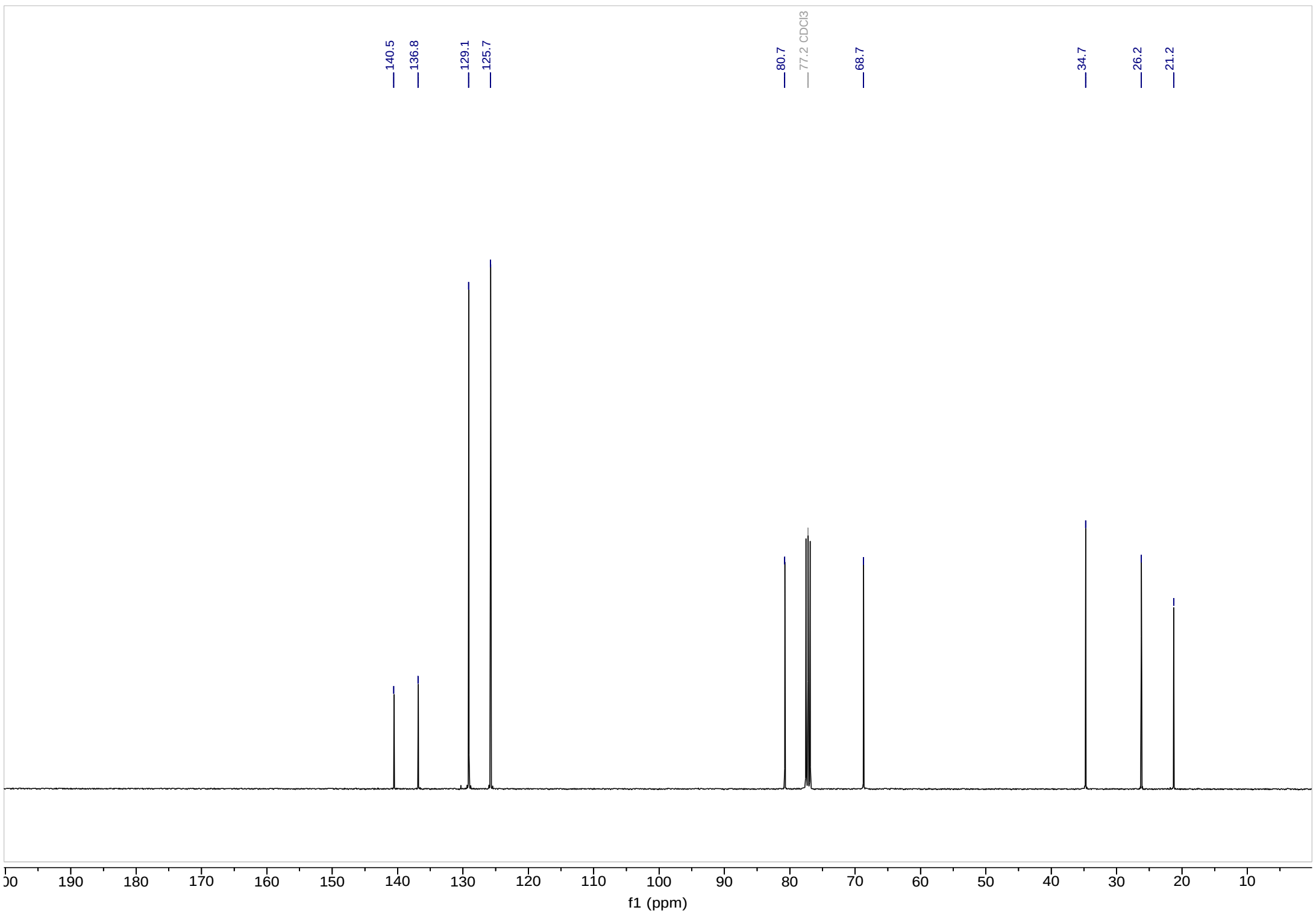

2-(4-(*tert*-butyl)phenyl)tetrahydrofuran (3aj)

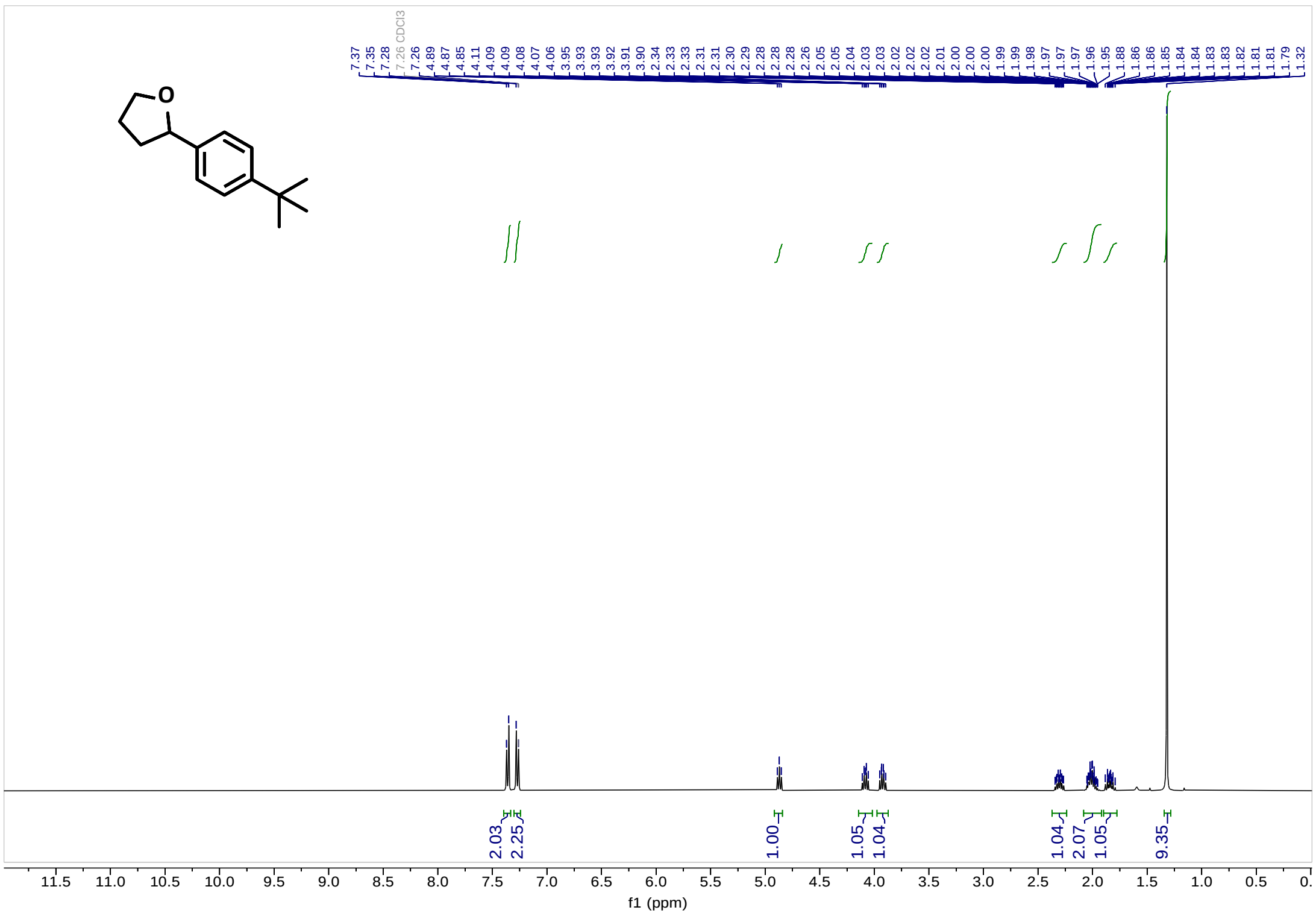

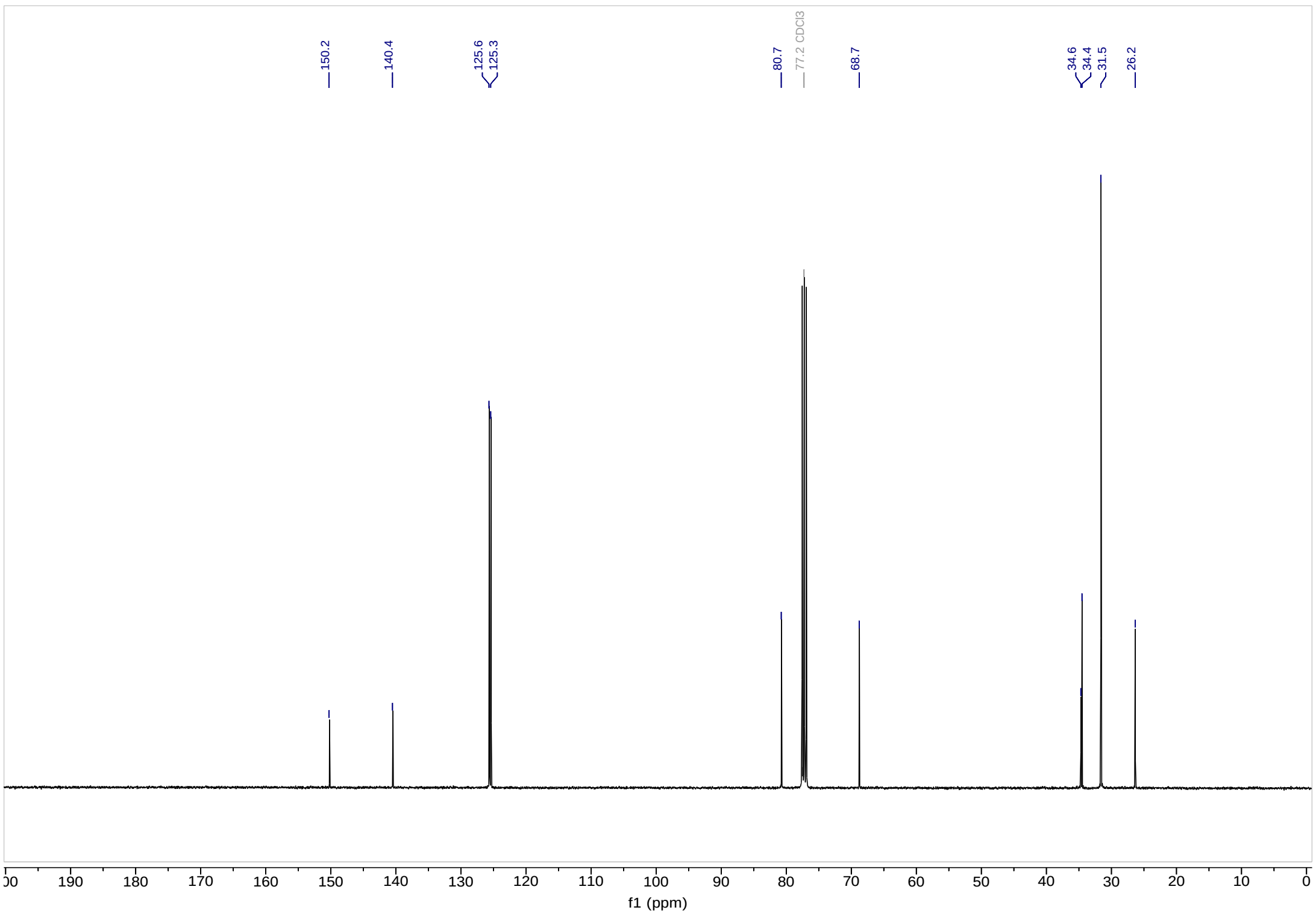

3-methyl-4-(tetrahydrofuran-2-yl)benzonitrile (3ak)

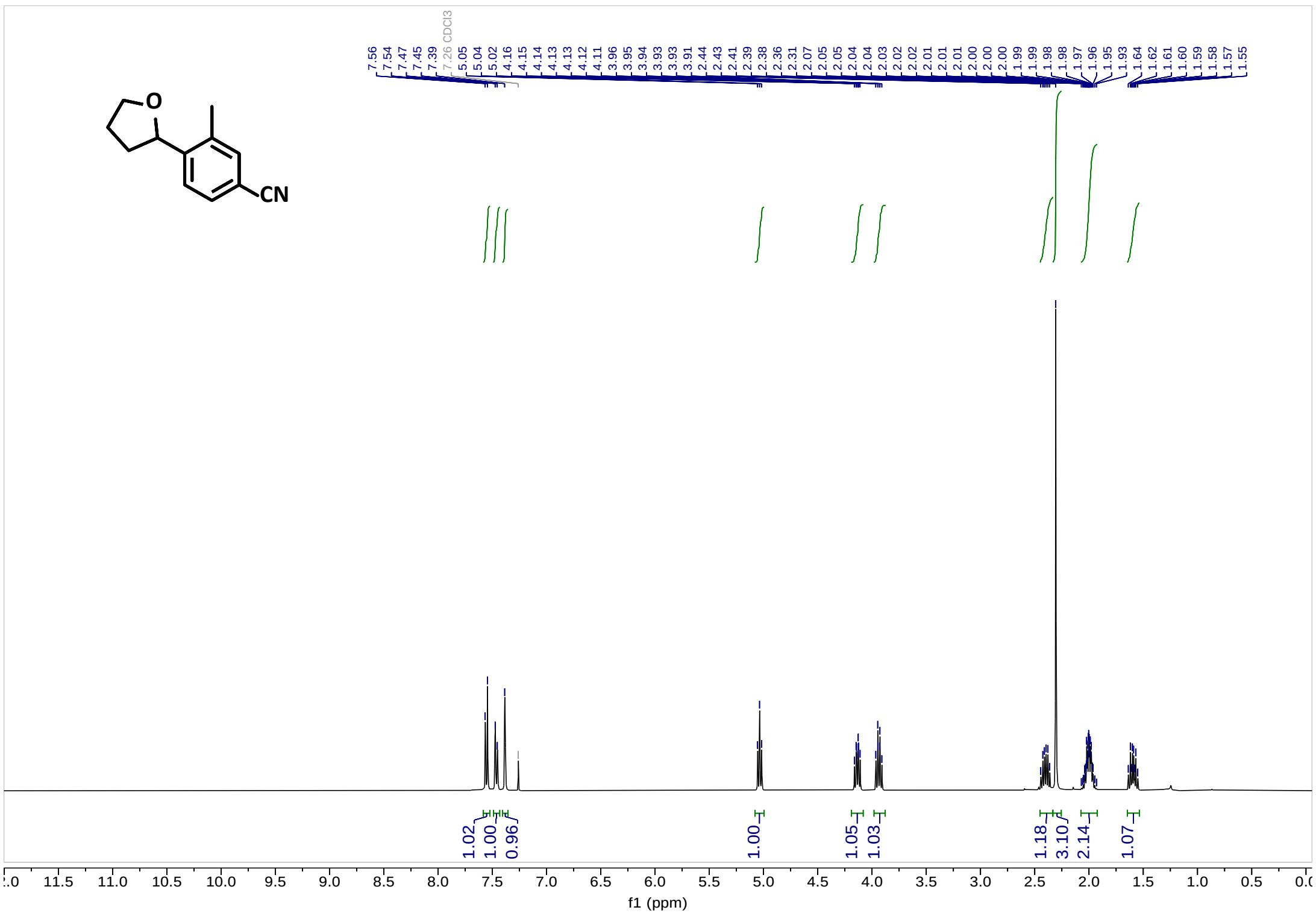

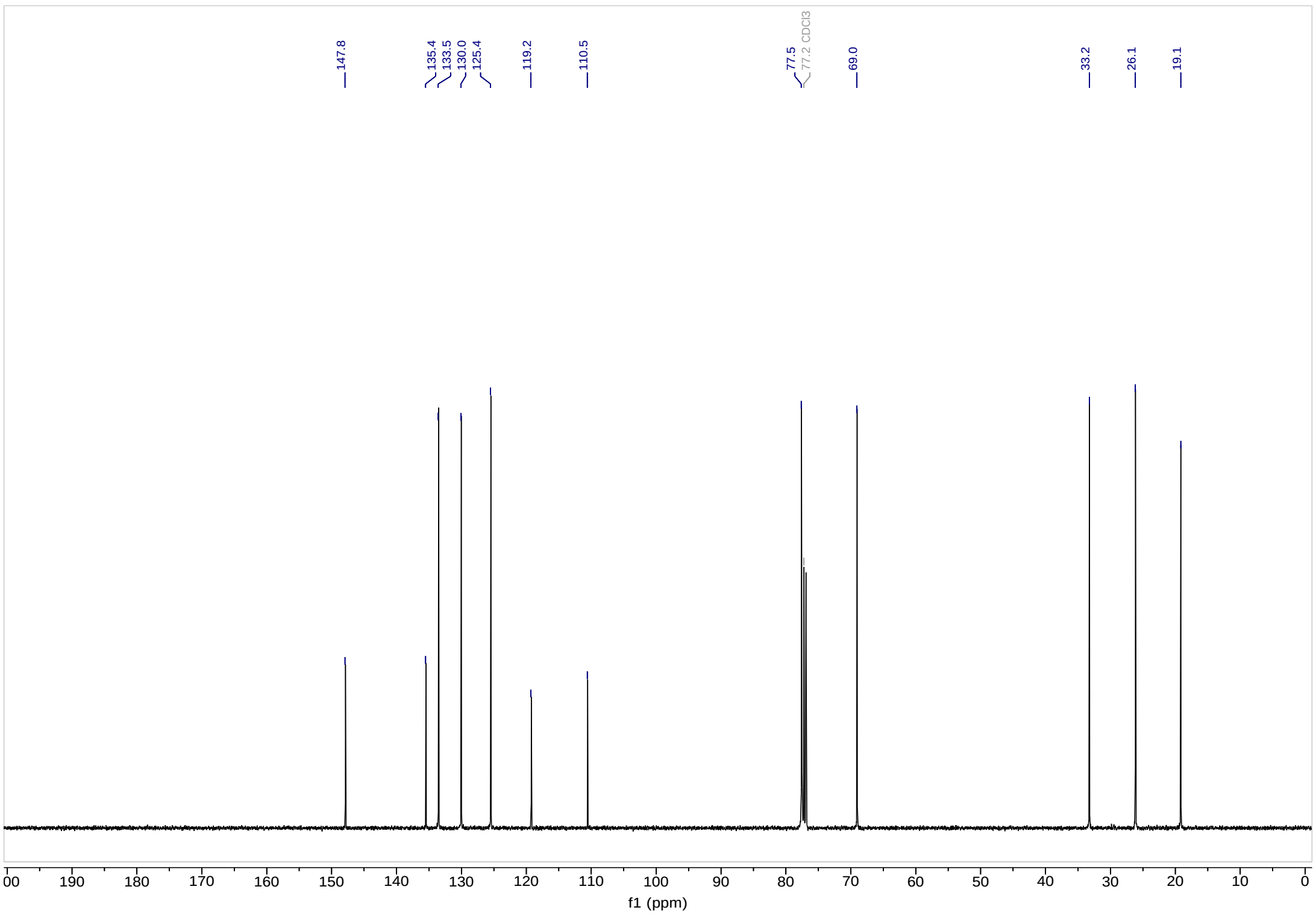

2-(3-phenylpropyl)tetrahydrofuran (3am)

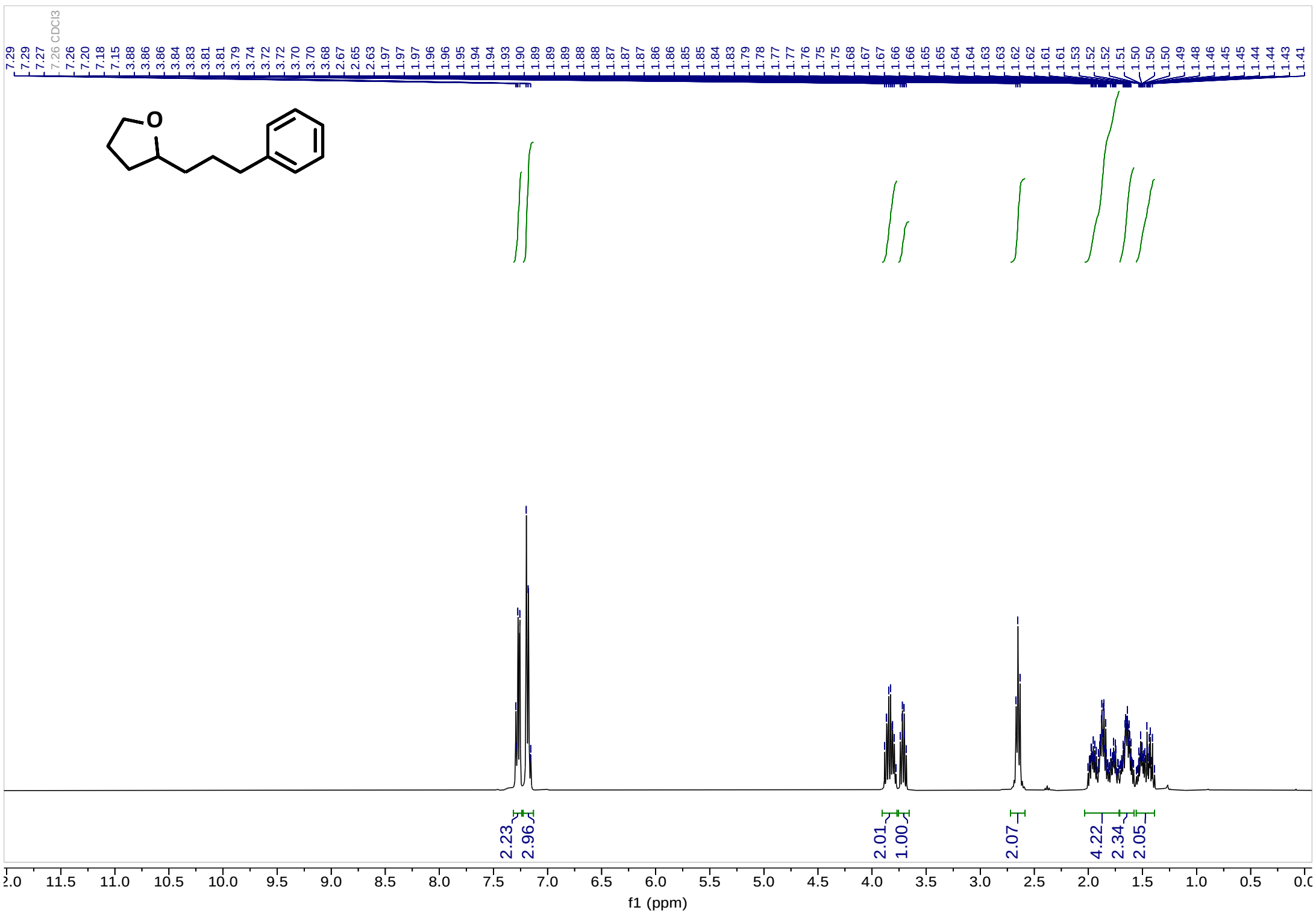

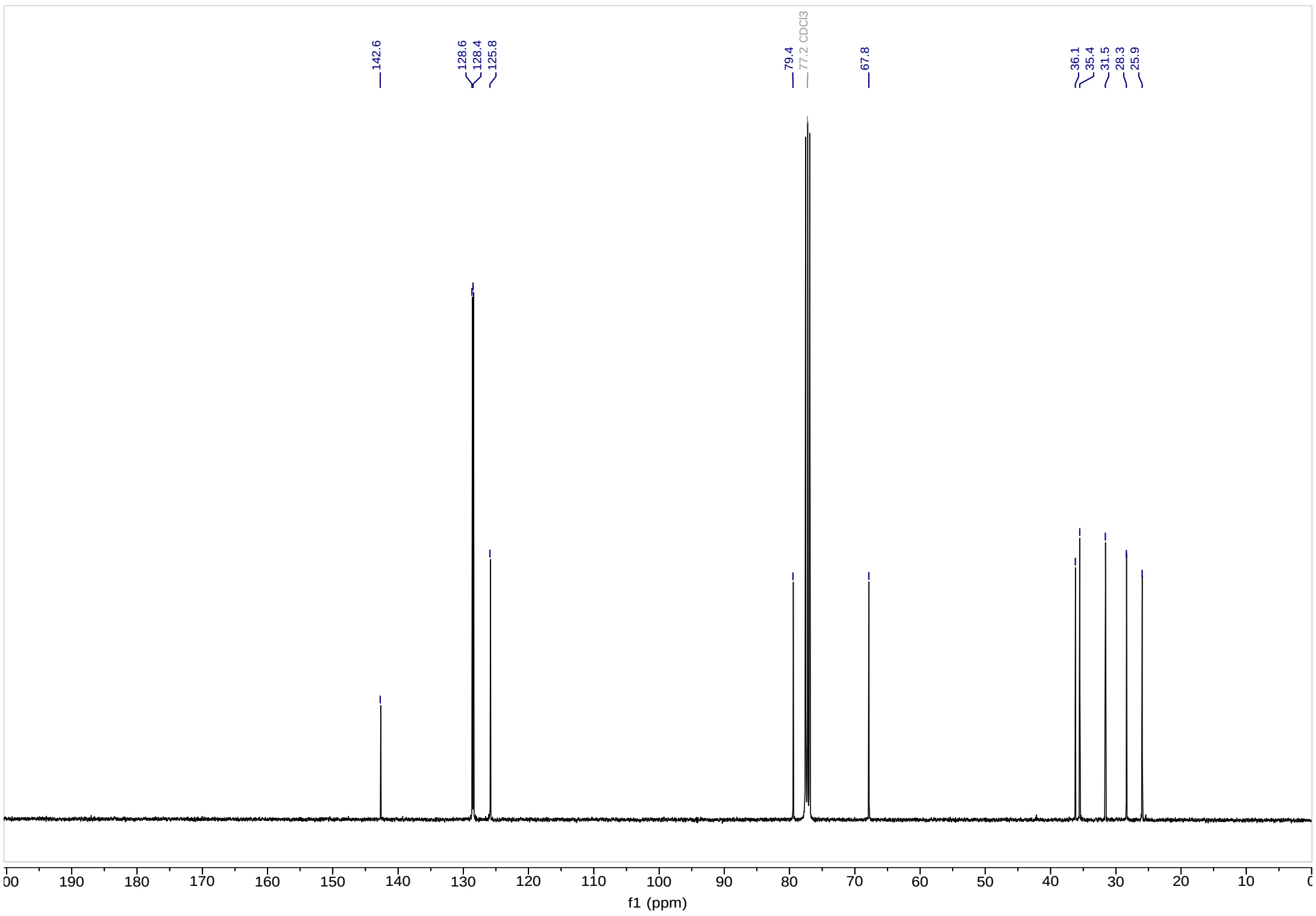

_N_-methyl-_N_-(4-phenylbutyl)acetamide (3fm)

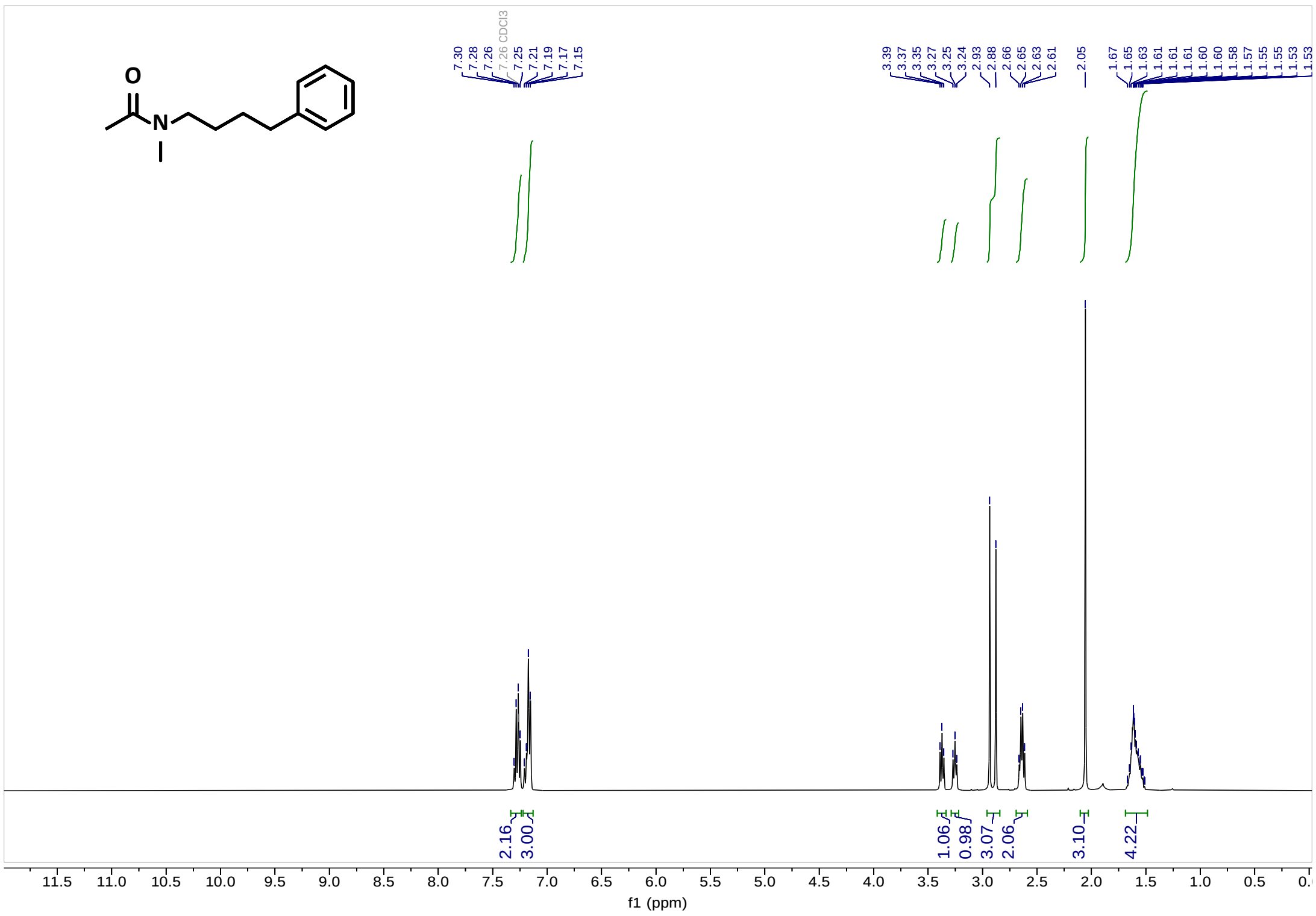

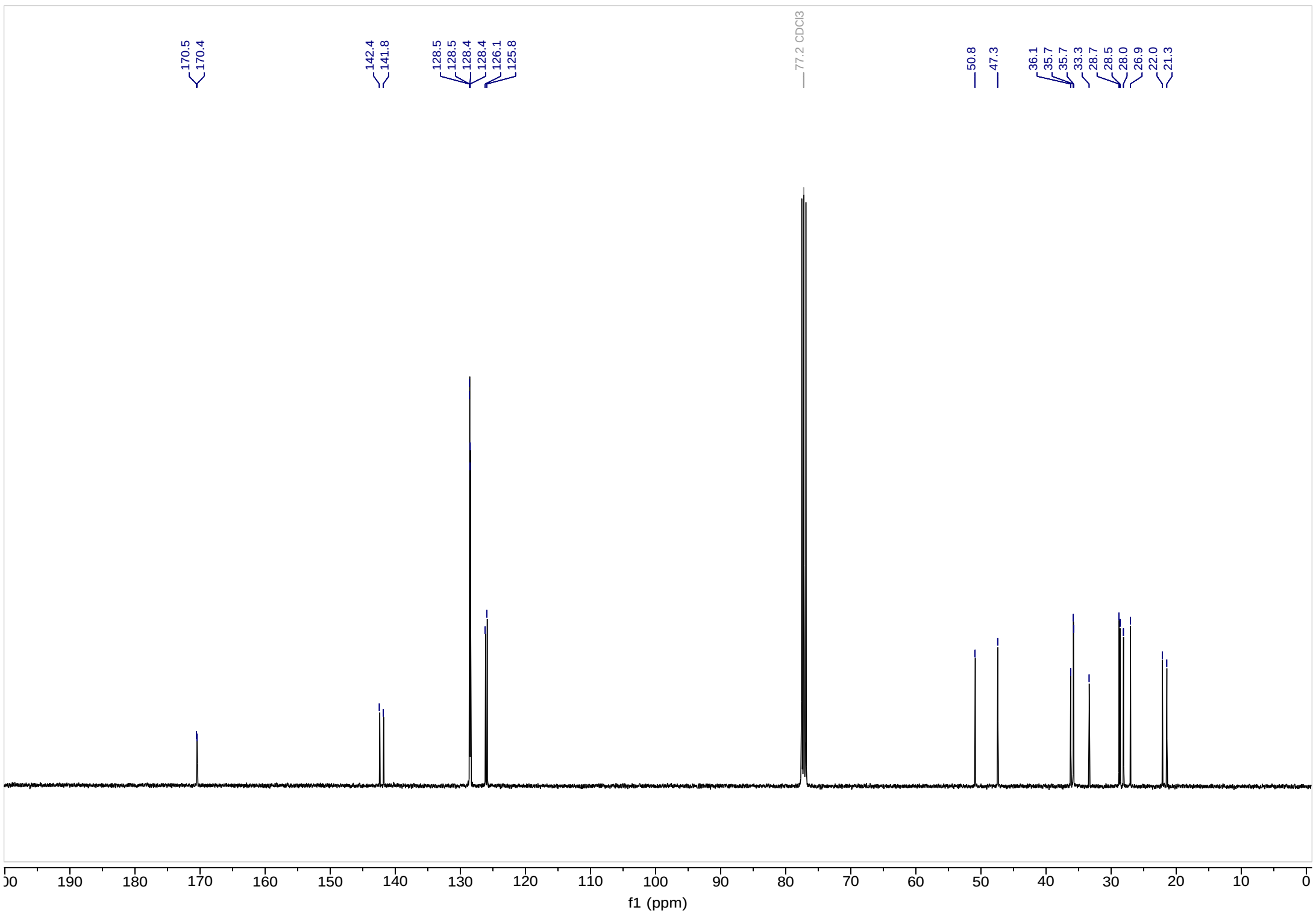

(4-(2-methoxyethoxy)butyl)benzene (3jm)

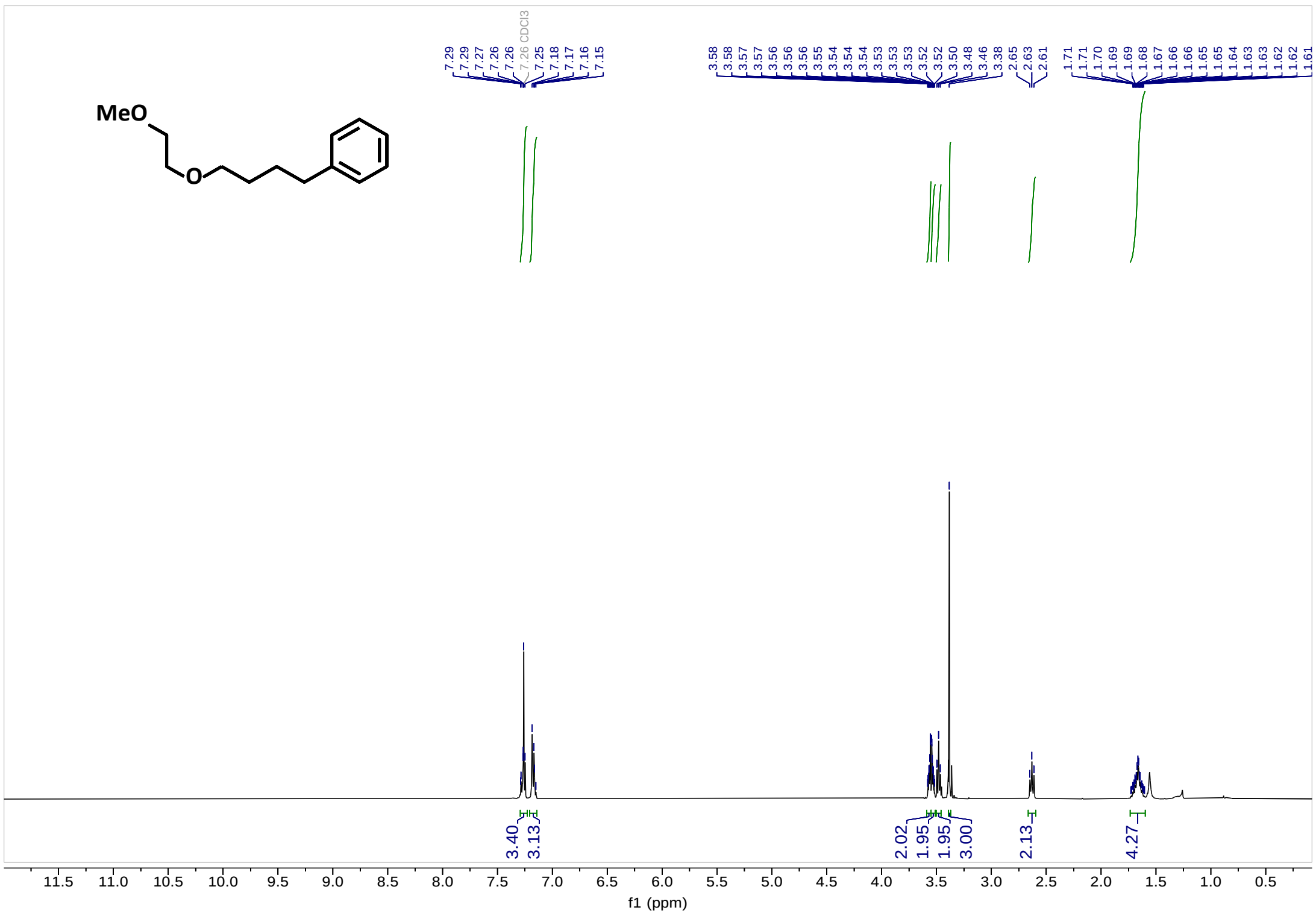

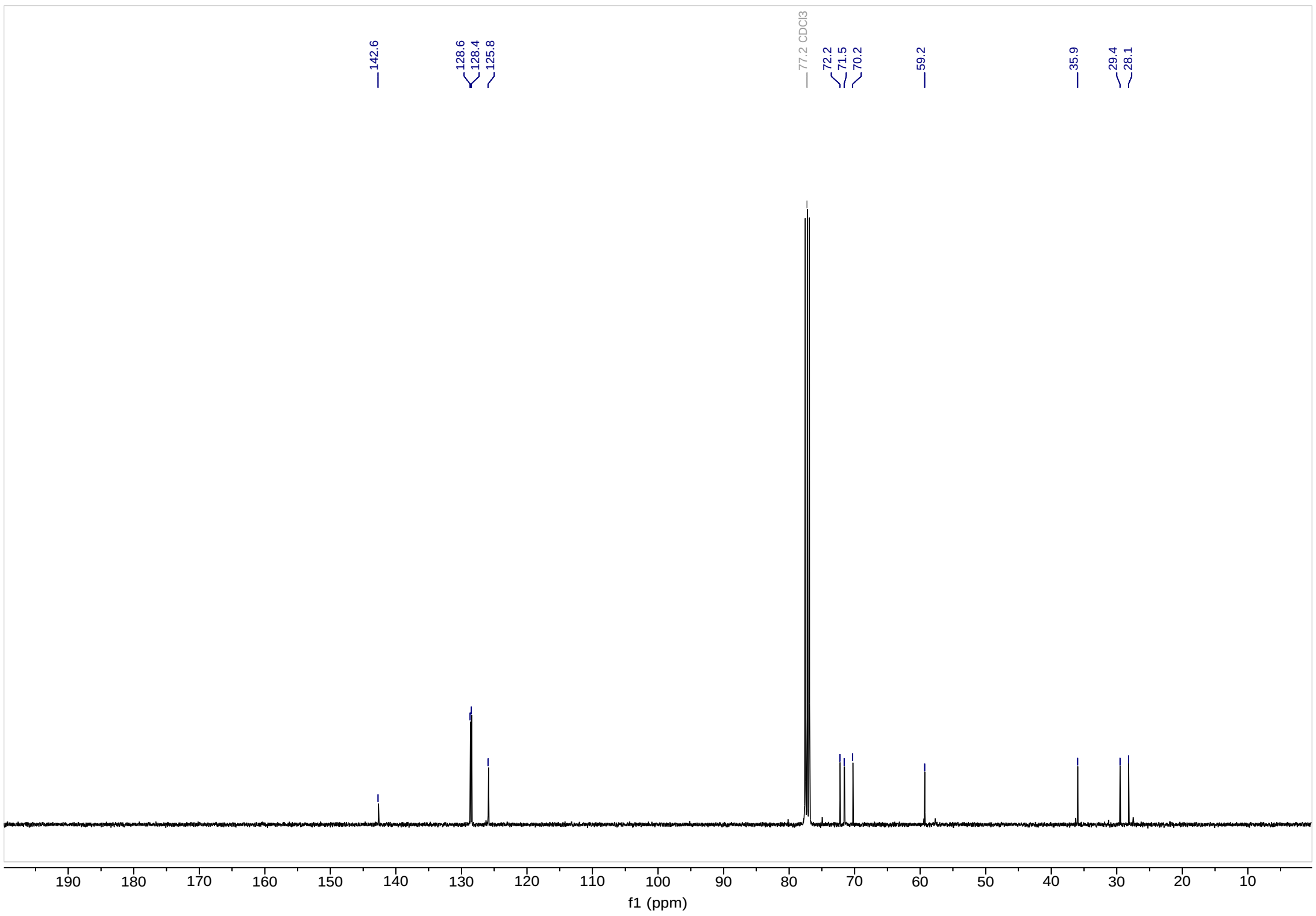

ethyl 4-(9-(tetrahydrofuran-2-yl)-5,6-dihydro-11H-benzo[5,6]cyclohepta[1,2-b]pyridin-11-ylidene) piperidine-1-carboxylate (3an)

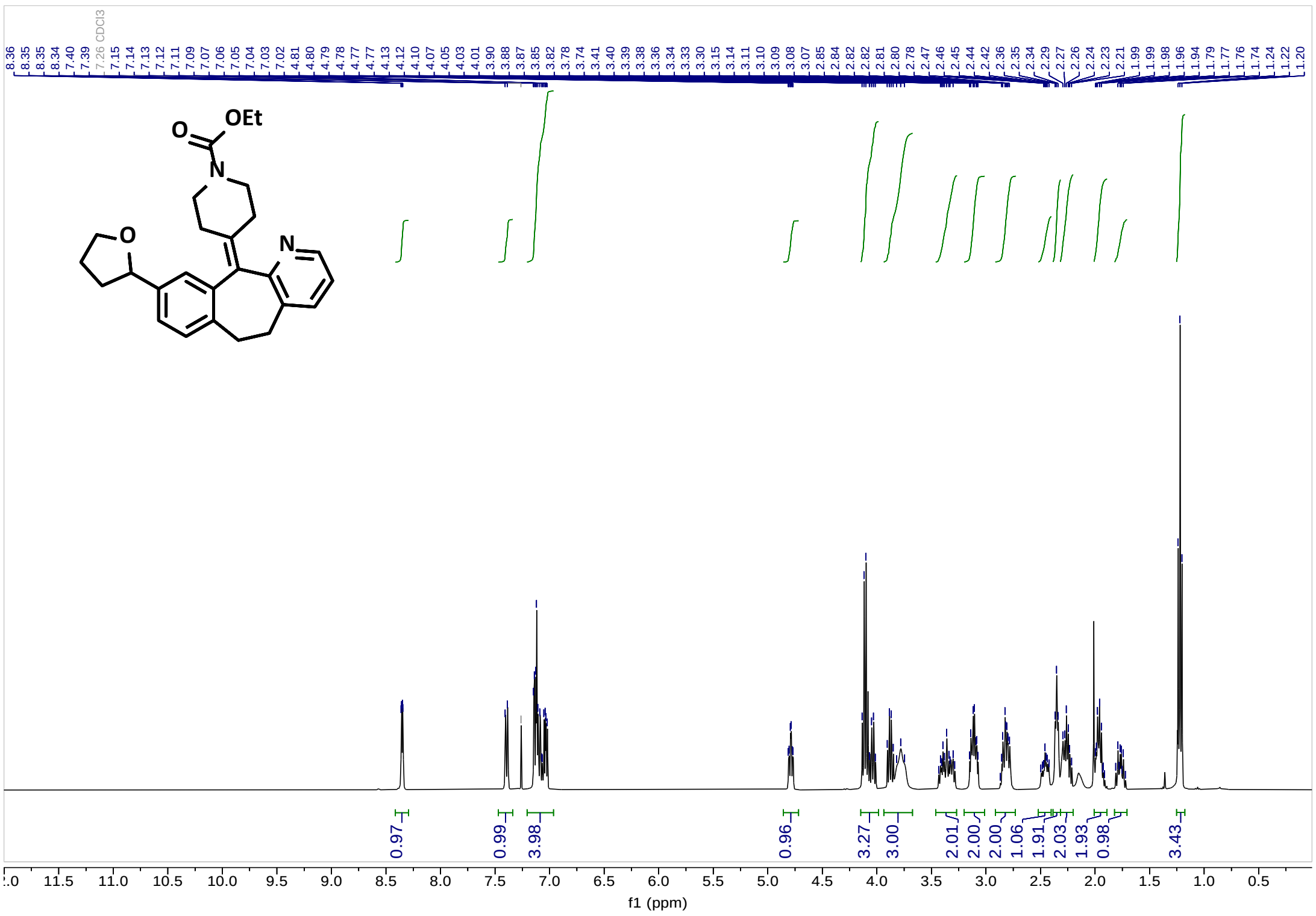

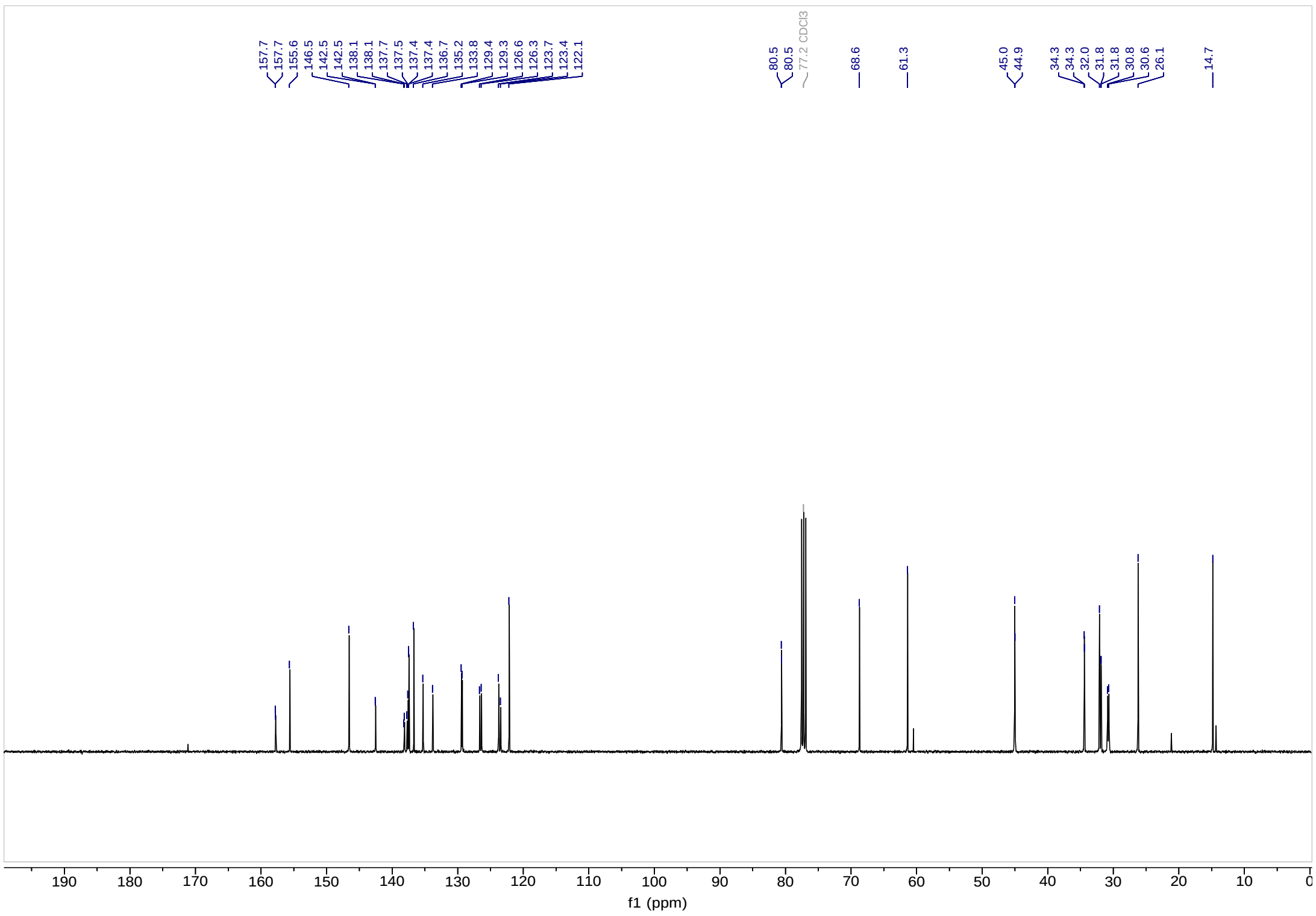

1,3,7-trimethyl-8-(tetrahydrofuran-2-yl)-3,7-dihydro-1H-purine-2,6-dione (3ao)

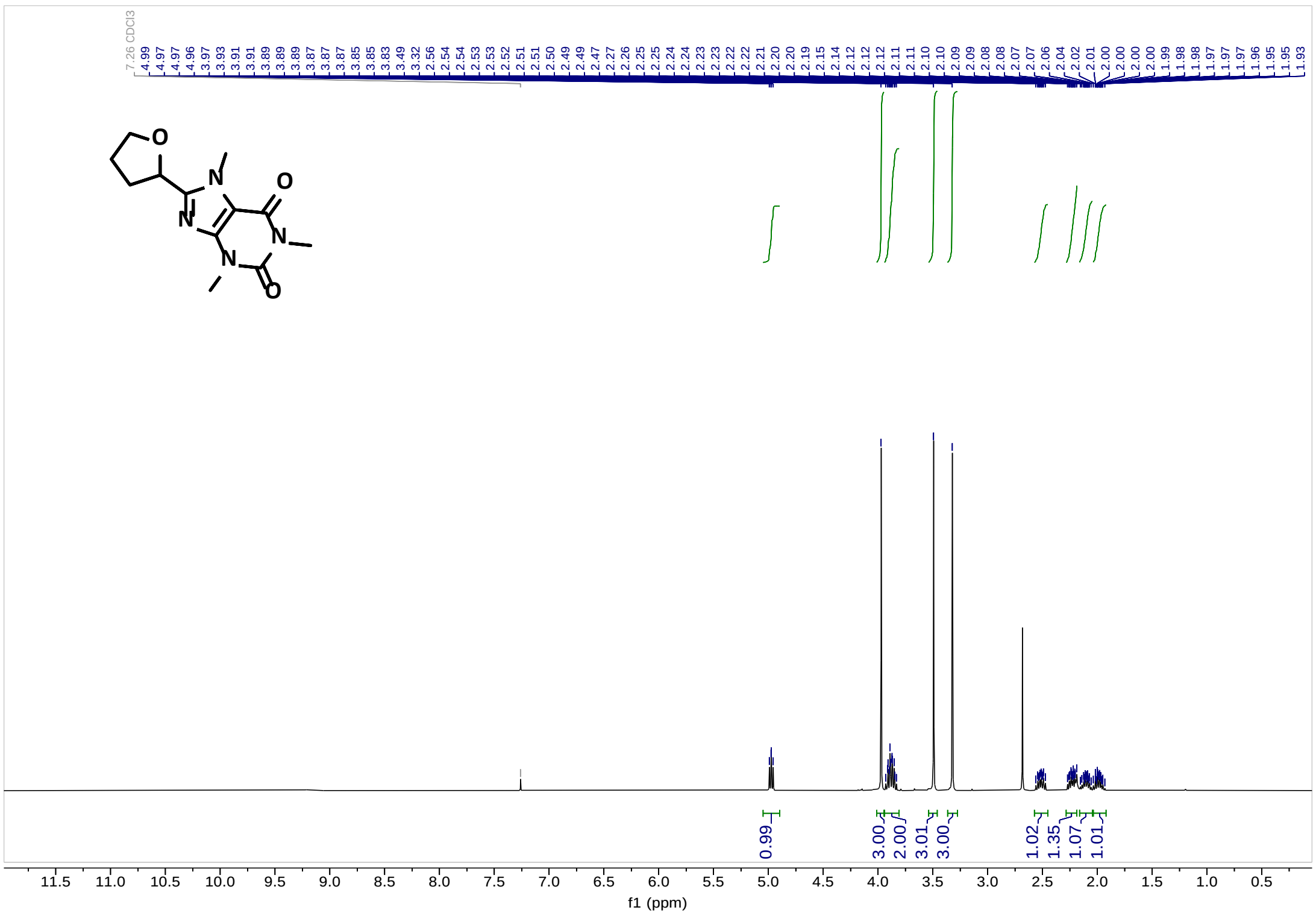

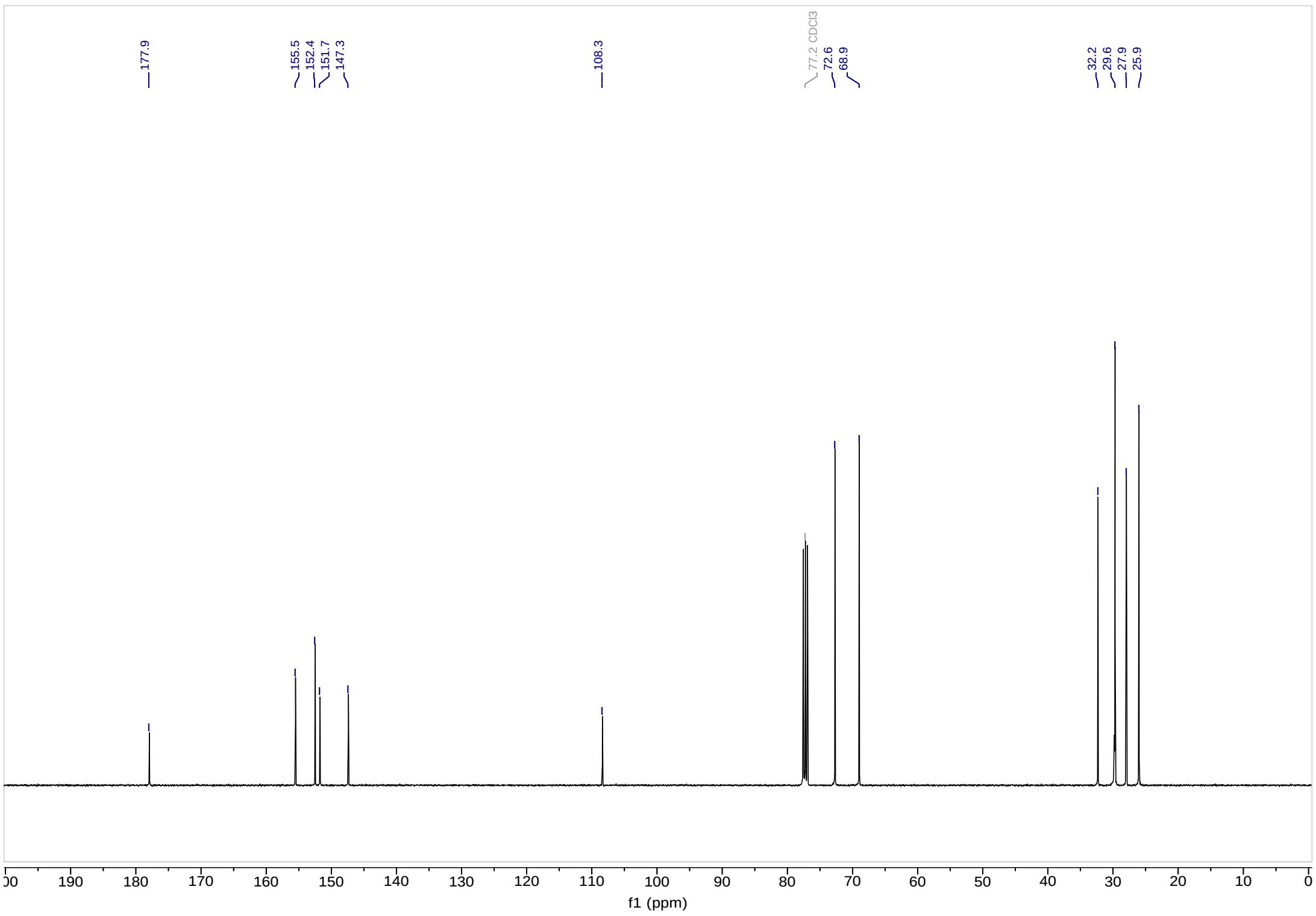

isopropyl 2-methyl-2-(4-(4-(tetrahydrofuran-2-yl)benzoyl)phenoxy)propanoate (3ap)

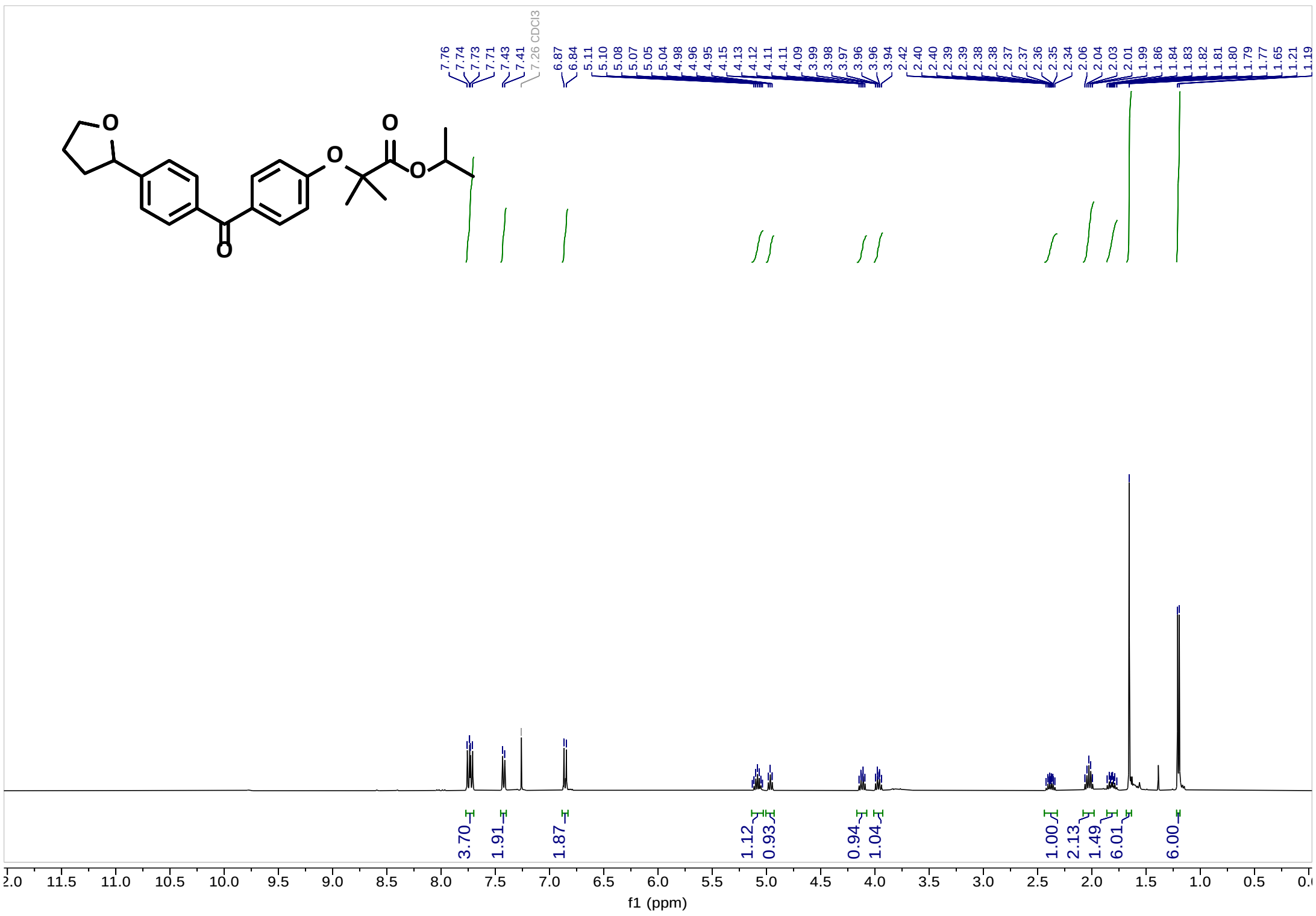

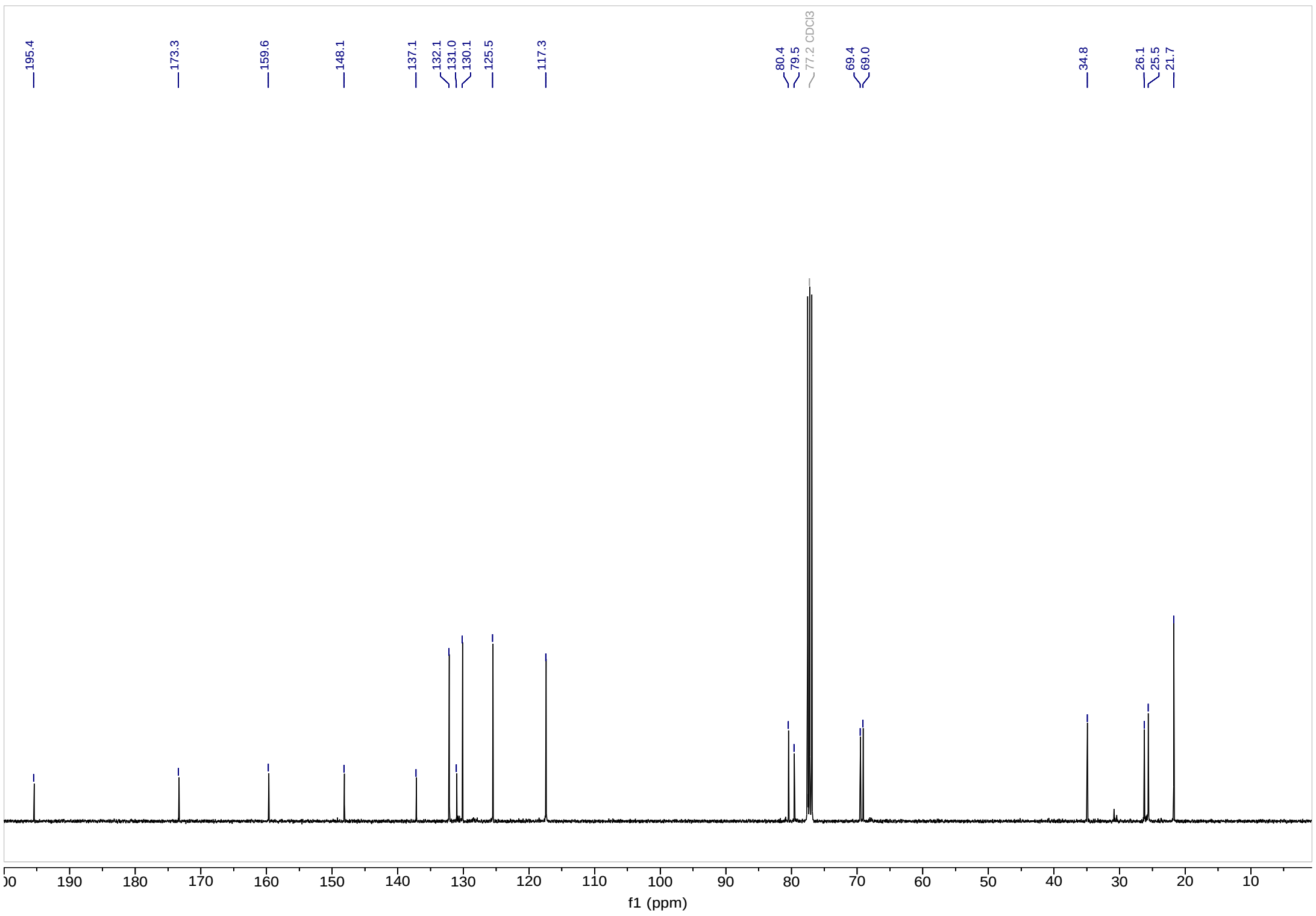

References of the Supporting Information

(1) Ueno, R.; Ikeda, Y.; Shirakawa, E. Tert-Butoxy-Radical-Promoted α-Arylation of Alkylamines with Aryl Halides. *Eur. J. Org. Chem.* **2017**, *2017* (28), 4188–4193.

(2) Gong, Y.; Su, L.; Zhu, Z.; Ye, Y.; Gong, H. Thermal Functionalization of Alkanes with Carbon Electrophiles. *Angew. Chem. Int. Ed.* **2022**, *61* (22), e202201662.

(3) Shar, M. H.; Shurtleff, V. W.; Terrett, J. A.; Cuthbertson, J. D.; MacMillan, D. W. C. Native Functionality in Triple Catalytic Cross-Coupling: sp^3 C–H Bonds Aslatent Nucleophiles. *Science* **2016**, *352* (6291), 1304–1308.

(4) Heitz, D. R.; Tellis, J. C.; Molander, G. A. Photochemical Nickel-Catalyzed C-H Arylation: Synthetic Scope and Mechanistic Investigations. *J. Am. Chem. Soc.* **2016**, *138* (39), 12715–12718.

(5) Shen, Y.; Gu, Y.; Martin, R. sp3 C-H Arylation and Alkylation Enabled by the Synergy of Triplet Excited Ketones and Nickel Catalysts. *J. Am. Chem. Soc.* **2018**, *140* (38), 12200–12209.

(6) Zhang, L.; Si, X.; Yang, Y.; Zimmer, M.; Witzel, S.; Sekine, K.; Rudolph, M.; Hashmi, A. S. K. The Combination of Benzaldehyde and Nickel-Catalyzed Photoredox $C(sp^3)$-H Alkylation/Arylation. *Angew. Chem. Int. Ed.* **2019**, *131* (6), 1837–1841.

(7) Maity, B.; Scott, T. R.; Stroscio, G. D.; Gagliardi, L.; Cavallo, L. The Role of Excited States of LNiII/III(Aryl)(Halide) Complexes in Ni-Halide Bond Homolysis in the Arylation of Csp^3-H Bonds. *ACS Catal.* **2022**, *12* (21), 13215–13224.

(8) Cusumano, A. Q.; Chaffin, B. C.; Doyle, A. G. Mechanism of Ni-Catalyzed Photochemical Halogen Atom-Mediated $C(sp^3)$-H Arylation. *J. Am. Chem. Soc.* **2024**, *146* (22), 15331–15344.

(9) Wang, Q. L.; Sun, Z.; Huang, H.; Mao, G.; Deng, G. J. Stoichiometric Couplings of Methylarenes through Visible-Light-Induced Bromo Radical Formation from Aryl Halides. *Green Chem.* **2022**, *24* (8), 3293–3299.

(10) Das, S.; Murugesan, K.; Villegas Rodríguez, G. J.; Kaur, J.; Barham, J. P.; Savateev, A.; Antonietti, M.; König, B. Photocatalytic (Het)Arylation of $C(sp^3)$-H Bonds with Carbon Nitride. *ACS Catal.* **2021**, *11* (3), 1593–1603.

(11) Frank, N.; Leutzsch, M.; List, B. Brønsted Acid-Catalyzed Reduction of Furans. *J. Am. Chem. Soc.* **2025**, *147* (9), 7932–7938.

(12) Hu, Y.; Wang, W.; Zhao, M.; Chen, J.; Kong, S.; Zhu, N.; Fang, Z.; Peng, Y.; Wu, Q.; Guo, K. Enantiocomplementary Synthesis of Tetrahydrofurans by Engineered Halohydrin Dehalogenases. *Adv. Synth. Catal.* **2024**, *366* (24), 5100–5107.

(13) Shields, B. J.; Doyle, A. G. Direct C(sp^3)-H Cross Coupling Enabled by Catalytic Generation of Chlorine Radicals. *J. Am. Chem. Soc.* **2016**, *138* (39), 12719–12722.

(14) Quan, Y.; Lan, G.; Fan, Y.; Shi, W.; You, E.; Lin, W. Metal-Organic Layers for Synergistic Lewis Acid and Photoredox Catalysis. *J. Am. Chem. Soc.* **2020**, *142* (4), 1746–1751.
